# Mechanically triggered on-demand degradation of polymers synthesized by radical polymerizations

**DOI:** 10.1038/s41557-024-01508-x

**Published:** 2024-04-12

**Authors:** Peng Liu, Sètuhn Jimaja, Stefan Immel, Christoph Thomas, Michael Mayer, Christoph Weder, Nico Bruns

**Affiliations:** 1 Adolphe Merkle Institute, University of Fribourg, Fribourg, Switzerland; 2 Swiss National Center of Competence in Research Bio-Inspired Materials, Fribourg, Switzerland; 3 Department of Chemistry and Centre for Synthetic Biology, University of Darmstadt, Darmstadt, Germany; 4 Waters GmbH, Eschborn, Germany; 5 Department of Pure and Applied Chemistry, University of Strathclyde, Glasgow, UK; 6 Present Address: Department of Materials, ETH Zürich, Zürich, Switzerland

**Keywords:** Polymer synthesis, Polymers, Chemical engineering, Polymer synthesis, Sustainability

## Abstract

Polymers that degrade on demand have the potential to facilitate chemical recycling, reduce environmental pollution and are useful in implant immolation, drug delivery or as adhesives that debond on demand. However, polymers made by radical polymerization, which feature all carbon-bond backbones and constitute the most important class of polymers, have proven difficult to render degradable. Here we report cyclobutene-based monomers that can be co-polymerized with conventional monomers and impart the resulting polymers with mechanically triggered degradability. The cyclobutene residues act as mechanophores and can undergo a mechanically triggered ring-opening reaction, which causes a rearrangement that renders the polymer chains cleavable by hydrolysis under basic conditions. These cyclobutene-based monomers are broadly applicable in free radical and controlled radical polymerizations, introduce functional groups into the backbone of polymers and allow the mechanically gated degradation of high-molecular-weight materials or cross-linked polymer networks into low-molecular-weight species.

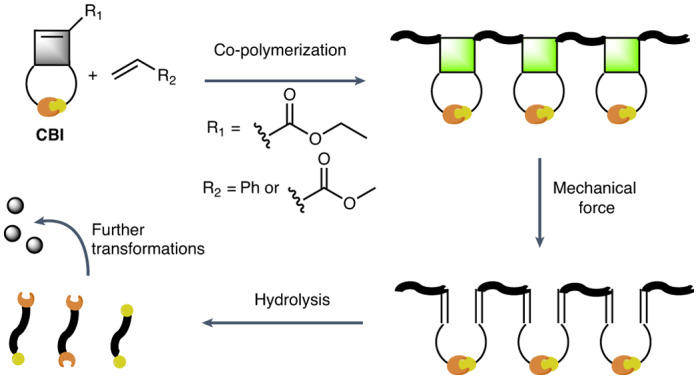

## Abstract (web summary)

Radical polymerizations yield polymers that cannot easily be degraded. The co-polymerization of cyclobutene-based monomers with conventional vinyl monomers has now been shown to result in co-polymers with cyclobutane mechanophores in their backbone, which facilitate on-demand degradation through a combination of mechanical activation and hydrolysis. This approach offers a promising avenue for the degradation of all-carbon-bond-backbone polymers.

## Main

**Fig. 1:**
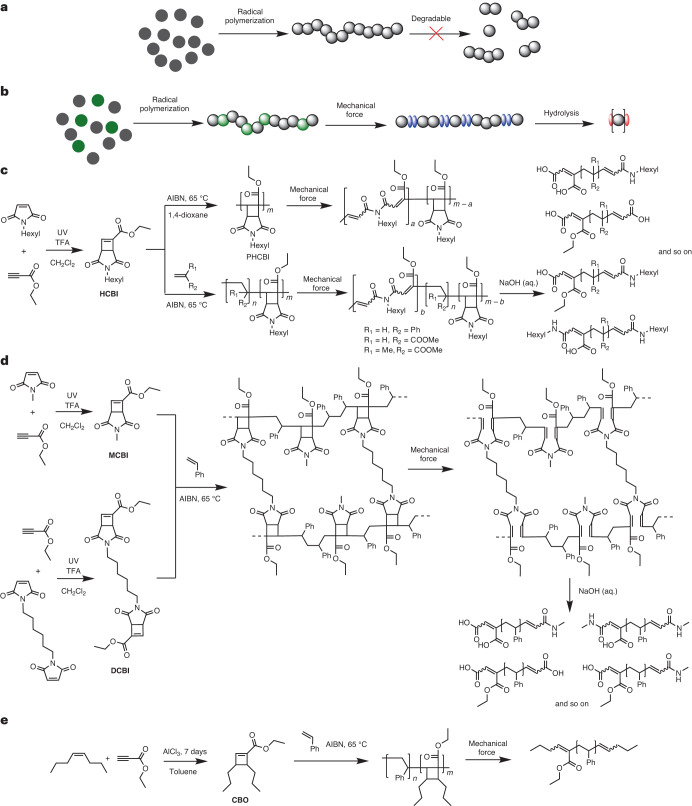
Concept and reaction schemes of the synthesis and degradation of cyclobutene imide-based and cyclobutene carboxylate-based co-polymers and polymer networks. **a**, Schematic representation of conventional C–C main-chain polymers made by radical polymerization that are not degradable under hydrolytic conditions. **b**, Schematic representation of polymers whose propensity to degrade under basic conditions is mechanically activated. Grey spheres, conventional monomers for radical polymerizations; green spheres, cyclobuten imide-based monomers that can be mechanically activated in the polymer backbone; blue spheres, hydrolyzable imide units in the polymer backbone; red spheres, end-groups of degraded, low-molecular-weight species. **c**, Synthesis of HCBI, radical homo- and co-polymerization of HCBI, force-induced rearrangement of cyclobutane rings to introduce imide groups into the polymer backbone and co-polymer degradation to small molecules after treatment with 1.0 M NaOH (aq.). **d**, Synthesis of MCBI, the cross-linker DCBI and polymer networks (PS-*co*-PMCBI)-*l*-PDCBI, as well as force-induced rearrangement of cyclobutane rings to introduce imide groups into the polymer backbone and degradation of the cross-linked polymer to small molecules after treatment with 1.0 M NaOH (aq.). **e**, Synthesis of the cyclobutene carboxylate monomer CBO, radical co-polymerization of CBO and styrene and degradation of the co-polymer to small molecules under grinding without hydrolysis. TFA, trifluoroacetic acid.

Most commercially exploited polymers are made by addition polymerizations of monomers featuring a C=C bond^1^. The most important products include polyolefins, such as polyethylene and polypropylene, vinyl polymers, such as polystyrene (PS) and polyvinyl chloride, and (meth)acrylates, such as poly(methyl acrylate) (PMA) and poly(methyl methacrylate) (PMMA), as well as co-polymers, such as styrene-butadiene rubber (SBR)^1^. The main chains of these polymers are all exclusively composed of carbon–carbon bonds and are, thus, highly resistant to degradation in a broad range of conditions^2,3^ (Fig. 1a). While high stability is an attractive feature for the service life of plastic products, it is also a key reason for their accumulation in the environment^4,5^.

In this context, polymers that degrade under a specific set of conditions represent a promising strategy^6,7^. Such materials have already been demonstrated to be useful for implant immolation^8^, drug delivery^9^, adhesives that debond on demand^10^, microelectronics^11^, signal amplification^12^ and other applications^13,14^. The chemical recycling of polymers through degradation into small molecules or oligomers is also receiving growing attention^15–17^ because the products can be used as fuels, additives or new monomers^18–20^. Moreover, low-molecular-weight products and short polymers show a higher biodegradability than high-molecular-weight polymers^21,22^. Degradability can a priori be achieved by modifying polymer backbones with functional groups that can be cleaved under certain conditions^23–25^. While this approach is relatively straightforward for polymers made by step-growth polymerizations, the introduction of cleavable groups into the C–C backbones of polymers made by chain polymerizations, such as radical polymerizations, is more challenging. So far, the most reliable method is the radical ring-opening polymerization of cyclic monomers that feature a carbon–carbon double bond^26–28^. However, radical ring-opening polymerization is often hampered by side reactions, such as ring retention, during the polymerization.

Independent of the polymerization mechanism, degradable polymers generally suffer from limited thermal and chemical stability, as the labile bonds may also cleave when this is not intended. To address this problem, the groups of Craig^29–31^, Wang^32^ and Xia^33^ introduced the general idea of mechanically gated degradable polymers, which only become degradable after mechanical activation. To achieve this function, the mechanophores made by fusing cyclobutanes with a hydrolysable cycle were (co)polymerized by ring-opening metathesis polymerization. The cyclobutane moieties can be ring opened upon application of mechanical force, so that the labile linkages become part of the polymer backbones and can then be cleaved under basic or acidic conditions. So far, however, this elegant approach has been limited to monomers that can be polymerized by ring-opening metathesis polymerization.

In this Article, we show that a similar approach can be used to bestow important commodity polymers, which are made by free radical polymerization, with on-demand degradability. To achieve this, we exploited that appropriately designed cyclobutenes can be polymerized using radical addition polymerizations^34–36^. We devised cyclobutene imide monomers (CBI) and cross-linkers that can be synthesized in one step from commercially available or easily accessible chemicals and demonstrated that these building blocks can be efficiently co-polymerized with styrene, methyl acrylate, methyl methacrylate and probably many other monomers by free radical polymerization and also reversible addition–fragmentation chain-transfer (RAFT) polymerization (Fig. 1). In response to mechanical activation, the CBI residues in the polymer backbone rearrange, and the imide groups are inserted into the C–C backbone of the polymers. These materials can further be degraded into small molecules and oligomers by hydrolysis of the imides under basic conditions. This feature is very useful in the context of mechanochemical recycling^37–39^ (for example, by ball milling) and could accelerate the degradation of such polymers upon release into seawaters, where they experience mechanical forces and basic conditions^40^.

## Results and discussion

### Polymer synthesis

Due to the stable π double bond, unsubstituted cyclobutene cannot be polymerized by radical polymerization under mild conditions. However, the introduction of electron-withdrawing substituents stabilizes the propagating radical and enables this process^34^. Building on this knowledge, we designed the monomer ethyl 3-hexyl-2,4-dioxo-3-azabicyclo[3.2.0]hept-6-ene-6-carboxylate (HCBI, Fig. 1c). HCBI features a cyclobutene ring that is activated by an ethyl carboxylate group and fused to a cyclic imide. It was synthesized in one step by the photochemical [2 + 2] cycloaddition of ethyl propiolate and *N*-hexyl maleimide (Fig. 1c). The structure and purity of the monomer were confirmed by ^1^H and ^13^C nuclear magnetic resonance (NMR) spectroscopy and by mass spectrometry (Supplementary Figs. 117 and 118).

To confirm that HCBI can be polymerized by free radical polymerization, we homopolymerized HCBI with azobisisobutyronitrile (AIBN) as the initiator in 1,4-dioxane at 65 °C. While the yield (5%), number-average molecular weight (*M*_n_ = 22 kg mol^−1^) and dispersity (*Đ* = 1.4) of the HCBI homopolymer (PHCBI) isolated after 72 h were relatively low, presumably due to the steric hindrance and high stability of the tertiary radicals^36^, the experiment confirms that free radical polymerization of HCBI is feasible.

**Fig. 2:**
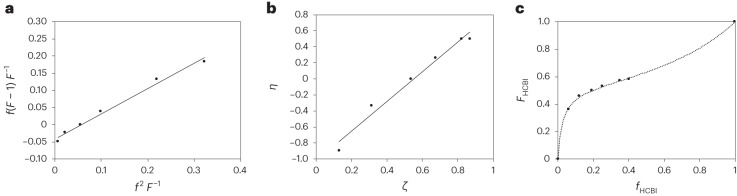
Determination of the co-polymerization parameters of HCBI and styrene. **a**–**c**, Plots used to determine the co-polymerization parameter (reactivity ratio) for the co-polymerization of HCBI and styrene by the methods of Fineman-Ross (**a**), Kelen-Tüdos (**b**) and Mayo-Lewis (**c**). The conditions include bulk polymerization and AIBN at 65 °C. All three methods yield co-polymerization parameters *r*_HCBI/styrene_ = 0.79 ± 0.3 and *r*_styrene/HCBI_ = 0.046 ± 0.05, indicating the formation of statistical co-polymers with a preference for the incorporation of HCBI over styrene into the growing polymer chain. The data are shown in Supplementary Table 1.

We next investigated the free radical co-polymerization of HCBI with styrene, which we selected as an industrially relevant example of a vinylic monomer^41^. The reactions were carried out in bulk at 65 °C with AIBN as the initiator and an initial HCBI fraction (*f*_HCBI_) of 0.14 in the monomer feed. After reaction for 60 h, the product (PS_55_-*co*-PHCBI_45_) was isolated by precipitation into cold methanol. Size exclusion chromatography (SEC) of the purified co-polymer shows an unimodal molecular weight distribution with an *M*_n_ = 38 kg mol^−1^ and *Đ* = 1.9 (Table 1). ^1^H NMR spectra exhibit the characteristic signals of both HCBI and styrene residues (Supplementary Fig. 1). The analysis of the ^1^H NMR data reveals a HCBI fraction in the co-polymer (*F*_HCBI_) of 0.45 (Supplementary Fig. 137), which suggests that the incorporation of HCBI is preferred over styrene. A similar preference can be observed for different HCBI fractions in the feed (*f*_HCBI_ = 0.02–0.69 resulting in *F*_HCBI_ = 0.18–0.62), although the difference between *f*_HCBI_ and *F*_HCBI_ decreases with increasing *f*_HCBI_ (Table 1). This trend agrees with the monomer reactivity ratio (see Fig. 2). Two-dimensional NMR spectroscopy experiments, specifically nuclear Overhauser effect spectroscopy and correlation spectroscopy, carried out with PS_55_-*co*-PHCBI_45_, show a strong correlation of signals, which suggests that the monomer distribution in the co-polymer is not blocky (Table 1 and Supplementary Figs. 2 and 3).

**Table 1:** Summary of synthesized HCBI homo and co-polymers, as well as reference polymers

Name	Polymerization Method^a^	Solvent	Reaction time (h)	Yield^b^	*f*_HCBI_^c^	*F*_HCBI_^d^	SEC		TGA *T*_d_ (°C)	DSC *T*_g_ (°C)	DMA	
							*M*_n_ (kg mol^−1^)	*Đ*			tan *δ* (°C)^e^	Storage modulus (MPa)^e,f^
PHCBI	FRP	1,4-Dioxane	72	5%	1.00	1.00	22	1.4	315	171	^–g^	^–g^
PS_82_-*co*-PHCBI_18_	FRP	–	60	12%	0.02	0.18	40	2.7	371	105	122 ± 2	1,265 ± 135
PS_75_-*co*-PHCBI_25_	FRP	–	60	16%	0.05	0.25	45	2.3	366	113	124 ± 4	1,155 ± 207
PS_60_-*co*-PHCBI_40_	FRP	–	60	20%	0.09	0.40	37	2.1	360	114	134 ± 5	1,128 ± 182
PS_55_-*co*-PHCBI_45_	FRP	–	60	23%	0.13	0.45	38	1.9	360	118	137 ± 6	1,205 ± 204
PS_50_-*co*-PHCBI_50_	FRP	–	24	37%	0.20	0.50	52	1.8	^–h^	^–h^	^–h^	^–h^
PS_49_-*co*-PHCBI_51_	FRP	–	24	28%	0.23	0.51	69	1.8	356	132	157 ± 2	870 ± 30
PS_45_-*co*-PHCBI_55_	FRP	–	24	37%	0.40	0.55	49	1.8	349	140	165 ± 3	749 ± 107
PS_38_-*co*-PHCBI_62_	FRP	–	24	38%	0.70	0.62	26	1.7	349	152	175 ± 1	575 ± 225
PS_73_-*co*-PHCBI_27_	RAFT	–	168	45%	0.10	0.27	39	1.3	366	102	125 ± 2	1,120 ± 382
PMA_87_-*co*-PHCBI_13_	FRP	–	15	30%	0.12	0.13	250	1.7	371	35	52 ± 2	1,045 ± 185
PMA_97_-*co*-PHCBI_3_	FRP	1,4-Dioxane	15	75%	0.02	0.03	220	1.6	373	20	32 ± 2	242 ± 134
PMMA_98_-*co*-PHCBI_2_	FRP	1,4-Dioxane	15	43%	0.02	0.02	300	1.6	313	128	146 ± 3	2579 ± 206
PS_34_-*co*-PB_49_-*co*-PHCBI_17_	FRP	THF	15	15%	0.05	34:49:17	40	1.6	371	14	30 ± 2	128 ± 130
PS^i^	–	–	–	–	–	–	45	1.03	395	104	121 ± 2	1,127 ± 434
PMA	RAFT	1,4-Dioxane	15	68%	–	–	250	1.7	372	19	30 ± 4	30 ± 19
PMMA	RAFT	1,4-Dioxane	15	35%	–	–	300	1.2	315	129	141 ± 2	2,637 ± 373
PS_47_-*co*-PB_53_ (SBR)	RAFT	THF	15	12%	–	47:53	36	1.6	370	10	21 ± 2	1.13 ± 0.59

^a^All free radical polymerizations (FRP) were carried out with AIBN as the initiator at 65 °C. All RAFT polymerizations were carried out with 4-cyano-4-(phenylcarbonothioylthio)pentanoic acid as the chain transfer agent and AIBN as the initiator at 65 °C

^b^Isolated yield after precipitation

^c^Fraction of HCBI in the monomer feed, determined by ^1^H NMR spectroscopy after degassing

^d^Fraction of HCBI in the isolated polymer, determined by ^1^H NMR spectroscopy

^e^Mean value ± standard deviation, *n* = 3

^f^At 25 °C

^g^No homogeneous film could be produced

^h^This sample was not characterized by TGA, DSC and DMA

^i^Commercial product

To develop a better understanding of the co-polymerization process and the reactivity of HCBI, we determined the co-polymerization parameters (monomer reactivity ratios) for HCBI and styrene. Thus, HCBI:styrene mixtures with *f*_HCBI_ = 0.06–0.40 were co-polymerized under bulk conditions at 65 °C and quenched at low conversion (<20% for each monomer) to limit the compositional drift. The composition of the co-polymers was determined by analysis of the ^1^H NMR spectra, and their *M*_n_ and *Đ* were determined by SEC (Supplementary Table 1). The reactivity ratios were determined by analyses according to Fineman-Ross^42^, Kelen-Tüdos^43^ and Mayo-Lewis^44,45^ (Fig. 2). The methods afford similar results, with average values of *r*_HCBI/styrene_ = 0.79 ± 0.3 and *r*_styrene/HCBI_ = 0.046 ± 0.05 (Supplementary Figs. 4–6). Since both co-polymerization parameters are below 1, the two monomers each preferably add to propagating centres formed by the other monomer, leading to statistical co-polymers. The data also reflect a higher reactivity of HCBI and the preferred accumulation of this monomer in the co-polymers.

Since reversible deactivation radical polymerizations can offer better control over the polymer molecular weight and microstructure, and yield polymers with lower *Đ* than free radical polymerizations^46–48^, we explored if HCBI can be co-polymerized with styrene by RAFT polymerization. Gratifyingly, the polymerization of styrene and HCBI (*f*_HCBI_ = 0.10) with the chain transfer agent 4-cyano-4-(phenylcarbonothioylthio)pentanoic acid and AIBN as initiator at 65 °C in bulk afforded a co-polymer with *M*_n_ = 34 kg mol^−1^ and *Đ* = 1.3 (PS_73_-*co*-PHCBI_27_) (Table 1). The *M*_n_ was lower than the theoretical value (*M*_n(theo.)_ = 120 kg mol^−1^) which, based on earlier studies^36^, we relate to the stable nature of the tertiary radical and the low C–H bond dissociation energy in HCBI. Nonetheless, HCBI can indeed be co-polymerized with styrene by RAFT polymerization, and the dispersity of the resulting co-polymer is lower than that of the corresponding material made by free radical polymerization.

To demonstrate versatility, we explored the possibility to co-polymerize HCBI with methyl acrylate and methyl methacrylate. With *f*_HCBI_ = 0.02, the free radical co-polymerization of HCBI with methyl acrylate afforded a co-polymer with *M*_n_ = 220 kg mol^−1^, *Đ* = 1.6 and *F*_CBI_ = 0.03. Increasing *f*_HCBI_ to 0.12 afforded a co-polymer with *M*_n_ = 250 kg mol^−1^, *Đ* = 1.7 and *F*_HCBI_ = 0.13 (Table 1). The same protocol was applied to co-polymerize HCBI with methyl methacrylate, and with *f*_HCBI_ = 0.02, a co-polymer with *M*_n_ = 300 kg mol^−1^, *Đ* = 1.6 and *F*_HCBI_ = 0.02 was obtained. Finally, we also co-polymerized HCBI with styrene and 1,3-butadiene (molar ratio of the monomer, 4:48:48) in tetrahydrofuran (THF) by free radical polymerization. The HCBI-functionalized SBR thus made PS_34_-*co*-PB_49_-*co*-PHCBI_17_, characterized by an *M*_n_ = 40 kg mol^−1^ and *Đ* = 1.6 (Table 1), and ^1^H NMR spectra reveal a composition of HCBI:styrene:1,3-butadiene of 17:34:49 (Supplementary Fig. 165).

### Thermal and mechanical properties of polymers

**Fig. 3:**
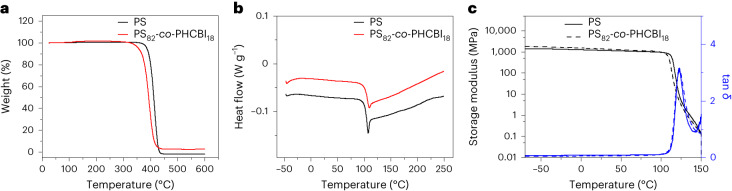
Thermal and mechanical characterization of PS_82_-*co*-PHCBI_18_ and PS. **a**, TGA showing a slightly lower *T*_d_ of the co-polymer compared with the PS homopolymer. **b**, DSC showing the same glass transition temperature for the co-polymer and the PS homopolymer. **c**, DMA showing that the storage modulus E′ and the ratio of loss and storage moduli (tan *δ*), which corresponds to the glass transition temperature are similar for this co-polymer and the PS homopolymer.

The thermal and mechanical properties of PHCBI and HCBI-containing co-polymers and of neat PS, PMA, PMMA and SBR reference polymers were characterized by thermogravimetric analysis (TGA), differential scanning calorimetry (DSC) and dynamic mechanical analyses (DMA) (Table 1). The TGA data show that the decomposition temperature (*T*_d_) of PHCBI (315 °C) is lower than that of the PS (395 °C, Fig. 3), PMA (372 °C) and SBR (370 °C) homopolymers. Consequently, the *T*_d_ of the co-polymers is also lower than that of the homopolymers (Fig. 3a), decreasing with increasing HCBI content (Supplementary Figs. 8–14). Similar *T*_d_ values were measured for PMMA-*co*-PHCBI and PMMA (Table 1 and Supplementary Figs. 18 and 22).

All DSC thermograms show exclusively one glass transition, which demonstrates that all materials are, as expected, fully amorphous (Fig. 3b and Supplementary Figs. 7–23). The glass transition temperature (*T*_g_) of PHCBI (171 °C) is higher than that of the neat PS (104 °C), and consequently, the *T*_g_ of PS-*co*-PHCBI increases with the HCBI content. However, for a HCBI fraction of 0.18, the *T*_g_ increase is negligible (Table 1 and Fig. 3b). No obvious *T*_g_ change, relative to the neat PMMA, is observed for PMMA_98_-*co*-PHCBI_2_ with a HCBI fraction of 0.02 (Table 1 and Supplementary Figs. 18 and 22). In the case of PMA and SBR, which have lower *T*_*g*_ values than PS and PMMA, the *T*_g_ increase is more pronounced (Table 1). Interestingly, an exothermic event can be observed in the first DSC heating thermogram of PHCBI just above *T*_g_ (Supplementary Fig. 7), which might be related to the thermally activated ring opening of the cyclobutane. Indeed, a ^1^H NMR spectrum recorded after heating PHCBI for 30 min under Ar at 180 °C shows considerable changes, including the appearance of weak signals that are consistent with newly formed C=C double bonds (Supplementary Fig. 40). However, the DSC thermograms of the co-polymers are devoid of exothermic signals (Supplementary Figs. 8–19), and the ^1^H NMR spectrum recorded after heating PS_49_-*co*-PHCBI_51_ for 30 min under Ar at 180 °C (Supplementary Fig. 41) shows no change in comparison to the as-prepared materials, which suggests that the co-polymers have a higher thermal stability than the PHCBI homopolymer.

The DMA data show that the storage moduli (*E*′) of the PS-*co*-PHCBI samples at 25 °C are statistically indifferent from the value of the neat PS control (1,127 MPa) up to *F*_HCBI_ = 0.45 (Table 1 and Fig. 3c). At higher HCBI contents, *E*′ drops and reaches 575 MPa at *F*_HCBI_ = 0.62 (Table 1). The maxima of the ratio of loss and storage moduli (tan *δ*), which mark the *T*_g_, of PS_82_-*co*-PHCBI_18_ and PS are the same (122 °C, Fig. 3c). The temperature of the tan *δ* peak increases to 175 °C with increasing HCBI content, that is, the trend is the same as established by DSC. The same observations, within the margin of error, are also made for PMMA-*co*-PHCBI and PMMA (Table 1 and Supplementary Figs. 34 and 38). In the case of PMA (Supplementary Figs. 32, 33 and 37) and PS-*co*-PB (Supplementary Figs. 35 and 39), the *E*′ of the co-polymers at 25 °C is influenced in a more pronounced manner by the incorporation of HCBI. The neat PMA and PS-*co*-PB both have a *T*_g_ just below room temperature, and these materials are therefore rubbery at ambient temperature, with *E*′ values of 30 and 1 MPa, respectively. The incorporation of HCBI brings the *T*_g_ closer to, or above, ambient temperature, and the related transformation from a rubbery to a glassy state is reflected by an increase of *E*′. This effect is particularly pronounced in PMA_87_-*co*-PHCBI_13_, which at room temperature exhibits an *E*′ that is 30-fold higher than that of the neat PMA (Table 1). However, at −20 °C and 75 °C, that is, at temperatures where both polymers are well below or above *T*_g_, the two materials display the same stiffness. Thus, overall, the thermal and mechanical data show that the incorporation of moderate amounts of HCBI impacts the thermomechanical properties of the polymers only in very moderate ways.

### Polymer degradation

**Fig. 4:**
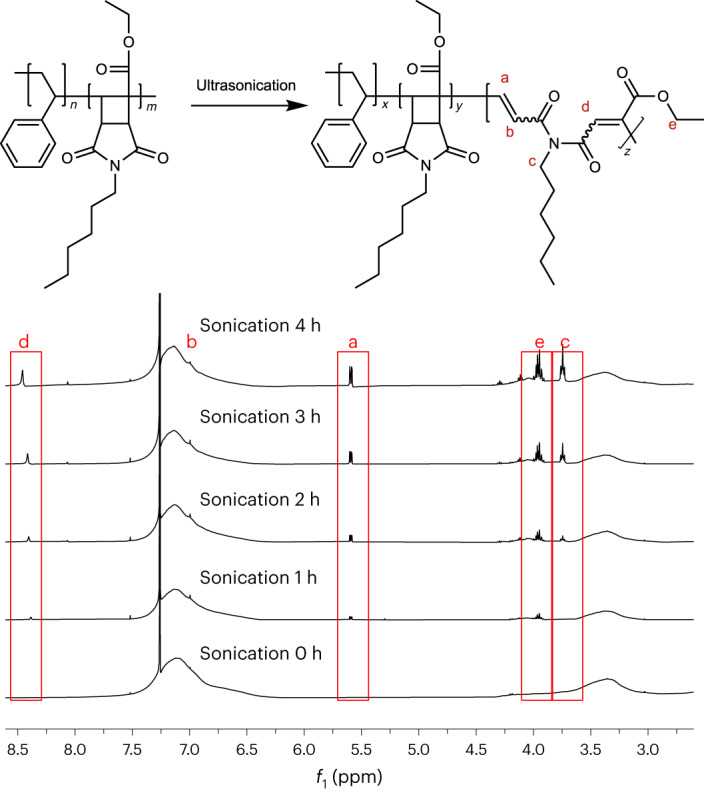
Mechanical conversion of PS_50_-*co*-PHCBI_50_ to a degradable polymer upon ultrasonication in THF. Reaction scheme and ^1^H NMR spectra documenting the mechanochemical conversion of PS_50_-*co*-PHCBI_50_ to polymers containing imide groups in their backbone after different sonication times. The new signals in the red boxes of the NMR spectra indicate carbon–carbon double bond formation associated with cyclobutane ring opening. The full NMR spectra are provided as Supplementary Figs. 44–46.

The mechanochemical behaviour of the HCBI-containing co-polymers was first investigated by way of ultrasonication of selected samples, which is a reliable and convenient method to explore mechanically induced bond scission event in polymers^49^. ^1^H NMR spectra acquired after ultrasonication of PS_50_-*co*-PHCBI_50_ in THF show four clear new signals (Fig. 4). We assign the signals at 8.46 and 5.60 ppm to the alkene groups formed upon the cyclobutane ring opening (H_d_ and H_a_), while the signal of another alkene proton (H_b_) appears to be largely hidden under the aromatic signals. The peak at 3.96 ppm can be assigned to the CH_2_ group of the acrylic ester formed upon ring opening (H_e_), and the signal around 3.75 ppm (H_c_) is assigned to the CH_2_ group next to acrylic amide formed upon ring opening. The intensity of all four signals increases with sonication time (Fig. 4). After 240 min of sonication, 12% of the cyclobutane rings opened according to the analysis of relevant integrals of the ^1^H NMR spectrum (Supplementary Figs. 44 and 45, see also Fig. 4). To further confirm the formation of carbon–carbon double bonds, we acquired Fourier-transform infra-red spectra of PS_49_-*co*-PHCBI_51_ before and after sonication. Signals between 1,600 and 1,700 cm^−1^ that appear upon sonication indicate the formation of carbon–carbon double bonds (Supplementary Fig. 47). No changes of the ^1^H NMR and IR spectra are seen in a control experiment in which neat PS was sonicated under the same conditions, confirming that the spectral changes observed in the co-polymers are indeed caused by the mechanochemical opening of the cyclobutane ring (Supplementary Figs. 47–49).

To shed more light onto the mechanism of the mechanochemical transformation of PS-*co*-PCBI, computational studies were carried out. The constrained geometries simulate external force (CoGEF)^50^ analysis of PS-*co*-PMCBI polymer models, as well as relaxed electronic transition states that were identified from unrestrained density functional theory calculations, suggest that the opening of the cyclobutane ring proceeds by a radical stepwise mechanism via initial diradical formation, which is in accord with the findings by Boulatov and coworkers^51^ (Supplementary Information, CoGEF).

The SEC chromatograms recorded after ultrasonicating solutions of the various co-polymers reveal molecular weight reductions that agree with previous investigations on ultrasound-induced polymer chain cleavage^29,32,33^ (Fig. 5a and Supplementary Figs. 50–68) and suggest that in addition to the intended mechanophore activation, some unspecific chain scission takes place. For example, the *M*_n_ of PS_49_-*co*-PHCBI_51_ decreased from 69 to 30 kg mol^−1^ after 240 min of sonication. Reference experiments with neat PS (Fig. 5b) also show some unspecific chain scission. Similar results were observed for other CBI-co-polymers and their reference polymers (Supplementary Figs. 69–83).

**Fig. 5:**
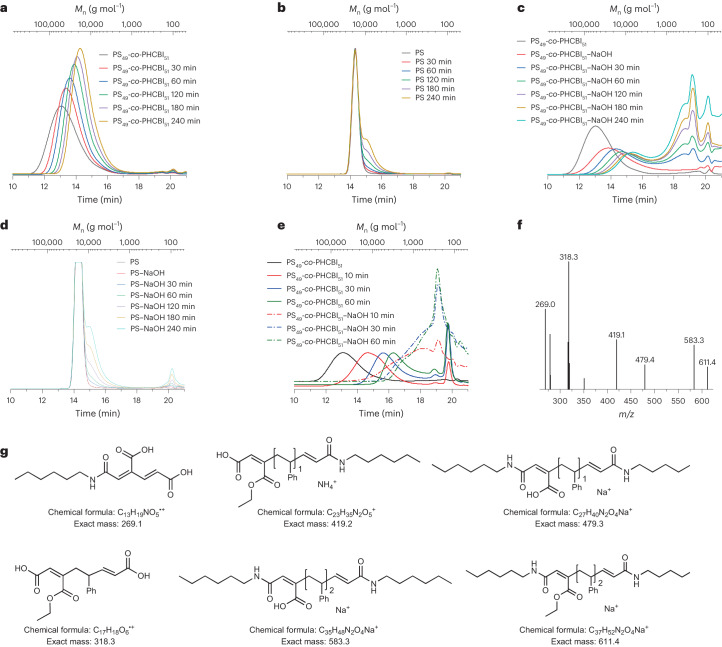
Polymer degradation by mechanical force and basic hydrolysis. **a**–**e**, SEC chromatograms of the mechanically gated degradable co-polymer PS_49_-*co*-PHCBI_51_ and unmodified PS in response to ultrasound or cryo-milling and NaOH treatment: PS_49_-*co*-PHCBI_51_ after different sonication times (**a**), PS after different sonication times (**b**), PS_49_-*co*-PHCBI_51_ hydrolysed with NaOH after different sonication times (**c**), PS treated with NaOH after different sonication times (**d**) and PS_49_-*co*-PHCBI_51_ treated by cryo-milling as well as cryo-milling and hydrolysis with NaOH (**e**). The differential refractive index (dRI) signal is shown in **a** and **b** and the UV signal is shown in **c**–**e**. **f**, Electrospray ionization mass spectrometry of PS_50_-*co*-PHCBI_50_ degradation products after ultrasonication and hydrolysis. **g**, Chemical structures and calculated molar masses (g mol^−1^) of possible degradation products.

To explore if the imide groups, which become part of the co-polymer backbones upon mechanical activation, can be hydrolytically cleaved, the sonicated polymer solutions were treated with 1.0 M aqueous NaOH at room temperature overnight. The SEC chromatogram of PS_49_-*co*-PHCBI_51_ thus treated exhibits several signals with long retention time and a nominal *M*_n_ below 1,000 g mol^−1^ (Fig. 5c). No such fragmentation is observed when the neat PS control polymer, sonicated or not, is exposed to the same hydrolysis conditions (Fig. 5d), which supports the conclusion that in the case of PS_49_-*co*-PHCBI_51_, the molecular weight decrease is indeed caused by the sequential mechanoactivation and imide hydrolysis. This is further supported by the observations that the small molecule fraction after base treatment of PS_49_-*co*-PHCBI_51_ increases with the sonication time (Fig. 5c) and that the molecular weight of the smallest fragments decreases with the concentration of the NaOH solution. Similar results were observed for the other co-polymers. In all cases, the small molecule fractions increase with sonication time and base treatment time (Supplementary Figs. 51–76).

All SEC chromatograms of HCBI-containing co-polymers that were sonicated and subsequently exposed to hydrolytic conditions show that some polymeric fragments (*M*_n_ ≈ 10 kg mol^−1^) persist (Fig. 5c and Supplementary Figs. 52–76), arguably because the mechanophores placed away from the centre of the polymer chains do not experience a sufficiently high mechanical force to allow the cyclobutane rings to open^52,53^. We, thus, subjected dry PS_49_-*co*-PHCBI_51_ to cryogenic grinding, as this process has been shown to exert considerable mechanical forces to all positions along the macromolecules^54^. The SEC chromatograms recorded after 60 min of cryo-milling (Fig. 5e) reveal a considerable reduction of *M*_n_ from 69 to 5 kg mol^−1^, on account of mechanically induced chain scission. Upon hydrolysis of this sample with NaOH, the *M*_n_ decreases further. The SEC chromatograms reveal signals associated with low-molecular-weight products with a broad distribution and a nominal *M*_n_ of below 1,000 g mol^−1^ (Fig. 5e). In the case of the neat PS control polymer, some random chain scission was observed after cryo-milling, but upon hydrolysis of this sample with NaOH, no further reduction in *M*_n_ was observed (Supplementary Fig. 85). Chromatographic separations of the products that resulted from the cryo-milling-hydrolysis and sonication-hydrolysis sequences of PS_50_-*co*-PHCBI_50_ revealed mostly oligomers and low-molecular-weight products (95%, *M*_n_ < 1 kg mol^−1^) for the former and polymeric fragments (34%, *M*_n_ = 10 kg mol^−1^) and degraded low-molecular-weight products (58%, *M*_n_ < 1 kg mol^−1^) for the latter sequence (Supplementary Figs. 100 and 101). Thus, cryo-milling followed by base treatment converts the co-polymer into much smaller fragments than ultrasonication, hydrolysis or cryogenic milling alone.

To compare the efficiency of the different methods to apply mechanical force, PS_60_-*co*-PHCBI_40_ was mechanically activated by ultrasonication, cryo-milling and ball milling, the latter method being widely considered a viable process in the context of polymer recycling^37–39^. The SEC traces recorded after subsequent hydrolysis in 1.0 M NaOH (aq.) show a bimodal distribution with *M*_n_ values of 15 kg mol^−1^ and 3 kg mol^−1^ for the ultrasonicated sample and monomodal distributions for the ball-milled (*M*_n_ = 8 kg mol^−1^) and the cryo-milled (*M*_n_ = 2 kg mol^−1^) samples (Supplementary Figs. 93–95). These results demonstrate that PS-*co*-PHCBI can be activated by different mechanical methods. The control experiments with the neat PS show that ball milling also caused some random chain scission, but again, subsequent hydrolysis caused no further *M*_n_ reduction (Supplementary Fig. 96).

To simplify the degradation process and make it more practical, dry powder of the co-polymer PS_50_-*co*-PHCBI_50_ was ball-milled together with solid NaOH in the absence of any solvent^55^. After 60 min, the SEC showed almost complete degradation of the co-polymer while in the case of ball milling a neat PS control under these conditions, only some random chain scission was observed (Supplementary Figs. 97 and 98).

To confirm the proposed degradation mechanism as well as the chemical identity of the low-molecular-weight degradation products, mixtures produced by treating PS_50_-*co*-PHCBI_50_ with ultrasound (4 mg ml^−1^ in THF for 240 min) or cryo-milling (60 min), followed by base hydrolysis (1.0 M NaOH (aq.)) were analysed by mass spectrometry (Fig. 5f and Supplementary Figs. 103 and 104) and ultraperformance liquid chromatography coupled to a hybrid quadrupole, orthogonal time-of-flight high-resolution mass spectrometer (Supplementary Figs. 105–109 and Supplementary Tables 2 and 3). Several small molecules could be identified using the accurate mass information, which correspond to degradation products expected from the sequence of cyclobutene ring opening and imide hydrolysis (Supplementary Fig. 102)^56^.

On-demand degradation is particularly important for cross-linked polymers, which are insoluble and constitute a particularly poorly degradable class of polymers^57,58^. To test if the approach presented here can be used to accelerate the degradation of such networks, a cross-linked PS-*co*-PMCBI was synthesized by co-polymerizing styrene, the monofunctional CBI derivative ethyl 3-methyl-2,4-dioxo-3-azabicyclo[3.2.0]hept-6-ene-6-carboxylate (MCBI, *f*_MCBI_ = 0.15) and the difunctional CBI-containing cross-linker diethyl 3,3’-(hexane-1,6-diyl)bis(2,4-dioxo-3-azabicyclo[3.2.0]hept-6-ene-6-carboxylate) (DCBI, *f*_DCBI_ = 0.05) (Fig. 1d). To increase the monomer diversity, MCBI was used for these materials. The polymer network thus made features degradable CBI residues in the chain segments and in the cross-links. Gratifyingly, cryo-milling (60 min) and hydrolysis (overnight, 1.0 M NaOH (aq.)) transformed the initially insoluble cross-linked (PS-*co*-PMCBI)-*l*-PDCBI into soluble low-molecular-weight products, which cannot be achieved by treatment of commercially available cross-linked PS as control under the same conditions (Supplementary Figs. 90–92).

Finally, we explored if cyclobut-1-ene-1-carboxylate motifs without the imide ring can be co-polymerized in a similar manner and if polymers containing such motifs can be mechanically cleaved without the need for subsequent hydrolysis. Thus, we synthesized ethyl-3,4-dipropylcyclobut-1-ene-1-carboxylate (CBO, Fig. 1e)^59^ and successfully co-polymerized this monomer with styrene (PS_65_-*co*-PCBO_35_, *f*_CBO_ = 0.25, *M*_n_ = 26 kg mol^−1^ and *Đ* = 2.2). After cryo-milling the resulting polymer, the fragments with an *M*_n_ = 3.5 kg mol^−1^ were observed in the SEC traces (Supplementary Fig. 89). The *M*_n_ of these species is comparable with that of the degradation products obtained after cryo-milling and hydrolysis of PS_60_-*co*-PHCBI_40_ (Supplementary Fig. 94), which suggests that the mechanically induced ring opening in CBO and HCBI residues is similar.

## Conclusions

In summary, we report new monomers that can be (co)polymerized by radical polymerizations and allow the incorporation of mechanoresponsive cyclobutene residues into the resulting polymers. The cyclobutene motif can be fused to a second ring, such as the imide that was employed in the present work. The force-activated ring-opening reaction of the residues of such bicyclic monomers causes a rearrangement that places the imide groups into the polymer backbone. Since the imide can be cleaved by hydrolysis under basic conditions, polymers containing the specific motifs studied here can be degraded on demand, that is, under conditions that combine mechanical forces and basic pH. This degradation pathway is absent in conventional polymers that feature backbones that exclusively contain C–C bonds. Thus, CBI-containing materials could become important for the mechanochemical recycling of polymers. Moreover, they should degrade faster than conventional polymers in environmental conditions that combine mechanical forces and basic pH, such as sea water, leading to polymer fragments, oligomers and small molecules that should be more prone to biodegradation. Finally, the specific chemical structure of the cyclobutene monomers, including the nature of the activating group and the latent labile group in the second ring, can be readily varied to prepare monomers that allow introducing mechanically responsive residues that can be transformed into degradable groups upon activation and rearrangement, or that cleave directly upon mechanoactivation. Thus, we envision that the properties of co-polymers that can be accessed through the presented approach can be further tailored for applications as degradable plastics, on-demand polymer cleavage or debonding on demand in polymer adhesives.

## Methods

### Synthesis of cyclobutene-based monomers

HCBI, MCBI and DCBI were synthesized as following procedure. Maleimide, ethyl propiolate and trifluoroacetic acid were dissolved in methylene chloride in a flask. After sparging the solution for 30 min with N_2_, the flask was sealed by septum and stirred under ultraviolet (UV) irradiation (mercury lamp, 400 W) for 48 h. After completion of the reaction (thin-layer chromatography monitoring), the solvent was removed under vacuum. The crude residue was purified by silica column chromatography to afford the product. CBO was synthesized as following procedure. Anhydrous aluminium chloride (1.79 g, 13.4 mmol and 0.5 equiv.) was charged to a 100 ml flame-dried round bottom flask under N_2_, followed by 20 ml anhydrous toluene. Ethyl propiolate (3.1 g, 32.1 mmol and 1.2 equiv.) was then added dropwise, followed by (*Z*)-oct-4-ene (3 g, 26.7 mmol and 1.0 equiv.). The reaction was stirred for 7 days at room temperature, then poured into an aqueous solution of KH_2_PO_4_ (0.2 M and 60 ml) with stirring, resulting in the formation of a white precipitate. A total of 20 ml of a 10% HCl solution was added to dissolve the precipitate. The aqueous layer was extracted with 3 × 20 ml portions of diethyl ether. The combined organic layers were washed with brine and dried over MgSO_4_. The solvent was removed under reduced pressure. The crude residue was purified by silica column chromatography (0–20% ethyl acetate/hexane) to afford the product as a colourless liquid.

#### Linear co-polymer synthesis

HCBI or CBO, comonomer (styrene, methyl acrylate, methyl methacrylate or a mixture of styrene and 1,3-butadiene) and AIBN were added into a Schlenk flask. The mixture was degassed by three freeze–pump–thaw degassing cycles before taking 0.1 ml solution for ^1^H NMR measurement to determine the monomer feed. After stirring for defined times at 65 °C under Ar, the reaction solution was cooled to room temperature and precipitated into cold methanol. The precipitate was filtered off and dried under vacuum to isolate the co-polymer. Experimental details can be found in Supplementary Information.

#### Polymer degradation by ultrasound, followed by hydrolysis

For a typical pulsed ultrasonication experiment, a solution of co-polymer (80 mg polymer in 20 ml THF) was placed in a Suslick reaction vessel and sparged with N_2_ for 15 min before sonication. Pulsed ultrasound (1 s on, 1 s off at 20% amplitude) was applied with a Branson 450 digital sonifier using a 13 mm tip while the solution was kept at 0 °C. At each sonication time (0, 30, 60, 120, 180 and 240 min), an aliquot (1.5 ml) was taken from the solution and directly subjected to SEC analysis. After SEC measurement, 150 μl NaOH aqueous solution (1 M or 0.5 M for PMA) was added into the aliquot. After stirring overnight (0.5 h for PMA), the NaOH treated solution was further analysed by SEC directly.

### Polymer degradation by cryo-milling and ball milling, followed by hydrolysis

PS, PS-*co*-PHCBI, PS-*co*-PCBO, (PS-*co*-PMCBI)-*l*-PDCBI or cross-linked PS was placed in a cryo-milling or ball milling machine. After milling, a sample (2 mg) was taken and dissolved in 1.5 ml THF, filtered through a syringe filter (0.22 µm) and then directly subjected to SEC analysis. Another 2 mg sample was taken and dissolved in 1.5 ml THF. Then, 150 μl NaOH solution (1 mol l^−1^) was added into the polymer solution. After stirring overnight at room temperature, the NaOH treated solution was further analysed by SEC.

### Polymer degradation by ball milling of dry polymers with solid NaOH

A total of 300 mg PS_50_-*co*-PHCBI_50_ (or PS) and 300 mg of solid NaOH were placed in a 10 ml top-screwed stainless steel grinding jar with stainless steel grinding balls with a diameter of 2 mm. The polymer was ground for 60 min with a 30 Hz frequency. Thereafter, a sample (2 mg) was taken and dissolved in 1.5 ml THF, filtered through a syringe filter (0.22 µm) and then directly subjected to SEC analysis.

## Online content

Any methods, additional references, Nature Portfolio reporting summaries, source data, extended data, supplementary information, acknowledgements, peer review information; details of author contributions and competing interests; and statements of data and code availability are available at 10.1038/s41557-024-01508-x.

### Supplementary information

Supplementary Information Experiment details, supporting data (TGA, DSC and DMA), CoGEF calculation and NMR spectra.

## Data Availability

The datasets generated and analysed during the current study are available in the Figshare repository at 10.6084/m9.figshare.21646715 (ref. ^60^).
