# Supplementary material for: Mechanically triggered on-demand degradation of polymers synthesized by radical polymerizations
Source: Nat Chem. 2024 Apr 12;16(7):1184–92. doi: 10.1038/s41557-024-01508-x (PMC11230896; doi:10.1038/s41557-024-01508-x)
Supplement: Supplementary file 1 — Experiment details, supporting data (TGA, DSC and DMA), CoGEF calculation and NMR spectra. [file 41557_2024_1508_MOESM1_ESM.pdf]

nature chemistry

Article

https://doi.org/10.1038/s41557-024-01508-x

Mechanically triggered on-demand degradation of polymers synthesized by radical polymerizations

In the format provided by the authors and unedited

Table of Contents

Experimental Section *3*

Materials 3

Instrumentation 3

Synthesis of ethyl 3-hexyl-2,4-dioxo-3-azabicyclo[3.2.0]hept-6-ene-6-carboxylate (HCBI) 6

Synthesis of ethyl 3-methyl-2,4-dioxo-3-azabicyclo[3.2.0]hept-6-ene-6-carboxylate (MCBI) 6

Synthesis of diethyl 3,3'-(hexane-1,6-diyl)bis(2,4-dioxo-3-azabicyclo[3.2.0]hept-6-ene-6-carboxylate) (DCBI) 7

Synthesis of ethyl 3,4-dipropylcyclobut-1-ene-1-carboxylate (CBO) 8

Determination of reactivity ratio 13

Ultrasonication of polymers followed by ^{1}H NMR and FT-IR measurements 13

Ultrasonication of polymers followed by treatment with NaOH solution 13

NaOH solution treatment of polymers followed by ultrasonication 14

Cryo-milling of polymers followed by treatment with NaOH solution 14

Ball-milling of polymers followed by treatment with NaOH solution 14

Ball-milling of dry polymers with solid NaOH 14

Ultrasonication of polymer followed by treatment with NaOH solutions of different concentrations 15

Degradation products analysis 15

Thermal stability experiments of PHCBI and PS-*co*-PHCBI 15

Supporting Data *16*

Determination of copolymer type by NMR experiments 16

Determination of monomer reactivity ratios 18

TGA and DSC data 21

Dynamic mechanical analysis (DMA) data 26

Thermal stability experiments of PHCBI and PS-*co*-PHCBI 30

Effect of ultrasonication on polymers monitored by ^{1}H NMR and FT-IR measurements 32

Treatment of polymers with NaOH solution followed by ultrasonication 47

Cryo-milling of polymers followed by treatment with NaOH solution **47**

Treatment of polymers with different mechanical methods and hydrolysis **50**

Ball-milling of dry polymers with solid NaOH **51**

Ultrasonication of polymer followed by treatment with NaOH solutions of different concentrations **52**

Chromatographic isolation and characterization of degradation products of PS_{50}-*co*-$PHCBI_{50}$ **52**

Characterization of the degradation products **54**

CoGEF Calculation **62**

NMR spectra **88**

Reference ***120***

Experimental Section

Materials

Styrene, methyl acrylate and methyl methacrylate were purchased from Sigma-Aldrich and passed through a short basic alumina column before use. 1,3-Butadiene (2 mol L^{-1} in THF) was purchased from TCI and passed through a short basic alumina column before use. Azobisisobutyronitrile (AIBN) was purchased from Sigma-Aldrich and recrystallized in methanol before use. Hexylamine, hexamethylenediamine, maleic anhydride, (*Z*)-oct-4-ene, aluminium chloride ($AlCl_3$), trifluoroacetic acid (TFA), acetic acid, acetic anhydride, nickel (II) acetate tetrahydrate, trimethylamine (Et_3N), cross-linked polystyrene (Poly(styrene-*co*-divinylbenzene)) and 1,3,5-trimethoxybenzene were purchased from Sigma-Aldrich and used without further purification. *N*-methylmaleimide was purchased from TCI and used without further purification. Ethyl propiolate was purchased from Fluorochem and used without further purification. Polystyrene (M.W. 50 kg mol^{-1}, $Đ$ = 1.06) was purchased from Alfa Aesar. Solvents were purchased from Acros Organics, Sigma Aldrich, Fischer Scientific, and Reactolab and were used without further purification. Deuterated chloroform ($CDCl_3$) was purchased from Cambridge Isotope Laboratories. *N*-hexyl maleimide is commercial available and here we synthesized it following the literature[1]. 1,1'-(hexane-1,6-diyl)bis(1H-pyrrole-2,5-dione) was synthesized following the literature[2].

Instrumentation

NMR spectra were recorded on a Bruker Avance III 300 MHz NMR spectrometer (^1H NMR 300 MHz, ^{13}C NMR 75 MHz) or Bruker Avance III 400 MHz NMR spectrometer (^1H NMR 400 MHz, ^{13}C NMR 101 MHz) or Bruker Avance III 400 MHz NMR spectrometer (^1H NMR 400 MHz, ^{13}C NMR 101 MHz) with Prodigy Cryoprobe. Data were evaluated with the MestReNova software suite and all chemical shifts δ are reported in parts per million (ppm) with coupling constant in Hz. IR spectra were measured on a PerkinElmer Spectrum 65 FT-IR spectrometer with an ATR set-up. The polymer solution was pipetted onto the crystal, and the solvent was allowed to evaporate before a measurement.

Relative molecular weights and molecular weight distributions of polymers were measured by size exclusion chromatography (SEC) on either A or B. (A) An Agilent 1200 series HPLC system equipped with an Agilent PLgel mixed guard column (particle size = 5 μm) and two Agilent PLgel mixed-D columns (ID = 7.5 mm, L = 300 mm, particle size = 5 μm). Signals were recorded by an UV detector (Agilent 1200 series, 346 nm), an Optilab REX interferometric refractometer, and a miniDawn TREOS light scattering detector (Wyatt Technology Corp.). Samples were run using THF as the eluent at 30 ℃ and a flow rate of 1.0 mL min^{-1}. The sample solutions were filtered through a 13 mm syringe filter (Nylon 66, nonsterile, pore size 0.22 μm, Swiss Labs) before injection. (B) An Agilent 1260 series pump equipped with two Agilent PLgel MIXED-B columns (ID = 7.5 mm, L = 300 mm, particle size = 10 μm), an Agilent 1200 series diode array detector (254 nm and 320 nm), a Wyatt 18-angle

DAWN HELEOS light scattering detector, and an Optilab rEX differential refractive index detector. The mobile phase was THF at room temperature and a flow rate of 1.0 mL min^{-1}. The sample solutions were filtered through a 13 mm syringe filter (PTFE, nonsterile, pore size 0.22 μm, Filtrous) before injection. Data analyses were carried out on Astra software (Wyatt Technology Corp.) and molecular weights were determined based on narrow molecular weight polystyrene standards calibration.

Thermogravimetric analysis (TGA) was conducted on a Mettler Toledo TGA/DSC 1 STAR system under nitrogen up to 600 ℃ with a heating rate of 10 ℃ min^{-1}. The reported degradation temperature (T_d) was taken at the extrapolated onset temperature.

Differential scanning calorimetry (DSC) was conducted on a Mettler Toledo DSC 2 STAR system under nitrogen up to 250 °C with a heating and cooling rate of 20 ℃ min^{-1}. The glass transition temperature (T_g) was taken from the mid-point of the thermal transition in the second heating cycle (T_g of PCBI was taken from the first heating cycle).

Dynamic mechanical analysis (DMA) measurements were performed using a TA Instruments DMA Q800 in tensile mode. The polymer films were cut into rectangular strips of a width of 2.8 mm and a length of ≈12 mm so that the measurement length of the sample was maintained at ≈10 mm. Experiments were conducted in a range of −70 ℃ to 200 ℃. The heating rate of 3 ℃ min^{-1} and a strain amplitude of 5 μm at a frequency of 1 Hz was applied.

Ultrasonication was performed with a Branson 450 digital sonifier using a 13 mm tip. Pulsed ultrasound (1 s on, 1 s off, 20 kHz, 20% amplitude) was applied at 0 ℃ with a chiller.

Cryo-milling was conducted on a SPEX SamplePrep 6770 Freezer/mill. The grinding was performed in a polycarbonate vial with 2 min-grinding-2 min-cooling setting at a rate of 12 cycles per second, under liquid nitrogen.

Ball-milling was conducted in a 10 ml screw-top grinding jar with stainless steel grinding balls with a diameter of 2 mm on a Retsch Mixer Mill MM400 with a frequency of 30 Hz.

Electrospray ionization mass spectrometry (ESI-MS) of synthesized compounds was carried out on a Bruker 4.7T BioAPEX II or an Agilent 6140 series Quadrupole LCMS Spectrometer system.

The high-resolution LC-MS analysis was performed by ultra-performance liquid chromatography coupled to a hybrid quadrupole, orthogonal acceleration time-of-flight high-resolution mass spectrometer (UPLC-QToF). A Waters ACQUITY™ UPLC™ I-Class coupled to a Waters Xevo™ G2-XS QTof (Waters Corporation, Wilmslow, UK) system was used in the Waters laboratory in Eschborn, Germany. The sample solutions were separated by reversed phase liquid chromatography using an ACQUITY UPLC I-Class system on a Waters ACQUITY UPLC BEH C18 analytical column (100 mm x 2.1 mm, 1.7 μm). In positive ionization mode, the injection volume was 2 μL (Solvent, PS_{49}-*co*-$PHCBI_{51}$, PS_{49}-*co*-$PHCBI_{51}$-cryo-milling-hydrolysis, PS_{49}-*co*-$PHCBI_{51}$-sonication-hydrolysis) and 8 μL (blank matrix). The higher injection volume of the blank matrix sample is to take into account the lower initial weight by a factor of four.

Due to the acidic chromatographic conditions and the therefore lower expected response in the negative ionization mode, the injection volume was 4 µL and 10 µL respectively. The UPLC parameters were as follows: column temperature 50 °C; flow rate 0.40 mL/min; temperature of the autosampler 8 °C; mobile phase, solvent A (0.1% formic acid in water, Biosolve, LCMS grade) and solvent B (0.1% formic acid in acetonitrile, Biosolve LCMS grade). The chromatographic separation started at 10% B for 0.5 min. The gradient was then increased to 98% B in 9 min and kept isocratic for 6 min. At 15.1 min the mobile phase ratio changed back to the initial conditions and was re-equilibrated for 2.4 min. The total run time per sample was 17 min. The LC-MS analysis was performed using the Xevo G2-XS QTof system with an electrospray (ESI) source operated in both positive (ESI^-) and negative (ESI^-) ion modes. The mass spectrometer operating conditions were as follows: cone voltage, 20 V; capillary voltage in ESI^+ mode 3.0 kV; capillary voltage in ESI^- mode -2.5 kV; source temperature 120 °C; desolvation temperature 550 °C; nitrogen cone gas flow rate 50 L/h; and nitrogen desolvation gas flow of 950 L/h. The high resolution time of flight mass analyzer was operated at 30,000 resolution (defined as FWHM at *m/z* 956) with an acquisition time of 0.15s. The mass spectrometer was calibrated using a solution of sodium formate over a mass range of 50–2000 Da. The MS^E mode was used for data collection, meaning alternating scans between a low-energy full scan and a high-energy full scan. The collision voltage was set as 6.0 eV for the low-energy scan and a 15-55 eV ramp for the high-energy scan. Data were acquired over a mass range of 50–2000 *m/z*. The acquired data were centroided and lock mass corrected during data acquisition using an external reference (Lock-Spray™) consisting of a 200 pg/mL solution of leucine enkephalin infused at a flow rate of 20 µL/min via a lockspray interface, generating a realtime reference ion of $[M+H]^+$ (*m/z* 556.2766) in positive ion mode and $[M-H]^-$ (*m/z* 554.2620) in negative ion mode to ensure accurate MS analyses. Data acquisition and data processing was performed using the waters_connect informatics platform and UNIFI™ (Waters Corporation, Wilmslow, UK). Peaks detection was performed by an Apex peak detection algorithm using the 3 dimensional peak shape[3]. An in-house, self-built compounds library (containing compound name, molecular formula, proposed chemical structure, and accurate molecular mass) of 18 selected target compounds was developeded (Fig. S96) and used in waters_connect software. An accurate mass window of ± 5 ppm was used as criteria for compounds confirmation on molecular ion masses. Typical adduct ions were selected including H^+, Na^+, $HCOO^-$ and H^-, and used for molecular ion confirmation purposes.The LC-MS system performance was assessed for mass accuracy, repeatability and sensitivity using a system suitability standard solution consisting of a 9 component mixture (acetaminophen, caffeine, leucine enkephalin, reserpine, sulfaguanidine, sulfadimethoxine, terfenadine, Val-Tyr-Val, verapamil) for both ionisation modes before measuring the actual samples of interest. Each sample was analysed in five replicate measurements.

UV reactions were conducted under a mercury lamp (36 W or 400 W) with cooling fans.
Silica flash column chromatography was performed on a Biotage Isolera system using SiliCycle SiliaSep HP flash cartridges.

Synthesis of ethyl 3-hexyl-2,4-dioxo-3-azabicyclo[3.2.0]hept-6-ene-6-carboxylate (HCBI)

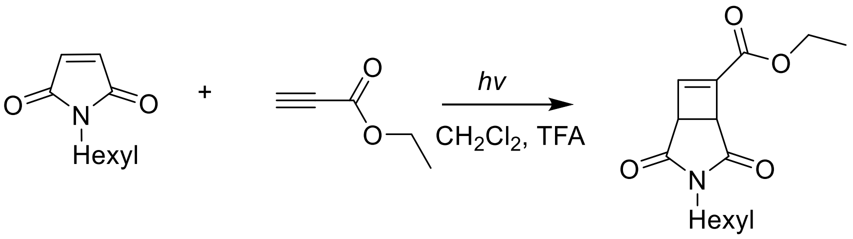

HCBI was synthesized following the literature[4] with some modifications. *N*-hexyl maleimide (1.81g, 10 mmol, 1.0 eq), ethyl propiolate (2.45 g, 25 mmol, 2.5 eq.) and trifluoroacetic acid (1.2 g, 11 mmol, 1.1 eq.) were dissolved in 60 mL methylene chloride in a 100 mL flask. After sparging the solution for 30 min with N_2, the flask was sealed by septum and stirred under UV irradiation (mercury lamp, 400 W) for 48 h. After completion of the reaction (thin layer chromatography (TLC) monitoring), the solvent was removed under vacuum. The crude residue was purified by silica column chromatography (5-50% ethyl acetate/hexane) to afford the product as a light yellow liquid.
Yield: 62%. ^{1}H NMR (400 MHz, Chloroform-d) δ 7.03 (d, *J* = 1.3 Hz, 1H), 4.26 (q, *J* = 7.1 Hz, 2H), 4.01 (d, *J* = 3.2 Hz, 1H), 3.75 (dd, *J* = 3.2, 1.3 Hz, 1H), 3.40 – 3.48 (m, 2H), 1.45 – 1.57 (m, 2H), 1.32 (t, *J* = 7.1 Hz, 3H), 1.18 – 1.28 (m, 6H), 0.80 – 0.95 (m, 3H).^{13}C NMR (101 MHz, Chloroform-*d*) δ 172.77, 172.26, 160.19, 145.29, 141.29, 61.35, 46.73, 44.29, 38.94, 31.25, 27.38, 26.36, 22.45, 14.13, 13.93. ESI-MS: 302.0 (M + Na^+).

Synthesis of ethyl 3-methyl-2,4-dioxo-3-azabicyclo[3.2.0]hept-6-ene-6-carboxylate (MCBI)

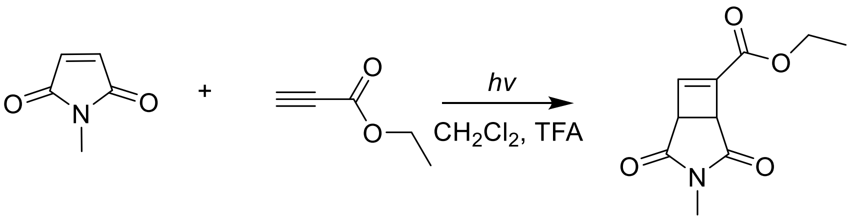

MCBI was synthesized following the literature[4] with some modifications. *N*-methylmaleimide (3.0 g, 27 mmol, 1.0 eq), ethyl propiolate (6.6 g, 67 mmol, 2.6 eq.) and trifluoroacetic acid (3.2 g, 28 mmol, 1.1 eq.) were dissolved in 80 mL methylene chloride in a 100 mL flask. After sparging the solution for 30 min with N_2, the flask

was sealed by septum and stirred under UV irradiation (mercury lamp, 400 W) for 48 h. After completion of the reaction (thin layer chromatography (TLC) monitoring), the solvent was removed under vacuum. The crude residue was purified by silica column chromatography (5-50% ethyl acetate/hexane) to afford the product as a light yellow liquid.

Yield: 77%. ^{1}H NMR (400 MHz, Chloroform-*d*) δ 6.94 (d, *J* = 1.3 Hz, 1H), 4.14 (q, *J* = 7.1 Hz, 2H), 3.97 (d, *J* = 3.2 Hz, 1H), 3.72 (dd, *J* = 3.2, 1.3 Hz, 1H), 2.85 (s, 1H), 1.21 (t, *J* = 7.1 Hz, 3H).^{13}C NMR (101 MHz, Chloroform-*d*) δ 172.81, 172.38, 160.13, 145.21, 140.94, 61.22, 46.80, 44.36, 24.93, 14.05. ESI-MS: 210.1 (M + H^{+}).

Synthesis of diethyl 3,3'-(hexane-1,6-diyl)bis(2,4-dioxo-3-azabicyclo[3.2.0]hept-6-ene-6-carboxylate) (DCBI)

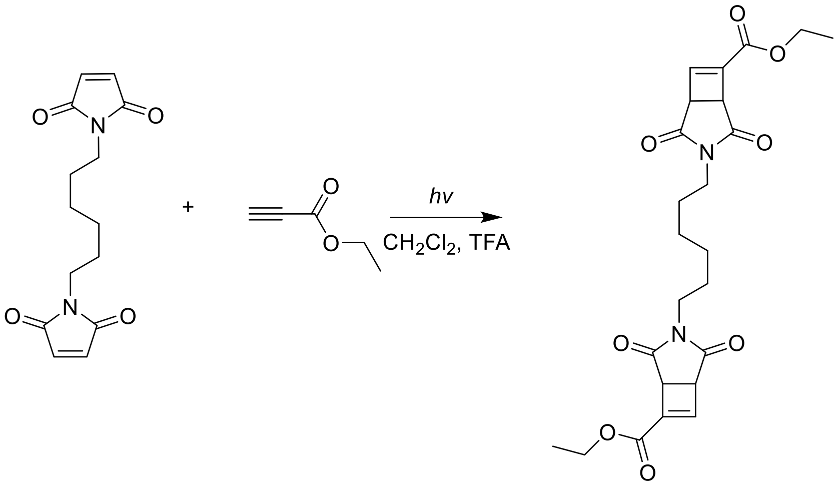

DCBI was synthesized following the literature[4] with some modifications. 1,1'-(hexane-1,6-diyl)bis(1H-pyrrole-2,5-dione) (3.0 g, 27 mmol, 1.0 eq), ethyl propiolate (6.6 g, 67 mmol, 2.6 eq.) and trifluoroacetic acid (3.2 g, 28 mmol, 1.1 eq.) were dissolved in 80 ml methylene chloride in a 100 mL flask. After sparging the solution for 30 min with N_2, the flask was sealed by septum and stirred under UV irradiation (mercury lamp, 36 W) for 48 h. After completion of the reaction (thin layer chromatography (TLC) monitoring), the solvent was removed under vacuum. The crude residue was purified by silica column chromatography (5-50% ethyl acetate/hexane) to afford the product as a light yellow liquid.

Yield: 19%. ^{1}H NMR (400 MHz, Chloroform-d) δ 7.01 (d, *J* = 1.3 Hz, 2H), 4.23 (q, *J* = 7.1 Hz, 4H), 4.01 (dd, *J* = 3.3, 1.7 Hz, 2H), 3.75 (d, *J* = 3.4 Hz, 2H), 3.40 (t, *J* = 7.3 Hz, 4H), 1.45 – 1.51 (m, 4H), 1.22 – 1.32 (m, 10H). ^{13}C NMR (101 MHz, Chloroform-*d*) δ 170.88, 170.41, 158.29, 143.41, 139.28, 59.44, 44.82, 42.39, 36.73, 25.29, 24.16, 24.15, 12.22. ESI-MS: 473.2 (M + H^{+}).

Synthesis of ethyl 3,4-dipropylcyclobut-1-ene-1-carboxylate (CBO)

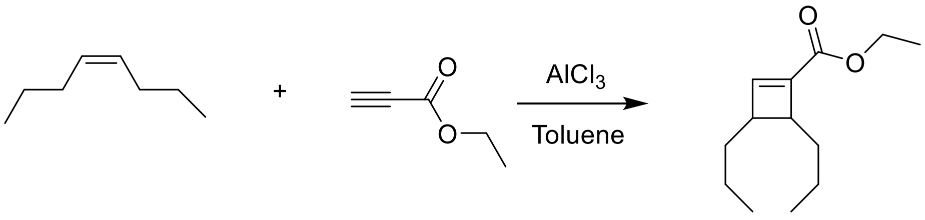

CBO was synthesized following the literature[5] with some modifications. Anhydrous aluminum chloride (1.79 g, 13.4 mmol, 0.5 eq.) was charged to a 100 mL flame-dried round bottom flask under N_2, followed by 20 mL anhydrous toluene. Ethyl propiolate (3.1 g, 32.1 mmol, 1.2 eq.) was then added dropwise, followed by (*Z*)-oct-4-ene (3 g, 26.7 mmol, 1.0 eq.). The reaction was stirred for 7 days at room temperature, then poured into an aqueous solution of KH_2PO_4 (0.2 M, 60 ml) with stirring, resulting in the formation of a white precipitate. 20 mL of a 10% HCl solution was added to dissolve the precipitate. The aqueous layer was extracted with 3 x 20 mL portions of diethyl ether. The combined organic layers were washed with brine and dried over $MgSO_4$. Solvent was removed under reduced pressure. The crude residue was purified by silica column chromatography (0-20% ethyl acetate/hexane) to afford the product as a colorless liquid.

Yield: 55 %. ^{1}H NMR (400 MHz, Chloroform-*d*) δ 6.88 (d, *J* = 1.3 Hz, 1H), 4.12 – 4.22 (m, 2H), 3.04 (td, J = 6.9, 4.4 Hz, 1H), 2.73 – 2.78 (m, 1H), 1.25 – 1.59 (m, 11H), 0.87 – 0.95 (m, 6H). ^{13}C NMR (101 MHz, Chloroform-*d*) δ162.92, 149.39, 142.04, 59.89, 45.32, 43.65, 30.95, 30.76, 21.63, 21.18, 14.37, 14.28, 14.23. ESI-MS: 211.2 (M + H^+).

Polymerizations

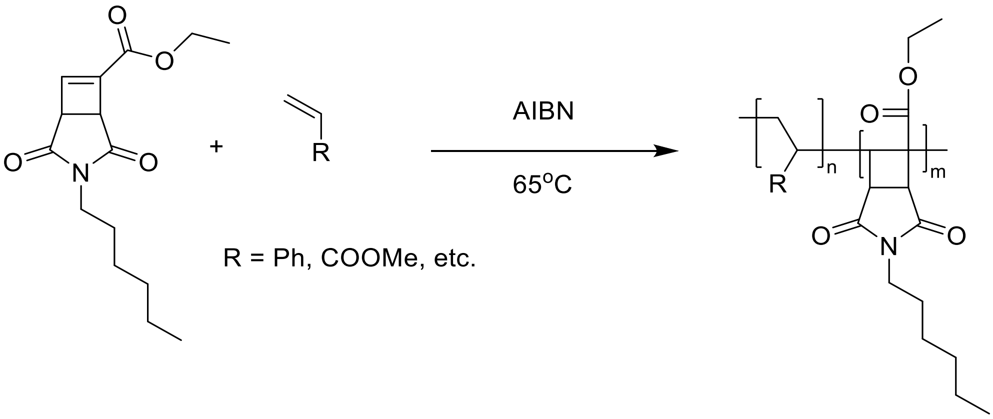

PHCBI. HCBI (8 g, 29 mmol), AIBN (3 mg, 0.018 mmol), and 10 mL 1,4-dioxane were added into a Schlenk flask. The mixture was sparged with Ar for 60 min, before heating to 65 ℃. After stirring for 72 h at 65 ℃, the reaction solution was cooled to room temperature and precipitated into cold methanol (200 mL x 5). The precipitate was filtered off and dried under vacuum, and the title polymer was isolated as a white powder.

^1H NMR (400 MHz, Chloroform-*d*) δ 3.75-4.54 (m), 1.89-3.68 (m), 1.08-1.62 (m), 0.76-0.95 (m). ^{13}C NMR (101 MHz, Chloroform-*d*) δ 177.02, 176.14, 62.94, 62.23, 39.16, 31.36, 29.70, 27.28, 26.57, 22.54, 13.97, 13.33.

PS-*co*-PHCBI (F_{CBI} = 18%, 25%, 40%, 45%). Styrene (6.25 g, 60 mmol), HCBI (0.84 g, 4.32 mmol or 1.68 g, 8.65 mmol or 3.36 g, 17.30 mmol or 5.04 g, 25.95 mmol), and AIBN (3 mg, 0.018 mmol) were added into a Schlenk flask. The mixture was degassed by three freeze-pump-thaw degassing cycles, before taking 0.1 mL solution for ^1H NMR measurement. After stirring for 60 h at 65 ℃ under Ar, the reaction solution was cooled to room temperature and precipitated into cold methanol (200 mL x 5). The precipitate was filtered off and dried under vacuum, and the title polymer was isolated as a white solid.

PS_{82}-*co*-$PHCBI_{18}$: ^1H NMR (400 MHz, Chloroform-*d*) δ 6.26-7.29 (m), 3.03-3.95 (m), 1.09-2.60 (m), 0.68-1.07 (m). ^{13}C NMR (101 MHz, Chloroform-*d*) δ 175.74, 172.08, 146.01, 145.21, 144.92, 127.96, 127.32, 125.65, 125.50, 60.92, 45.68, 43.85, 40.79, 40.54, 40.44, 31.35, 29.72, 27.69, 26.61, 22.52, 22.50, 14.05.

PS_{75}-*co*-$PHCBI_{25}$: ^1H NMR (400 MHz, Chloroform-*d*) δ 6.25-7.23 (m), 3.08-3.86 (m), 1.09-2.68 (m), 0.63-1.09 (m). ^{13}C NMR (101 MHz, Chloroform-*d*) δ 175.64, 172.12, 145.38, 145.17, 128.56, 127.95, 127.84, 127.42, 125.93, 125.61, 60.93, 40.62, 40.30, 40.06, 31.33, 27.70, 27.68, 26.56, 26.50, 22.54, 22.52, 14.04.

PS_{60}-*co*-$PHCBI_{40}$:^1H NMR (400 MHz, Chloroform-*d*) δ 6.28-7.23 (m), 3.01-3.79 (m), 1.10-2.65 (m), 0.60-1.10 (m). ^{13}C NMR (101 MHz, Chloroform-*d*) δ 176.16, 174.45, 144.97, 143.45, 128.31, 127.82, 127.61, 125.64, 61.40, 60.65, 42.26, 40.67, 40.39, 38.77, 31.34, 27.68, 27.28, 26.54, 22.50, 14.03.

PS_{55}-*co*-$PHCBI_{45}$:^1H NMR (400 MHz, Chloroform-*d*) δ 6.31-7.25 (m), 3.00-3.85 (m), 1.10-2.98 (m), 0.59-1.08 (m). ^{13}C NMR (101 MHz, Chloroform-*d*) δ 175.94, 172.34, 144.12, 128.57, 128.27, 127.49, 125.73, 60.71, 42.87, 40.00, 38.77, 31.35, 27.72, 27.48, 26.54, 22.50, 14.02.

PS-*co*-PHCBI (F_{HCBI} = 50%, 51%, 55%, 62%). Styrene and HCBI (For F_{HCBI} = 50%, 55 mmol in total, styrene: HCBI = 20:80, for F_{CBI} = 51%, 55%, 62%, 60 mmol in total, styrene: HCBI =20:80, 40:60, 70:30), as well as AIBN (3 mg, 0.018 mmol) were added into a Schlenk flask. The mixture was degassed by three freeze-pump-thaw degassing cycles, before taking 0.1 mL solution for ^1H NMR measurement. After stirring 24 h at 65 ℃ under Ar, the reaction solution was cooled to room temperature and precipitated into cold methanol (200 mL x 5). The precipitate was filtered off and dried under vacuum, and the title polymer was isolated as a white solid.

PS$_{50}$-*co*-PHCBI$_{50}$: Yield: 37%. ^{1}H NMR (400 MHz, Chloroform-*d*) δ 6.38-7.67 (m), 1.89-4.28 (m), 0.34-1.67 (m). ^{13}C NMR (101 MHz, Chloroform-*d*) δ 128.58, 61.78, 31.36, 28.00, 26.58, 22.53, 14.04.

PS$_{49}$-*co*-PHCBI$_{51}$:^{1}H NMR (400 MHz, Chloroform-*d*) δ 6.28-7.40 (m), 1.10-4.20 (m), 0.55-1.09 (m). ^{13}C NMR (101 MHz, Chloroform-*d*) δ 176.59, 175.80, 172.10, 128.11, 126.90, 61.01, 42.25, 39.03, 31.48, 27.70, 27.39, 26.58, 26.46, 22.49, 14.02.

PS$_{45}$-*co*-PHCBI$_{55}$:^{1}H NMR (400 MHz, Chloroform-*d*) δ 6.28-7.52 (m), 1.10-4.30 (m), 0.55-1.09 (m). ^{13}C NMR (101 MHz, Chloroform-*d*) δ 176.92, 128.86, 127.70, 126.79, 60.84, 43.03, 40.91, 38.66, 31.35, 27.52, 26.65, 22.50, 14.01.

PS$_{38}$-*co*-PHCBI$_{62}$:^{1}H NMR (400 MHz, Chloroform-*d*) δ 6.69-7.80 (m), 1.83-4.32 (m), 1.10-1.82, 0.55-1.09 (m). ^{13}C NMR (101 MHz, Chloroform-*d*) δ 177.04, 175.59, 172.70, 128.42, 127.10, 60.92, 42.20, 40.66, 38.88, 31.37, 31.32, 27.67, 27.47, 26.61, 22.52, 22.50, 14.02.

PS$_{73}$-*co*-PHCBI$_{27}$. Styrene (5.2 g, 50 mmol), HCBI (2.5 g, 9 mmol), 4-cyano-4-(phenylcarbonothioylthio)pentanoic acid (16 mg, 0.06 mmol), and AIBN (3 mg, 0.018 mmol) were added into a Schlenk flask. The mixture was degassed by three freeze-pump-thaw degassing cycles, before taking 0.1 mL solution for ^{1}H NMR measurement. After stirring 168 h at 65 ℃ under Ar, the reaction solution was cooled to room temperature and precipitated into cold methanol (200 mL x 5). The precipitate was filtered off and dried under vacuum, and the title polymer was isolated as a pink solid.
^{1}H NMR (400 MHz, Chloroform-*d*) δ 6.30-7.20 (m), 3.02-3.84 (m), 1.08-2.60 (m), 0.7-1.06 (m). ^{13}C NMR (101 MHz, Chloroform-*d*) δ 176.47, 175.47, 146.22, 145.08, 128.26, 127.95, 127.82, 127.78, 125.49, 47.34, 40.52, 39.76, 38.55, 31.40, 31.33, 27.49, 26.63, 26.55, 22.48, 14.03, 13.61.

PMA$_{87}$-*co*-PHCBI$_{13}$. Methyl acrylate (4.13 g, 48 mmol), HCBI (3.35 g, 12 mmol), and AIBN (3 mg, 0.018 mmol) were added into a Schlenk flask. The mixture was degassed by three freeze-pump-thaw degassing cycles, before heating to 65 ℃. After stirring 15 h at 65 ℃ under Ar, the reaction solution was cooled to room temperature and precipitated into cold methanol (200 mL x 5). The precipitate was filtered off and dried under vacuum, and the title polymer was isolated as a colorless gel.
^{1}H NMR (400 MHz, Chloroform-*d*) δ 3.98-4.35 (m), 3.55-3.80 (m), 3.41-3.50 (m), 2.86-3.28 (m), 2.13-2.62 (m), 1.07-2.03 (m), 0.76-0.90 (m). ^{13}C NMR (101 MHz, Chloroform-*d*) δ 174.89, 61.84, 53.42, 51.71, 50.81, 41.53, 41.31, 41.12, 35.16, 31.30, 31.27, 27.59, 26.52, 22.49, 13.98, 13.59.

PMA$_{97}$-*co*-PHCBI$_{3}$. Methyl acrylate (5.2 g, 60 mmol), HCBI (0.84 g, 3 mmol), AIBN (3 mg, 0.018 mmol), and 6 mL 1,4-dioxane were added into a Schlenk flask. The

mixture was degassed by three freeze-pump-thaw degassing cycles, before heating to 65 ℃. After stirring 15 h at 65 ℃, the reaction solution was cooled to room temperature and precipitated into cold methanol (200 mL x 5). The precipitate was filtered off and dried under vacuum, and the title polymer was isolated as a colorless gel.

^{1}H NMR (400 MHz, Chloroform-*d*) δ 3.98-4.37 (m), 3.55-3.82 (m), 2.86-3.51 (m), 1.04-2.44 (m), 0.77-0.92 (m). ^{13}C NMR (101 MHz, Chloroform-*d*) δ 174.89, 51.84, 51.81, 51.71, 50.83, 41.39, 41.31, 41.13, 35.84, 35.82, 35.56, 35.16, 34.96, 34.95, 34.74, 34.46, 34.26, 31.27, 27.60, 26.50, 22.50, 13.98.

$PMMA_{98}$-*co*-$PHCBI_{2}$. Methyl methacrylate (6.01 g, 60 mmol), HCBI (0.84 g, 3 mmol), AIBN (3 mg, 0.018 mmol), and 6 mL 1,4-dioxane were added into a Schlenk flask. The mixture was degassed by three freeze-pump-thaw degassing cycles, before heating to 65 ℃. After stirring 15 h at 65 ℃, the reaction solution was cooled to room temperature and precipitated into cold methanol (200 mL x 5). The precipitate was filtered off and dried under vacuum, and the title polymer was isolated as a white solid.

^{1}H NMR (400 MHz, Chloroform-*d*) δ 4.02-4.27 (m), 3.36-3.96 (m), 3.01-2.21 (m), 2.35-2.85 (m), 1.52-2.12 (m), 1.15-1.46 (m), 0.67-1.12 (m). ^{13}C NMR (101 MHz, Chloroform-*d*) δ 178.08, 177.78, 176.97, 176.93, 54.49, 54.18, 52.70, 44.92, 44.57, 18.76, 16.51, 16.48.

PS_{34}-*co*-PB_{49}-*co*-$PHCBI_{17}$. Styrene (3.12 g, 30 mmol), 1,3-butadiene (15 mL of a 2 mol L^{-1} solution in THF, 30 mmol), HCBI (0.84 g, 3 mmol), and AIBN (3 mg, 0.018 mmol) were added into a Schlenk flask. The mixture was degassed by three freeze-pump-thaw degassing cycles, before transferring into a 100 mL ACE pressure tube under Ar. After stirring 72 h at 65 ℃ in the pressure flask, the reaction solution was cooled to room temperature and precipitated into cold methanol (200 mL x 5). The precipitate was filtered off and dried under vacuum, and the title polymer was isolated as a colorless gel.

^{1}H NMR (400 MHz, Chloroform-*d*) δ 6.71-7.56 (m), 4.77-5.62 (m), 3.03-4.24 (m), 1.38-3.01 (m), 0.67-1.37 (m). ^{13}C NMR (101 MHz, Chloroform-*d*) δ 178.33, 176.47, 172.21, 145.36, 145.00, 131.43, 130.01, 129.98, 128.14, 127.79, 125.85, 125.81, 61.31, 53.42, 45.73, 43.46, 40.07, 38.89, 32.68, 31.32, 30.46, 27.71, 27.37, 26.59, 22.51, 14.01.

PS. Commercially available polystyrene was used. ^{1}H NMR (400 MHz, Chloroform-*d*) δ 6.90-7.20 (m), 6.30-6.58 (m), 1.26-1.57 (m), 1.84-2.06 (m). ^{13}C NMR (101 MHz, Chloroform-*d*) δ 145.22, 128.03, 127.95, 127.59, 127.42, 125.63, 125.48, 40.71m 40.41, 40.26.

PMA. Methyl acrylate (12 g, 140 mmol), AIBN (3 mg, 0.018 mmol), 4-cyano-4-(phenylcarbonothioylthio)pentanoic acid (4.8 mg, 0.017 mmol), and 10 mL 1,4-dioxane were added into a Schlenk flask. The mixture was degassed by three freeze-pump-thaw degassing cycles, before heating to 65 ℃. After stirring 15 h at 65 ℃, the reaction

solution was cooled to room temperature and precipitated into cold methanol (200 mL x 5). The precipitate was filtered off and dried under vacuum, and the title polymer was isolated as a colorless gel.
^{1}H NMR (400 MHz, Chloroform-*d*) δ 3.46-3.82 (m), 2.23-2.38 (m), 1.87-1.96 (m), 1.60-1.77 (m), 1.38-1.56 (m). ^{13}C NMR (101 MHz, Chloroform-*d*) δ 174.87, 67.08, 51.79, 51.70, 41.30, 41.12, 35.81, 35.16, 34.26.

PMMA. Methyl methacrylate (11 g, 110 mmol), AIBN (3 mg, 0.018 mmol), 4-cyano-4-(phenylcarbonothioylthio)pentanoic acid (4.8 mg, 0.017 mmol), and 10 mL 1,4-dioxane were added into a Schlenk flask. The mixture was degassed by three freeze-pump-thaw degassing cycles, before heating to 65 ℃. After stirring 15 h at 65 ℃, the reaction solution was cooled to room temperature and precipitated into cold methanol (200 mL x 5). The precipitate was filtered off and dried under vacuum, and the title polymer was isolated as a white solid.
^{1}H NMR (400 MHz, Chloroform-*d*) δ 3.40-3.76 (m), 1.62-2.06 (m), 1.35-1.48 (m), 1.15-1.22 (m), 0.74-1.08 (m). ^{13}C NMR (101 MHz, Chloroform-*d*) δ 178.11, 178.08, 177.78, 176.97, 176.90, 67.08, 54.43, 54.21, 52.72, 51.81, 44.91, 44.56, 18.74, 16.50.

PS_{47}-*co*-PB_{53}. Styrene (4.5 g, 43 mmol), 1,3-butadiene (15 mL of a 2 mol L^{-1} solution in THF, 30 mmol), 4-cyano-4-(phenylcarbonothioylthio)pentanoic acid (2.5 mg, 0.009 mmol), and AIBN (3 mg, 0.018 mmol) were added into a Schlenk flask. The mixture was degassed by three freeze-pump-thaw degassing cycles, before transferring into a 100 mL ACE pressure tube under Ar. After stirring 72 h at 65 ℃ in the pressure tube, the reaction solution was cooled to room temperature and precipitated into cold methanol (200 mL x 5). The precipitate was filtered off and dried under vacuum, and the title polymer was isolated as a pink gel.
^{1}H NMR (400 MHz, Chloroform-*d*) δ 6.46-7.31 (m), 4.74-5.51 (m), 1.00-2.52 (m). ^{13}C NMR (101 MHz, Chloroform-*d*) δ 145.31, 136.88, 131.55, 130.20, 128.51, 127.63, 126.20, 125.84, 113.79, 53.42, 50.85, 45.30, 43.39, 39.98, 35.82, 32.66, 30.32, 27.28, 25.26.

PS_{65}-*co*-$PCBO_{35}$. Styrene (1.24 g, 11.9 mmol) and CBO (1 g, 4.75mmol), as well as AIBN (1 mg, 6 μmol) were added into a Schlenk flask. The mixture was degassed by three freeze-pump-thaw degassing cycles, before taking 0.1 mL solution for ^{1}H NMR measurement. After stirring 48 h at 65 ℃ under N_2, the reaction solution was cooled to room temperature and precipitated into cold methanol (100 mL x 5). The precipitate was filtered off and dried under vacuum, and the title polymer was isolated as a white solid.
1.02 g, 45%. ^{1}H NMR (400 MHz, Chloroform-*d*) δ 6.26-7.36 (m), 2.31-4.23 (m), 0.11-2.26 (m). ^{13}C NMR (101 MHz, Chloroform-*d*) δ 145.71, 127.93, 125.60, 59.28, 40.41, 14.49, 13.78. M_n: 26 kg mol^{-1}, *Đ*: 2.2.

(PS-*co*-PMCBI)-*l*-PDCBI Styrene (2.29 g, 21.98 mmol), MCBI (0.84 g, 4.01 mmol), DCBI (0.57 g, 1.2 mmol), and AIBN (1 mg, 6 μmol) were added into a Schlenk flask. The mixture was degassed by three freeze-pump-thaw degassing cycles, before taking 0.1 mL solution for ^{1}H NMR measurement. After stirring overnight at 65 ℃ under N_2, the reaction mixture was cooled to room temperature. The insoluble solid was washed by THF and dried under vacuum to give the title polymer.

Determination of reactivity ratio

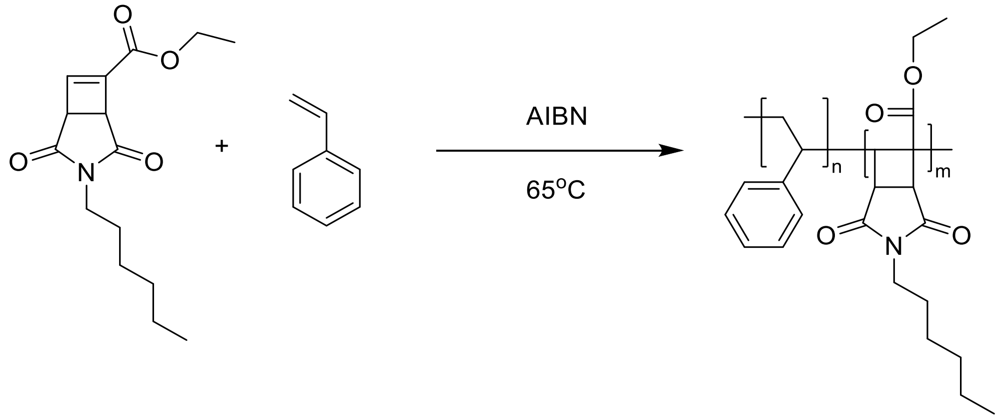

Styrene, HCBI (styrene and HCBI, overall amount 50 mmol), and AIBN (3 mg, 0.018 mmol) were added into a Schlenk flask. The mixture was degassed by three freeze-pump-thaw degassing cycles, before heating to 65 ℃. After 2 h (or 1.5 h), the reaction solution was cooled to room temperature, 0.1 mL was taken for ^{1}H NMR measurements. and the rest was precipitated into cold methanol (100 mL x 5). The precipitate was filtered off and dried under vacuum, and the polymer was isolated as a white solid.

Ultrasonication of polymers followed by ^{1}H NMR and FT-IR measurements

A solution of PS_{50}-*co*-$PHCBI_{50}$ (80 mg polymer in 20 mL THF) was placed in a Suslick reaction vessel and sparged with N_2 for 15 min prior to sonication. Pulsed ultrasound was applied (1 s on, 1 s off, 20 % amplitude) while the was solution kept in an ice/water bath. At 1 h, 2 h, 3 h and 4 h sonication time (on time), an aliquot (2.0 mL) was taken from the solution. After removing the solvent under reduced pressure, the samples were subjected to ^{1}H NMR analysis. Furthermore, for PS_{49}-*co*-$PHCBI_{51}$, 0.5 mL of withdrawn solution (4 h sonication) was subjected to FT-IR analysis.

Ultrasonication of polymers followed by treatment with NaOH solution

For a typical pulsed ultrasonication experiment, a solution of polymer (80 mg polymer in 20 mL THF) was placed in a Suslick reaction vessel and sparged with N_2 for 15 min prior to sonication. Pulsed ultrasound was applied (1 s on, 1 s off, 20 % amplitude) while the was solution kept in an ice/water bath. At each sonication time (0, 30, 60, 120, 180 and 240 min, on time), an aliquot (1.5 mL) was taken from the solution and directly subjected to SEC analysis. After measurement, 150 μL NaOH solution (1 mol L^{-1} or 0.5 mol L^{-1} for PMA) was added into the aliquot. After stirring overnight (0.5 h for PMA), the NaOH treated solution was further analyzed by SEC.

NaOH solution treatment of polymers followed by ultrasonication

A solution of PS_{49}-*co*-$PHCBI_{51}$ (120 mg polymer in 30 mL THF) was placed in a 50 mL beaker. Then, 3 mL NaOH solution (1 mol L^{-1}) was added and the solution was kept stirring overnight at room temperature. In the next day, after stopping stirring, the solution separated into two phases, the aqueous phase was removed with a separatory funnel. An aliquot (1.5 mL) was taken from the THF phase and directly subjected to SEC analysis. The rest of the THF phase was subjected to pulsed ultrasound (1 s on, 1 s off, 20 % amplitude) while the solution was kept at 0 ℃ with a chiller. At each sonication time point (0, 30, 60, 120, 180, and 240 min, on time), an aliquot (1.5 mL) was taken from the solution and directly subjected to SEC analysis.

Cryo-milling of polymers followed by treatment with NaOH solution

200 mg PS, PS_{49}-*co*-$PHCBI_{51}$, PS_{60}-*co*-$PHCBI_{40}$, PS_{50}-*co*-$PHCBI_{50}$, PS_{65}-*co*-$PCBO_{35}$, (PS-*co*-PMCBI)-*l*-PDCBI or cross-linked PS was placed in a cryo-milling machine. After cryo-milling (30 and 60 min for PS and PS_{49}-*co*-$PHCBI_{51}$, 60 min for PS_{60}-*co*-$PHCBI_{40}$, PS_{50}-*co*-$PHCBI_{50}$, PS_{65}-*co*-$PCBO_{35}$, (PS-*co*-PMCBI)-*l*-PDCBI, and cross-linked PS on time), a sample (2 mg) was taken and dissolved in 1.5 mL THF, filtered through a syringe filter (0.22 μm) and then directly subjected to SEC analysis. Another 2 mg sample was taken and dissolved in 1.5 mL THF. Then, 150 μL NaOH solution (1 mol L^{-1}) was added into the polymer solution. After stirring overnight at room temperature, the NaOH treated solution was further analyzed by SEC.

Ball-milling of polymers followed by treatment with NaOH solution

350 mg PS_{60}-*co*-$PHCBI_{40}$ (or PS) was placed in a 10 mL top screwed stainless steel grinding jar with stainless steel grinding balls with a diameter of 2 mm. The polymer was grinded for 60 min with 30 Hz frequency. Thereafter, a sample (2 mg) was taken and dissolved in 1.5 mL THF, filtered through a syringe filter (0.22 μm) and then directly subjected to SEC analysis. Another 2 mg sample was taken and dissolved in 1.5 mL THF. Then, 150 μL NaOH solution (1 mol L^{-1}) was added into the polymer solution. After stirring overnight at room temperature, the NaOH treated solution was further analyzed by SEC.

Ball-milling of dry polymers with solid NaOH

300 mg PS_{50}-*co*-$PHCBI_{50}$ (or PS) and 300 mg of solid NaOH were placed in a 10 mL top screwed stainless steel grinding jar with stainless steel grinding balls with a diameter of 2 mm. The polymer was grinded for 60 min with 30 Hz frequency. Thereafter, a sample (2 mg) was taken and dissolved in 1.5 mL THF, filtered through a syringe filter (0.22 μm) and then directly subjected to SEC analysis.

Ultrasonication of polymer followed by treatment with NaOH solutions of different concentrations

For a typical pulsed ultrasonication experiment, a solution of PS_{49}-*co*-$PHCBI_{51}$ (120 mg polymer in 30 mL THF) was placed in a 50 mL beaker. Pulsed ultrasound was applied (1 s on, 1 s off, 20 % amplitude) while the solution was kept at 0 ℃. After 240 min sonication, six aliquots (1.5 mL each) were taken from the solution and 150 μL NaOH solution of different concentrations (1.0 M, 0.5 M, 0.1 M, 0.05 M, 0.01 M, 0.001 M) was added into each of the aliquots. After stirring overnight at room temperature, the NaOH treated solutions were analyzed by SEC.

Degradation products analysis

For ultrasonication products: After sonication (240 min) and NaOH treatment, 0.1 mL of the PS_{50}-*co*-$PHCBI_{50}$ mixture was taken for mass analysis, the remainder of the mixture was purified by flash silica column chromatography (0-100% MeOH/DCM) to afford two fractions, F_1 (26 mg, 34%, M_n: 10 kg mol^{-1}) and F_2 (49 mg, 58%, M_n < 1 kg mol^{-1}). The fractions were further analyzed by ^{1}H NMR, SEC and Mass Spectrometry.

For cryo-milling products: After cryo-milling (60 min) and NaOH treatment, 0.1 mL of the PS_{50}-*co*-$PHCBI_{50}$ mixture was taken for mass analysis and the remainder of the mixture was purified by silica flash column chromatography (0-100% MeOH/DCM) to afford two fractions, F_1 (63 mg, 69%, M_n < 1 kg mol^{-1}) and F_2 (28 mg, 26%, M_n < 1 kg mol^{-1}). The fractions were further analyzed by ^{1}H NMR, SEC and Mass Spectrometry.

High-Resolution LC-MS Analysis*:* Several samples were provided for LC-MS analysis: the non-degraded PS_{49}-*co*-$PHCBI_{51}$, and the samples PS_{49}-*co*-$PHCBI_{51}$-cryo-milling-hydrolysis, PS_{49}-*co*-$PHCBI_{51}$-sonication-hydrolysis after degradation. Furthermore, a procedural blank sample (blank matrix) that did not contain the polymeric material used to assess possible artifacts/impurities generated from the sample preparation procedure (solvents, vessels and chemicals), and a solvent sample that took into account the solvents used to dissolve and dilute the polymeric samples. Material was taken from the sample vessels and weighed into Eppendorf vials. The weights were 0.80 mg for the non-degraded PS_{49}-*co*-$PHCBI_{51}$, 0.80 mg for the PS_{49}-*co*-$PHCBI_{51}$-cryomilling-hydrolysis, 0.70 mg for the PS_{49}-*co*-$PHCBI_{51}$-sonication-hydrolysis and 0.20 mg for the blank matrix sample. The samples were dissolved in 1.0 mL tetrahydrofuran/water 70/30 (v/v) prior to diluting 1:10 with acetonitrile/water 90/10 (v/v) for LC-MS analysis.

Thermal stability experiments of PHCBI and PS-*co*-PHCBI

20 mg of PHCBI or PS_{49}-*co*-$PHCBI_{51}$ was added into a dry 10 mL round bottom flask. The flask was vacuum degassed and refilled with Ar three times and then, while being under Ar, dipped into a heated oil bath (180 ℃). After heating for 30 min, the flask was cooled down to room temperature. Then, samples were characterized by ^{1}H NMR spectroscopy and SEC.

Supporting Data

Determination of copolymer type by NMR experiments

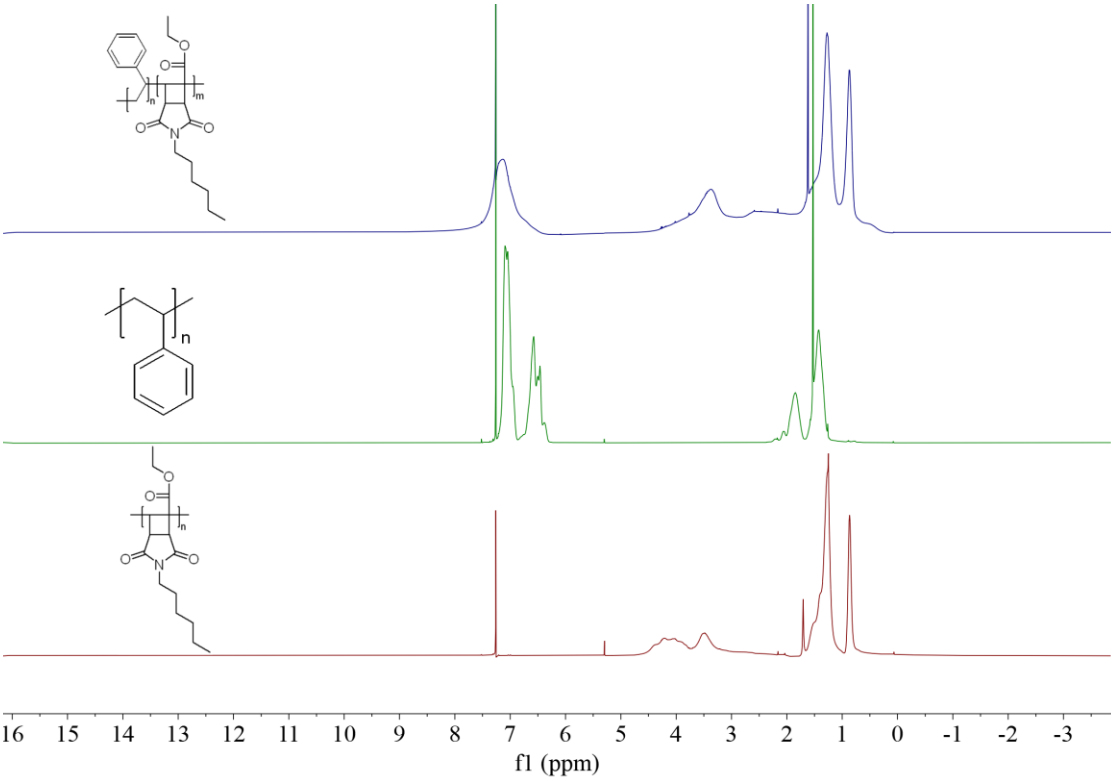

Fig. S1 ^{1}H NMR spectra (400 MHz, Chloroform-*d*) of PS_{55}-*co*-$PHCBI_{45}$, PS and PHCBI.

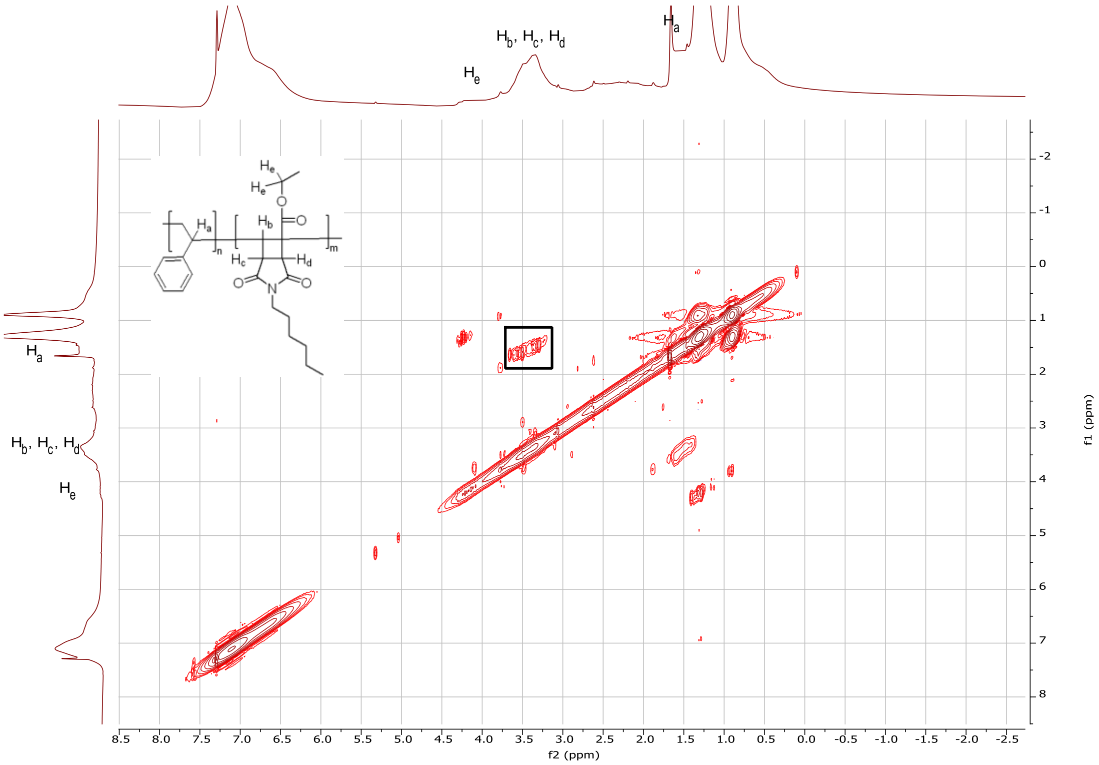

Fig. S2 NOESY NMR spectra (400 MHz, Chloroform-*d*) of PS_{55}-*co*-$PHCBI_{45}$. The strong correlation signals shown in the box between protons H_a and H_b, H_c, H_d, H_e prove that the copolymer did not contain blocks of the individual monomers (There would not be such strong correlation signals for a block copolymer).

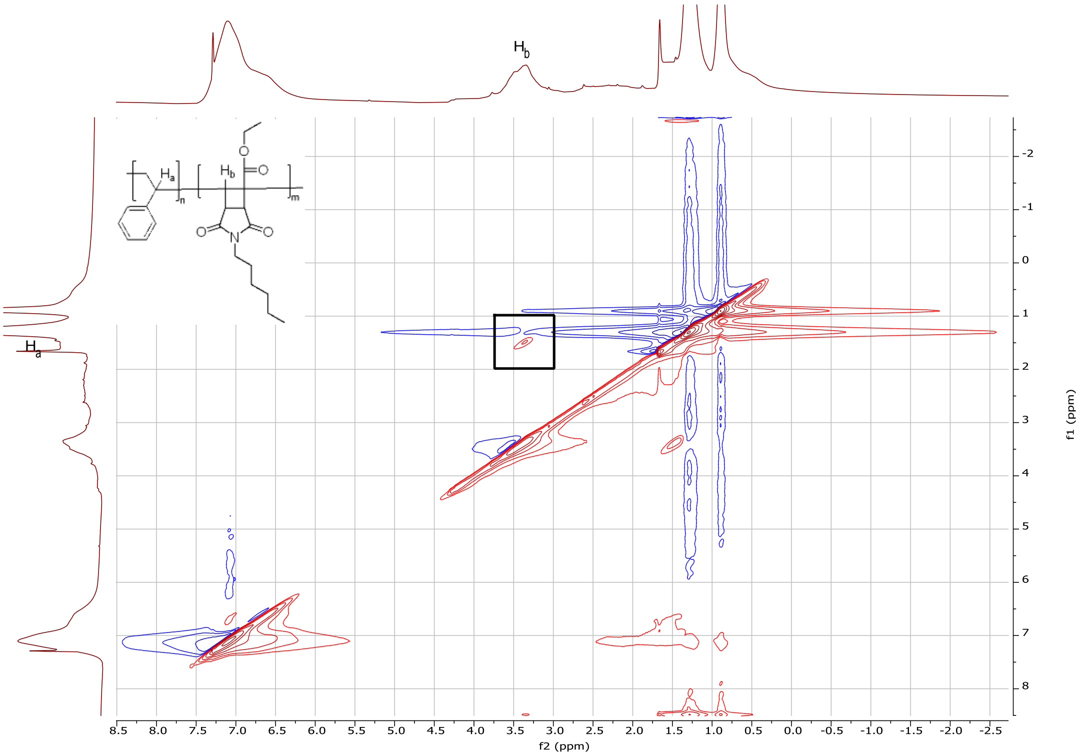

Fig. S3 COSY NMR spectra (400 MHz, Chloroform-*d*) of PS_{52}-*co*-$PHCBI_{48}$. The strong correlation signals shown in box between protons H_a and H_b prove that the copolymer did not contain blocks of the individual monomers (There would not be such strong correlation signals for a block copolymer).

Determination of monomer reactivity ratios

Table S1 Free radical copolymerizations of CBI and styrene to determine monomer reactivity ratios

Entry	$f_{HCBI}/f_{styrene}$ [a]	React. time (h)	Conversion[a]			$F_{HCBI}/F_{styrene}$ [a]	Yield[b]	SEC	
			Styrene	HCBI	Total			M_n (kg·mol^{-1})	*Đ*
**1**	6/94	2	12%	18%	18%	36/64	3.1%	76	1.72
**2**	12/88	2	9%	8%	8%	46/54	1.3%	67	1.78
**3**	19/81	2	15%	10%	11%	50/50	1.7%	72	1.65
**4**	25/75	2	16%	9%	11%	53/47	1.5%	65	1.74
**5**	35/65	1.5	20%	12%	15%	57/43	2.3%	55	1.69
**6**	40/60	1.5	20%	7%	12%	58/42	1.8%	45	1.73

[a] Determined by ^{1}H NMR spectroscopy with 1,3,5-trimethoxybenzene as internal standard. [b] Determined after precipitation.

The mole fractions of HCBI and styrene in the monomer feed are represented by f_{HCBI} and $f_{styrene}$. F_{HCBI} and $F_{styrene}$ are the mole fractions of the corresponding residues in the copolymers.

The analysis according to the Fineman-Ross linearization method[6] was based on:

$$f(F\text{-}1)/F = r_{HCBI}(f^{\,2}/F) - r_{styrene}$$

where $f = f_{HCBI}/f_{styrene}$ and $F = F_{HCBI}/F_{styrene}$. r_{HCBI} and $r_{styrene}$ are the reactivity ratios of the two monomers.

The analysis according to Fineman-Ross (Fig. S4) affords a reactivity ratio $r_{CBI} = 0.74$ for HCBI and a reactivity ratio $r_{styrene} = 0.04$ for styrene.

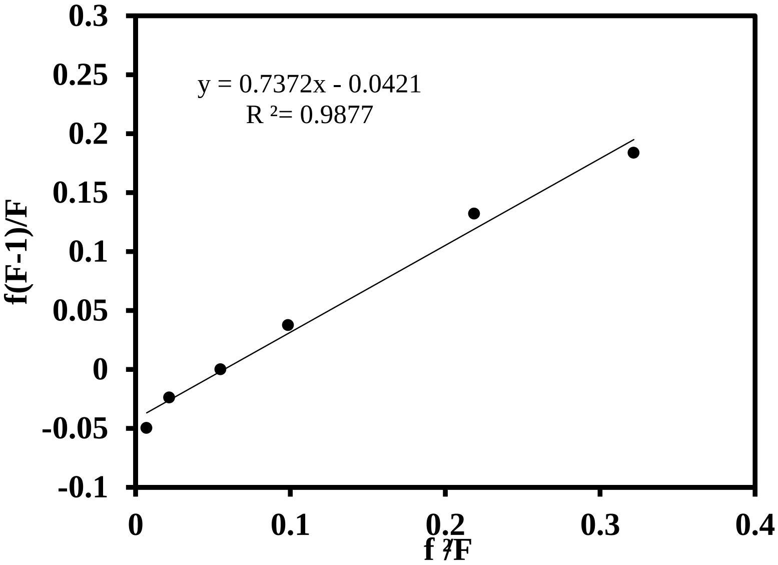

Fig. S4 Fineman-Ross plot for the free radical copolymerization of HCBI and styrene (The data shown here is also presented in Fig. 2 of the main manuscript) under bulk condition at 65 ℃ with AIBN as initiator.

The analysis according to the Kelen-Tüdos linearization method[7] was based on:

$$\eta = (r_{HCBI} + (r_{styrene}/\ \alpha))\ \zeta - (r_{styrene}/\ \alpha)$$

where $\eta = (f(F\text{-}1))/(F(\alpha + (f^2/F))$, $\zeta = (f^2/F)/(\alpha + (f^2/F)$

and $\alpha = ((f^2/F)_{max} \times (f^2/F)_{min})^{0.5}$.

The analysis according to Kelen-Tüdos affords a reactivity ratio $r_{HCBI} = 0.82$ for HCBI and a reactivity ratio $r_{styrene} = 0.05$ for styrene.

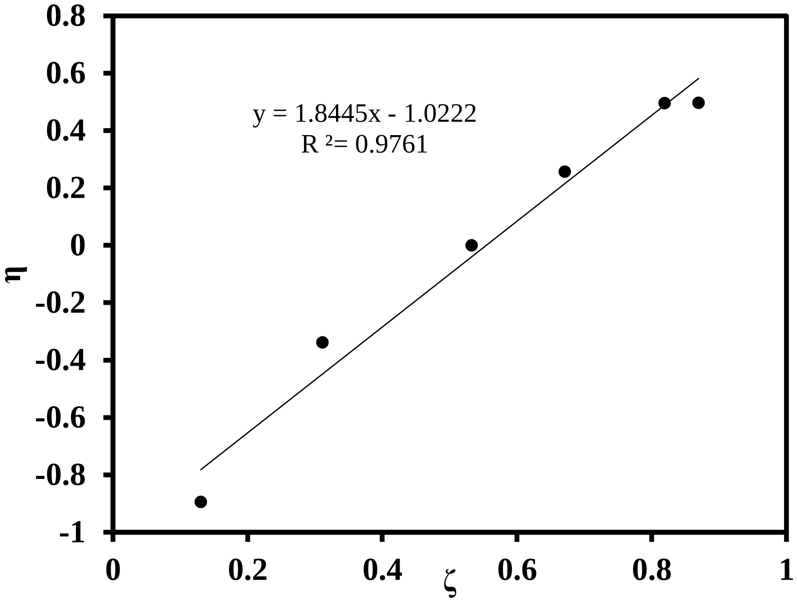

Fig. S5 Kelen-Tüdos plot for the free radical copolymerization of HCBI and styrene (The data shown here is also presented in Fig. 2 of the main manuscript) under bulk condition at 65 ℃ with AIBN as initiator.

The analysis according to the Mayo-Lewis nonlinear least-squares method[8,9] was based on:

$$F_{HCBI} = (r_{HCBI}f_{HCBI}^2 + f_{HCBI}f_{styrene})/(r_{HCBI}f_{HCBI}^2 + 2\, f_{HCBI}f_{styrene} + r_{styrene}f_{styrene}^2)$$

The analysis according to Mayo-Lewis affords a reactivity ratio r_{CBI} = 0.81 for HCBI and a reactivity ratio $r_{styrene}$ = 0.05 for styrene.

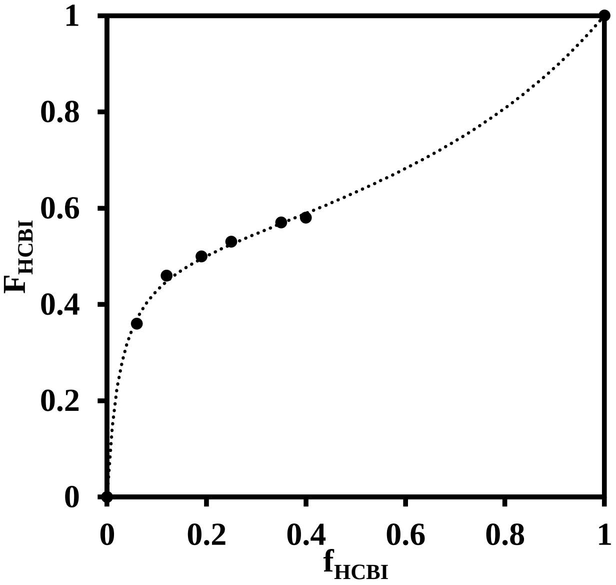

Fig. S6 Mayo-Lewis plot for the free radical copolymerization of HCBI and styrene (The data shown here is also presented in Fig. 2 of the main manuscript) under bulk condition at 65 ℃ with AIBN as initiator.

TGA and DSC data

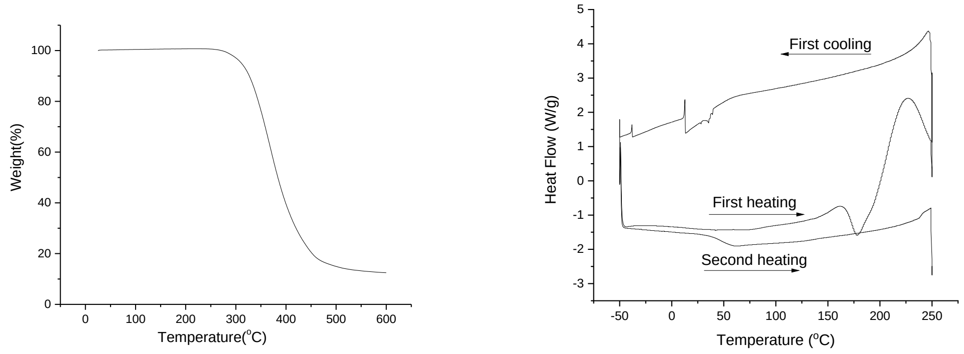

Fig. S7 TGA (left) and DSC (right) traces of PHCBI.

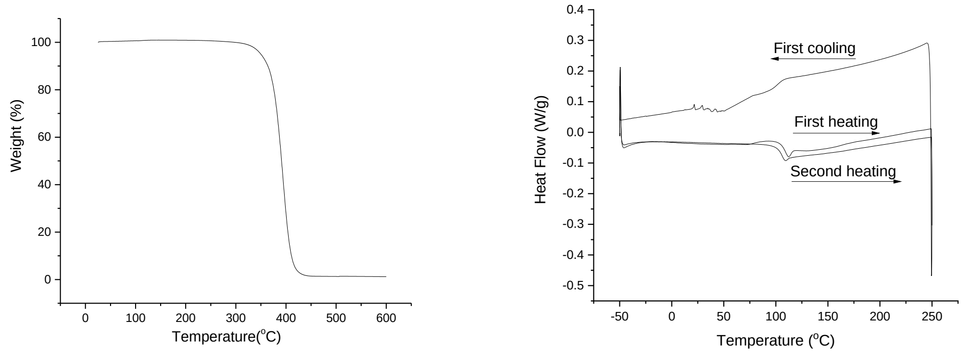

Fig. S8 TGA (left) and DSC (right) traces of PS_{82}-*co*-$PHCBI_{18}$.

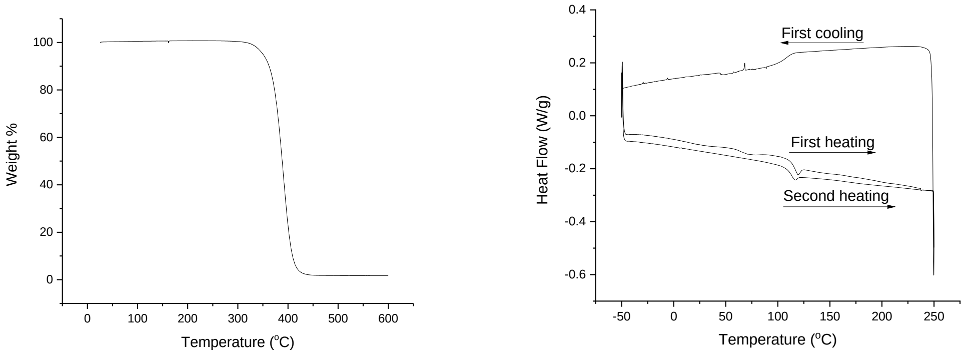

Fig. S9 TGA (left) and DSC (right) traces of PS_{75}-*co*-$PHCBI_{25}$.

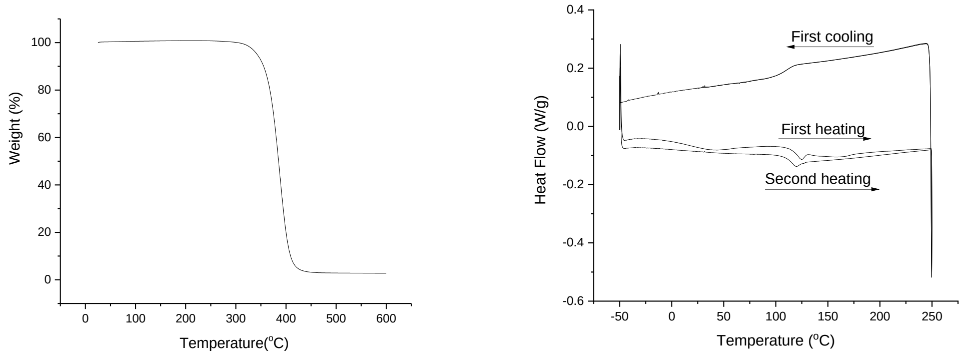

Fig. S10 TGA (left) and DSC (right) traces of PS$_{60}$-*co*-PHCBI$_{40}$.

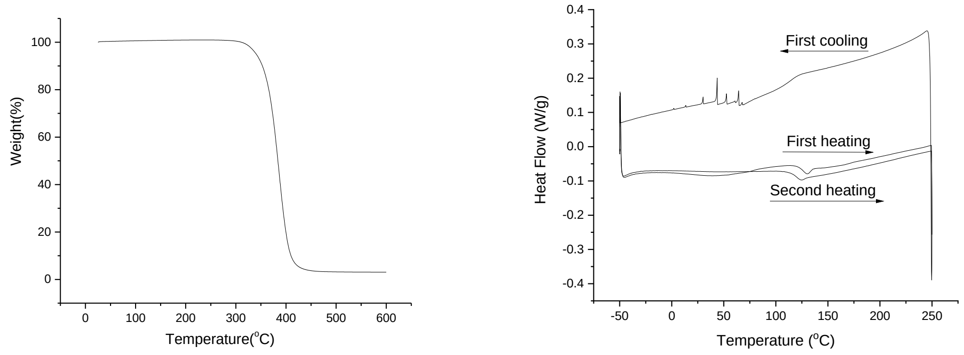

Fig. S11 TGA (left) and DSC (right) traces of PS$_{55}$-*co*-PHCBI$_{45}$.

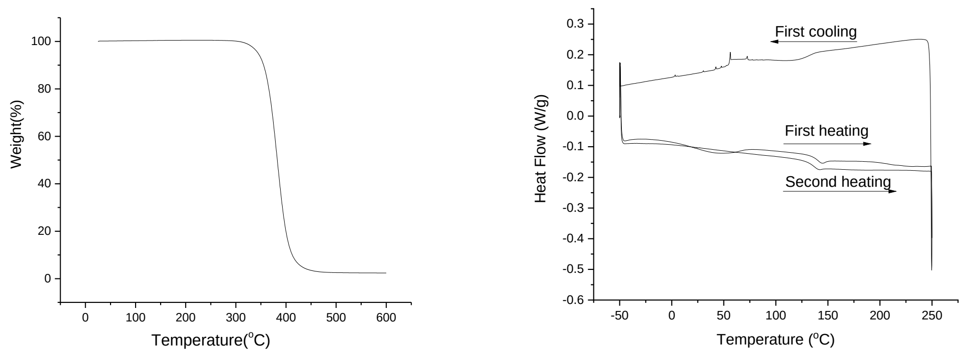

Fig. S12 TGA (left) and DSC (right) traces of PS$_{49}$-*co*-PHCBI$_{51}$.

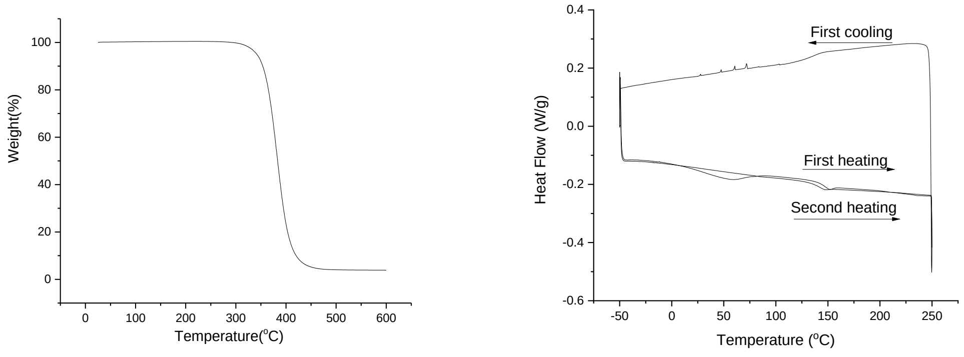

Fig. S13 TGA (left) and DSC (right) traces of PS_{45}-*co*-$PHCBI_{55}$.

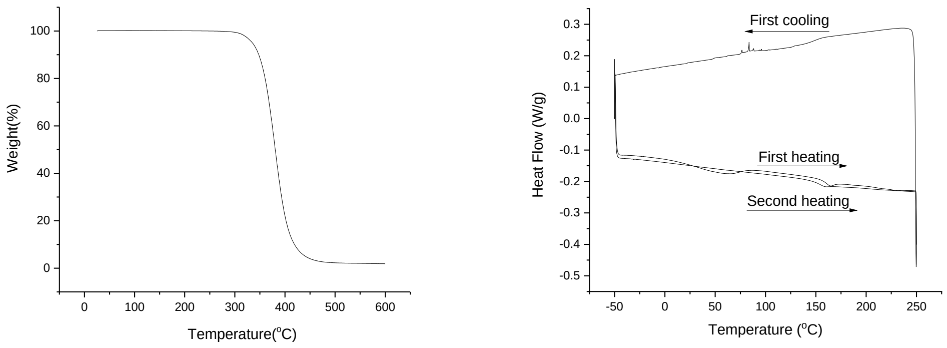

Fig.S14 TGA (left) and DSC (right) traces of PS_{38}-*co*-$PHCBI_{62}$.

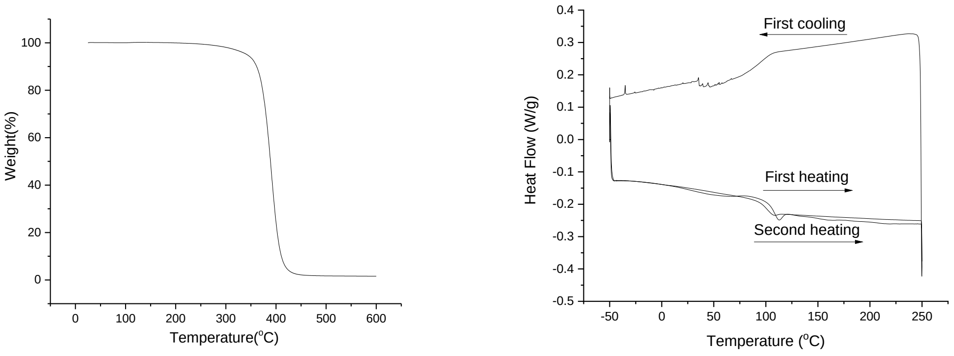

Fig. S15 TGA (left) and DSC (right) traces of PS_{73}-*co*-$PHCBI_{27}$.

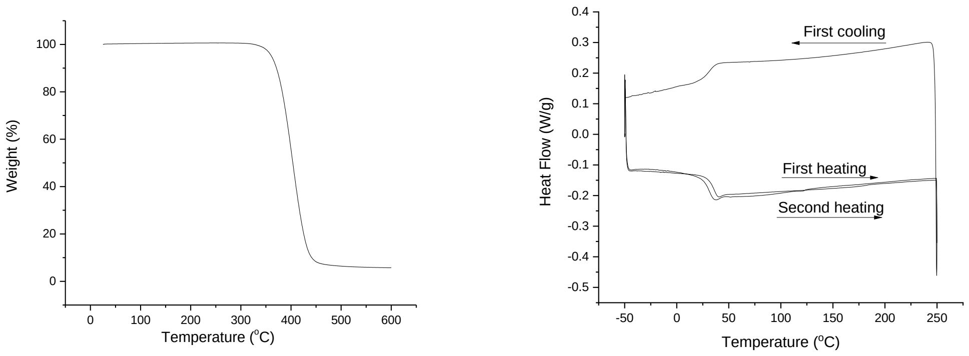

Fig. S16 TGA (left) and DSC (right) traces of PMA_{87}-*co*-$PHCBI_{13}$.

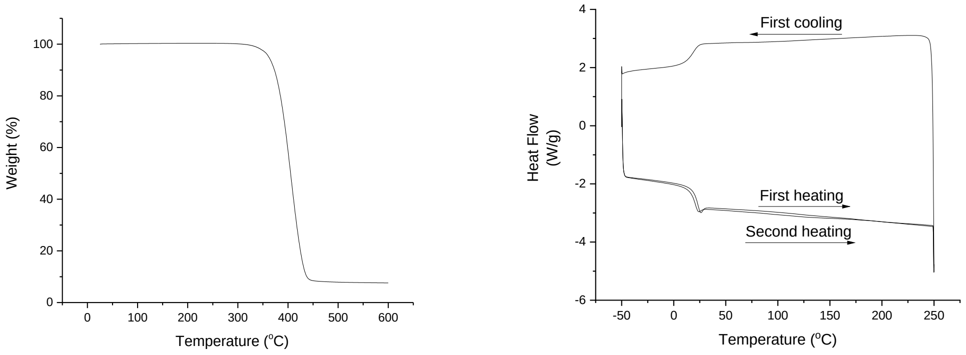

Fig. S17 TGA (left) and DSC (right) traces of PMA$_{97}$-*co*-PHCBI$_3$.

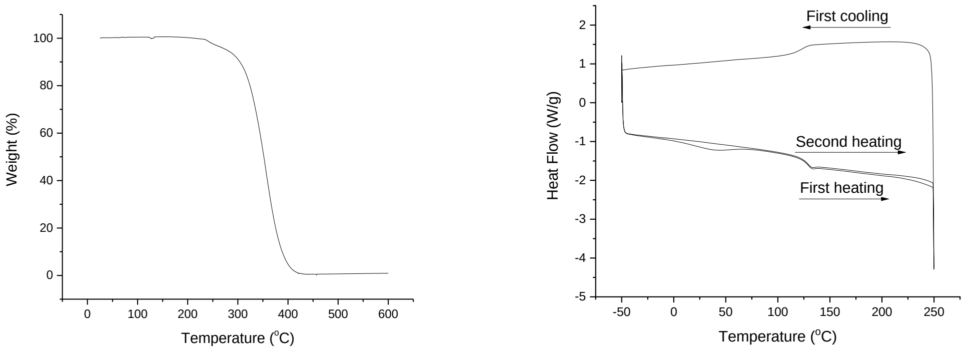

Fig. S18 TGA (left) and DSC (right) traces of PMMA$_{98}$-*co*-PHCBI$_2$.

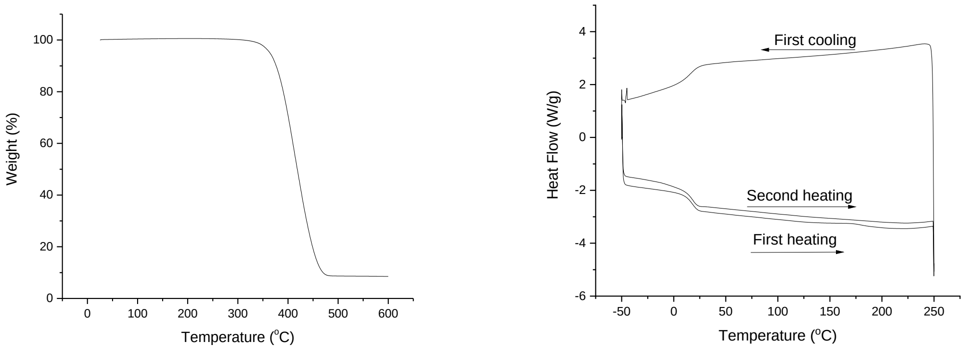

Fig. S19 TGA (left) and DSC (right) traces of PS$_{34}$-*co*-PB$_{49}$-*co*-PHCBI$_{17}$.

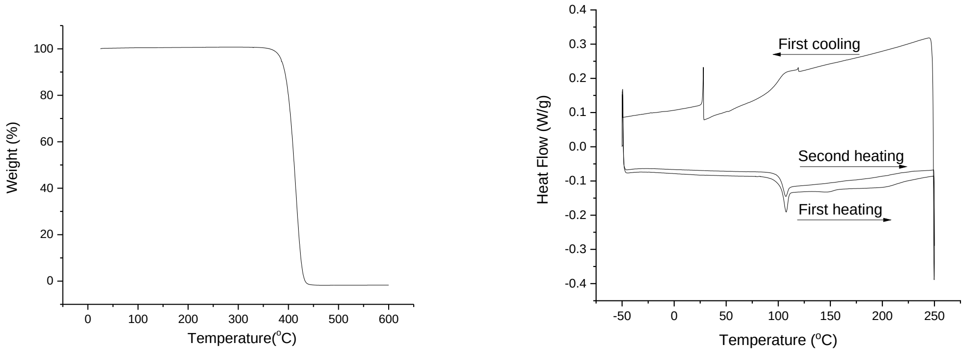

Fig. S20 TGA (left) and DSC (right) traces of PS.

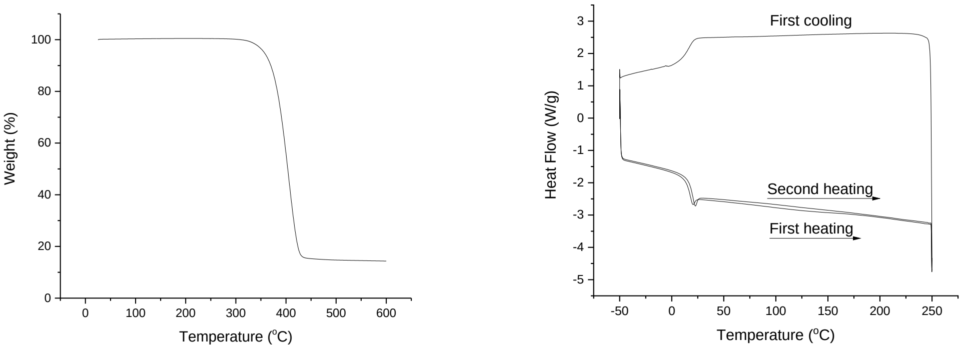

Fig. S21 TGA (left) and DSC (right) traces of PMA.

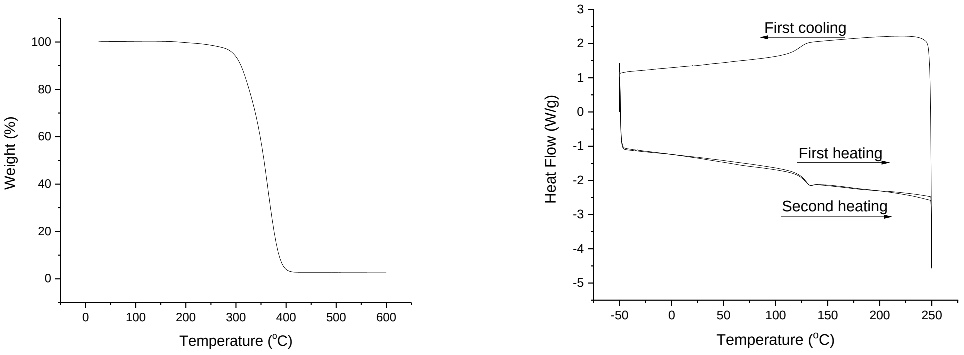

Fig. S22 TGA (left) and DSC (right) traces of PMMA.

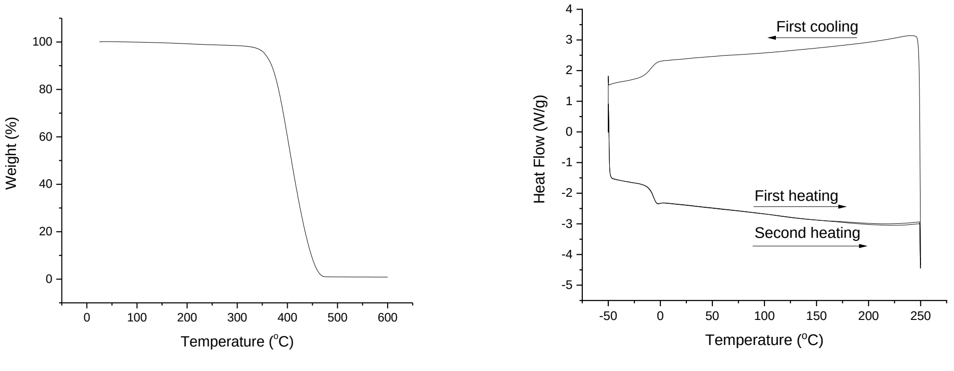

Fig. S23 TGA (left) and DSC (right) traces of PS_{47}-*co*-PB_{53}.

Dynamic mechanical analysis (DMA) data

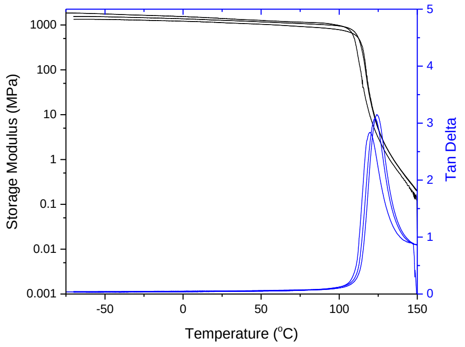

Fig. S24 DMA traces (three different samples) of PS_{82}-*co*-$PHCBI_{18}$.

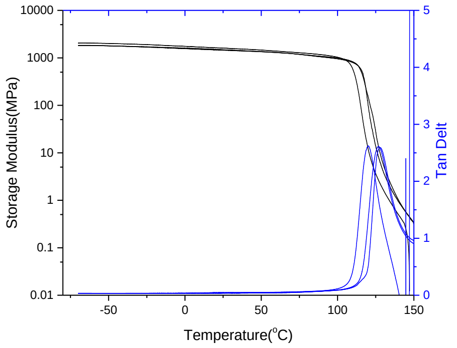

Fig. S25 DMA traces (three different samples) of PS_{75}-*co*-$PHCBI_{25}$.

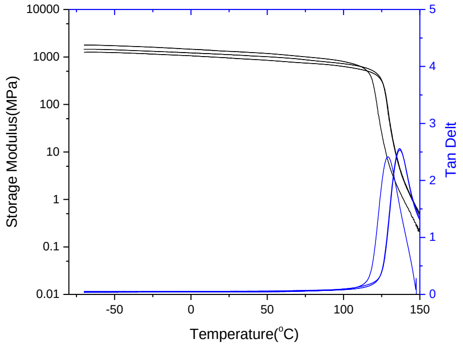

Fig. S26 DMA traces (three different samples) of PS_{60}-*co*-$PHCBI_{40}$.

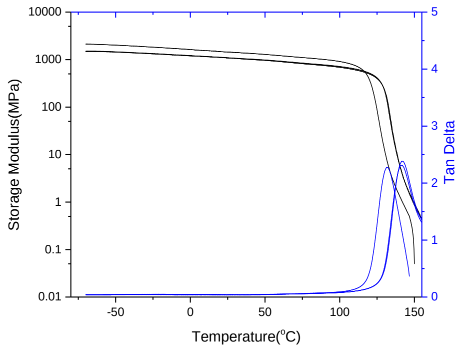

Fig. S27 DMA traces (three different samples) of PS_{55}-*co*-$PHCBI_{45}$.

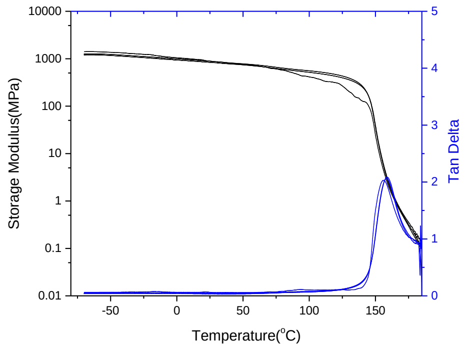

Fig. S28 DMA traces (three different samples) of PS_{49}-*co*-$PHCBI_{51}$.

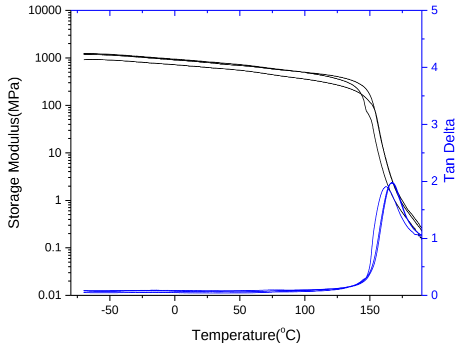

Fig. S29 DMA traces (three different samples) of PS_{45}-*co*-$PHCBI_{55}$.

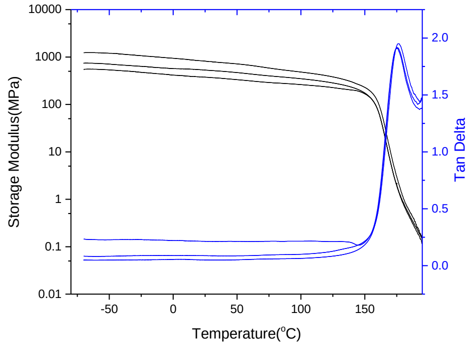

Fig. S30 DMA traces (three different samples) of PS_{38}-*co*-$PHCBI_{62}$.

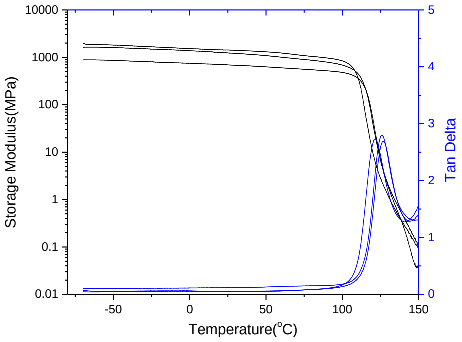

Fig. S31 DMA traces (three different samples) of PS_{73}-*co*-$PHCBI_{27}$.

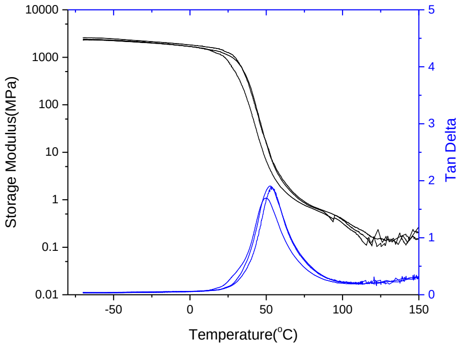

Fig. S32 DMA traces (three different samples) of PMA_{87}-*co*-$PHCBI_{13}$.

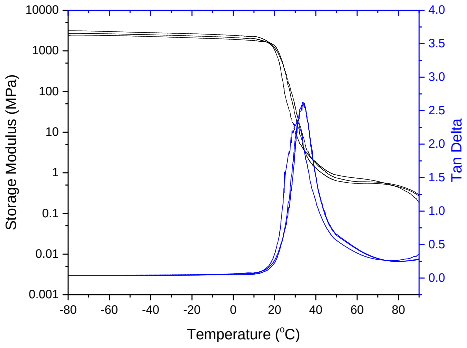

Fig. S33 DMA traces (three different samples) of PMA_{97}-*co*-$PHCBI_3$.

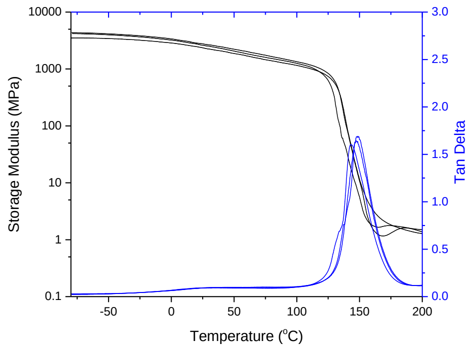

Fig. S34 DMA traces (three different samples) of PMMA$_{98}$-*co*-PHCBI$_2$.

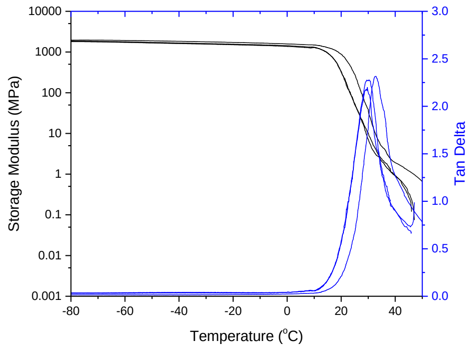

Fig. S35 DMA traces (three different samples) of PS$_{34}$-*co*-PB$_{49}$-*co*-PHCBI$_{17}$.

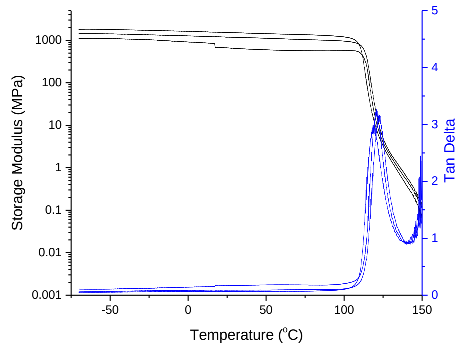

Fig. S36 DMA traces (three different samples) of PS.

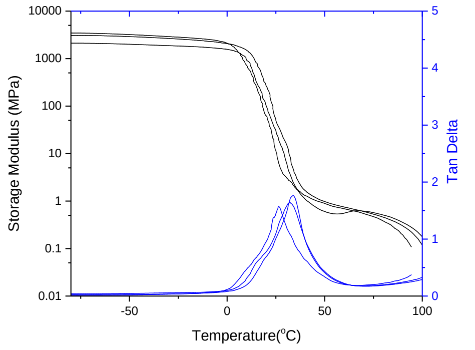

Fig. S37 DMA traces (three different samples) of PMA.

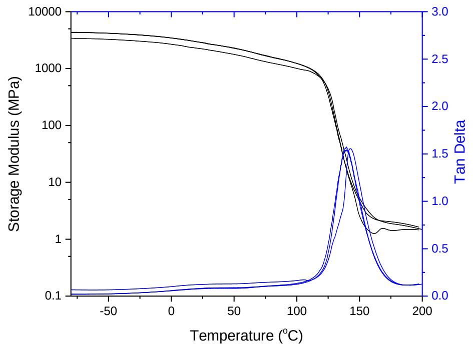

Fig. S38 DMA traces (three different samples) of PMMA.

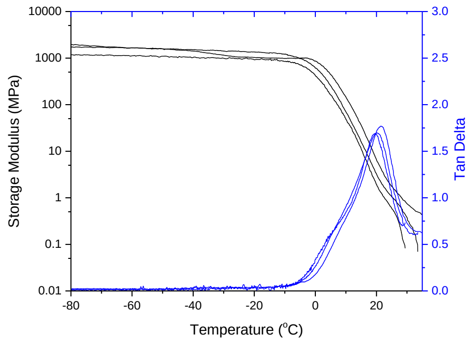

Fig. S39 DMA traces (three different samples) of PS_{47}-*co*-PB_{53}.

Thermal stability experiments of PHCBI and PS-*co*-PHCBI

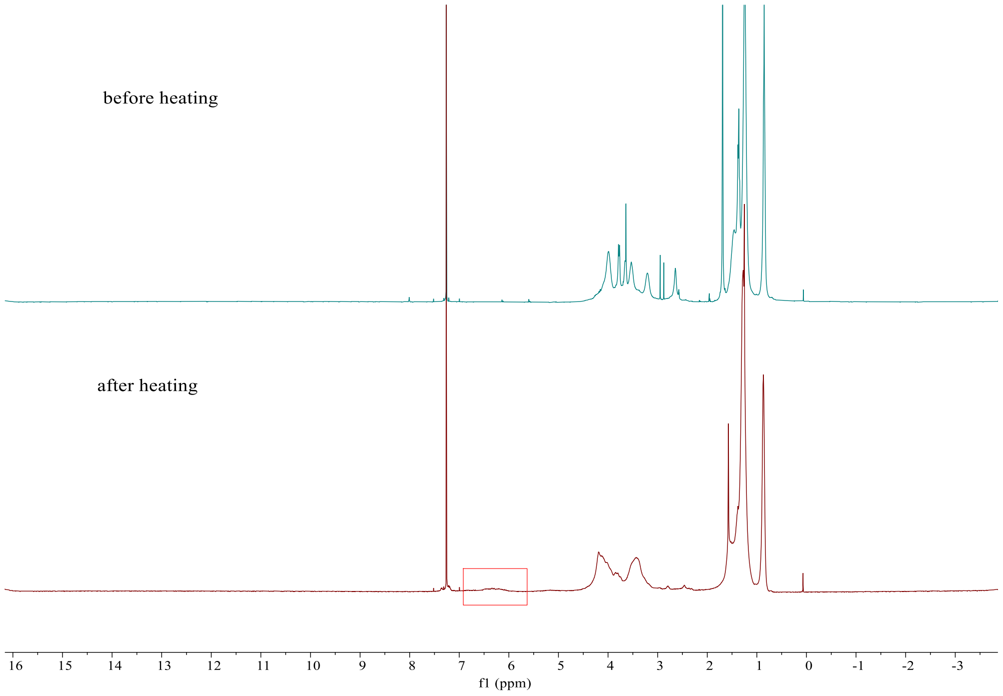

Fig. S40 ^1H NMR spectra (400 MHz, $CDCl_3$) of PHCBI before and after heating under Ar atmosphere for 30 min at 180 ℃. New signals in the red box are assigned to ring-opened cyclobutanes. Other proton signals change can also be seen between 3 and 4.5 ppm.

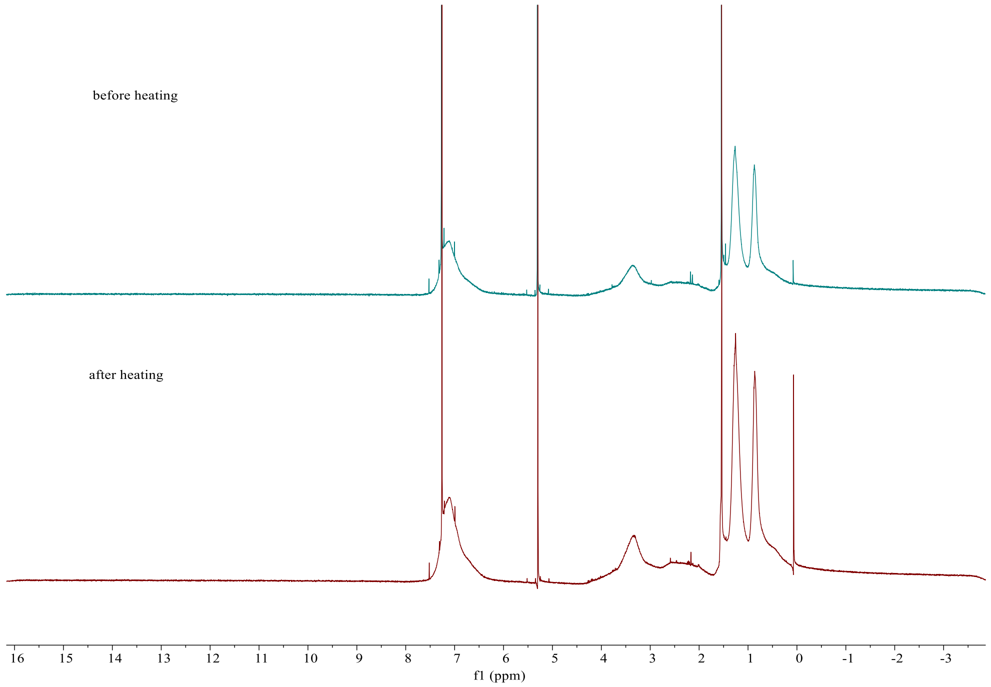

Fig. S41 ^{1}H NMR spectra (400 MHz, $CDCl_3$) of PS_{49}-*co*-$PHCBI_{51}$ before and after heating under Ar atmosphere for 30 min at 180 ℃. The similarity between the two spectra indicates that the copolymers are stable at 180 ℃.

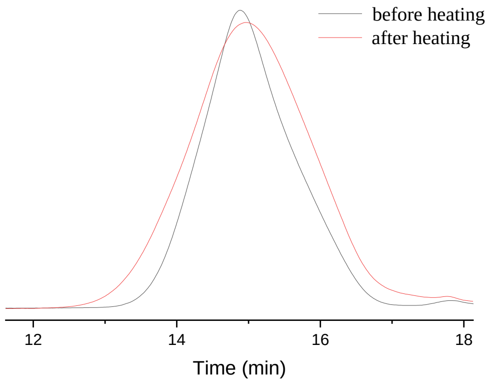

Fig. S42 SEC trace of PHCBI before and after heating under Ar atmosphere for 30 min at 180 ℃.

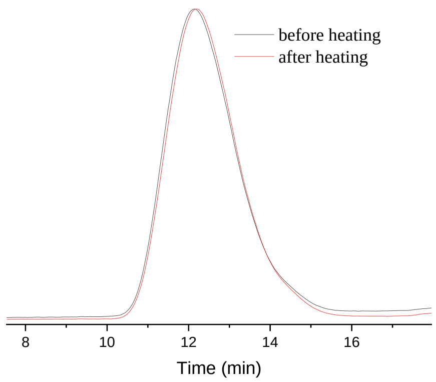

Fig. S43 SEC trace of PS_{49}-*co*-$PHCBI_{51}$ before and after heating under Ar atmosphere for 30 min at 180 ℃. The similarity between the two SEC traces indicates that the copolymers are stable at 180 ℃.

Effect of ultrasonication on polymers monitored by ^{1}H NMR and FT-IR measurements

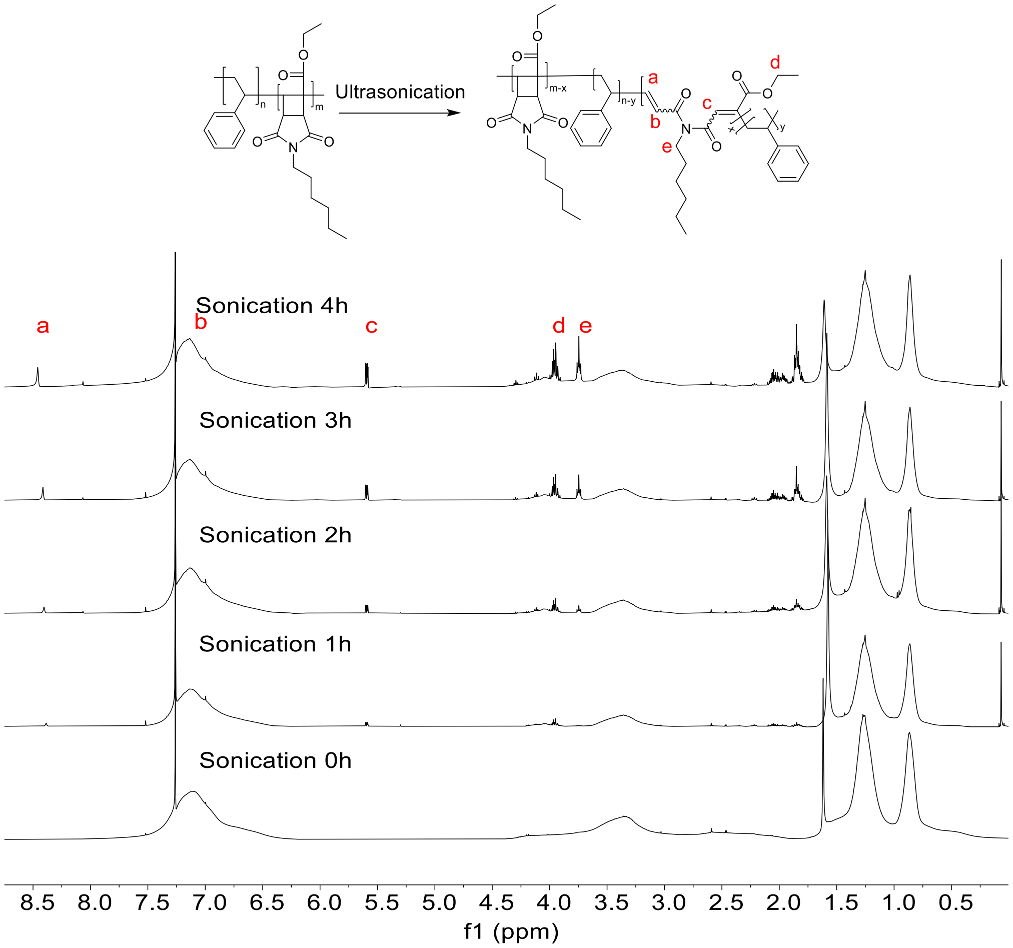

Fig. S44 ^{1}H NMR spectra (400 MHz, Chloroform-*d*) of PS_{50}-*co*-$PHCBI_{50}$ after sonication for the time indicated (magnified portions of the spectra are shown in Fig. 4 of the main manuscript).

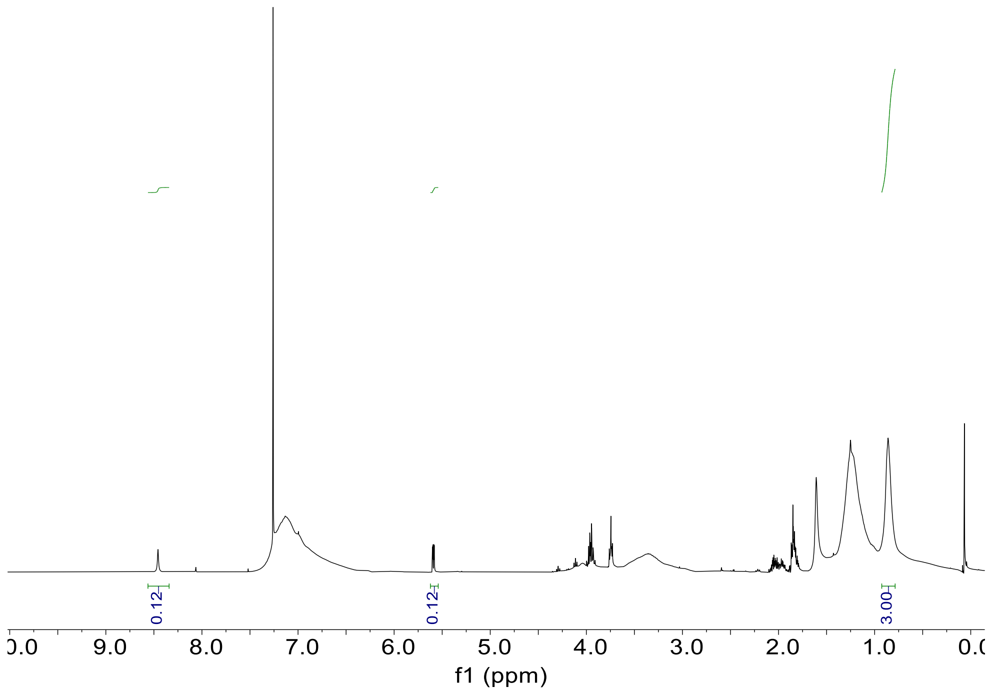

Fig. S45 ^{1}H NMR spectrum (400 MHz, Chloroform-*d*) of PS_{50}-*co*-$PHCBI_{50}$ after sonication for 240 min.

The peak at 0.8 ppm is assigned to the -CH_3 in *N*-hexyl, while the peaks at 5.60 ppm and 8.46 ppm are assigned to the alkene peaks formed upon the cyclobutane ring-opening. If all of the cyclobutane rings would have opened, the integrals of both these peaks at 5.60 ppm and 8.46 ppm should be 1. Therefore, based on ^{1}H NMR measurements, the fraction of ring-opened cyclobutane is $\frac{0.12}{1} \times 100\% = 12\%$.

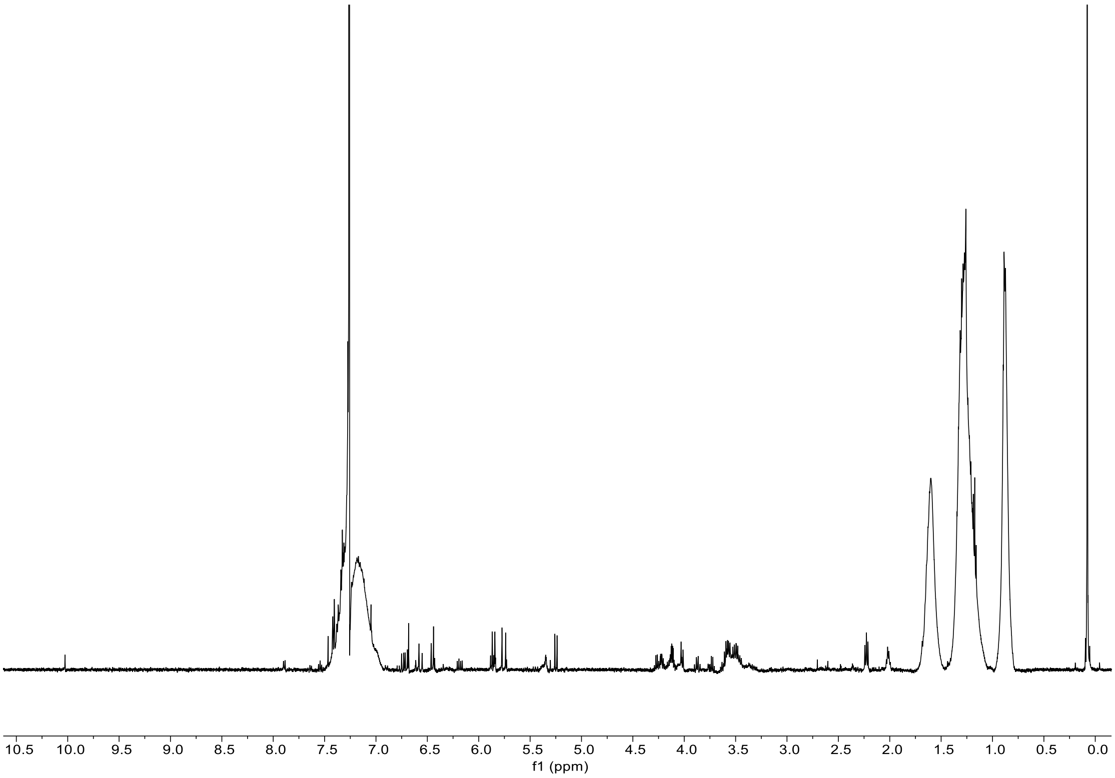

Fig. S46 ^{1}H NMR spectrum (400 MHz, $CDCl_3$) of PS_{50}-*co*-$PHCBI_{50}$ after cryo-milling for 60 min. (The alkene peaks are too messy to quantify the amount of cyclobutane ring-opening.)

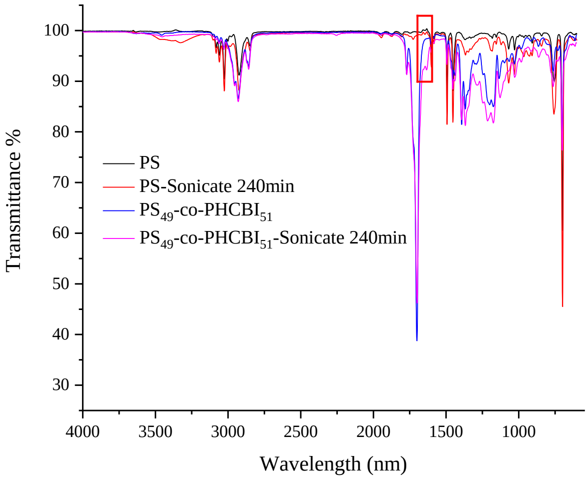

Fig. S47 IR spectra of PS and PS_{49}-*co*-$PHCBI_{51}$ before and after sonication (4 mg mL^{-1} in THF). The new signals in the red boxes of the IR spectra indicate carbon-carbon double bond formation associated with cyclobutane ring opening.

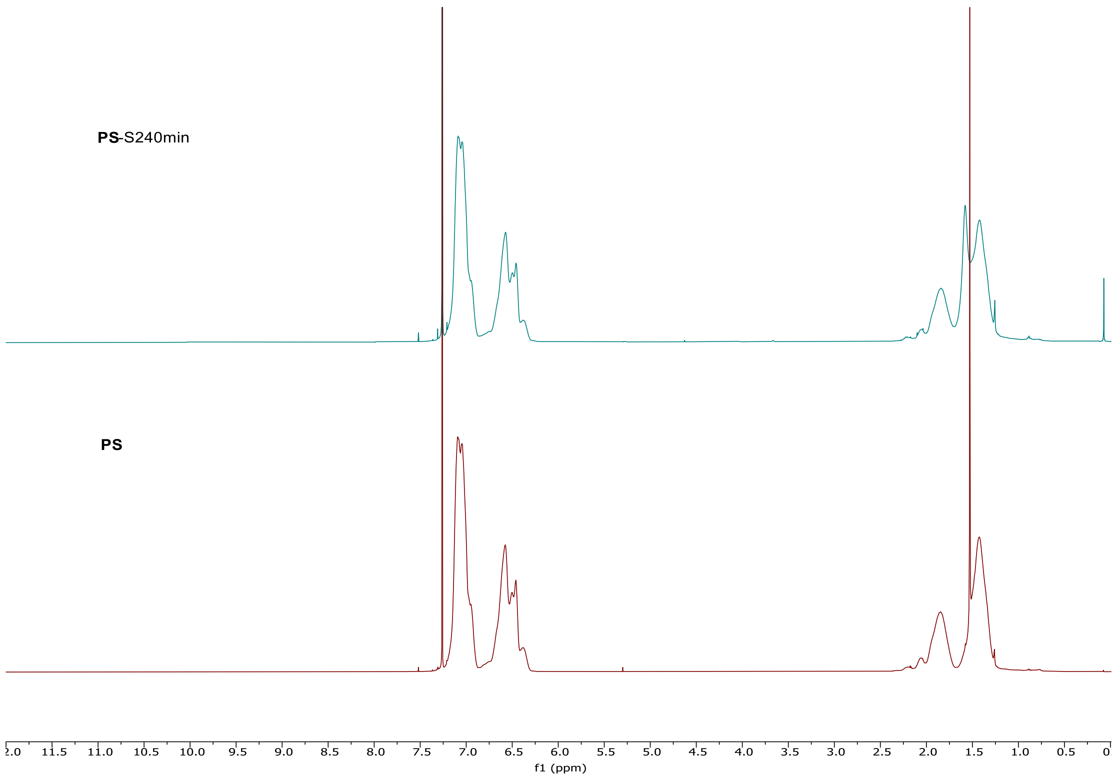

Fig. S48 ^{1}H NMR spectrum (400 MHz, Chloroform-*d*) of PS after sonication for 240 min (full spectrum). There was no change observed after sonication for 240min.

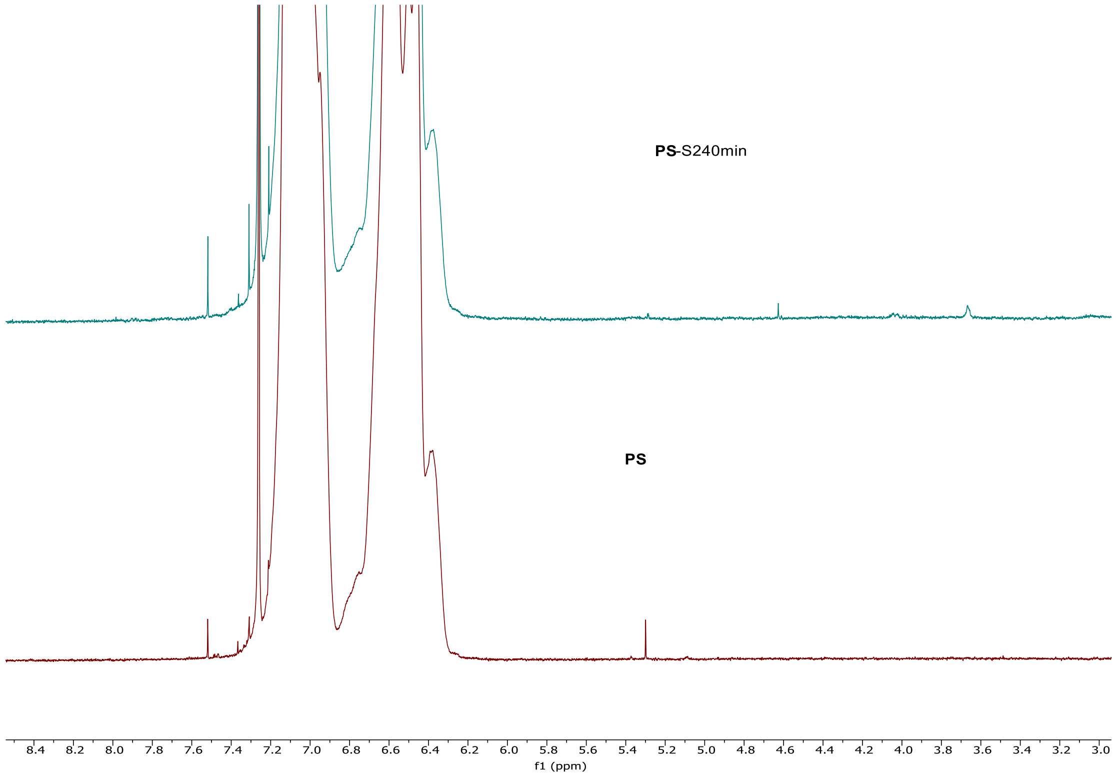

Fig. S49 ^{1}H NMR spectrum (400 MHz, Chloroform-*d*) of PS after sonication for 240 min (zoomed in spectrum). There was no change observed after sonication for 240min.

Ultrasonication of polymers followed by NaOH solution treatment

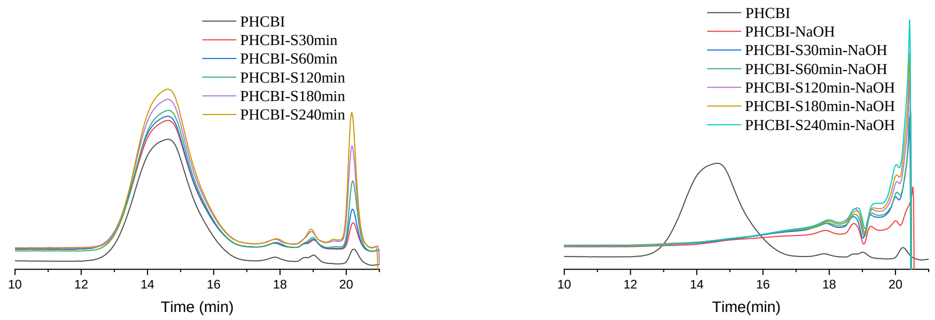

Fig. S50 SEC traces of PHCBI after sonication (left) and sonication-hydrolysis (right) (differential refractive index (dRI) signal). (After the NaOH solution addition, the mixture became dark blue and the polymers precipitated out from THF. This seems most likely because the formation of carboxylate salts. Therefore, there is no signal in SEC after NaOH treatment).

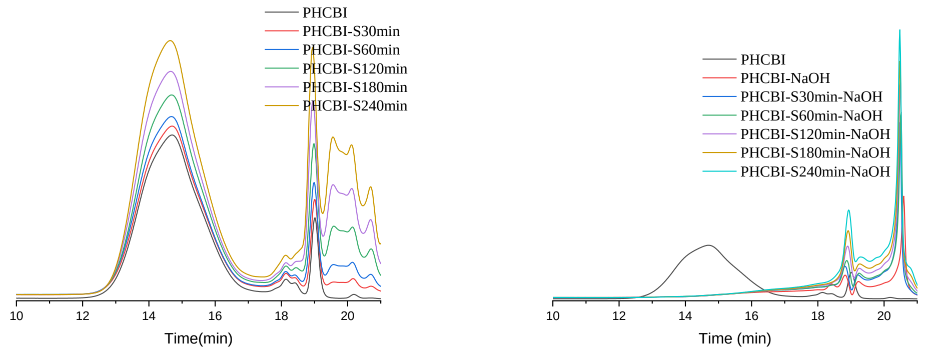

Fig. S51 SEC traces of PHCBI after sonication (left) and sonication-hydrolysis (right) (UV signal). (After the NaOH solution addition, the mixture became dark blue and the polymers precipitated out from THF. This seems most likely because the formation of carboxylate salts. Therefore, there is no signal in SEC after NaOH treatment).

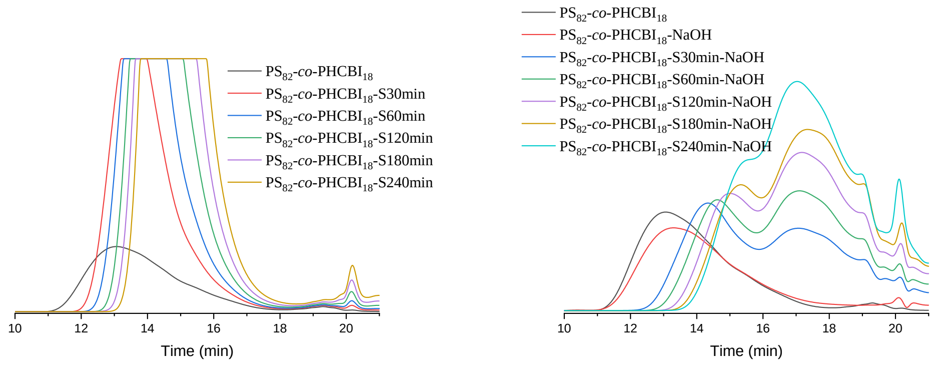

Fig. S52 SEC traces of PS_{82}-*co*-$PHCBI_{18}$ after sonication (left) and sonication-hydrolysis (right) (UV signal).

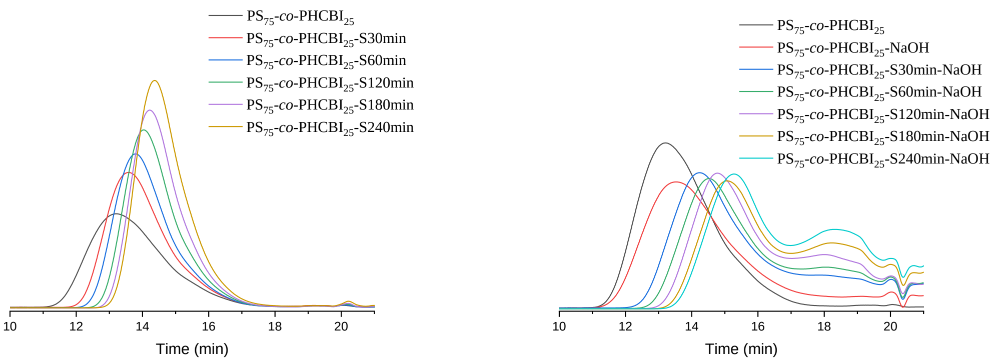

Fig. S53 SEC traces of PS_{75}-co-$PHCBI_{25}$ after sonication (left) and sonication-hydrolysis (right) (dRI signal).

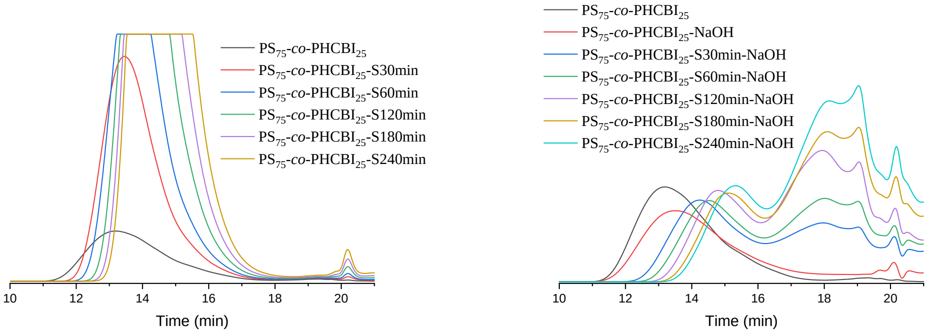

Fig. S54 SEC traces of PS_{75}-*co*-$PHCBI_{25}$ after sonication (left) and sonication-hydrolysis (right) (UV signal).

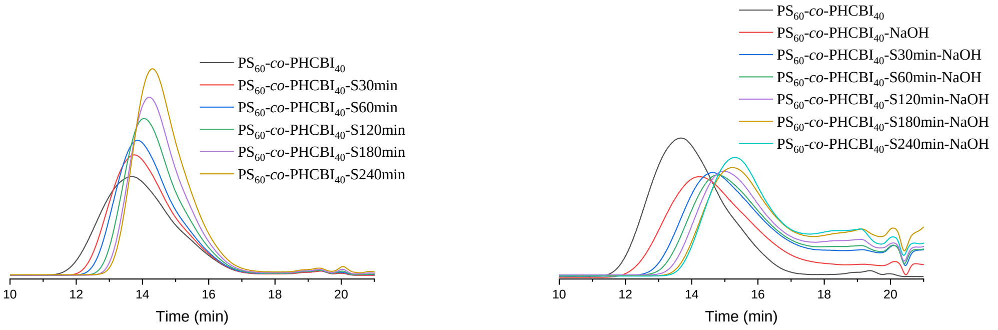

Fig. S55 SEC traces of PS_{60}-*co*-$PHCBI_{40}$ after sonication (left) and sonication-hydrolysis (right) (dRI signal).

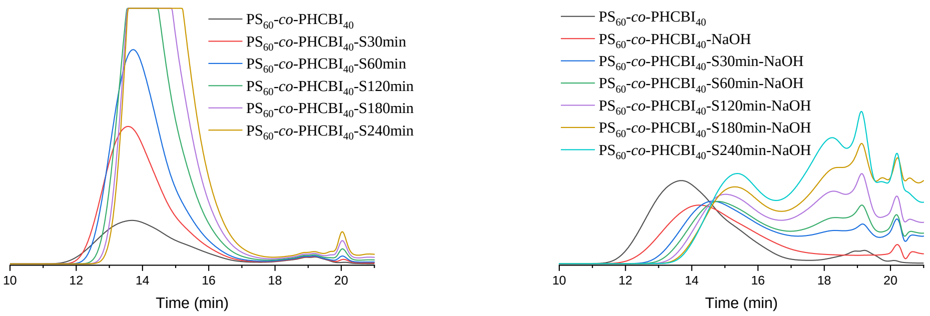

Fig. S56 SEC traces of PS_{60}-*co*-$PHCBI_{40}$ after sonication (left) and sonication-hydrolysis (right) (UV signal).

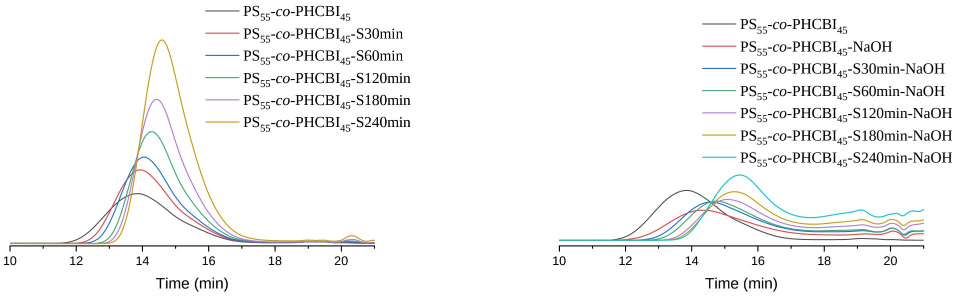

Fig. S57 SEC traces of PS_{55}-*co*-$PHCBI_{45}$ after sonication (left) and sonication-hydrolysis (right) (dRI signal).

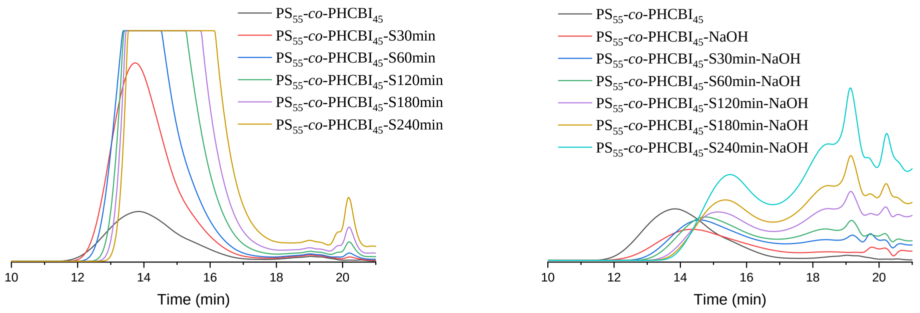

Fig. S58 SEC traces of PS_{55}-*co*-$PHCBI_{45}$ after sonication (left) and sonication-hydrolysis (right) (UV signal).

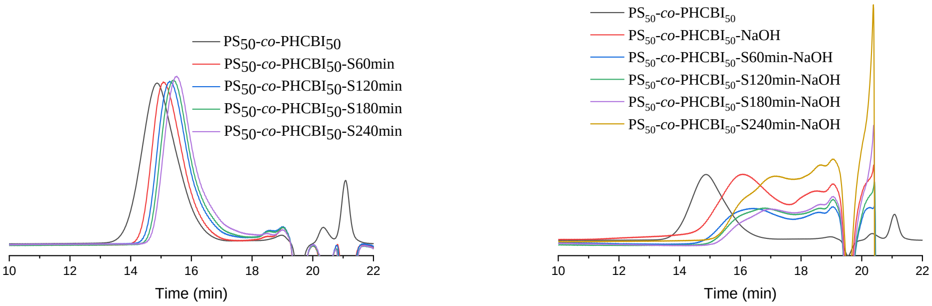

Fig. S59 SEC traces of PS_{50}-*co*-$PHCBI_{50}$ after sonication (left) and sonication-hydrolysis (right) (dRI signal).

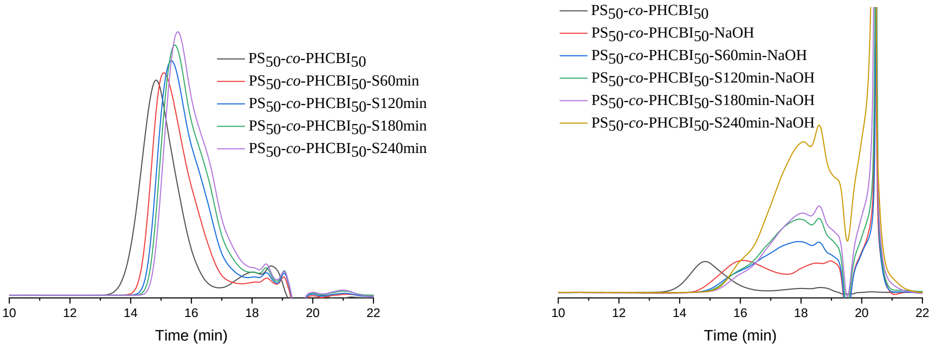

Fig. S60 SEC traces of PS_{50}-*co*-$PHCB_{50}$ after sonication (left) and sonication-hydrolysis (right) (UV signal).

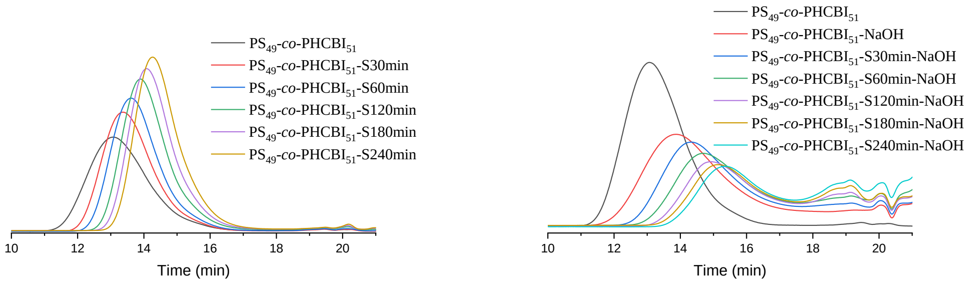

Fig. S61 SEC traces of PS_{49}-*co*-$PHCBI_{51}$ after sonication (left) and sonication-hydrolysis (right) (dRI signal).

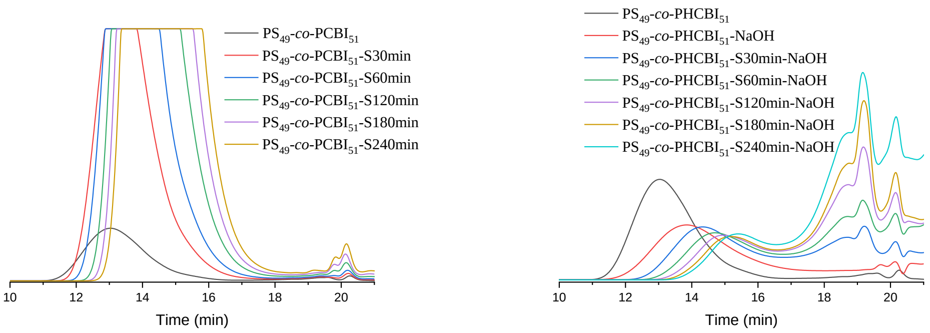

Fig. S62 SEC traces of PS_{49}-*co*-$PHCBI_{51}$ after sonication (left) and sonication-hydrolysis (right) (UV signal).

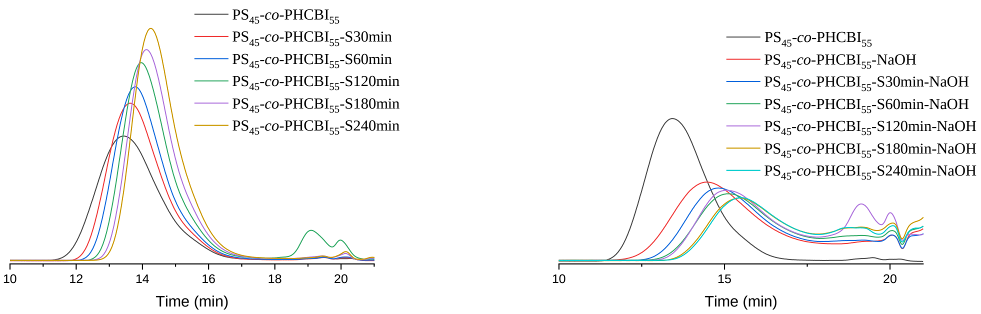

Fig. S63 SEC traces of PS_{45}-*co*-$PHCBI_{55}$ after sonication (left) and sonication-hydrolysis (right) (dRI signal).

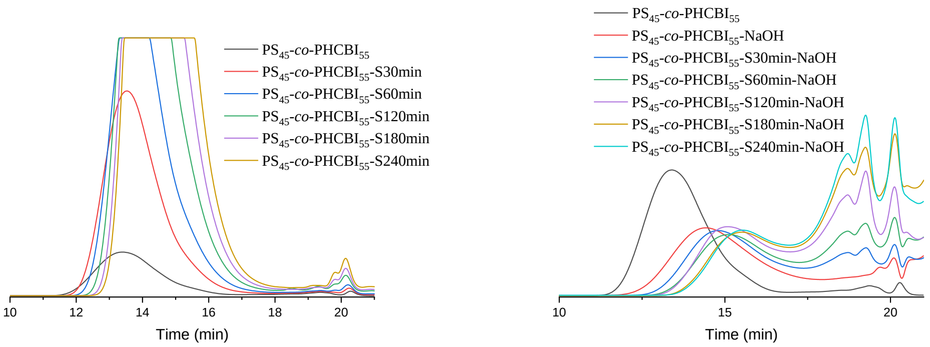

Fig. S64 SEC traces of PS_{45}-*co*-$PHCBI_{55}$ after sonication (left) and sonication-hydrolysis (right) (UV signal).

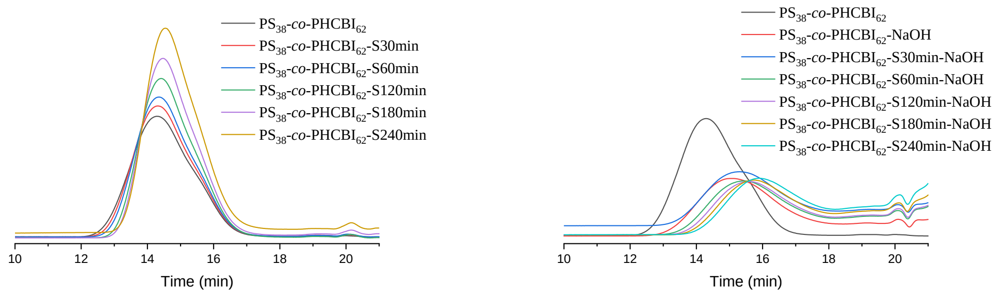

Fig. S65 SEC traces of PS_{38}-*co*-$PHCBI_{62}$ after sonication (left) and sonication-hydrolysis (right) (dRI signal).

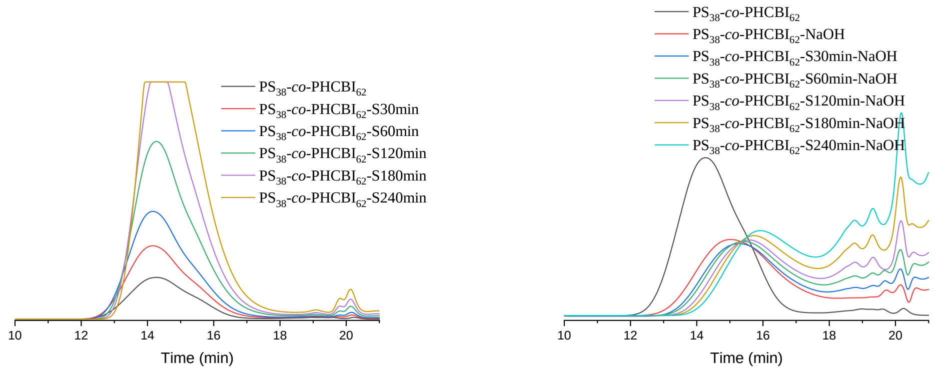

Fig. S66 SEC traces of PS_{38}-*co*-$PHCBI_{62}$ after sonication (left) and sonication-hydrolysis (right) (UV signal).

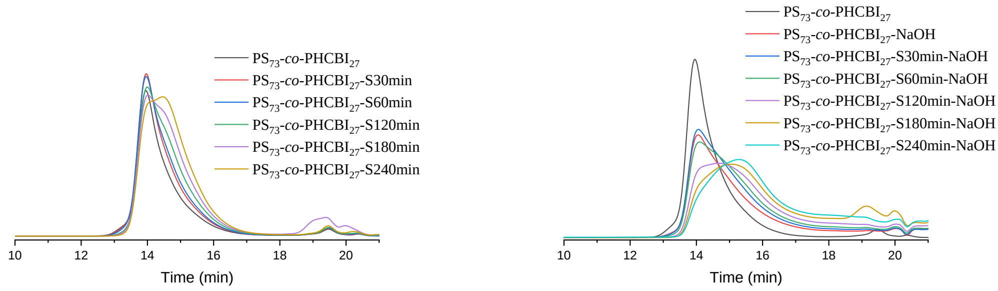

Fig. S67 SEC traces of PS_{73}-*co*-$PHCBI_{27}$ after sonication (left) and sonication-hydrolysis (right) (dRI signal).

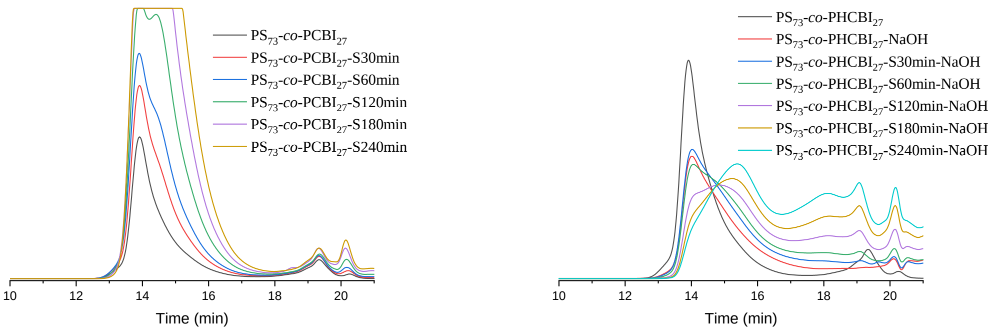

Fig. S68 SEC traces of PS$_{73}$-*co*-PHCBI$_{27}$ after sonication (left) and sonication-hydrolysis (right) (UV signal).

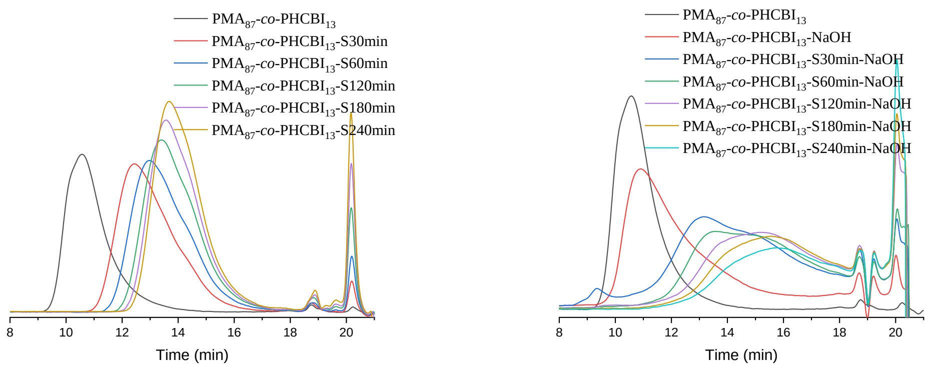

Fig. S69 SEC traces of PMA$_{87}$-*co*-PHCBI$_{13}$ after sonication (left) and sonication-hydrolysis (right) (dRI signal).

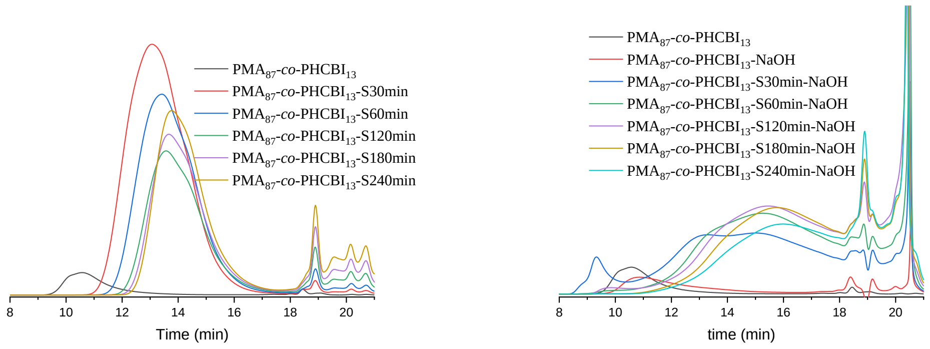

Fig. S70 SEC traces of PMA$_{87}$-*co*-PHCBI$_{13}$ after sonication (left) and sonication-hydrolysis (right) (UV signal).

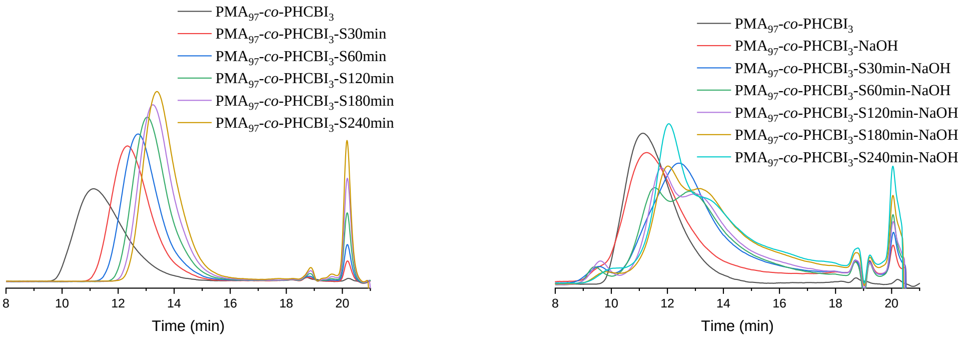

Fig. S71 SEC traces of PMA_{97}-*co*-$PHCBI_3$ after sonication (left) and sonication-hydrolysis (right) (dRI signal).

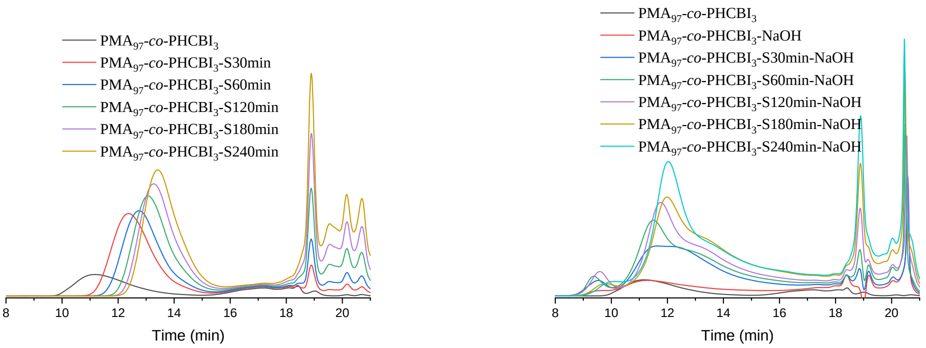

Fig. S72 SEC traces of PMA_{97}-*co*-$PHCBI_3$ after sonication (left) and sonication-hydrolysis (right) (UV signal).

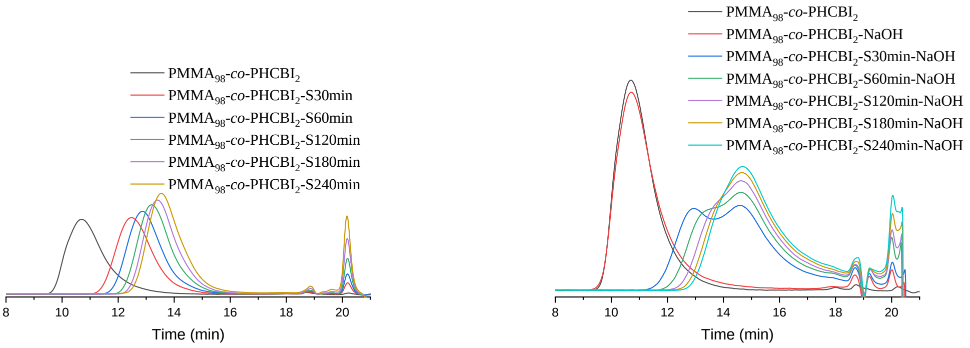

Fig. S73 SEC traces of $PMMA_{98}$-*co*-$PHCBI_2$ after sonication (left) and sonication-hydrolysis (right) (dRI signal).

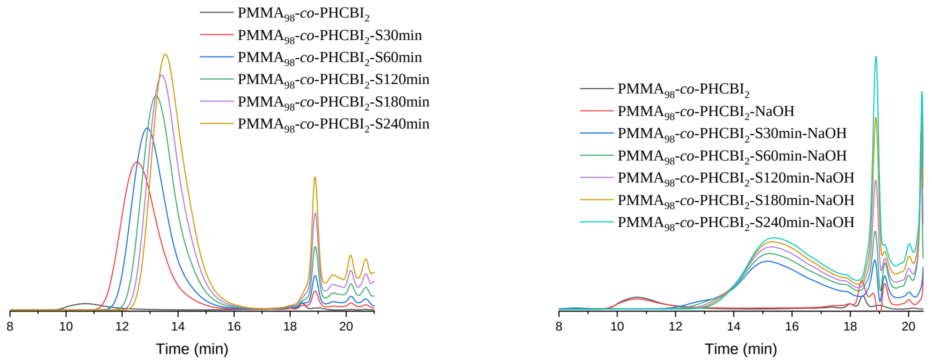

Fig. S74 SEC traces of PMMA$_{98}$-*co*-PHCBI$_2$ after sonication (left) and sonication-hydrolysis (right) (UV signal).

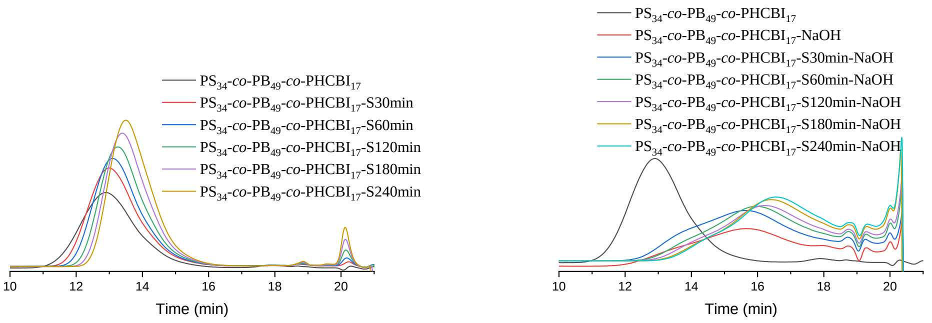

Fig. S75 SEC traces of PS$_{34}$-*co*-PB$_{49}$-*co*-PHCBI$_{17}$ after sonication (left) and sonication-hydrolysis (right) (dRI signal).

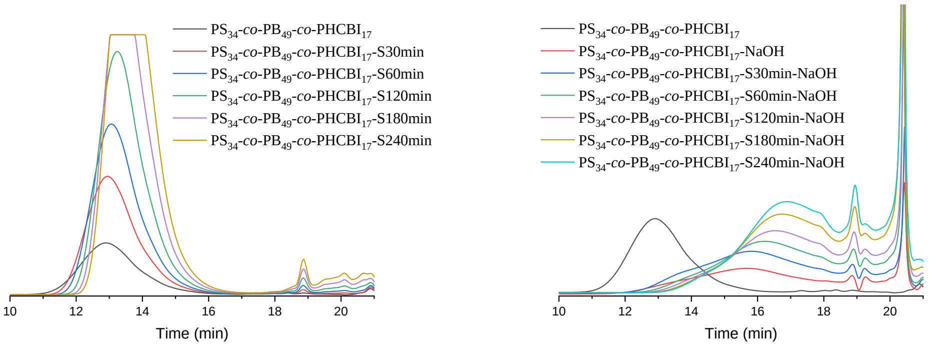

Fig. S76 SEC traces of PS$_{34}$-*co*-PB$_{49}$-*co*-PHCBI$_{17}$ after sonication (left) and sonication-hydrolysis (right) (UV signal).

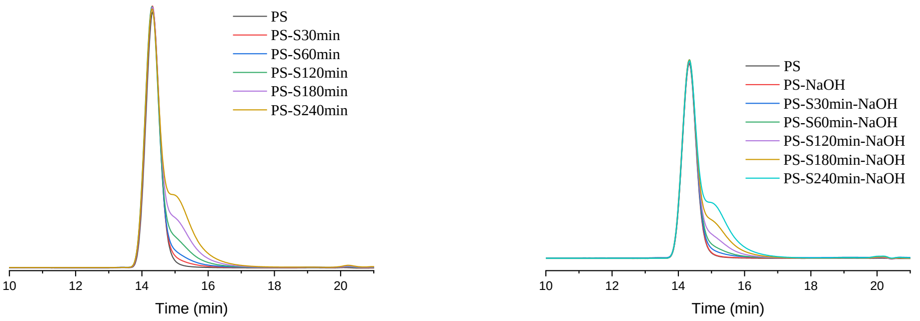

Fig. S77 SEC traces of PS (commercially available polystyrene, 50 kg mol^{-1}) after sonication (left) and sonication-hydrolysis (right) (dRI signal).

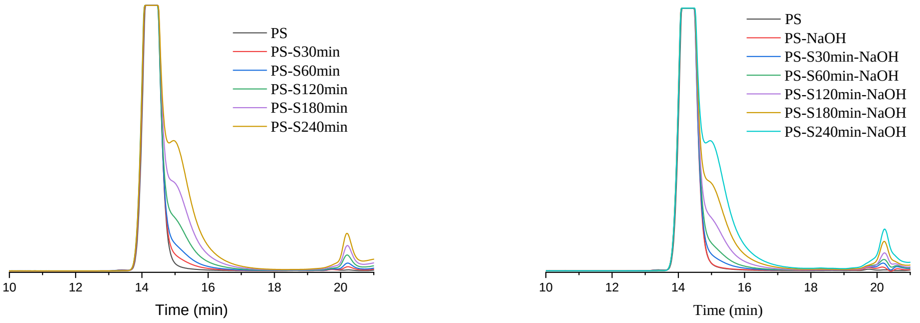

Fig. S78 SEC traces of PS (commercially available polystyrene, 50 kg mol^{-1}) after sonication (left) and sonication-hydrolysis (right) (UV signal).

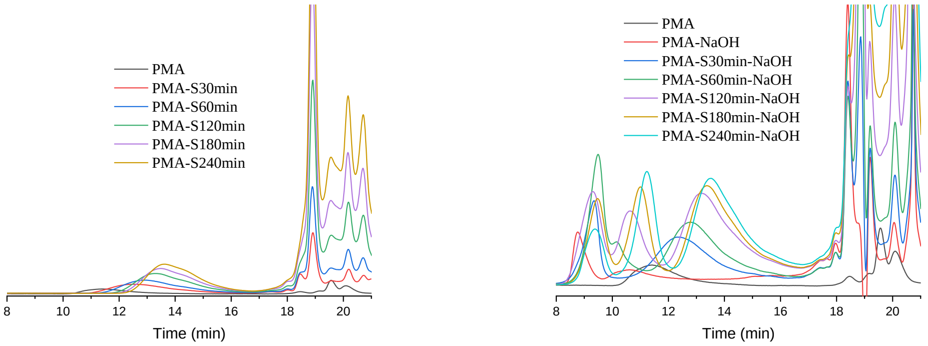

Fig. S79 SEC traces of PMA after sonication (left) and sonication-hydrolysis (right) (UV signal).

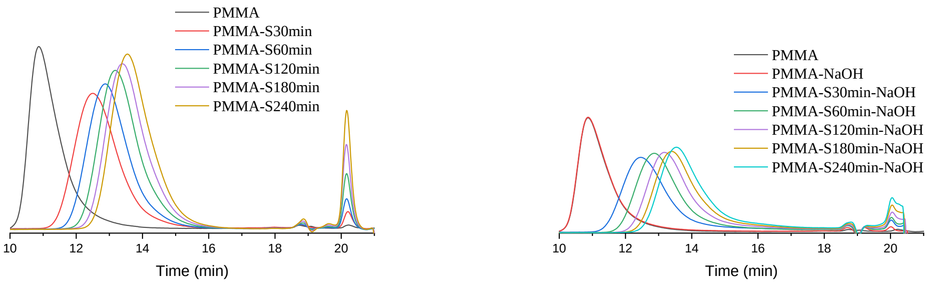

Fig. S80 SEC traces of PMMA after sonication (left) and sonication-hydrolysis (right) (dRI signal).

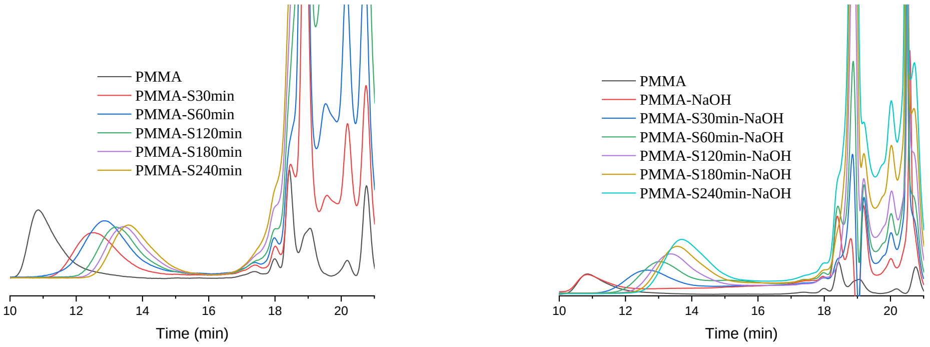

Fig. S81 SEC traces of PMMA after sonication (left) and sonication-hydrolysis (right) (UV signal).

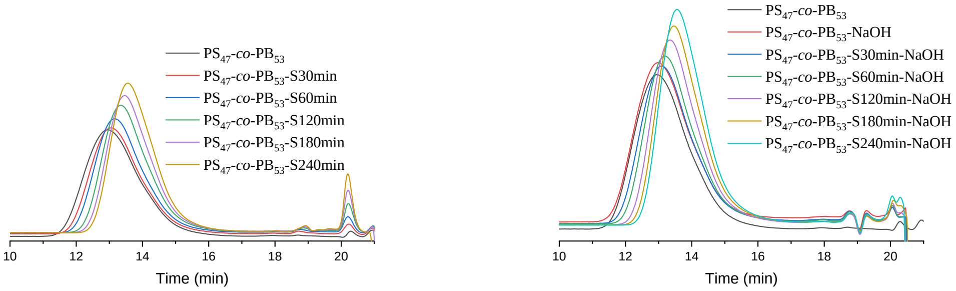

Fig. S82 SEC traces of PS_{47}-*co*-PB_{53} after sonication (left) and sonication-hydrolysis (right) (dRI signal).

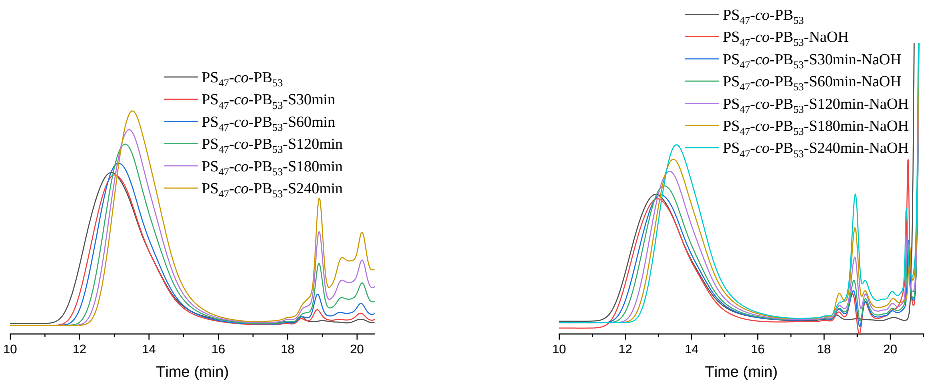

Fig. S83 SEC traces of PS$_{47}$-*co*-PB$_{53}$ after sonication (left) and sonication-hydrolysis (right) (UV signal).

Treatment of polymers with NaOH solution followed by ultrasonication

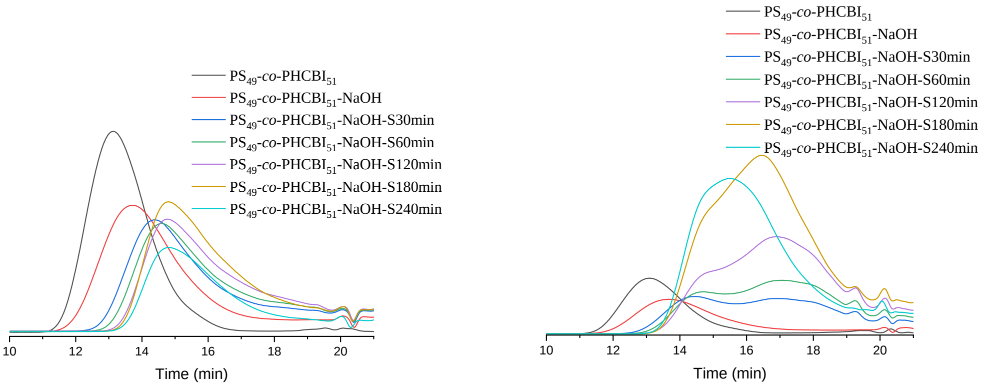

Fig. S84 SEC traces of PS$_{49}$-*co*-PHCBI$_{51}$ after treatment with NaOH, followed by ultrasonication (left: dRI signal; right: UV signal).

Cryo-milling of polymers followed by treatment with NaOH solution

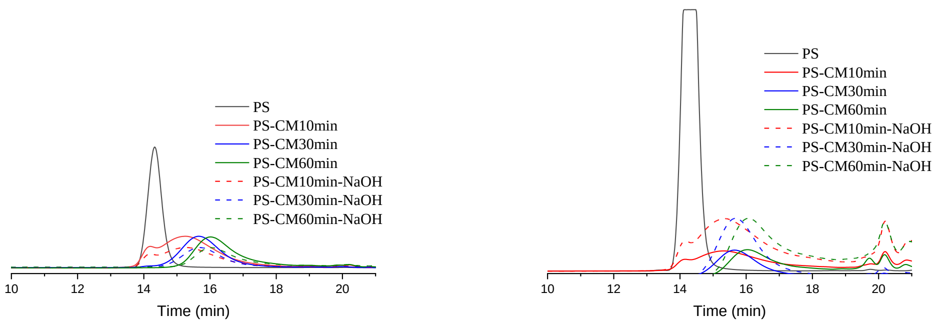

Fig. S85 SEC traces of PS after cryo-milling and cryo-milling-hydrolysis (left: dRI signal; right: UV signal).

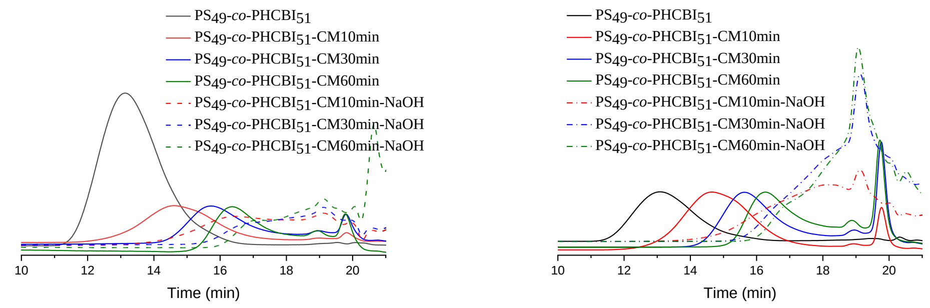

Fig. S86 SEC traces of PS_{49}-*co*-$PHCBI_{51}$ after cryo-milling and cryo-milling-hydrolysis (left: dRI signal; right: UV signal).

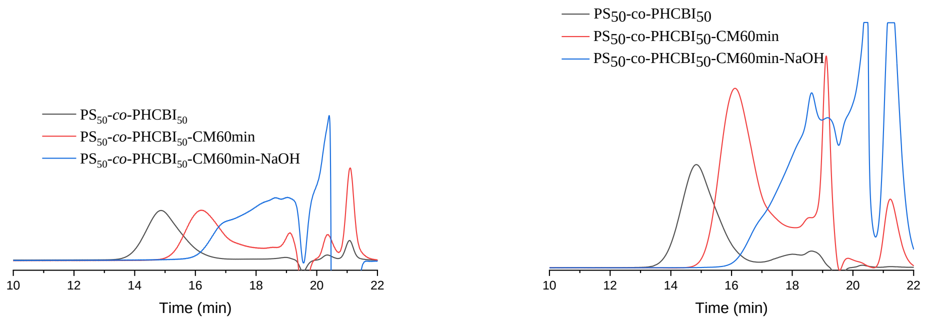

Fig. S87 SEC traces of PS_{50}-*co*-$PHCBI_{50}$ after cryo-milling and cryo-milling-hydrolysis (left: dRI signal; right: UV signal).

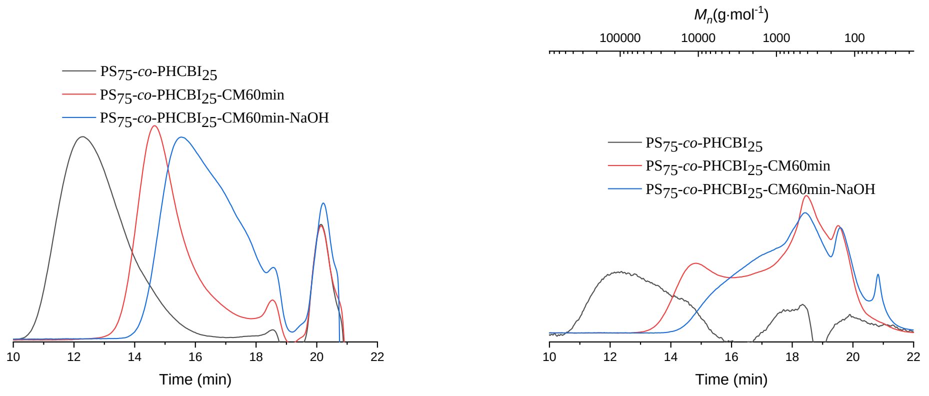

Fig. S88 SEC traces of PS_{75}-*co*-$PHCBI_{25}$ after cryo-milling and cryo-milling-hydrolysis (left: dRI signal; right: UV signal).

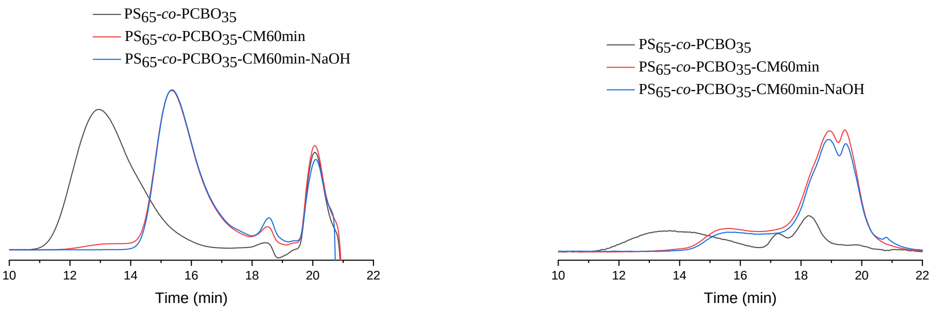

Fig. S89 SEC traces of PS_{65}-*co*-$PCBO_{35}$ after cryo-milling and cryo-milling-hydrolysis (left: dRI signal; right: UV signal).

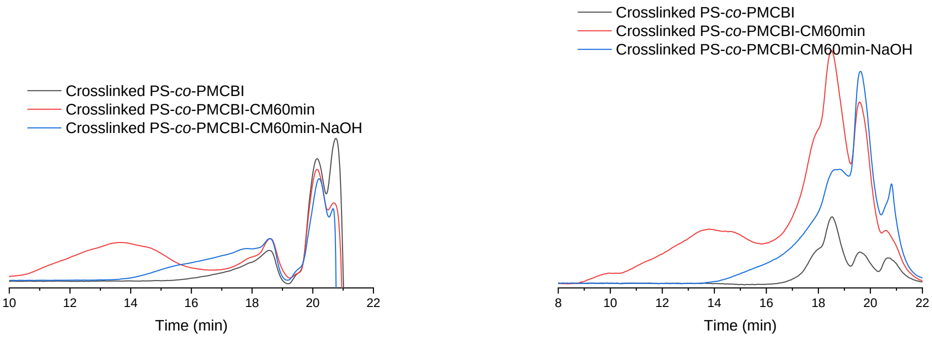

Fig. S90 SEC traces of (PS-*co*-PMCBI)-*l*-PDCBI, i.e. cross-linked PS-*co*-PMCBI, after cryo-milling and cryo-milling-hydrolysis (left: dRI signal; right: UV signal). The SEC trace (black) of cross-linked PS-*co*-PHCBI originates from the extractable sol fraction of the material (i.e. residual monomers and polymer chains that were not linked into the polymer network). After cryo-milling, the copolymer was slightly soluble in THF (red trace). After the hydrolysis step, the polymer was soluble in THF (blue trace).

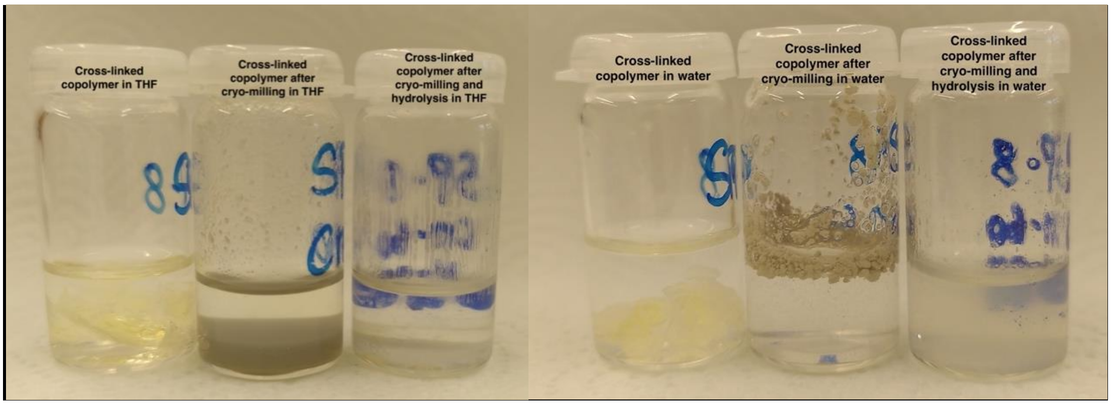

Fig. S91 Photographs of (PS-*co*-PMCBI)-*l*-PDCBI (i.e. cross-linked PS-*co*-PMCBI), (PS-*co*-PMCBI)-*l*-PDCBI after cryo-milling and (PS-*co*-PMCBI)-*l*-PDCBI after cryo-milling-hydrolysis (left in THF, right in water). The cross-linked copolymer was neither soluble in THF nor in water. After cryo-milling, the copolymer was still not soluble in THF and water (The grey color of the copolymers is from the stainless

steel end-caps and grinding cylinder of the cyro-mill). After cryo-milling and hydrolysis the copolymer was well-soluble in THF and partly soluble in water.

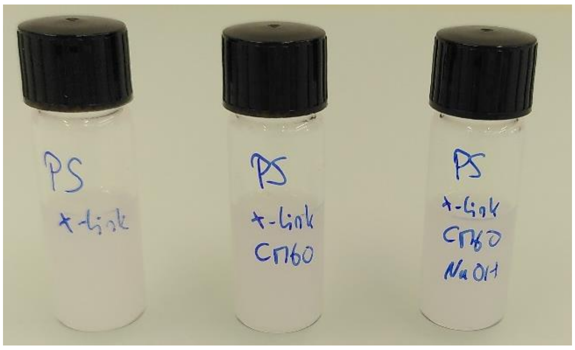

Fig. S92 Photographs of cross-linked PS, cross-linked PS after cryo-milling and cross-linked PS after cryo-milling-hydrolysis in THF. The cross-linked PS was not soluble in THF and did not become soluble in THF after cryo-milling and hydrolysis.

Treatment of polymers with different mechanical methods and hydrolysis

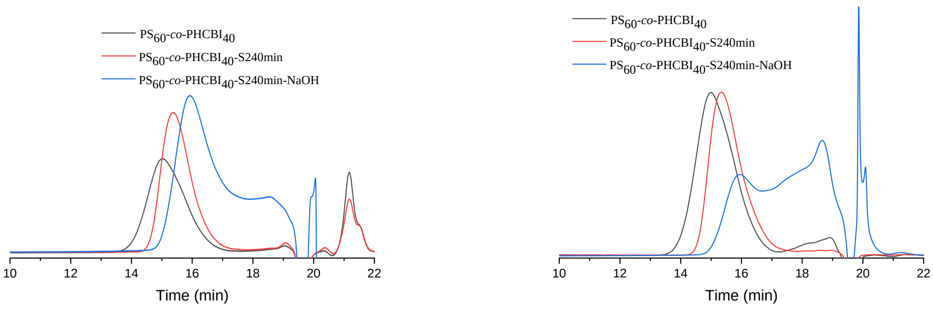

Fig. S93 SEC traces of PS_{60}-*co*-$PHCBI_{40}$ after sonication-hydrolysis (left: dRI signal; right: UV signal).

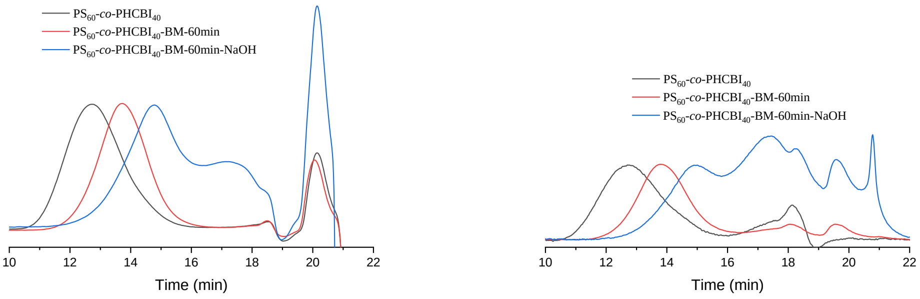

Fig. S94 SEC traces of PS_{60}-*co*-$PHCBI_{40}$ after ball-milling-hydrolysis (left: dRI signal; right: UV signal).

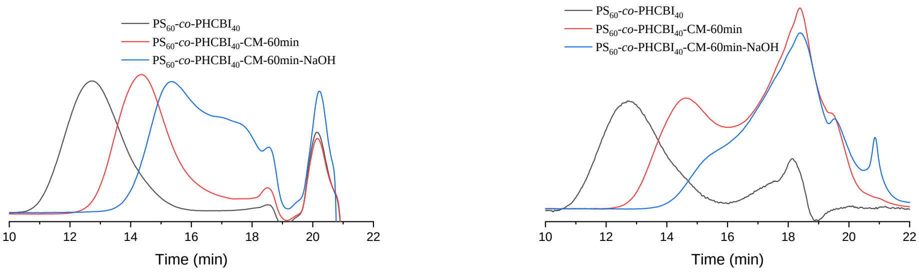

Fig. S95 SEC traces of PS_{60}-*co*-$PHCBI_{40}$ after cryo-milling-hydrolysis (left: dRI signal; right: UV signal).

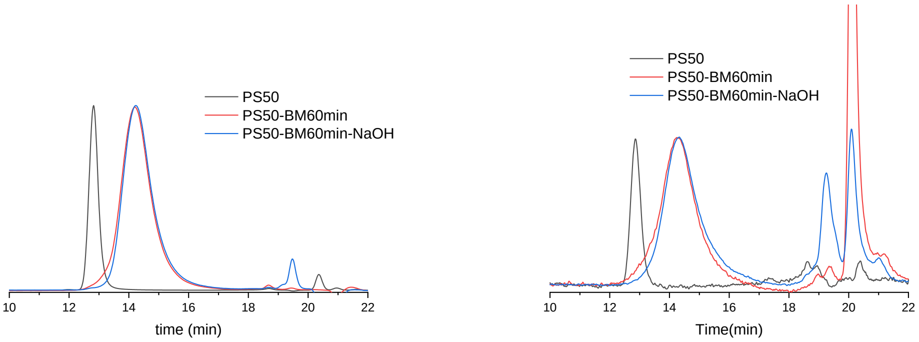

Fig. S96 SEC traces of PS after ball-milling-hydrolysis (left: dRI signal; right: UV signal).

Ball-milling of dry polymers with solid NaOH

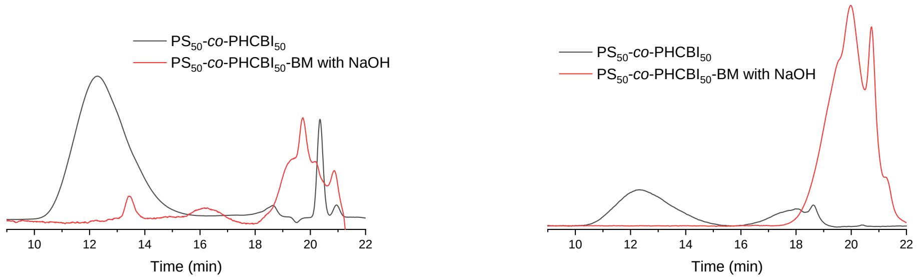

Fig. S97 SEC traces of PS_{50}-*co*-$PHCBI_{50}$ after dry ball-milling (60 min) in the presence of solid NaOH (left: dRI signal; right: UV signal).

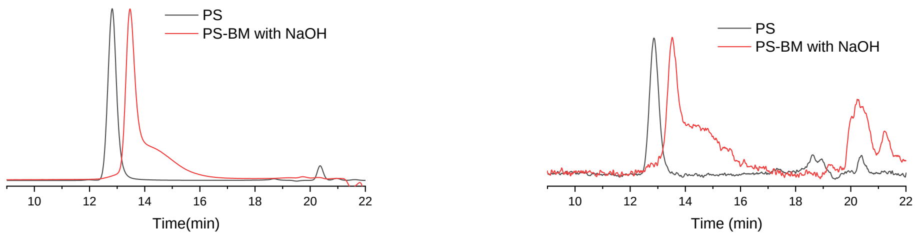

Fig. S98 SEC traces of PS after dry ball-milling (60 min) in the presence of solid NaOH (left: dRI signal; right: UV signal).

Ultrasonication of polymer followed by treatment with NaOH solutions of different concentrations

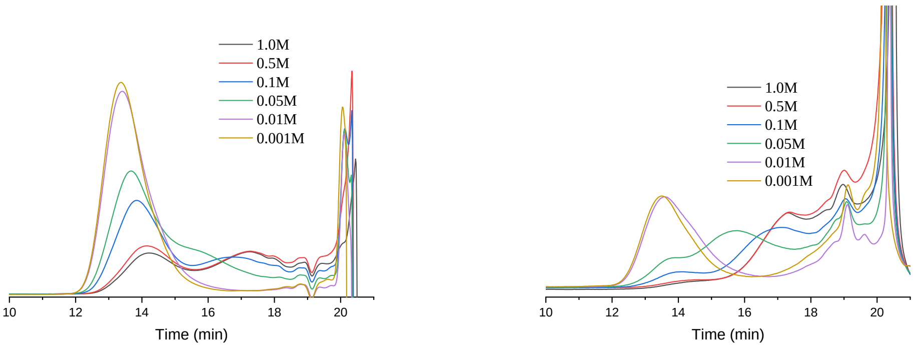

Fig. S99 SEC traces of PS_{49}-*co*-$PHCBI_{51}$ after sonication for 240 min and hydrolysis with NaOH solutions of different concentrations (left dRI signal, right UV signal). The hydrolysis efficiency increased with increasing NaOH concentration.

Chromatographic isolation and characterization of degradation products of PS_{50}-*co*-$PHCBI_{50}$

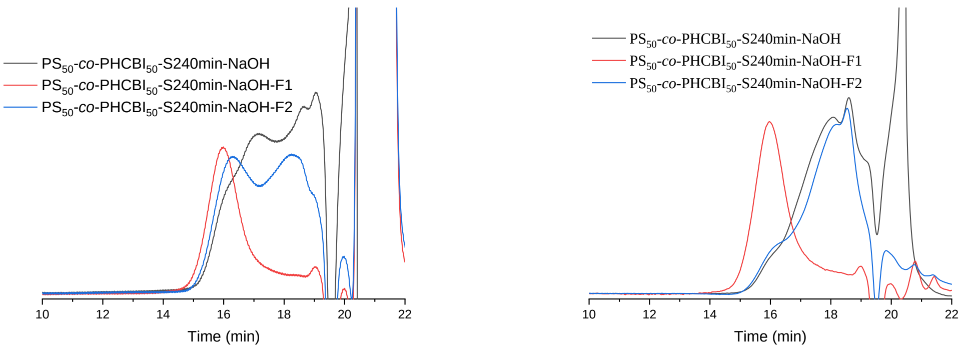

Fig. S100 SEC traces of PS_{50}-*co*-$PHCBI_{50}$ after sonication for 240 min and hydrolysis with NaOH, and of fractions obtained by flash silica chromatography (left dRI signal, right UV signal).

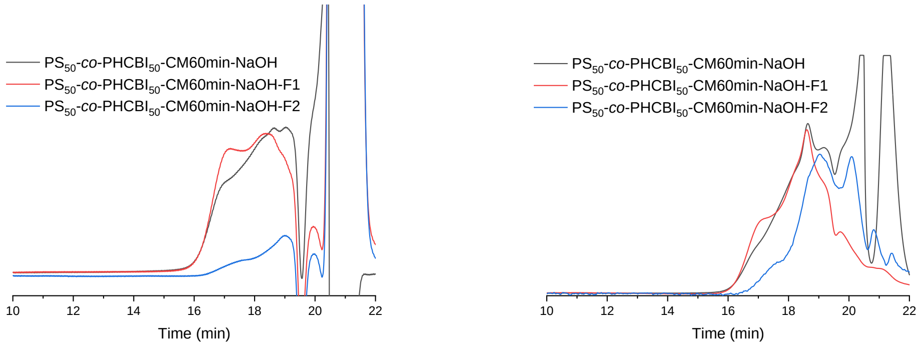

Fig. S101 SEC traces of PS_{50}-*co*-$PHCBI_{50}$ after cryo-milling and hydrolysis with NaOH, and of fractions obtained by flash silica chromatography (left dRI signal, right UV signal).

Characterization of the degradation products

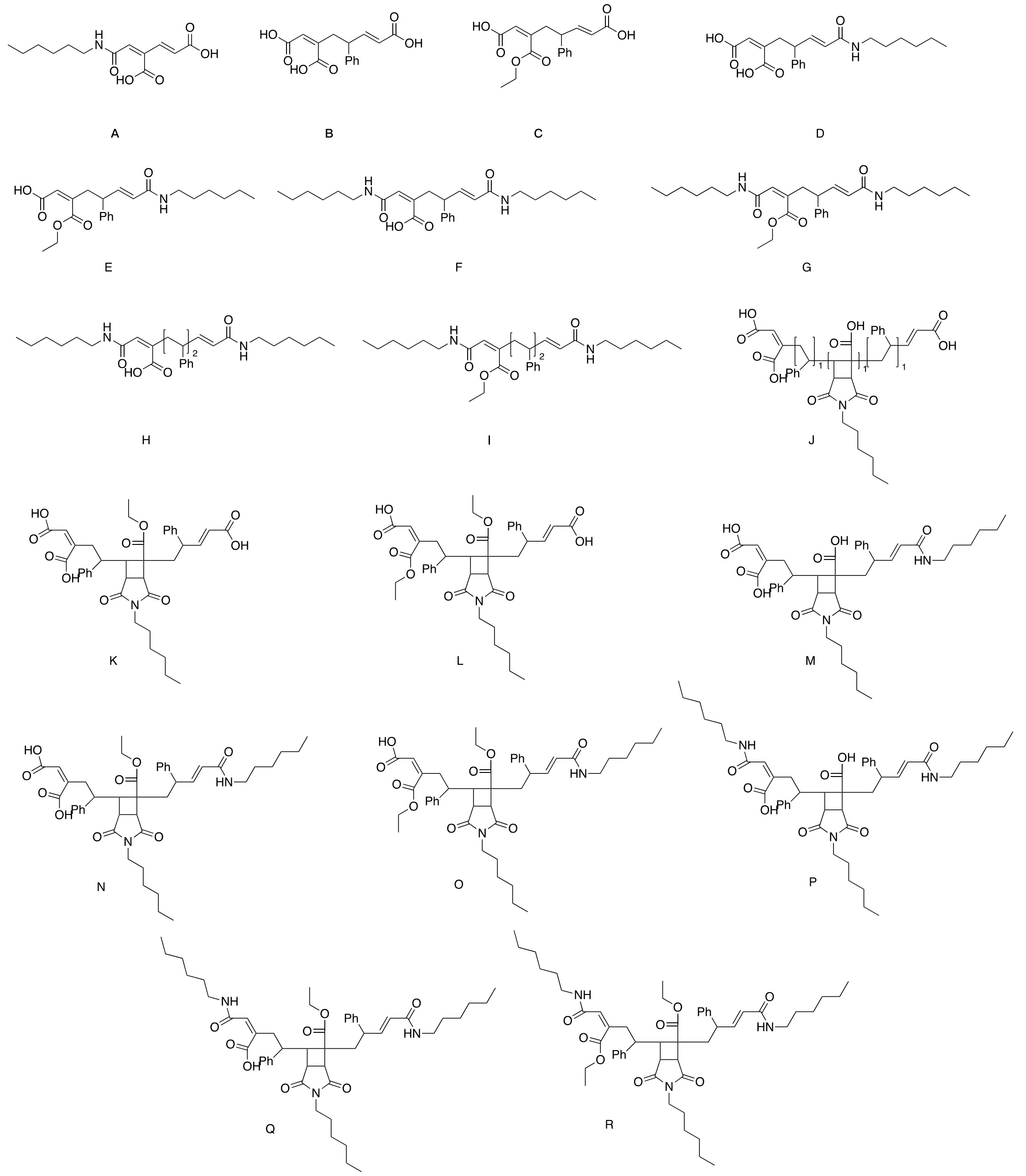

Fig. S102 List of expected degradation products.

LC-MS:

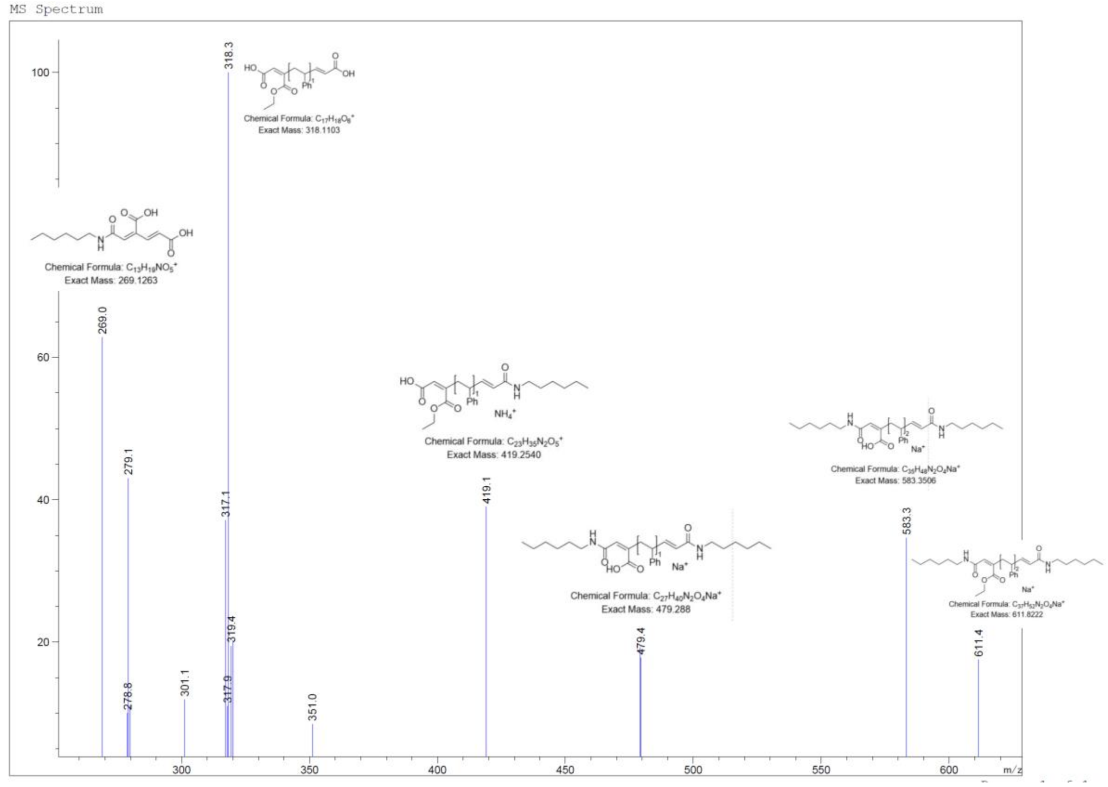

Fig. S103 ESI-MS analysis of PS_{50}-*co*-$PHCBI_{50}$ after sonication (240 min) and hydrolysis with NaOH solution overnight at room temperature (This figure was also showed as Fig. 5f in manuscript).

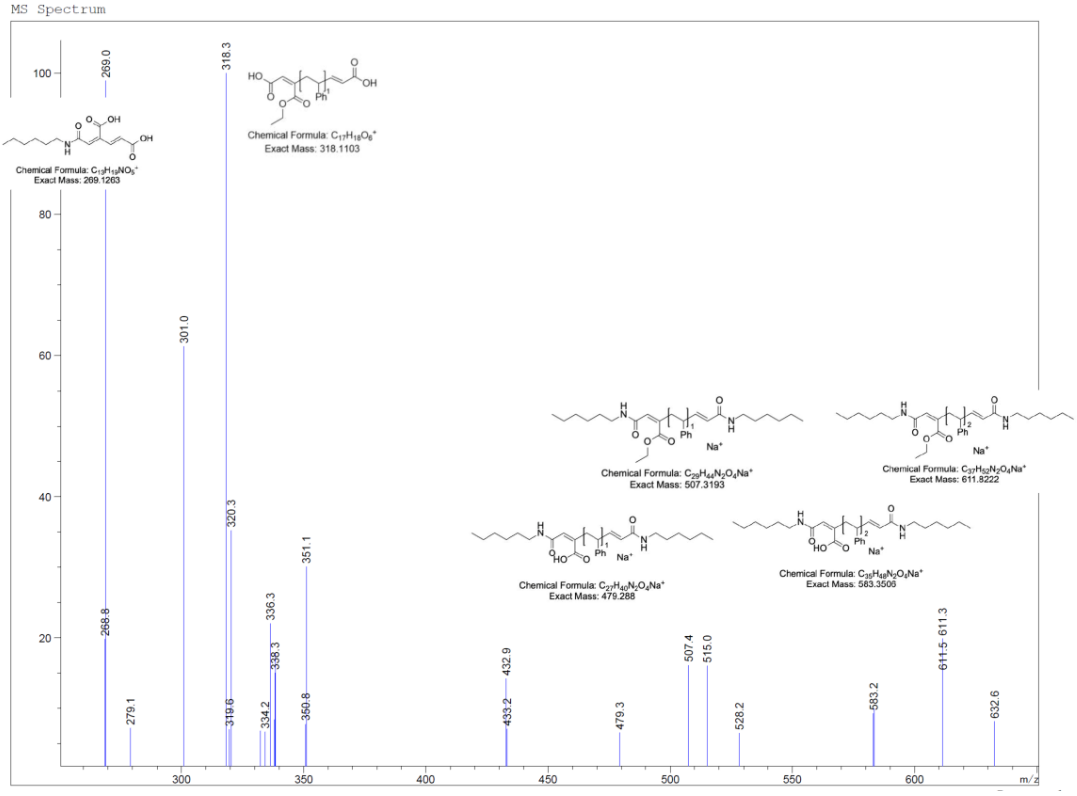

Fig. S104 ESI-MS of PS_{50}-*co*-$PHCBI_{50}$ after cryo-milling (60 min) and hydrolysis with NaOH solution overnight at room temperature.

UPLC-QToF:

The waters_connect software platform with UNIFI™ was used to process the MS data. Fig. S105 and Fig. S106 show the base peak intensity chromatograms (BPI) in ESI^+ and ESI^- mode for the Solvent, Blank Matrix, PS_{49}-*co*-$PHCBI_{51}$, PS_{49}-*co*-$PHCBI_{51}$-cryo-milling-hydrolysis and PS_{49}-*co*-$PHCBI_{51}$-sonication-hydrolysis. The BPI full scan chromatograms give an indication of the abundance of compounds detected in the samples. It is obvious that the blank matrix sample already shows numerous peaks. Nevertheless, some potential unique peaks and differences between the samples can already be observed by visual inspection, such as peaks at retention time at 8.14 and 9.55 min in ESI^+ mode (Fig. S105) and at retention time at 4.12 and 7.94 min in ESI^- mode (Fig. S106). A precise evaluation of eluting *m/z*-RT pairs with their corresponding intensities was carried out with the UNIFI™ software. Data was processed in two steps: 1) a three-dimensional (3D) peak detection, which is based on the calculation of peak volumes the detection of all ion clusters in a given retention time range, and 2) a screening step, where the software matches the retention time, *m/z* (±5 ppm) and intensity pairs (3D peak picking) of detected peaks (features) automatically against library containing 18 target compounds. For most compounds several isomeric species were observed in the accurate extracted ion chromatogram (XIC). Initially, only the up to 4 most intense chromatographic peaks were evaluated. A total of 17 target compounds were identified using this approach from data acquired in either ESI^+ or ESI^- mode. The detailed MS information of these compounds is summarized in Table S2 for sample PS_{49}-*co*-$PHCBI_{51}$-cryo-milling-hydrolysis and in Table S3 for sample PS_{49}-*co*-$PHCBI_{51}$-sonication-hydrolysis. The compounds listed as identified in the Table S2 and S3 can be confirmed with high confidence based on mass accuracy (±5 ppm) and the fit of the experimental isotope data to the theoretical isotope distribution (i-FIT). For all identified compounds, the isotope fit criteria defined as the deviation of the isotope match intensity in percent was less than 25 %. The mass accuracy was calculated as the mean value (ppm) of the five replicated measurements. Additional target compounds not identified in Table S2 and Table S3 could not be confirmed with sufficient level of confidence and were therefore not considered for evaluation or were not detectable. If two adducts are mentioned in the adduct column, both adducts could be detected. The adduct that is mentioned the first is the more abundant ion and was used for the confirmation purposes and accurate mass determination. All confirmed compounds in PS_{49}-*co*-$PHCBI_{51}$-cryo-milling-hydrolysis and PS_{49}-co-$PHCBI_{51}$-sonication-hydrolysis were not detected in the reference samples (solvent, blank matrix sample, non-degraded PS_{49}-*co*-$PHCBI_{51}$).

As an example, Compound I in ESI^+ mode is selected here. This compound has two isomers (at RT=7.95 min and RT=8.05 min). Fig. S101 shows a comparative XIC of this compound (*m/z* 589.400) for all five samples analyzed. While no signals can be observed in the solvent, blank matrix and PS_{49}-*co*-$PHCBI_{51}$ samples, two species of Compound I can be observed in PS_{49}-*co*-$PHCBI_{51}$-cryo-milling-hydrolysis and PS_{49}-*co*-$PHCBI_{51}$-sonication-hydrolysis. The corresponding low and high energy

(fragmented) mass spectra of the peaks at retention time 7.95 and 8.05 min are shown in Fig. S108 and S109 with their accurate molecular ion and product ion information, respectively. Compound I isomers were confirmed using the accurate mass information of the protonated precursor ions and compound structure (and elemental composition) was supported by the analysis of the fragment ions and their corresponding mass accuracies. The fragment match functionality of UNIFI™ was used to match the *m/z* values of theoretical sub-structures to measured fragment ions in the high energy spectrum function (Fig. S108, S109).

The chromatograms of the target compounds with *m/z* > 673 became very complex due to the presence of numerous isomeric species. Further investigation using fragment ion data is necessary to elucidate the chemical structure of these compounds. The intensity distribution of the observed isomeric species was partly different depending on the sample PS_{49}-*co*-$PHCBI_{51}$-cryo-milling-hydrolysis or PS_{49}-*co*-$PHCBI_{51}$-sonication-hydrolysis. Some isomeric species could only be partially detected in one or the other sample (e.g. compound 373 or 811). The exact elucidation of the structure is subject of further evaluations.

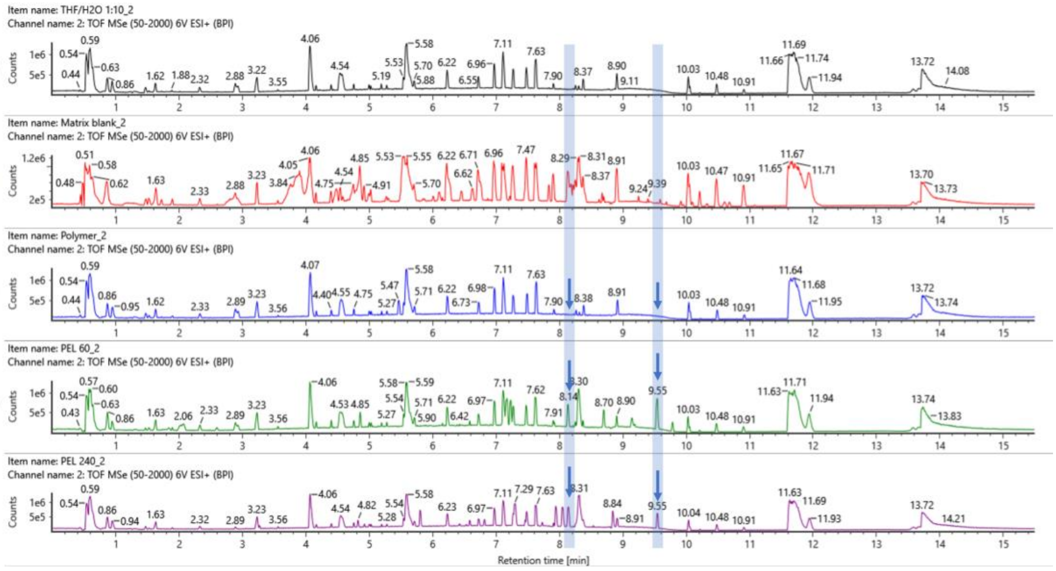

Fig. S105 BPI Chromatograms ESI^{+} mode (a) Solvent (b) Blank Matrix (c) PS_{49}-*co*-$PHCBI_{51}$ (d) PS_{49}-*co*-$PHCBI_{51}$-cryo-milling-hydrolysis (e) PS_{49}-*co*-$PHCBI_{51}$-sonication-hydrolysis. Blue bars with blue arrows indicate a selection of potential target peaks and also possible differences between the samples. Item name THF/H_2O 1:10_2 corresponds to sample solvent; Item name Matrix blank_2 corresponds to blank matrix sample; Item name Polymer_2 corresponds to PS_{49}-*co*-$PHCBI_{51}$; Item name PEL 60_2 corresponds to PS_{49}-*co*-$PHCBI_{51}$-cryo-milling-hydrolysis; Item name PEL 240_2 corresponds to PS_{49}-*co*-$PHCBI_{51}$-sonication-hydrolysis.

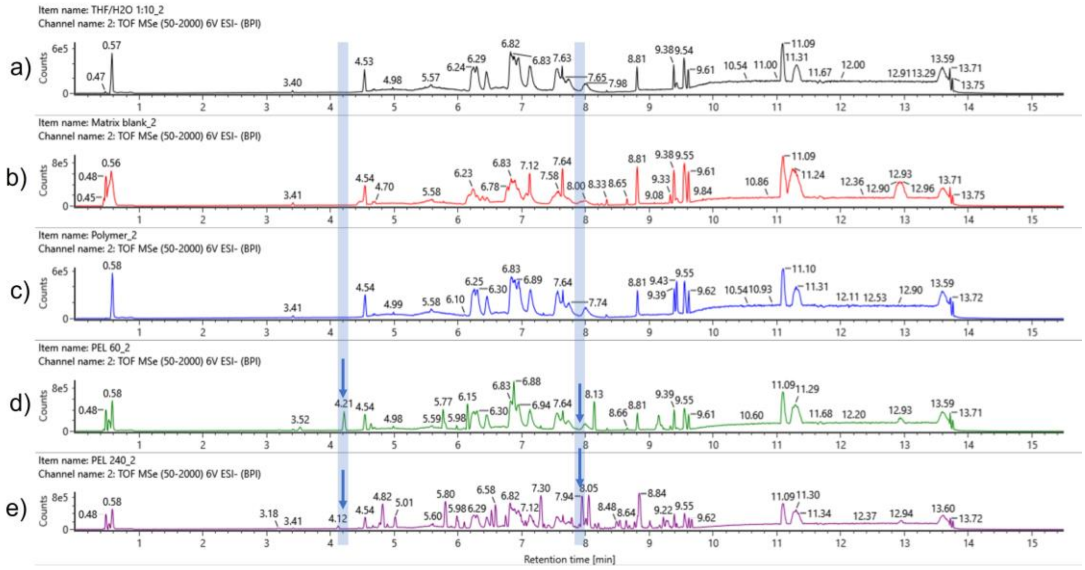

Fig. S106 BPI Chromatograms ESI$^-$ mode (a) Solvent (b) Blank Matrix (c) PS_{49}-*co*-$PHCBI_{51}$ (d) PS_{49}-*co*-$PHCBI_{51}$-cryomilling-hydrolysis (e) PS_{49}-*co*-$PHCBI_{51}$-sonication-hydrolysis. Blue bars with blue arrows indicate a selection of potential target peaks and also possible differences between the samples. Item name THF/H_2O 1:10_2 corresponds to sample solvent; Item name Matrix blank_2 corresponds to blank matrix sample; Item name Polymer_2 corresponds to PS_{49}-*co*-$PHCBI_{51}$; Item name PEL 60_2 corresponds to PS_{49}-*co*-$PHCBI_{51}$-cryo-milling-hydrolysis; Item name PEL 240_2 corresponds to PS_{49}-*co*-$PHCBI_{51}$-sonication-hydrolysis.

Table S2 Identification of expected targets in PS_{49}-*co*-$PHCBI_{51}$-cryo-milling-hydrolysis, ESI^- and ESI^+ mode. (Compound names correspond to the letter code shown in Fig. S102.)

Compound name	Formula	t_R (min)	Adduct	Theoretical mass (*m/z*)	Experimental mass (*m/z*)	Mass accuracy (ppm)
A	$C_{13}H_{19}NO_5$	3.56	$[M-H]^-$	268.1191	268.1189	-0.8
B	$C_{15}H_{14}O_6$	2.66	$[M-H]^-$	289.0718	289.0713	-1.7
C	$C_{17}H_{18}O_6$	4.19	$[M-H]^-$	317.1031	317.1029	-0.6
D	$C_{21}H_{27}NO_5$	4.60	$[M-H]^-$	372.1817	372.1816	-0.2
		4.60	$[M+H]^+$	374.1962	374.1962	±0.0
E	$C_{23}H_{31}NO_5$	5.80	$[M-H]^-$	400.2130	400.2129	-0.2
		5.80	$[M+H]^+$, $[M+Na]^+$	402.2275	402.2270	-1.2
E	$C_{23}H_{31}NO_5$	5.99	$[M-H]^-$, $[M-H+HCOO]^-$	400.2130	400.2125	-1.2
		5.99	$[M+H]^+$, $[M+Na]^+$	424.2094	402.2271	-0.9
G	$C_{29}H_{44}N_2O_4$	7.30	$[M-H+HCOO]^-$	529.3283	529.3284	+0.1
		7.30	$[M+H]^+$, $[M+Na]^+$	485.3374	485.3379	+1.1
I	$C_{37}H_{52}N_2O_4$	7.94	$[M-H+HCOO]^-$	633.3909	633.3904	-0.8
		7.94	$[M+H]^+$, $[M+Na]^+$	589.4000	589.3997	-0.4
I	$C_{37}H_{52}N_2O_4$	8.05	$[M-H+HCOO]^-$	633.3909	633.3900	-1.4
		8.05	$[M+H]^+$, $[M+Na]^+$	589.4000	589.3986	-2.3
K	$C_{38}H_{43}NO_{10}$	6.00	$[M-H]^-$	672.2814	672.2805	-1.3
L	$C_{40}H_{47}NO_{10}$	6.86	$[M-H]^-$	700.3127	700.3120	-1.1
		6.86	$[M+H]^+$, $[M+Na]^+$	702.3273	702.3257	-2.2
L	$C_{40}H_{47}NO_{10}$	7.01	$[M-H]^-$	700.3127	700.3117	-1.4
N	$C_{44}H_{56}N_2O_9$	7.18	$[M-H]^-$	755.3913	755.3893	-2.7
		7.18	$[M+H]^+$, $[M+Na]^+$	757.4059	757.4044	-2.0
N	$C_{44}H_{56}N_2O_9$	7.38	$[M-H]^-$	755.3913	755.3893	-2.7
		7.38	$[M+H]^+$, $[M+Na]^+$	757.4059	757.4034	-3.3
O	$C_{46}H_{60}N_2O_9$	8.01	$[M+H]^+$	785.4372	785.4348	-3.1
O	$C_{46}H_{60}N_2O_9$	8.17	$[M-H]^-$	783.4226	783.4205	-2.6
		8.17	$[M+H]^+$, $[M+Na]^+$	785.4372	785.4351	-2.7
P	$C_{48}H_{65}N_3O_8$	8.85	$[M+H]^+$	812.4844	812.4828	-2.0
P	$C_{48}H_{65}N_3O_8$	9.08	$[M+H]^+$	812.4844	812.4830	-1.7
Q	$C_{50}H_{69}N_3O_8$	8.53	$[M+Na]^+$, $[M+H]^+$	862.4977	862.4958	-2.2
R	$C_{52}H_{73}N_3O_8$	9.14	$[M-H+HCOO]^-$	912.5380	912.5378	-0.2
		9.14	$[M+H]^+$	868.5470	868.5474	+0.4

Table S3 Identification of expected targets in PS_{49}-*co*-$PHCBI_{51}$-sonication-hydrolysis, ESI^- and ESI^+ mode. (Compound names correspond to the letter code shown in Fig. S102.)

Compound name	Formula	t_R (min)	Adduct	Theoretical mass (*m/z*)	Experimental mass (*m/z*)	Mass accuracy (ppm)
A	$C_{13}H_{19}NO_5$	3.56	$[M-H]^-$	268.1191	268.1187	-1.8
B	$C_{15}H_{14}O_6$	2.66	$[M-H]^-$	289.0718	289.0715	-0.2
D	$C_{21}H_{27}NO_5$	4.82	$[M-H]^-$, $[M-H+HCOO]^-$	372.1817	372.1815	-0.4
		4.82	$[M+H]^+$, $[M+Na]^+$	374.1962	374.1968	+1.2
E	$C_{23}H_{31}NO_5$	5.80	$[M-H]^-$	400.2130	400.2131	+0.2
		5.80	$[M+H]^+$, $[M+Na]^+$	402.2275	402.2280	+1.2
E	$C_{23}H_{31}NO_5$	5.99	$[M-H]^-$, $[M-H+HCOO]^-$	400.2130	400.2129	-0.2
		5.99	$[M+Na]^+$, $[M+H]^+$	424.2094	424.2094	-1.6
F	$C_{27}H_{40}N_2O_4$	6.53	$[M-H]^-$, $[M-H+HCOO]^-$	455.2915	455.2917	+0.4
		6.53	$[M+H]^+$, $[M+Na]^+$	457.3061	457.3062	+0.2
G	$C_{29}H_{44}N_2O_4$	7.30	$[M-H]^-$, $[M-H+HCOO]^-$	529.3283	529.3284	+0.2
		7.30	$[M+H]^+$, $[M+Na]^+$	485.3374	485.2277	+0.6
H	$C_{35}H_{48}N_2O_4$	7.18	$[M-H]^-$	559.3541	559.3545	+0.6
		7.18	$[M+H]^+$, $[M+Na]^+$	561.3687	561.3681	-1.1
H	$C_{35}H_{48}N_2O_4$	7.35	$[M-H]^-$	559.3541	559.3537	-0.7
		7.35	$[M+H]^+$, $[M+Na]^+$	561.3687	561.3688	+0.2
I	$C_{37}H_{52}N_2O_4$	7.94	$[M-H+HCOO]^-$	633.3911	633.3912	+0.3
		7.94	$[M+H]^+$, $[M+Na]^+$	589.4000	589.4005	+0.9
I	$C_{37}H_{52}N_2O_4$	8.05	$[M-H+HCOO]^-$	633.3909	633.3912	+0.5
		8.05	$[M+H]^+$, $[M+Na]^+$	589.4000	589.4002	+0.4
K	$C_{38}H_{43}NO_{10}$	5.87	$[M+H]^+$, $[M+Na]^+$	674.2960	674.2948	-1.7
K	$C_{38}H_{43}NO_{10}$	6.00	$[M-H]^-$	672.2814	672.2801	-1.9
		6.00	$[M+H]^+$	674.2960	674.2955	-0.7
L	$C_{40}H_{47}NO_{10}$	6.86	$[M-H]^-$	700.3127	700.3122	-0.7
		6.86	$[M+H]^+$, $[M+Na]^+$	702.3273	702.3264	-1.2
L	$C_{40}H_{47}NO_{10}$	7.01	$[M-H]^-$	700.3127	700.3125	-0.3
		7.01	$[M+H]^+$, $[M+Na]^+$	702.3273	702.3268	-0.6
M	$C_{42}H_{52}N_2O_9$	6.61	$[M-H]^-$	727.3600	727.3594	-0.7
		6.61	$[M+H]^+$, $[M+Na]^+$	729.3746	729.3734	-1.6
M	$C_{42}H_{52}N_2O_9$	6.76	$[M-H]^-$	727.3600	727.3596	-0.6
		6.76	$[M+H]^+$, $[M+Na]^+$	729.3746	729.3736	-1.3
N	$C_{44}H_{56}N_2O_9$	7.18	$[M-H]^-$	755.3913	755.3910	-0.4
		7.18	$[M+H]^+$, $[M+Na]^+$	757.4059	757.4054	-0.6
N	$C_{44}H_{56}N_2O_9$	7.38	$[M-H]^-$	755.3913	755.3909	-0.6
		7.38	$[M+H]^+$, $[M+Na]^+$	757.4059	757.4046	-1.7
O	$C_{46}H_{60}N_2O_9$	7.90	$[M-H]^-$	783.4226	783.4227	+0.1
		7.90	$[M+H]^+$, $[M+Na]^+$	785.4372	785.4367	-0.6
O	$C_{46}H_{60}N_2O_9$	8.01	$[M-H]^-$	783.4226	783.4225	-0.1
		8.01	$[M+H]^+$, $[M+Na]^+$	785.4372	785.4370	-0.2
O	$C_{46}H_{60}N_2O_9$	8.17	$[M-H]^-$	783.4226	783.4230	+0.5
		8.17	$[M+H]^+$, $[M+Na]^+$	785.4372	785.4361	-1.4
P	$C_{48}H_{65}N_3O_8$	7.80	$[M-H]^-$	810.4699	810.4693	-0.8
		7.80	$[M+H]^+$	812.4844	812.4831	-1.6
P	$C_{48}H_{65}N_3O_8$	8.15	$[M-H]^-$	810.4699	810.4703	+0.5
		8.15	$[M+H]^+$, $[M+Na]^+$	812.4844	812.4823	-2.5
Q	$C_{50}H_{69}N_3O_8$	8.20	$[M-H]^-$	838.5012	838.5017	+0.6
		8.20	$[M+H]^+$	840.5157	840.5151	-0.8
Q	$C_{50}H_{69}N_3O_8$	8.53	$[M-H]^-$	838.5012	838.5013	+0.1
		8.53	$[M+H]^+$, $[M+Na]^+$	840.5157	840.5149	-0.9
R	$C_{52}H_{73}N_3O_8$	8.84	$[M-H+HCOO]^-$	912.5380	912.5378	-0.2
		8.84	$[M+Na]^+$	890.5290	890.5285	-0.6
R	$C_{52}H_{73}N_3O_8$	9.14	$[M+H]^+$, $[M+Na]^+$	868.5470	868.5486	+1.9

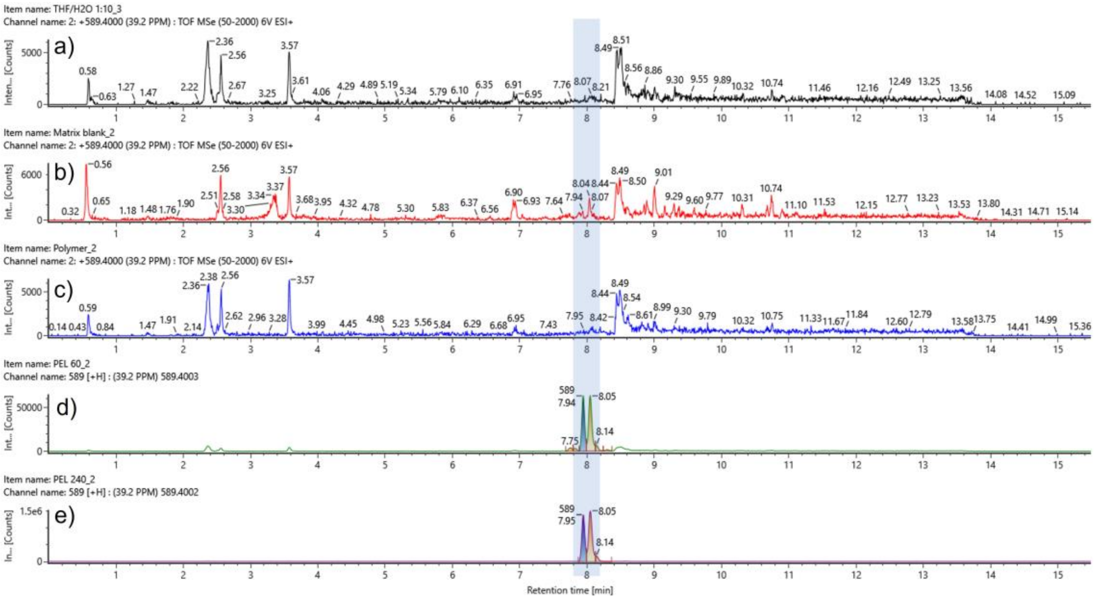

Fig. S107 XIC *m/z* 589.4000 of Compound I; ESI+ mode; (a) Solvent (b) Blank Matrix (c) PS_{49}-*co*-$PHCBI_{51}$; observed two isomers at RT=7.95 and RT=8.05 min in (d) PS_{49}-*co*-$PHCBI_{51}$-cryo-milling-hydrolysis and (e) PS_{49}-*co*-$PHCBI_{51}$-sonication-hydrolysis. The y-achsis (Intensity [counts]) is not linked. Item name THF/H_2O 1:10_3 corresponds to sample solvent; Item name Matrix blank_2 corresponds to blank matrix sample; Item name Polymer_2 corresponds to PS_{49}-*co*-$PHCBI_{51}$; Item name PEL 60_2 corresponds to PS_{49}-*co*-$PHCBI_{51}$-cryo-milling-hydrolysis; Item name PEL 240_2 corresponds to PS_{49}-*co*-$PHCBI_{51}$-sonication-hydrolysis.

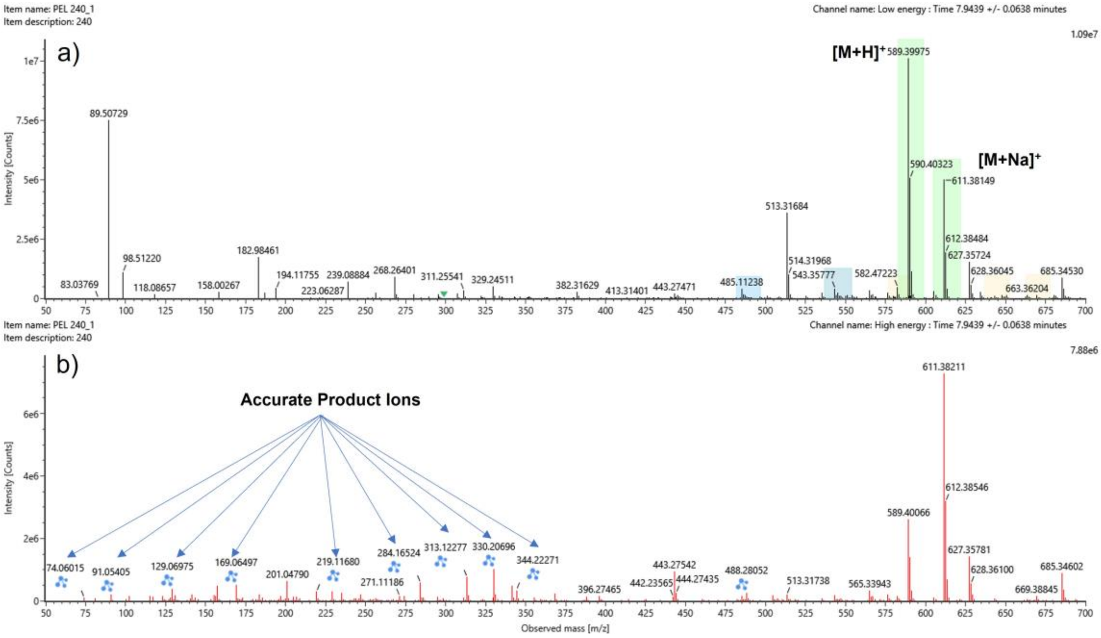

Fig. S108 (a) Low and (b) high energy mass spectra of Compound I at RT=7.95 min; PS_{49}-*co*-$PHCBI_{51}$-sonication-hydrolysis. Item name PEL 240_1 corresponds to PS_{49}-*co*-$PHCBI_{51}$-sonication-hydrolysis. Blue icons indicate matched product ions in the high-energy spectrum.

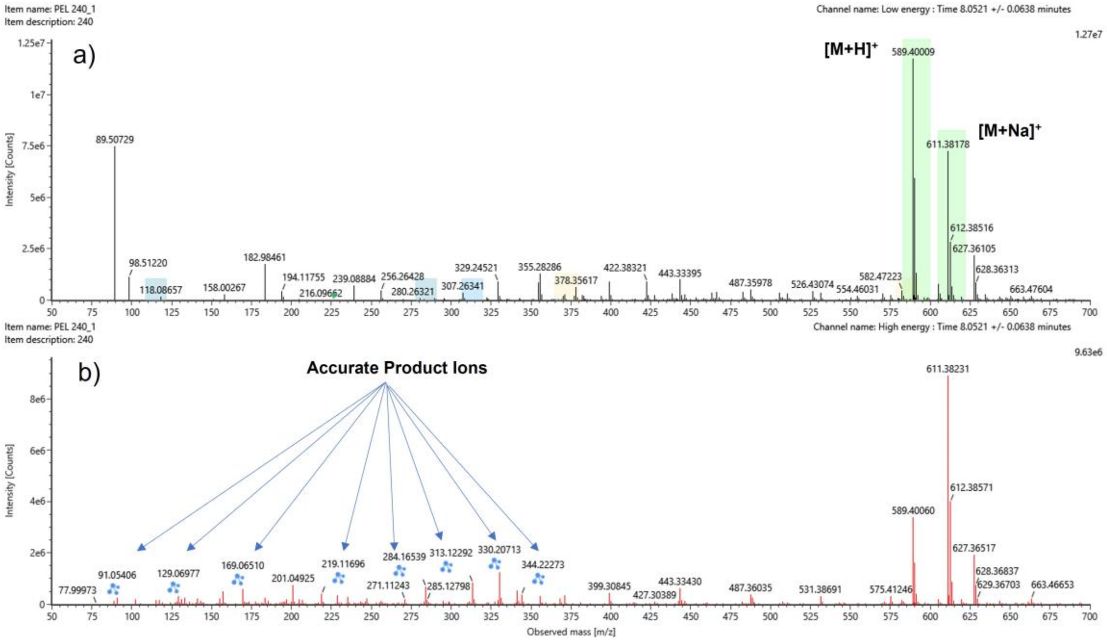

Fig. S109 (a) Low and (b) high energy mass spectra of Compound I at RT=8.05 min; PS_{49}-*co*-$PHCBI_{51}$-sonication-hydrolysis. Item name PEL 240_1 corresponds to PS_{49}-*co*-$PHCBI_{51}$-sonication-hydrolysis. Blue icons indicate matched product ions in the high-energy spectrum.

CoGEF Calculation

In order to shed some further light onto the degradation mechanism of PS-*co*-PCBI copolymers under influence of mechanical stress, a detailed computational study was carried out using the constrained geometries simulate external force (CoGEF) method[10-12] as well as unrestrained DFT calculationst similar to previous reports[13-17]. The CoGEF method yields bond ruptures (chemical transitions) under the influence of strain applied to the chain model. These can be different from "relaxed" TS that occur in unconstrained systems. However, these bond rupture points represent "transition states" (though strained ones) that formally meet the criteria for a TS, namely the first derivative of the energy with respect to the nuclear coordinates vanishes (becomes zero), and the energy along this intrinsic reaction coordinate (IRC) is a maximum. As TS in constrained and relaxed calculations can differ, the "true" and fully relaxed transition states (in the traditional sense of a TS) for the models under consideration were located by DFT calculations.

The CoGEF method was employed on models containing a central MCBI unit, flanked on each side by single styrene moieties, and terminated on both ends by methyl groups to be used as pulling positions. For computational efficiency, MCBI and not HCBI was applied in the *in-silico* models. As the individual degradation pathway may depend on the specific stereochemical arrangement of the chain models on both side of the MCBI unit, four different diastereomeric models ***cis*/*trans*-1/2** were analyzed (Fig. S110a). Here *cis*- and *trans*-descriptors refer to the relative arrangement of the styrene units (propagating chains) on the central cyclobutene ring, and the numbering *1* and *2* refers

to the orientation of the five-membered cycloimide ring. In each case, CoGEF analysis was started from fully density functional theory (DFT) optimized molecular geometries (B3LYP/6-31G(d)) with end-to-end separations d between $d \approx 4.5$ (*cis*) and $d \approx 5.5$ Å (*trans*).

The mechanical stress (pulling force) was simulated by constraining and gradually increasing the end-to-end distance between the terminal methyl groups in steps of 0.05 Å at the same level of theory (Fig. S110b), whilst optimizing all other internal coordinates at each point. With increasing stress, some low-barrier and low-force initial conformational changes between $d \approx 5$-8 Å led to extended molecular models. Notably, with increasing mechanical stress in particular the cyclobutane C-C-bond located in the polymer chain underwent significant elongation. Thus, it became apparent at an early stage of the analysis that diradical species had to be expected along the degradation pathway, and all simulations after the last conformational transitions ($d \approx 8.5$ Å) were continued on an unrestricted open-shell level of theory (UB3LYP/6-31G(d)). The DFT calculations with an unrestricted UB3LYP functional may yield heterolytic or homolytic bond ruptures, while a restricted (closed shell) RB3LYP DFT calculation can only yield heterolytic bond cleavage and will never locate radical or diradical transition states as it forces electrons always to be paired.

All models examined – with exception of ***trans*-2**, which underwent a C-C-chain break event and the loss of a styrene unit – displayed the formation of a diradical species through rupture of the cyclobutane ring upon increasing stress (transition state TS1 in Figs. S110 and S111). Note that the energies plotted in Fig. S110 do not visualize real "transitions states", but only show the gradually increasing electronic energy of the systems upon action of the pulling constraint. Bond breakage is characterized by a gradual and continuous increase of the corresponding C-C separation from an equilibrium bond length of ≈ 1.59 Å to ≈ 2.0 Å without a sharp energy step (Fig. S110b, grey shaded area) during formation of the singlet diradical species (the reverse process would be an essentially barrier-free diradical recombination process with a very early transition state, hence the missing distinct energy jumps).

Increasing the end-to-end pulling force even further absorbs energy by radical separation ($d_{C\text{-}C} > 3.0$ Å) and elongation of the second cyclobutane C-C-bond, the transition between both bond elongation regimes is marked by a characteristic kink in the energy plots (Fig. S110b, end of grey shaded area). As the effective force acting on the polymer models corresponds to the first derivative $\partial E/\partial d$ of the DFT self-consistent field (SCF) electronic energy E with respect to end-to-end distance d, Fig. 110c displays a maximum at the point where diradical formation along transition TS1 (Fig. S111) is observed.

Two different reaction pathways are conceivable in the further course of the chain degradation profile: ***cis*-1** and ***trans*-1** both undergo direct cleavage of the second C-C bond and ring opening along TS2 (Fig. S111), accompanied by formation of two double bonds. Molecular geometries before and after rearrangement, as well as inherent strain energies of the species involved are visualized in Fig. S112, and some characteristic

parameters are listed in Table S4. A “true” transition state TS2‡ for the final cyclobutane ring opening of ***cis*-1** is shown in Fig. S113. In contrast, ***cis*-2** displays a rearrangement and radical recombination via the carbonyl group of the ethyl ester, furnishing a cyclic ketene acetal derivative (TS3 in Fig. S111). This formal cyclobutane → pyran ring expansion releases strain, and in the case of ***cis*-2** was preferred due to a favorable orientation of the ester group. Though in principle this 3,4-dihydro-2*H*-pyran intermediate could be imagined to transform via a hetero retro-Diels Alder reaction (TS4 in Fig. S111) into the same cyclobutane degradation product as before, this was not observed here. Due to the unfavorable direction of the pulling vector (see Figs. S111 and S112) relative to a putative Diels-Alder transition state, a final C-C chain break (loss of a terminal methyl group) is observed in the case of ***cis*-2** at high energies and forces (Fig. S110b,c; rightmost points of the blue curves marked by an asterisk). A ring opening of the cyclobutane units through a concerted [2π+2π] cycloreversion process (TS5) was not observed in any case.

In summary, the CoGEF analysis of the PS$_x$-*co*-PMCBI$_y$ polymer models reveal that all rearrangements did proceed via initial diradical formation. Though in principle, conrotatory thermally allowed [2+2]-cycloreversions (TS5 in Fig. S111) or strain-induced disrotatory (thermally forbidden) ring openings are conceivable,[18-19] the corresponding processes were not observed here.

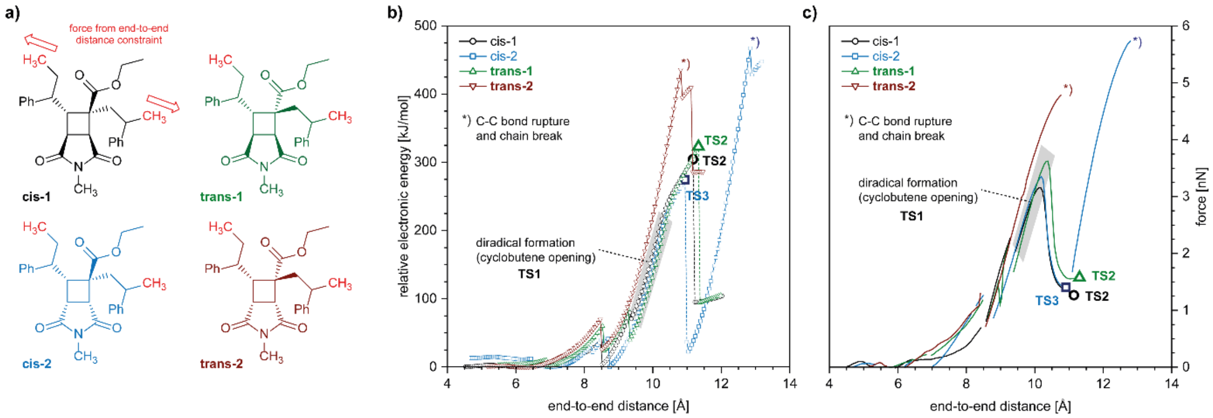

Fig. S110 (a) Different diastereomeric PS-*co*-PMCBI polymer models used for the CoGEF analysis of the degradation mechanism. (b) CoGEF energy profiles (DFT UB3LYP/6-31G(d)) as a function of the constrained end-to-end distances between terminal methyl groups. Characteristic reaction transitions (TS1-3) are marked by bold symbols, and characteristics of the corresponding molecular geometries are listed in Table S4; points marked by asterisks correspond to chain break events trough C-C bond rupture. (c) Plot of the effective forces originating from chain elongation constraints (first derivative $\partial E/\partial d$ of plot b); curve segments of negative forces (strain releasing conformational transitions and reaction events) are not plotted for clarity (gaps in curves).

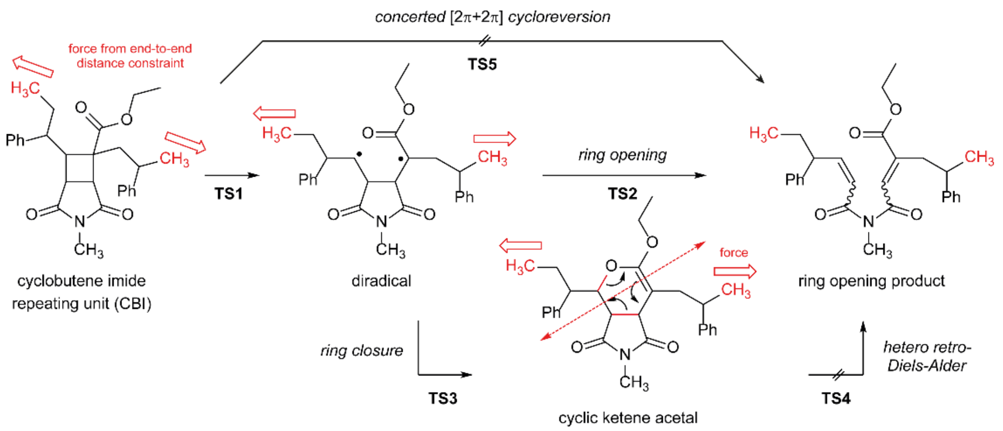

Fig. S111 Reaction and conceivable degradation pathways (labelled TS1-5) emerging from the CoGEF analysis of PS-*co*-PMCBI polymer models (Fig. S110). In the lower formula, the misalignment of the vector of strain with the force direction required to initiate retro-Diels-Alder ring opening (dashed arrow) is indicated.

Table S4. Characteristics of the CoGEF analysis on the degradation mechanism of different diastereomeric of PS-*co*-PMCBI polymer models, c.f. Figs. S110 and S111 (reaction pathways TS1-3), molecular structures are plotted in Fig. S112.

config.[a]	d [b] [Å]	$d_{C\text{-}C}$ [c] [Å]	F_{max} [nN]	d [b] [Å]	$d_{C\text{-}C}$ [c] [Å]	ΔE_{max} [kJ/mol]	remark[d]
***cis*-1**	10.176	1.955	3.2	11.176	3.173	304.2	→TS2
***cis*-2**	10.243	1.949	3.3	10.943	2.953	274.3	→TS3
***trans*-1**	10.383	1.963	3.6	11.333	3.270	322.7	→TS2
***trans*-2**	10.765	1.779	4.8	10.815	[e]	435.6	[e]

[a] initial configuration cf. Fig. S1a. [b] end-to-end (methyl-methyl) distance at F_{max} or ΔE_{max}. [c] C-C distance between radical positions at F_{max} or ΔE_{max}. [d] reaction transition c.f. Fig. S111. [e] chain break.

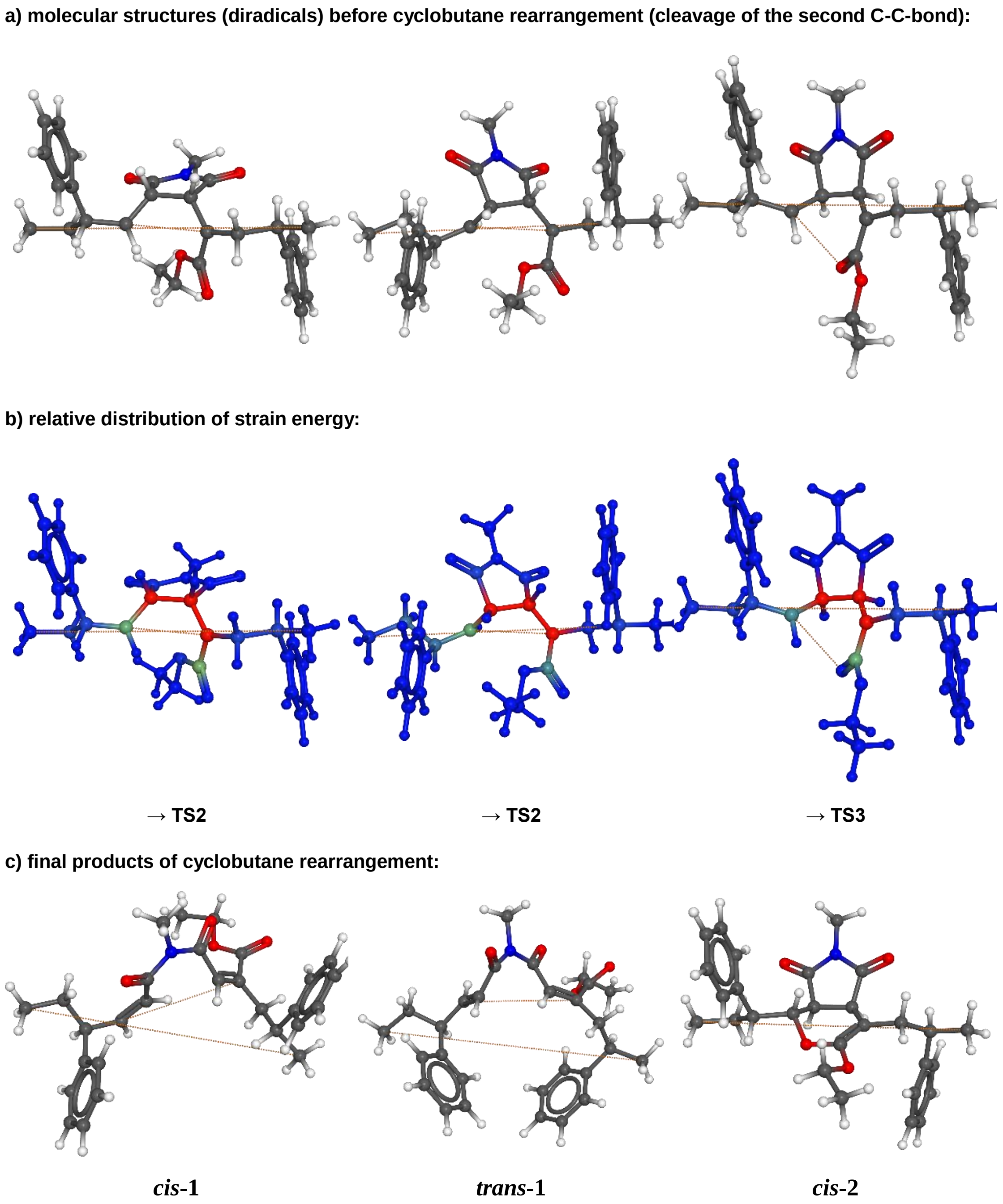

Fig. S112 Molecular structures of PS-*co*-PMCBI polymer models obtained from CoGEF analysis. (a) Molecular structures of the diradicals before rearrangement at the points labelled TS2/3 in Fig. S110c. The dashed orange lines display the strain vector from constrained end-to-end distances, and relevant C···C or C···O vectors for the observed rearrangements. (b) Relative strain-energy mapped onto atomic positions of structures shown in the top row (blue: low strain, red: high strain). (c) Molecular structures of final CoGEF reaction products. Note that the rearrangement of both models ***cis*-1** and ***trans*-1** (left and center column) proceeds via pathway TS2 (cf. Fig. S111), whereas for ***cis*-2** the formation of a pyran ring (right column, TS3) was computed.

a) **b)** **c)**

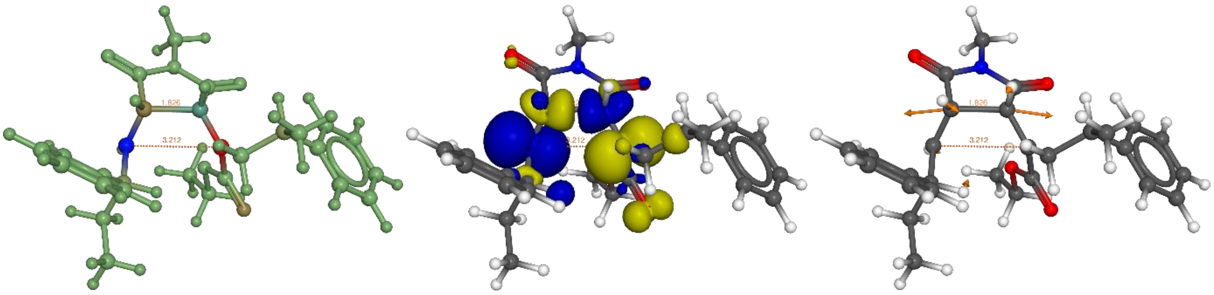

Fig. S113 True transition state computed for the model ***cis*-1** and the cyclobutane ring opening TS2‡ process c.f. Fig. S111. The molecular model was obtained from a separate unconstrained DFT simulation (UB3LYP/6-31G(d)). (a) Molecular model with Mulliken spin densities mapped onto atomic positions of this singlet diradical species. The blue and red carbon atoms correspond to the two radical positions that emerged from the first (completed) C-C-bond rupture with a C-C separation of 3.212 Å. The second cyclobutane bond is elongated to 1.826 Å in this transition state, indicating imminent bond rupture to complete the cyclobutane ring opening. (b) Iso-contour plot of the spin density (difference of electron density for α and β electrons plotted as blue and yellow surfaces) for the transitions state TS2‡. (c) Atomic displacement vectors (scaled by a factor 2.5 for visualization) corresponding to the single negative (imaginary) vibrational eigenvalue of the transition state ($\tilde{\nu}$ = -601.7 cm^{-1}). The large orange vectors associated with the single C-C-bond indicate bond rupture along the intrinsic reaction coordinate (IRC) of this transition state.

The above considerations on the degradation mechanism are further supported by the “true” relaxed electronic transition states identified from unrestrained DFT calculations (Figs. S113-S116). In contrast to the above CoGEF-derived strain-induced transitions states of debonding (labeled TS1-5), these relaxed TS (labeled TS1-5‡) were computed without additional constraints applied to the polymer chain. Thus, TS1-5‡ represent the “natural” intrinsic reaction pathways that are plausible for the corresponding molecular models, and these models have been included here for the sake of completeness of the computational study. In combination with the energy profiles along the various intrinsic reaction coordinates shown in Figs. S114-S116, these TS yield a concise picture of possible reaction pathways that additionally supports the CoGEF analysis described above. The stepwise process (TS1‡ and TS2‡) involving successive bond breakage (first C1 C2 and then C3 C4) and a diradical type intermediate is the most favorable mechanism (Fig. S116), while the concerted ring opening through [2+2] cycloreversion (TS5‡) is disfavored.

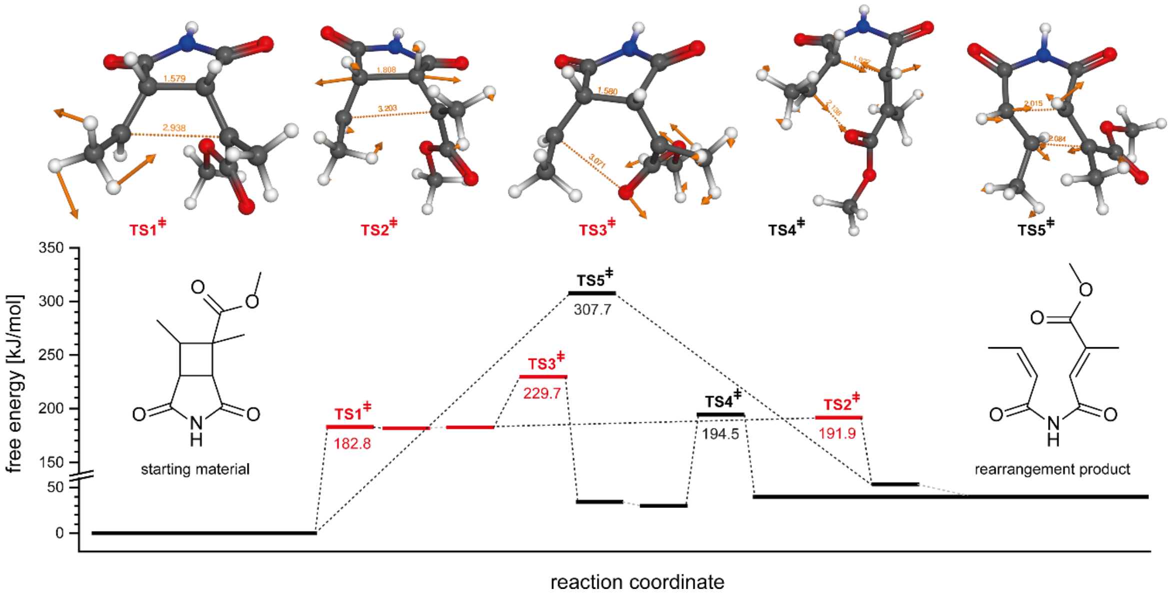

Fig. S114 True transition states and free energy profile computed for a simplified molecular model as shown and the processes TS1-5 cf. Fig. S111 (DFT UB3LYP/6-31G(d), including thermal vibrational corrections for *T*=298.15 K). The dashed lines connect the transition states with forward and reverse reaction products identified from intrinsic reaction coordinate (IRC) analysis following the corresponding eigenvectors in forward and reverse direction, free energies of activation ($\Delta G^{\ddagger}$) are given in [kJ/mol]. Molecular models of the transition states TS1-5‡ are plotted with atomic displacement vectors (scaled by a factor 2.5 for visualization) corresponding to the single negative (imaginary) vibrational eigenvalue of each TS; these vectors define the IRC for each process. Note that for TS1‡ this seems to imply a methyl rotation, but in fact is the process of diradical formation that in reverse corresponds to the TS of radical recombination with a very shallow barrier of activation ($\Delta G^{\ddagger}$=1.0 kJ/mol, $\tilde{\nu}$ = -89.3 cm^{-1}). Red labels correspond to singlet diradical species, closed-shell TS are labeled in black.

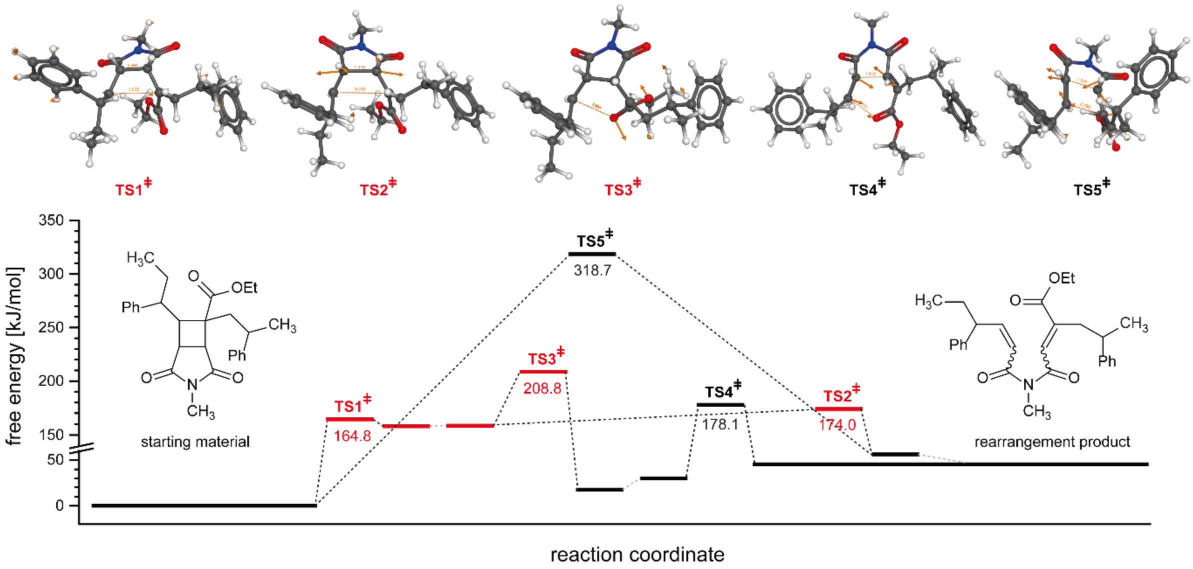

Fig. S115 True transition states and free energy profile computed for the polymer model ***cis*-1** and the processes TS1-5 cf. Fig. S111 (DFT UB3LYP/6-31G(d), including thermal vibrational corrections for *T*=298.15 K). The dashed lines connect the transition states with forward and reverse reaction products

identified from intrinsic reaction coordinate (IRC) analysis following the corresponding eigenvectors in forward and reverse direction, free energies of activation ($\Delta G^{\ddagger}$) are given in [kJ/mol]. Molecular models of the transition states TS1-5‡ are plotted with atomic displacement vectors (scaled by a factor 2.5 for visualization) corresponding to the single negative (imaginary) vibrational eigenvalue of each TS; these vectors define the IRC for each process. Red labels correspond to singlet diradical species, closed-shell TS are labeled in black.

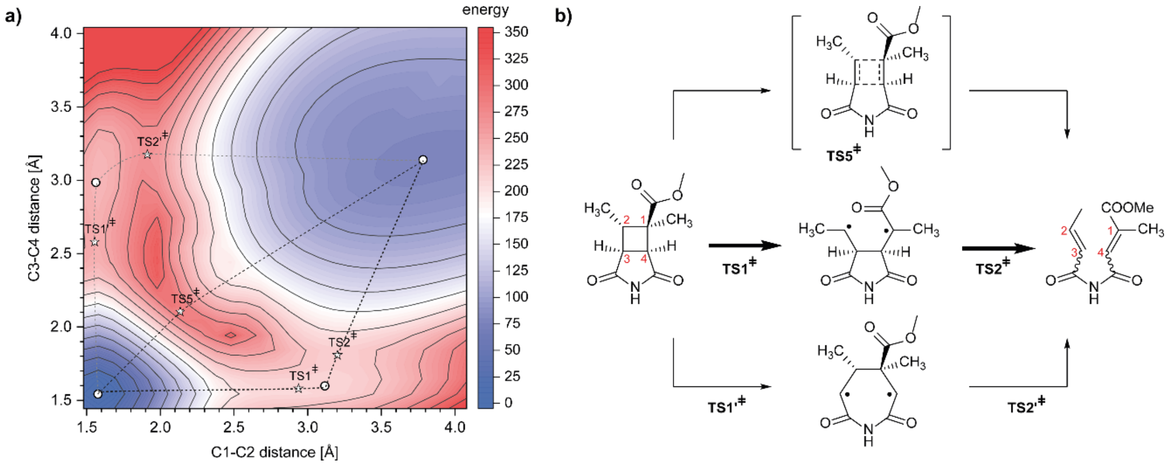

Fig. S116 (a) Adiabatic potential energy surface (PES, DFT UB3LYP/6-31G(d)) electronic energies of the simplified molecular model plotted as a 2D-function of the cyclobutane C1-C2 and C3-C4 separations. (b) Reaction scheme with plausible transition pathways for the cyclobutane ring opening. The transition states are marked in plot a) by asterisks, the corresponding intrinsic reaction coordinates (IRC) are indicated by dashed lines connecting the transition states with the relaxed molecular geometries of the starting material and the product (solid circles), respectively. The concerted ring opening through [2+2] cycloreversion (TS5‡) is disfavored over the stepwise process (TS1‡ and TS2‡) involving successive bond breakage (first C1-C2 and then C3-C4) and a diradical type intermediate. The latter is located at a broad high energy plateau of the PES, with very shallow forward and reversion energy barriers of reaction. On the potential energy surface, the process with the reverse order of bond cleavage (first C3-C4 and then C1-C2, TS1'‡ and TS2'‡) can be identified, but was not observed in the CoGEF analysis of the polymer models (mismatching mode of stress excerpted from external forces).

Computational Methods

Quantum chemical DFT calculations were carried out using the *Gaussian09* program package[20] Unless stated otherwise, calculations were carried out at the unrestricted UB3LYP/6-31G(d) level of theory. All optimized stationary molecular structures were verified by vibrational analysis, and all transition states (TS, first order saddle points) are characterized by exactly one negative (imaginary) vibrational mode. Transition states were located using the quadratic synchronous transit method (QST3) as implemented in Gaussian,[21-22] and all TS were additionally confirmed by visual inspection of the imaginary vibrational mode as well as by intrinsic reaction coordinate (IRC) calculations. Energies reported here are given relative to the most stable conformers of the reactants, all energies were calculated using the harmonic oscillator

approximation. Enthalpies (ΔH) and free energies (ΔG) including zero-point correction, temperature correction, and vibrational energy were computed for standard conditions (T = 298.15 K, P = 1.0 atm) using unscaled vibrational frequencies. Plots and atomic coordinates of all molecular structures are given in the Tables below.

CoGEF Analysis

For all structures along the stretching trajectory of the mechanophores, the total SCF electronic energy $E_{total}(d)$ (DFT UB3LYP/6-31G(d)) was computed as a function of an end-to-end constraint d applied to each molecular geometry:

$$E_{total}(d) = E_{SCF}(d) - E_{SCF}(d_0),$$

where d_0 was the equilibrium end-to-end distance of a fully optimized structure of the mechanophore unit, and $E(d_0)$ was the corresponding global minimum SCF energy. The distance constraint d was increased in steps of 0.05 Å. At each step, a full SCF geometry optimization of all other internal coordinates was carried out. Relative electronic energies (in [kJ mol^{-1}]) were then plotted as a function of the end-to-end distance (in [Å]); the resulting forces (in [nN]) were computed from the partial derivative $\partial E_{total}/\partial d$.
As DFT calculations do not yield split terms of energy on bond length or angle deformations,[23] an *ad hoc* chosen geometry-based mapping of strain-energies onto atomic positions was used. For this, classical harmonic potentials were assumed for both types of deformations:

$$E_{bonds}(d) = \frac{1}{2}K_{bonds}\sum_{bonds}(r - r_0)^2 = \frac{1}{2}K_{bonds}\sum_{bonds}\Delta r^2$$

$$E_{angles}(d) = \frac{1}{2}K_{angles}\sum_{angles}(\alpha - \alpha_0)^2 = \frac{1}{2}K_{angles}\sum_{angles}\Delta\alpha^2$$

where $\Delta r = r - r_0$ and $\Delta\alpha = \alpha - \alpha_0$ are the deformations of all distance and angle parameters relative to the equilibrium (energy minimum) molecular geometry, respectively. Setting

$$E_{total}(d) = E_{bonds}(d) + E_{angles}(d),$$

and choosing the "force constants" K_{bonds} and K_{angles} from:

$$K_{bonds} = E_{total}(d)/\sum_{bonds}(r - r_0)^2$$

$$K_{angles} = E_{total}(d)/\sum_{angles}(\alpha - \alpha_0)^2$$

implies that:

$$E_{bonds}(d) = E_{angles}(d) = \frac{1}{2}E_{total}(d).$$

Note that this ensures an "equipartition" of bond length and angle deformation energies irrespective of the different orders of magnitude of the Δr and $\Delta\alpha$ terms, which is kind of intuitive: higher strain set on bonds will induce angle deformations, and vice versa, until all contributions are equally distributed. With these purely empirical "force constants" K_{bonds} and K_{angles}, individual deformations Δr and $\Delta\alpha$ can now be mapped onto individual atomic positions i:

$$E_i(d) = \frac{1}{4}K_{bonds}\sum \Delta d^2 + \frac{1}{2}K_{angles}\sum \Delta\alpha^2$$

where the sums now run only over all bonds and angles a specific atom is associated with. Note that an additional pre-factor ½ is required on distance violations due to two atoms being involved in each bond, whereas angle deformations are associated with a single central atom only. This definition implies that the total electronic energy of each molecular geometry is also simply the sum of all atomic contributions:

$$E_{total}(d) = \sum_{atoms} E_i(d)$$

Atomic deformation energies were then mapped onto the individual atoms using a 16-color code ranging from blue (low strain) to red (highest strain).

The following tables provide structure plots and atomic coordinates for all characteristic molecular geometries identified from CoGEF analysis (Tables S5a-b) of the polymer models, including all relaxed transition states computed (Tables S6a-e and S7a-e).

Table S5a Molecular structures and atomic coordinates (in [Å]) obtained from the CoGEF analysis of the polymer models just before final rupture of the cyclobutane ring is observed. The dotted orange lines in the structure plots connect the characteristics radical positions involved in the rearrangements, respectively, as well as the terminal methyl groups of the models that were used as constrained "pulling points" in the CoGEF analysis.

***cis*-1**				***trans*-1**				***cis*-2**			
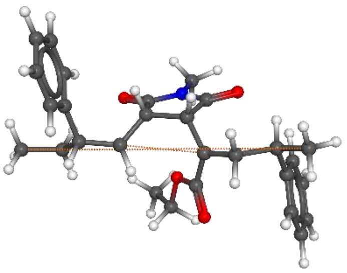				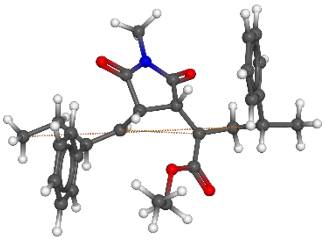				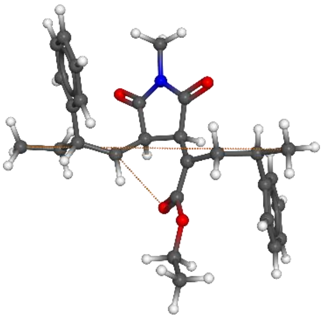			
C	-3.62998551	-2.03536362	4.20713178	C	-5.66610914	-1.48106696	0.72636013	C	-2.02286231	-3.56894401	4.29564735
C	-2.48878428	-1.47143883	3.30645048	C	-4.11809981	-1.70520182	0.65070941	C	-1.05432592	-2.77558775	3.35994076
C	-2.87416735	-1.18774081	1.78958815	C	-3.17799967	-0.43955747	0.93772655	C	-1.70449440	-1.92443715	2.19164641
C	-3.49165018	-2.40915580	1.10308042	C	-3.56779152	0.73371576	0.04718515	C	-2.45500228	-2.73064158	1.12083334
C	-4.68839634	-2.25601796	0.38932093	C	-4.07099740	1.90485135	0.62982186	C	-3.32564427	-2.03089088	0.26851293
C	-2.90103503	-3.68108982	1.15132467	C	-3.50942658	0.65824934	-1.35385110	C	-2.33413027	-4.11535161	0.95159338
C	-5.28656112	-3.33996770	-0.25570795	C	-4.50185371	2.97545559	-0.15626692	C	-4.05851945	-2.69087775	-0.71611913
C	-3.49737801	-4.76550075	0.50455202	C	-3.93409383	1.73063787	-2.14115715	C	-3.07233688	-4.78111426	-0.03107970
C	-4.69165850	-4.60064019	-0.19986256	C	-4.43365679	2.89165175	-1.54737666	C	-3.93665449	-4.07403134	-0.86765612
C	-1.80459176	-0.51903200	0.93572908	C	-1.69452744	-0.77710926	0.98293464	C	-0.74387582	-0.91968645	1.56744723
C	-0.06427636	1.01557473	-1.22916194	C	1.42168841	0.15735929	1.30249171	C	0.07194306	1.10557311	-0.42088754
C	-0.83743716	-1.22951599	0.04611959	C	-0.70121950	-0.61959299	-0.12461457	C	0.75088826	-0.25328684	-0.44333481
C	-0.05821707	-0.49413906	-1.10080038	C	0.79929580	-0.74691559	0.27484897	C	0.50311006	-1.20248904	0.75910101
C	0.26076866	-1.99518564	0.83996188	C	-0.88302700	-1.94244075	-0.94677613	C	0.43424819	-1.14345547	-1.65709625
C	1.36846645	-1.07557297	-0.99216709	C	0.93130240	-2.28057457	0.46498262	C	0.56401904	-2.60006546	0.14357864
O	0.13827269	-2.64601679	1.85948620	O	-1.70020230	-2.17426588	-1.81660688	O	0.30877439	-0.81020933	-2.81804075
O	2.29130572	-0.91889299	-1.76449589	O	1.71895200	-2.92056244	1.12626441	O	0.72485452	-3.66065039	0.71603433
N	1.45349250	-1.86111013	0.15746991	N	0.00883123	-2.86196121	-0.41293177	N	0.41724480	-2.46833048	-1.22695819
C	2.68812886	-2.49337745	0.60470941	C	0.02720315	-4.27526830	-0.75519814	C	0.32580163	-3.60924693	-2.12638042
C	0.06333983	1.85109302	-0.07028813	C	0.88394910	1.45902632	1.67686756	C	0.18328015	1.81717576	0.82999167
O	-0.09161092	3.06758623	-0.00720526	O	1.27031207	2.08684895	2.65919004	O	0.79458221	1.40860988	1.82847333
O	0.40653418	1.10834345	1.06100326	O	-0.00929340	1.98542494	0.77965737	O	-0.46577565	3.02296171	0.84526283
C	0.65770703	1.89063560	2.24514542	C	-0.57112561	3.27359425	1.09886087	C	-0.30950452	3.79828870	2.04536376
C	1.18914305	0.95601625	3.31670739	C	-0.78966964	4.03043371	-0.19976950	C	-1.16389395	5.04476245	1.89730314
C	-0.00720494	1.69213418	-2.58770455	C	2.86133913	-0.06887138	1.76009270	C	-0.15317411	1.91362149	-1.69807955
C	1.42453298	1.92771389	-3.24782116	C	3.99424110	0.75513648	1.00129962	C	1.14085450	2.39299178	-2.49418769
C	2.35600324	2.75285768	-2.36869311	C	4.04455781	0.37769556	-0.47188245	C	2.19871605	3.02506824	-1.60083000
C	2.09053831	4.09996621	-2.08029091	C	4.45309628	-0.90198136	-0.88330018	C	1.97841997	4.24694141	-0.94568248
C	3.53379648	2.18871133	-1.86011926	C	3.66845753	1.29714514	-1.46094863	C	3.44849167	2.41173198	-1.43982558
C	4.42393460	2.94679826	-1.09528705	C	3.70032220	0.95653112	-2.81558165	C	4.44455690	2.98796434	-0.64783377
C	2.97536250	4.85989782	-1.31720458	C	4.48784878	-1.24640992	-2.23462067	C	2.96950526	4.82832194	-0.15510554
C	4.14959246	4.28693709	-0.82321816	C	4.11151934	-0.31742437	-3.20803029	C	4.20820643	4.20045211	-0.00157921
C	1.34974467	2.55824477	-4.68080686	C	5.42351625	0.66130323	1.65392632	C	0.81436350	3.34319202	-3.69926523
H	-3.25390135	-2.14214850	5.22993900	H	-6.16857665	-2.43793727	0.55151550	H	-1.43322770	-4.07389201	5.06778770
H	-4.49600316	-1.36327325	4.23054866	H	-5.97067935	-1.10812961	1.71114523	H	-2.73188137	-2.89684524	4.79291471
H	-3.96870084	-3.01576510	3.86005572	H	-6.00327905	-0.76760858	-0.03069413	H	-2.59757900	-4.32368496	3.75135121
H	-1.63501986	-2.15143197	3.32088608	H	-3.86181930	-2.09180276	-0.34063336	H	-0.31588931	-3.45182512	2.91960578
H	-2.13766428	-0.52129200	3.72784396	H	-3.83812640	-2.48381737	1.37214452	H	-0.48790546	-2.07136331	3.98200815
H	-3.66784019	-0.42841331	1.85009293	H	-3.42091038	-0.15526831	1.97499918	H	-2.46407286	-1.29837487	2.68152380
H	-5.16003491	-1.27680319	0.34067743	H	-4.13524080	1.97655257	1.71361672	H	-3.42227598	-0.95325662	0.38054975
H	-1.96985787	-3.82132266	1.69125454	H	-3.13488973	-0.24242782	-1.83281012	H	-1.65248414	-4.68242651	1.57446687
H	-6.21667100	-3.19740078	-0.79959316	H	-4.89328842	3.87088240	0.31953742	H	-4.72599111	-2.12638343	-1.36182689
H	-3.02709308	-5.74415585	0.55626504	H	-3.87993971	1.65319273	-3.22401691	H	-2.96899518	-5.85790327	-0.13846591
H	-5.15431648	-5.44744338	-0.69953979	H	-4.76974691	3.72135559	-2.16353684	H	-4.51077751	-4.59478195	-1.62937058
H	-1.83153301	0.55991314	0.88535108	H	-1.41400851	-1.47022517	1.77582929	H	-0.73815554	0.05974371	2.02155560
H	-1.39616890	-2.03119947	-0.46462869	H	-0.91525507	0.24498734	-0.74910932	H	1.84711185	-0.09459560	-0.54103912
H	-0.44693431	-0.88706251	-2.04965839	H	1.39453259	-0.63060260	-0.65764139	H	1.33704110	-1.12605572	1.47831534
H	2.47876728	-3.52001206	0.91176283	H	-0.82727294	-4.47014784	-1.40448922	H	0.30830135	-3.22444129	-3.14631898
H	3.38871022	-2.47366647	-0.23044590	H	0.95410658	-4.53099646	-1.27731189	H	1.18777064	-4.26547648	-1.97979675
H	3.11066599	-1.94751702	1.45375911	H	-0.03980246	-4.87741272	0.15490369	H	-0.58869135	-4.17139088	-1.92020551
H	1.37429865	2.67885136	1.99491231	H	-1.51916011	3.10924343	1.62606229	H	-0.61605329	3.19799063	2.90874490
H	-0.26969611	2.38552158	2.55521376	H	0.10997394	3.79825186	1.77173291	H	0.75022679	4.04313694	2.17743975
H	2.13320975	0.49873408	3.00201589	H	-1.46273171	3.48551485	-0.86824418	H	-2.21784528	4.78194591	1.75865037
H	1.37160877	1.51406935	4.24183336	H	-1.23673381	5.00882040	0.01111085	H	-1.07854577	5.66637986	2.79561751
H	0.47560319	0.15331704	3.53091835	H	0.16230134	4.19250032	-0.71688050	H	-0.84445339	5.64073773	1.03581981
H	-0.59271898	1.10882723	-3.31267824	H	2.90229280	0.25168242	2.80791833	H	-0.73817062	1.32652464	-2.41315131
H	-0.49266271	2.66973283	-2.48144963	H	3.10140585	-1.13351044	1.72017623	H	-0.73980367	2.79493962	-1.42688511

H	1.87393446	0.93577351	-3.35352742	H	3.69120744	1.80703367	1.06716734	H	1.57461448	1.48190434	-2.92169765
H	1.17474510	4.55930558	-2.44202790	H	4.74143397	-1.64031391	-0.13896900	H	1.01907309	4.74634131	-1.04989221
H	3.74828216	1.14287199	-2.06049550	H	3.34469299	2.29238306	-1.16448474	H	3.64813095	1.47249492	-1.95241888
H	5.33363135	2.48763488	-0.71517077	H	3.40444545	1.68885870	-3.56257420	H	5.40484914	2.48975702	-0.54070617
H	2.74473108	5.90052944	-1.10337557	H	4.81033036	-2.24235790	-2.52815489	H	2.77642995	5.77729239	0.33947926
H	4.84116512	4.88015364	-0.23001816	H	4.13908869	-0.58509472	-4.26097830	H	4.98000285	4.65405535	0.61456520
H	0.90959144	3.56136470	-4.65772705	H	5.80475810	-0.36564553	1.64821843	H	0.37234488	4.28592419	-3.35820065
H	2.35904567	2.63826217	-5.09569867	H	6.11983418	1.28953798	1.09013634	H	1.73526267	3.57334565	-4.24374946
H	0.74503932	1.93710760	-5.35311588	H	5.39475695	1.01022365	2.69195319	H	0.10993433	2.86515100	-4.38962221

Table S5b Molecular structures and atomic coordinates (in [Å]) obtained from the CoGEF analysis of the polymer models after final rupture of the cyclobutane ring.

***cis*-1**				***trans*-1**				***cis*-2**			
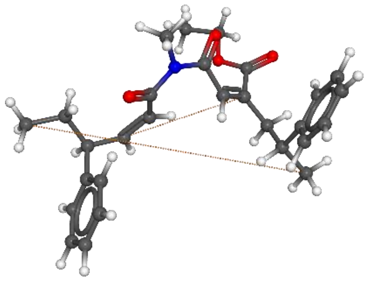				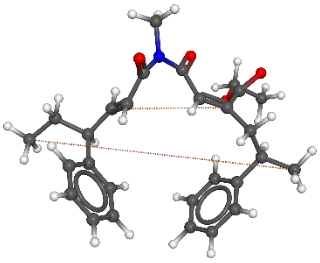				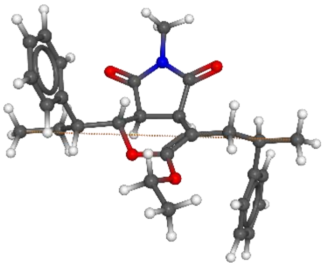			
C	-3.46312702	-4.09883619	3.66577900	C	-6.10878026	0.41192019	-1.19356954	C	-1.18827946	-3.39773350	4.22860164
C	-2.51063924	-3.09934134	2.99618757	C	-5.06214322	-0.48191326	-0.51254554	C	-0.44408510	-2.66459640	3.05264782
C	-3.14200251	-2.42381420	1.75510708	C	-3.57669586	-0.12858115	-0.82464259	C	-1.31074449	-1.66652970	2.20632829
C	-3.39043750	-3.38429774	0.58600420	C	-3.18390960	-0.21089400	-2.29443856	C	-2.62371310	-2.30616419	1.75694473
C	-4.69556173	-3.63652668	0.14499681	C	-2.21179454	0.67005330	-2.79024616	C	-3.84672361	-1.72218873	2.11202666
C	-2.32534165	-4.02480485	-0.06635888	C	-3.71954658	-1.16586005	-3.16956153	C	-2.64776774	-3.47411911	0.97738857
C	-4.93945215	-4.51085074	-0.91653880	C	-1.78888810	0.60461866	-4.11718719	C	-5.06024151	-2.27947557	1.70319503
C	-2.56842820	-4.89906587	-1.12523424	C	-3.30227945	-1.23161770	-4.50113345	C	-3.85898462	-4.03312065	0.56774117
C	-3.87476945	-5.14566452	-1.55542733	C	-2.33625587	-0.34652441	-4.98030694	C	-5.07041104	-3.43866487	0.92771885
C	-2.48227202	-1.15019296	1.24217343	C	-2.69549598	-0.97564087	0.07672825	C	-0.67361018	-0.88558237	0.95781747
C	0.72144761	2.46628732	-0.36894625	C	2.00494578	-0.48009811	1.39868983	C	0.36474928	1.11676131	-0.80633879
C	-1.21793998	-0.69356860	1.13032844	C	-1.91571803	-2.00391221	-0.29364615	C	1.08703587	-0.22534381	-0.80024311
C	0.86335498	1.17249749	-0.03217672	C	0.86621235	-1.15026501	1.14113224	C	0.71447799	-1.23141482	0.35219793
C	0.04319155	-1.39209450	1.46702434	C	-1.39855423	-2.99664451	0.68271900	C	0.92224254	-1.04779188	-2.08235648
C	1.44270230	0.72522319	1.26798198	C	-0.07560722	-1.52038812	2.23365691	C	0.71220697	-2.59149779	-0.34638300
O	0.12796144	-2.60048516	1.65654184	O	-1.46118415	-4.19176948	0.44515965	O	0.97776446	-0.66398781	-3.23317056
O	2.23031093	1.42500042	1.88834952	O	0.05944396	-1.10129788	3.37724908	O	0.65641499	-3.69797867	0.16084320
N	1.19789716	-0.60428424	1.67763697	N	-1.01410652	-2.53492851	1.96795152	N	0.75344148	-2.38507604	-1.71637625
C	2.24864526	-1.26084678	2.47463400	C	-1.36267180	-3.40703710	3.10154784	C	0.68377472	-3.47719348	-2.67547466
C	0.98233319	3.57036071	0.62362154	C	2.32518917	0.06149951	2.77296812	C	-0.35361832	1.39616444	0.30250490
O	1.73818273	4.49263542	0.39305957	O	3.13849125	-0.46327386	3.50267031	O	-0.61458626	0.51175003	1.32409088
O	0.22898624	3.45755715	1.72786555	O	1.71030423	1.23427201	3.01404826	O	-0.85244185	2.62881416	0.56000029
C	0.51255588	4.41056098	2.78213396	C	1.88040144	1.78433314	4.34636316	C	-2.19236349	2.70739051	1.09055559
C	-0.44933812	4.12801971	3.91975278	C	3.14563397	2.62147313	4.45639365	C	-2.56312843	4.17733576	1.15810300
C	0.35934264	2.96758467	-1.75230810	C	3.17049183	-0.40709085	0.43715259	C	0.45088040	2.10669539	-1.98715449
C	0.80058968	2.12344096	-2.96628216	C	3.75680323	1.01232771	0.16401968	C	1.84927665	2.58087369	-2.52174930
C	2.29006774	1.79383239	-3.00765404	C	2.86693322	1.79602503	-0.79549642	C	2.79056190	3.07442636	-1.43150521
C	3.26811431	2.62816163	-2.44864659	C	3.03160612	1.72693064	-2.18625845	C	2.35171810	3.88553034	-0.37402233
C	2.70992322	0.63740691	-3.68105125	C	1.82435825	2.58785077	-0.29167837	C	4.15449166	2.75129082	-1.48978950
C	4.06320646	0.32358205	-3.80434902	C	0.97832763	3.29380932	-1.14832246	C	5.05492483	3.22578156	-0.53455833
C	4.62386407	2.31506821	-2.56846983	C	2.18846663	2.43269564	-3.04650252	C	3.24806747	4.36095838	0.58445876
C	5.02771554	1.16582735	-3.24848673	C	1.15881411	3.22181092	-2.53113744	C	4.60362892	4.03597352	0.50807442
C	0.38317040	2.85630197	-4.26180043	C	5.22748392	0.90009692	-0.28997217	C	1.74096188	3.66548275	-3.66908121
H	-2.99220838	-4.54597061	4.54833252	H	-7.11863051	0.11273254	-0.89246157	H	-0.46817487	-4.01348121	4.77669102
H	-4.39198179	-3.61463728	3.99411254	H	-5.97344161	1.46372193	-0.91346723	H	-1.63340387	-2.68306878	4.93111245
H	-3.73457963	-4.91220829	2.98386075	H	-6.04702169	0.34732221	-2.28451047	H	-1.98378362	-4.04560724	3.85061282
H	-1.57749398	-3.59164137	2.72193966	H	-5.24064570	-1.53247391	-0.77282527	H	-0.00927433	-3.42016224	2.39261005
H	-2.24652835	-2.31195566	3.71495026	H	-5.19468501	-0.42211938	0.57578441	H	0.40040887	-2.10250101	3.47471319
H	-4.13799958	-2.08758803	2.07751128	H	-3.43282321	0.91594541	-0.50741017	H	-1.57907581	-0.84399204	2.88013384
H	-5.53185457	-3.14737582	0.64025937	H	-1.76931789	1.40803570	-2.12466540	H	-3.84916930	-0.82043856	2.72069016
H	-1.30878887	-3.83306816	0.26287314	H	-4.47028285	-1.86701673	-2.81610903	H	-1.71411590	-3.95097159	0.69230098
H	-5.96066734	-4.69478177	-1.24059296	H	-1.02834981	1.29506521	-4.47187001	H	-5.99573120	-1.80824952	1.99408313
H	-1.73270593	-5.39006321	-1.61767488	H	-3.73410411	-1.97824786	-5.16251687	H	-3.85560654	-4.93919128	-0.03314014
H	-4.05995878	-5.82771462	-2.38117638	H	-2.01190965	-0.39856271	-6.01619231	H	-6.01234212	-3.87688240	0.60859403
H	-3.22600209	-0.43225925	0.89285517	H	-2.79873225	-0.76202294	1.14082065	H	-1.39863322	-0.95984509	0.13678413
H	-1.11661897	0.33080336	0.78874233	H	-1.83403594	-2.30242357	-1.33493476	H	2.17119871	-0.03376965	-0.73593073
H	0.68282105	0.39918378	-0.77401076	H	0.71752446	-1.57989271	0.15537008	H	1.46366701	-1.23401816	1.14868268
H	2.61596963	-2.14044775	1.94148379	H	-2.29880309	-3.91871110	2.87774928	H	0.82572162	-3.05061992	-3.66885644
H	3.04769634	-0.54110720	2.63458776	H	-0.58458100	-4.15816469	3.27502429	H	1.46328391	-4.21217015	-2.45880106
H	1.84536415	-1.58655408	3.43711588	H	-1.46864925	-2.78472330	3.98883604	H	-0.29007169	-3.97136864	-2.61551901

H	1.55661099	4.27996522	3.08252229	H	0.98357458	2.38954554	4.49836998	H	-2.87447072	2.15382743	0.43132950
H	0.39804647	5.42289065	2.38246514	H	1.88166554	0.95802237	5.06031418	H	-2.22149989	2.24301905	2.08069573
H	-0.31965976	3.10769345	4.29386089	H	3.15129213	3.42425309	3.71109850	H	-2.54081933	4.63282191	0.16292074
H	-0.26215086	4.82451962	4.74426970	H	3.20034458	3.07980327	5.45082733	H	-3.57208771	4.29106889	1.56943714
H	-1.48819838	4.24978269	3.59591639	H	4.03330796	1.99881199	4.31649792	H	-1.86407457	4.72079934	1.80188883
H	-0.73308264	3.09352691	-1.80163039	H	3.96402231	-1.01304291	0.89859763	H	-0.05640942	1.65165107	-2.84692014
H	0.76882000	3.97945682	-1.84527312	H	2.90521670	-0.88699749	-0.51133559	H	-0.14230757	2.98190647	-1.70622083
H	0.25247970	1.17081327	-2.94352309	H	3.76059954	1.55661843	1.11656787	H	2.31707066	1.70190820	-2.98301496
H	2.98229391	3.51430367	-1.88923067	H	3.82980414	1.12316437	-2.60922453	H	1.29851374	4.13436598	-0.28294452
H	1.96395726	-0.02609319	-4.11538215	H	1.67205759	2.64314721	0.78301690	H	4.51518683	2.12081871	-2.30068531
H	4.36273766	-0.58016826	-4.32898792	H	0.18376353	3.90908252	-0.73325890	H	6.10687780	2.95966381	-0.60418274
H	5.36475909	2.97142193	-2.11958723	H	2.34278391	2.37062914	-4.12086358	H	2.88354454	4.98426583	1.39736792
H	6.08347519	0.92387397	-3.33749777	H	0.51004579	3.78236713	-3.19939411	H	5.29972734	4.40612229	1.25633512
H	0.90923726	3.81394820	-4.34863173	H	5.33870136	0.25412513	-1.16871343	H	1.30502110	4.59567417	-3.28702642
H	0.63152784	2.25774199	-5.14379190	H	5.63601312	1.88431473	-0.54131371	H	2.73882908	3.88783461	-4.05823681
H	-0.69564818	3.05484945	-4.27499107	H	5.83605983	0.46928995	0.51233300	H	1.11604130	3.29714827	-4.49070388

Transition States

Table S6a Molecular structure plots of the truncated polymer models (MCBI unit only), computed energies (excerpts from the *Gaussian* output) and atomic coordinates (in [Å]) for the intrinsic reaction coordinate **TS1** (starting geometry, transition state, and final product geometry). The dotted orange lines indicate characteristic intramolecular atom-atom distances. For the transition state, the orange vectors visualize the eigenvector associated with the vibrational mode of negative (imaginary) frequency (atomic displacement vectors are scaled by a factor of 2.5 for clarity). For the singlet diradical species involved in this process, the models displayed in the second row show the Mulliken spin densities mapped on atomic positions in color coded form (blue and red: high negative and positive spin density; N/A: closed-shell non-radical species). The models displayed in the third row show the 3D-spin SCF density iso-contour surfaces of the diradicals (blue and yellow lobes of excess α- and β-electron density).

	**starting geometry**	**transition state**	**product geometry**
**TS1**	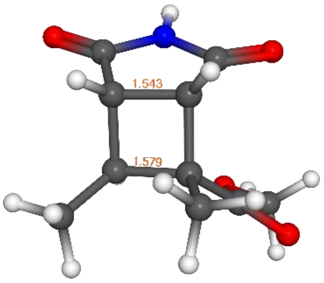	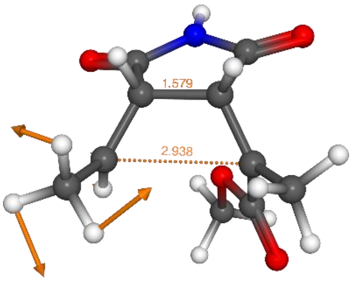	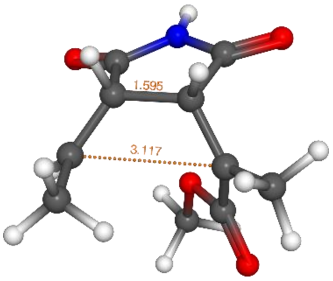
	N/A	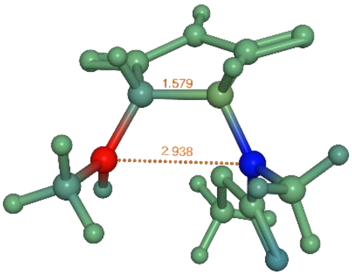	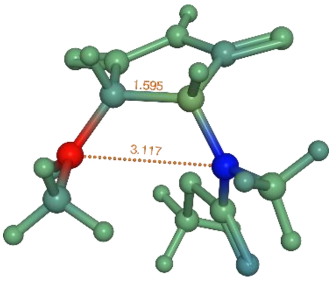

N/A	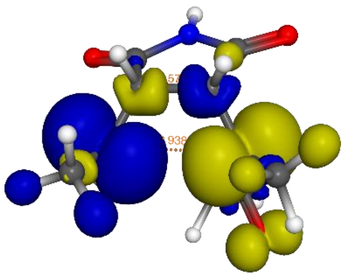	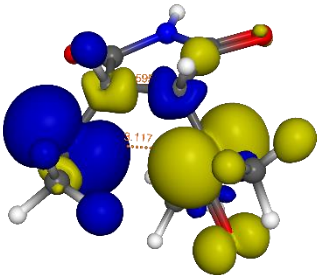
SCF Energy: E(UB3LYP) = -744.556822101 A.U.<br>Zero-point correction= 0.226808<br>Thermal correction to Energy= 0.241364<br>Thermal correction to Enthalpy= 0.242308<br>Thermal correction to Gibbs Free Energy= 0.185067<br>Sum of electronic and zero-point Energies= -744.330014<br>Sum of electronic and thermal Energies= -744.315458<br>Sum of electronic and thermal Enthalpies= -744.314514<br>Sum of electronic and thermal Free Energies= -744.371755	SCF Energy: E(UB3LYP) = -744.477190461 A.U.<br>Zero-point correction= 0.220137<br>Thermal correction to Energy= 0.236068<br>Thermal correction to Enthalpy= 0.237012<br>Thermal correction to Gibbs Free Energy= 0.175075<br>Sum of electronic and zero-point Energies= -744.257053<br>Sum of electronic and thermal Energies= -744.241123<br>Sum of electronic and thermal Enthalpies= -744.240179<br>Sum of electronic and thermal Free Energies= -744.302116<br>Imaginary frequency: -89.2919 cm-1	SCF Energy: E(UB3LYP) = -744.477572195 A.U.<br>Zero-point correction= 0.220280<br>Thermal correction to Energy= 0.236808<br>Thermal correction to Enthalpy= 0.237752<br>Thermal correction to Gibbs Free Energy= 0.175058<br>Sum of electronic and zero-point Energies= -744.257292<br>Sum of electronic and thermal Energies= -744.240764<br>Sum of electronic and thermal Enthalpies= -744.239820<br>Sum of electronic and thermal Free Energies= -744.302515
C -2.67520015 -0.39920242 1.56261535	C -2.88829507 -0.86908084 1.81291420	C -2.62177399 -0.52231235 1.81744771
C -1.31445172 -0.35160112 0.88161487	C -1.42882878 -0.78119775 1.51546771	C -1.21473525 -0.98316997 1.67234563
C -1.02994820 0.73503319 -0.22843073	C -0.92149507 1.00792115 -0.75962284	C -0.97937292 0.97242406 -0.74279598
C -0.98042585 -1.48219100 -0.15351401	C -0.81093090 -1.38340091 0.29450521	C -0.68700937 -1.44737412 0.36349128
C -0.42891187 -0.41773350 -1.12464851	C -0.67674079 -0.46397667 -0.98217990	C -0.73641016 -0.49667553 -0.91632389
C 0.19870524 -2.36113507 0.22548276	C 0.61147495 -1.92018273 0.51616109	C 0.77341700 -1.90141038 0.43725770
C 1.08439354 -0.62678393 -1.11539906	C 0.73928292 -0.76348273 -1.52190975	C 0.62926094 -0.75524768 -1.60346641
O 0.21227551 -3.35704476 0.91524314	O 1.03542807 -2.51249965 1.48246831	O 1.32613380 -2.46661424 1.35384876
O 1.93222442 0.03581287 -1.67263480	O 1.23636473 -0.33086336 -2.53719811	O 0.98796026 -0.31884184 -2.67401990
N 1.32565989 -1.77149172 -0.35652516	N 1.34301700 -1.64379699 -0.63684120	N 1.37162521 -1.58308232 -0.77885662
H 2.26148766 -2.13070642 -0.20711696	H 2.31476441 -1.91502696 -0.73290897	H 2.33913106 -1.80996636 -0.97818342
C -0.05161924 1.84834986 0.13489174	C -0.05777947 1.82663976 0.08292947	C -0.16940842 1.79513237 0.14606857
O -0.12103706 2.98956256 -0.26558868	O -0.21415984 3.02103928 0.29192478	O -0.34128629 2.98820723 0.35135553
O 0.93326803 1.40530672 0.94762059	O 0.97113304 1.11336755 0.62545999	O 0.82833253 1.08119107 0.74343908
C 1.98476661 2.34986725 1.21716848	C 1.87809490 1.86748340 1.44366576	C 1.66622774 1.82486769 1.64067127
H 2.65948961 1.84547697 1.90921143	H 2.61457204 1.14798968 1.80253956	H 2.37872415 1.10277248 2.04041988
C -2.27171896 1.34116062 -0.88362663	C -2.04538765 1.67653438 -1.47631129	C -1.99095887 1.64967819 -1.60620001
H -1.97826772 1.95622892 -1.73830451	H -1.87864421 1.64308426 -2.56424655	H -1.67008330 1.61658864 -2.65935669
H -0.53422154 -0.30509194 1.64508533	H -0.77915026 -0.26554983 2.21471765	H -0.52202489 -0.90967972 2.50276105
H -1.83989166 -2.07578602 -0.47142890	H -1.40568599 -2.26044127 -0.00193552	H -1.25451156 -2.34451077 0.05637059
H -0.78167230 -0.40231263 -2.15971145	H -1.37693025 -0.79767774 -1.75503059	H -1.49510831 -0.86911323 -1.61238294
H 2.50323449 2.60298815 0.28929782	H 2.36044602 2.65308354 0.85592980	H 2.18542629 2.62428913 1.10508784
H 1.57676348 3.25841609 1.66607756	H 1.34810966 2.32677422 2.28232431	H 1.07144184 2.26577915 2.44499786
H -2.95403323 0.55949921 -1.23320922	H -2.99811542 1.15896892 -1.29295753	H -2.96350200 1.14031069 -1.55982541
H -2.81402739 1.98442964 -0.18383764	H -2.13748871 2.71977309 -1.17004018	H -2.11451162 2.69427802 -1.31509553
H -3.49860826 -0.46801356 0.84410881	H -3.40121965 -1.57336503 1.14728057	H -3.32991201 -1.19058324 1.30451930
H -2.73020267 -1.27546977 2.21867176	H -3.06746039 -1.19519376 2.84760863	H -2.91940179 -0.45397598 2.86852813
H -2.83906188 0.49123817 2.18082622	H -3.39528301 0.10488227 1.71124342	H -2.77276064 0.47930817 1.37317517

Table S6b Molecular structure plots, computed energies (excerpts from the *Gaussian* output) and atomic coordinates (in [Å]) for the intrinsic reaction coordinate **TS2** (for details, see also Table S6a).

	**starting geometry**	**transition state**	**product geometry**
**TS2**	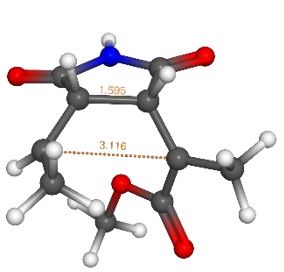	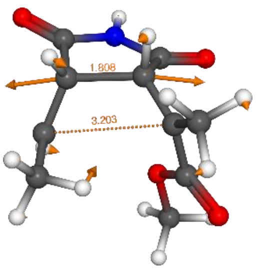	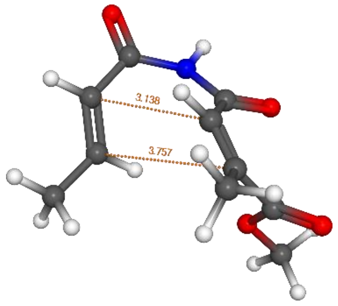
	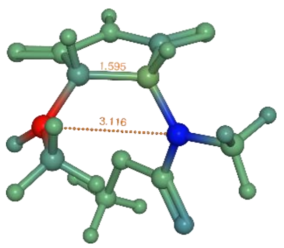	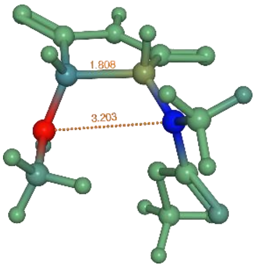	N/A
	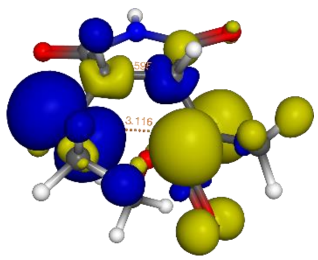	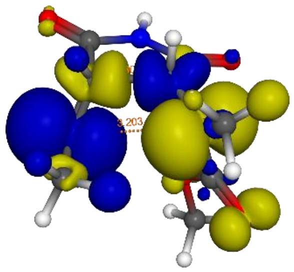	N/A
	SCF Energy: E(UB3LYP) = -744.477545170 A.U.<br>Zero-point correction= 0.220339<br>Thermal correction to Energy= 0.236800<br>Thermal correction to Enthalpy= 0.237744<br>Thermal correction to Gibbs Free Energy= 0.175258<br>Sum of electronic and zero-point Energies= -744.257206<br>Sum of electronic and thermal Energies= -744.240745<br>Sum of electronic and thermal Enthalpies= -744.239801<br>Sum of electronic and thermal Free Energies= -744.302287	SCF Energy: E(UB3LYP) = -744.474679180 A.U.<br>Zero-point correction= 0.219792<br>Thermal correction to Energy= 0.235632<br>Thermal correction to Enthalpy= 0.236576<br>Thermal correction to Gibbs Free Energy= 0.176007<br>Sum of electronic and zero-point Energies= -744.254887<br>Sum of electronic and thermal Energies= -744.239047<br>Sum of electronic and thermal Enthalpies= -744.238103<br>Sum of electronic and thermal Free Energies= -744.298672<br>Imaginary frequency: -552.5044 cm-1	SCF Energy: E(UB3LYP) = -744.528225548 A.U.<br>Zero-point correction= 0.222608<br>Thermal correction to Energy= 0.239321<br>Thermal correction to Enthalpy= 0.240265<br>Thermal correction to Gibbs Free Energy= 0.176792<br>Sum of electronic and zero-point Energies= -744.305618<br>Sum of electronic and thermal Energies= -744.288905<br>Sum of electronic and thermal Enthalpies= -744.287960<br>Sum of electronic and thermal Free Energies= -744.351434
	H -3.09080623 -0.82776209 2.56485946<br>C -2.72248358 -0.79247729 1.53498217<br>H -3.38018960 -1.42145518 0.91554696<br>C -1.29874898 -1.21325992 1.44373251<br>C -0.95553089 1.03296250 -0.68914306<br>C -0.66358717 -1.49884557 0.13168149<br>C -0.65395532 -0.39716433 -1.02187977<br>C 0.80241295 -1.92252836 0.25809289<br>C 0.76488614 -0.53502967 -1.63198931<br>O 1.30710420 -2.58486095 1.13656493<br>O 1.18306083 0.03878066 -2.61235387<br>N 1.47486637 -1.43915100 -0.86090777<br>H 2.46093888 -1.61637948 -1.01428919<br>C -0.23868668 1.75699674 0.35259802<br>O -0.46128232 2.91171050 0.68741350<br>O 0.73395261 0.99615749 0.93367235<br>C 1.48152257 1.64142448 1.97523437<br>H 2.18426251 0.89066256 2.33770355<br>C -1.91596687 1.79229130 -1.54287473<br>H -1.50450069 1.91129250 -2.55777429<br>H -2.86574897 0.24308060 1.17371896<br>H -0.67340714 -1.24342895 2.32845198<br>H -1.17718933 -2.36443414 -0.32503513<br>H -1.34797912 -0.69785913 -1.81355283	H -2.31776088 -0.41086478 2.88022251<br>C -2.23801948 -0.55589653 1.79983854<br>H -3.14656742 -1.05298228 1.43536036<br>C -1.00395423 -1.26106805 1.39495801<br>C -0.91439376 1.12145067 -0.74376200<br>C -0.80126708 -1.58911582 0.01928418<br>C -0.38884743 -0.18343539 -1.04006803<br>C 0.42003250 -2.46770641 -0.23245534<br>C 1.12853345 -0.38044482 -1.12170533<br>O 0.51188566 -3.65322914 0.00214290<br>O 1.93483631 0.40995385 -1.55492076<br>N 1.44321117 -1.68238159 -0.74048320<br>H 2.37210493 -2.05919229 -0.89249488<br>C -0.32144893 1.96574545 0.28794784<br>O -0.58628806 3.14215898 0.48205533<br>O 0.57151968 1.27349265 1.05967885<br>C 1.24085350 2.05180998 2.06101052<br>H 1.90331132 1.35790044 2.58022970<br>C -2.04396663 1.69898313 -1.54680045<br>H -1.67558249 2.13631607 -2.48748168<br>H -2.23324268 0.44816763 1.31709098<br>H -0.15547047 -1.27397276 2.07242403<br>H -1.69915567 -1.97944470 -0.46604662<br>H -0.79984290 -0.55049733 -1.98766092	H -2.50177039 -1.57803520 3.11401372<br>C -2.65879049 -1.44584230 2.03540232<br>H -3.42285241 -2.15693161 1.70705166<br>C -1.37289704 -1.61750234 1.28811514<br>C -0.73332403 1.42159746 -0.82523013<br>C -1.16041525 -2.54419590 0.34232111<br>C -0.36818310 0.15054927 -1.05640868<br>C 0.14386538 -2.82282694 -0.30275975<br>C 0.96740339 -0.40193783 -0.69696584<br>O 0.46614257 -3.95961665 -0.61005640<br>O 1.98209880 0.27892169 -0.68478811<br>N 1.07462309 -1.78029800 -0.47967512<br>H 2.02622282 -2.11912624 -0.58991656<br>C 0.12641179 2.37415910 -0.04043793<br>O 0.41570944 3.48376804 -0.43172683<br>O 0.45814751 1.88109674 1.16947503<br>C 1.37514381 2.69134724 1.92721141<br>H 1.48571541 2.18664696 2.88716118<br>C -1.99755070 2.02743895 -1.36979120<br>H -1.75019047 2.91800370 -1.95842532<br>H -3.04768276 -0.42756971 1.90101228<br>H -0.57072068 -0.93360497 1.56016763<br>H -1.92791148 -3.27458855 0.09661026<br>H -1.03734520 -0.50004743 -1.61279898

H 2.01401864 2.51123246 1.58108106	H 1.81796816 2.85524629 1.59574171	H 2.33539602 2.74430968 1.40805449
H 0.81679592 1.96776348 2.77943935	H 0.51996785 2.49094801 2.75619693	H 0.97686124 3.69987436 2.06178463
H -2.86802456 1.25506628 -1.65256734	H -2.77778182 0.92985886 -1.81815887	H -2.54332128 1.32040029 -2.00022153
H -2.10434007 2.78452324 -1.12895494	H -2.54621893 2.49896932 -0.99808293	H -2.66035814 2.35878283 -0.55973788

Table S6c Molecular structure plots, computed energies (excerpts from the *Gaussian* output) and atomic coordinates (in [Å]) for the intrinsic reaction coordinate **TS3** (for details, see also Table S6a).

	**starting geometry**	**transition state**	**product geometry**
**TS3**	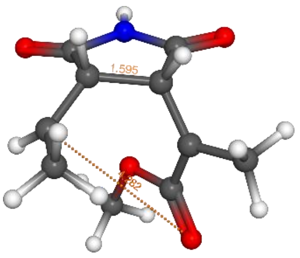	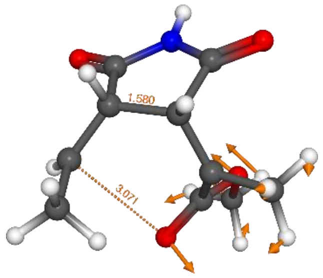	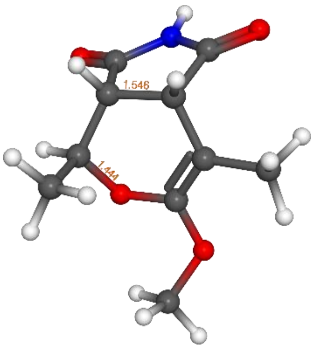
	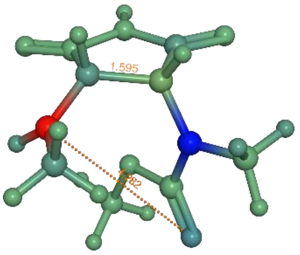	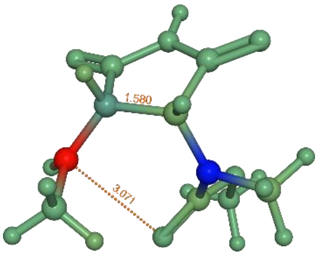	N/A
	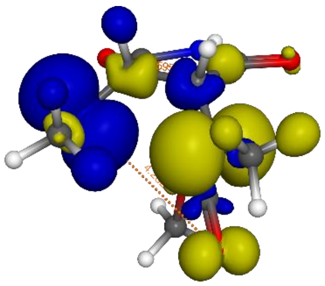	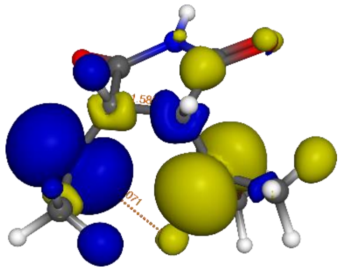	N/A
	SCF Energy: E(UB3LYP) = -744.477587781 A.U.<br>Zero-point correction= 0.220296<br>Thermal correction to Energy= 0.236815<br>Thermal correction to Enthalpy= 0.237759<br>Thermal correction to Gibbs Free Energy= 0.175068<br>Sum of electronic and zero-point Energies= -744.257291<br>Sum of electronic and thermal Energies= -744.240773<br>Sum of electronic and thermal Enthalpies= -744.239829<br>Sum of electronic and thermal Free Energies= -744.302520	SCF Energy: E(UB3LYP) = -744.460255895 A.U.<br>Zero-point correction= 0.219794<br>Thermal correction to Energy= 0.235629<br>Thermal correction to Enthalpy= 0.236574<br>Thermal correction to Gibbs Free Energy= 0.175978<br>Sum of electronic and zero-point Energies= -744.240462<br>Sum of electronic and thermal Energies= -744.224626<br>Sum of electronic and thermal Enthalpies= -744.223682<br>Sum of electronic and thermal Free Energies= -744.284278<br>Imaginary frequency: -68.2630 cm-1	SCF Energy: E(UB3LYP) = -744.545276483 A.U.<br>Zero-point correction= 0.227754<br>Thermal correction to Energy= 0.242019<br>Thermal correction to Enthalpy= 0.242963<br>Thermal correction to Gibbs Free Energy= 0.186649<br>Sum of electronic and zero-point Energies= -744.317522<br>Sum of electronic and thermal Energies= -744.303257<br>Sum of electronic and thermal Enthalpies= -744.302313<br>Sum of electronic and thermal Free Energies= -744.358627
	H -3.38300421 -1.65520372 1.63514572<br>C -2.56637427 -0.93070245 1.71241287<br>H -2.85592108 -0.16736748 2.45076612<br>C -2.23289698 -0.33263570 0.39193959<br>C 0.74440292 -0.41654044 1.31572218<br>C -1.36679947 0.86971952 0.28729636<br>C 0.09678015 0.87651375 0.92188568<br>C -1.15121121 1.32246407 -1.15937761<br>C 0.94251358 1.61760446 -0.14577695<br>O -1.94284365 1.27522651 -2.07385781<br>O 2.10353871 1.94371819 -0.04170358<br>N 0.13363727 1.85108222 -1.24464893<br>H 0.49927305 2.24023495 -2.10612799<br>C 0.88843071 -1.52816335 0.38455063	H -3.55651826 -1.38562298 1.95363775<br>C -2.63750845 -0.78992308 1.95550104<br>H -2.74804733 0.00739611 2.70467885<br>C -2.33570432 -0.25019897 0.60179471<br>C 0.72581418 -0.48054043 1.19631156<br>C -1.40878165 0.89989524 0.41989056<br>C 0.05256216 0.85174375 1.01776546<br>C -1.19718279 1.28903145 -1.04777386<br>C 0.87393071 1.67690184 -0.00847877<br>O -1.98728985 1.21435584 -1.96123517<br>O 1.99587925 2.11051658 0.13198153<br>N 0.09883675 1.79404999 -1.15341318<br>H 0.45546695 2.19914759 -2.01155942<br>C 0.59782584 -1.54922484 0.16047394	H -2.28978943 -2.72469879 0.89722387<br>C -2.07510968 -1.70109296 1.21966263<br>H -2.98432813 -1.28621780 1.66859020<br>C -1.64549094 -0.85764314 0.02077584<br>C 1.02124662 -0.36721353 0.83055576<br>C -1.31421485 0.59456705 0.37349390<br>C 0.06529633 0.79451700 1.04314508<br>C -1.29140088 1.49142236 -0.87212945<br>C 0.63245672 2.04818455 0.34793035<br>O -2.10071143 1.51372374 -1.77383374<br>O 1.61236766 2.69142492 0.65178543<br>N -0.17829905 2.30972183 -0.75788325<br>H 0.04360021 3.03061657 -1.43522282<br>C 0.68562169 -1.34494964 -0.03194679

O	1.38082444	-2.61404749	0.65559041	O	-0.22653776	-2.44021285	0.16833997	O	-0.52302285	-1.46136794	-0.65769563
O	0.40445868	-1.22761709	-0.85542796	O	1.55369286	-1.43243804	-0.79100356	O	1.56579312	-2.30086809	-0.40954279
C	0.50436750	-2.27819509	-1.82818420	C	1.53957908	-2.44626459	-1.81563090	C	1.04985858	-3.60840665	-0.68410847
H	0.05208191	-1.87850357	-2.73624904	H	2.36694069	-2.19842848	-2.48071126	H	1.92504991	-4.22152104	-0.90552863
C	1.39284250	-0.52928077	2.65519587	C	1.89495333	-0.56395058	2.13125958	C	2.34115321	-0.33291122	1.55264358
H	2.26361396	0.14322228	2.71019820	H	2.76052622	-0.01790309	1.72270633	H	2.94033822	0.53612341	1.25588887
H	-1.70108069	-1.46270822	2.15012835	H	-1.82702685	-1.45172550	2.30703862	H	-1.29444819	-1.73431590	1.98565996
H	-2.55458111	-0.80816418	-0.52743701	H	-2.67888024	-0.77356374	-0.28322701	H	-2.43427810	-0.85368496	-0.73596744
H	-1.87980321	1.71447065	0.78215897	H	-1.86159433	1.79749187	0.88244328	H	-2.11474685	0.98580459	1.01418822
H	0.10209552	1.52269825	1.80579036	H	0.10614749	1.38372073	1.97363488	H	-0.01407494	1.01644328	2.11483747
H	1.55151428	-2.54099609	-2.00117036	H	1.68145640	-3.43612710	-1.37446108	H	0.52720463	-4.01919535	0.18857918
H	-0.03262091	-3.16793764	-1.48884917	H	0.58982746	-2.42932583	-2.35628056	H	0.37412887	-3.60017585	-1.54275258
H	0.70983974	-0.22260180	3.45920188	H	1.65550974	-0.10407688	3.09830359	H	2.18930794	-0.25230691	2.63900774
H	1.73387291	-1.54997460	2.83740815	H	2.20217797	-1.59979895	2.30862067	H	2.91930997	-1.23722291	1.35206475

Table S6d Molecular structure plots, computed energies (excerpts from the *Gaussian* output) and atomic coordinates (in [Å]) for the intrinsic reaction coordinate **TS4** (for details, see also Table S6a).

	**starting geometry**	**transition state**	**product geometry**
**TS4**	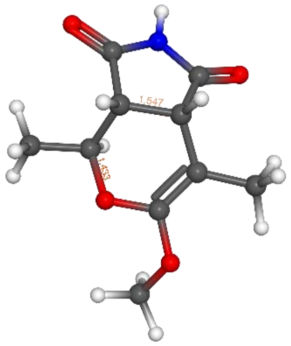	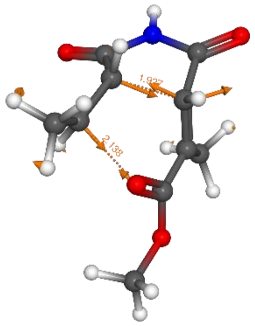	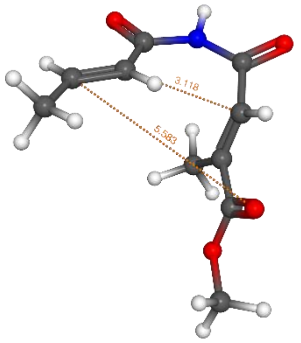
	SCF Energy: E(UB3LYP) = -744.546606022 A.U.<br>Zero-point correction= 0.227569<br>Thermal correction to Energy= 0.241914<br>Thermal correction to Enthalpy= 0.242858<br>Thermal correction to Gibbs Free Energy= 0.186286<br>Sum of electronic and zero-point Energies= -744.319037<br>Sum of electronic and thermal Energies= -744.304692<br>Sum of electronic and thermal Enthalpies= -744.303748<br>Sum of electronic and thermal Free Energies= -744.360320	SCF Energy: E(UB3LYP) = -744.477243700 A.U.<br>Zero-point correction= 0.222172<br>Thermal correction to Energy= 0.237437<br>Thermal correction to Enthalpy= 0.238381<br>Thermal correction to Gibbs Free Energy= 0.179556<br>Sum of electronic and zero-point Energies= -744.255072<br>Sum of electronic and thermal Energies= -744.239807<br>Sum of electronic and thermal Enthalpies= -744.238862<br>Sum of electronic and thermal Free Energies= -744.297688<br>Imaginary frequency: -501.0658 cm-1	SCF Energy: E(UB3LYP) = -744.532820836 A.U.<br>Zero-point correction= 0.222883<br>Thermal correction to Energy= 0.239647<br>Thermal correction to Enthalpy= 0.240591<br>Thermal correction to Gibbs Free Energy= 0.176320<br>Sum of electronic and zero-point Energies= -744.309938<br>Sum of electronic and thermal Energies= -744.293174<br>Sum of electronic and thermal Enthalpies= -744.292230<br>Sum of electronic and thermal Free Energies= -744.356501

H	-0.71657758	-1.02485979	-3.22988118	H	-0.37882307	-1.46987461	-3.24083547	H	-1.69607311	-1.22805918	-3.11381838
C	-1.47394675	-0.93591547	-2.44394449	C	-1.32727703	-1.05960093	-2.87890292	C	-2.51937639	-1.71434570	-2.58164710
H	-1.85265108	-1.93459649	-2.21353985	H	-2.10298367	-1.82559814	-3.02625147	H	-2.80793814	-2.61620371	-3.13802802
C	-0.86883067	-0.30046542	-1.20338226	C	-1.24220079	-0.69823695	-1.43230834	C	-2.15616391	-2.06155460	-1.17364591
C	0.83479421	1.16575031	0.47872982	C	0.75450483	0.98317172	0.35269021	C	0.43186705	1.15004084	0.27319072
C	0.94874195	-0.33733654	0.57791757	C	1.09866661	-0.36638097	0.05443803	C	1.32171601	0.14603764	0.18285341
C	0.28231248	-1.12192278	-0.57679039	C	-0.42083908	-1.39891873	-0.52816186	C	-0.96929945	-1.81821234	-0.59955632
C	0.35865887	-0.97016682	1.85271110	C	1.44448001	-1.24628276	1.24241687	C	1.50851397	-0.96818527	1.15815253
C	-0.27986821	-2.38253582	0.08327957	C	-0.85968627	-1.75773542	0.86963388	C	-0.72255901	-2.25267054	0.79436747
O	0.42059482	-0.58405867	2.99851784	O	2.52442576	-1.35093760	1.78401350	O	2.56428084	-1.06084936	1.76418078
O	-0.71966932	-3.37741325	-0.45404918	O	-1.98904852	-1.96030387	1.26269700	O	-1.48391820	-2.97636054	1.41861132
N	-0.25090945	-2.16201054	1.45466702	N	0.27964451	-1.87796616	1.67662827	N	0.48325771	-1.87593578	1.42376208
H	-0.64181906	-2.81998228	2.11963540	H	0.23292333	-2.31904172	2.58858617	H	0.67097602	-2.43865922	2.24970116
C	0.26375610	1.69889764	-0.61623112	C	0.16843197	1.66220482	-0.73393332	C	0.57853858	2.23119458	-0.76112155
O	-0.37940320	0.98925827	-1.59155828	O	-0.10542151	1.07100294	-1.81567152	O	1.38464400	2.22675028	-1.67019726
O	0.33747525	3.01911651	-0.90420657	O	-0.23750397	2.94395677	-0.51290515	O	-0.31427459	3.22588539	-0.56813188
C	-0.84329666	3.63477585	-1.43355106	C	-0.88201803	3.59374795	-1.61085664	C	-0.24442512	4.30552580	-1.51463004
H	-0.59895549	4.69358802	-1.53423214	H	-1.08202668	4.61119406	-1.27113156	H	-1.01873184	5.01023894	-1.21090340
C	1.52450966	2.00773872	1.51998137	C	0.68347035	1.54070663	1.75315698	C	-0.65721509	1.28446213	1.30362048
H	2.61097521	1.83305696	1.51155630	H	1.47237033	1.12775314	2.38841991	H	-0.61652192	0.48332292	2.04371905
H	-2.29330499	-0.31708570	-2.82232548	H	-1.58816169	-0.19097248	-3.48854559	H	-3.39133397	-1.04692259	-2.60402523
H	-1.63945430	-0.14411102	-0.43254519	H	-2.05990418	-0.11058753	-1.02398121	H	-2.91453048	-2.55612218	-0.56801920
H	2.01848799	-0.60700096	0.61570701	H	1.68447173	-0.52756360	-0.84737263	H	2.09617235	0.21670697	-0.57690936
H	0.98697894	-1.39857448	-1.36639990	H	0.14176819	-2.22107148	-0.97688495	H	-0.17383781	-1.32923294	-1.15057733
H	-1.68875351	3.51818321	-0.74393409	H	-1.82074531	3.09497315	-1.87197510	H	-0.43068808	3.93883265	-2.52746479
H	-1.10748619	3.21988635	-2.40951959	H	-0.23546668	3.61007117	-2.49388241	H	0.74135094	4.77665412	-1.48367586
H	1.17105114	1.75772575	2.52527028	H	-0.28275440	1.33836144	2.24074306	H	-1.64510757	1.26756639	0.82869979
H	1.35687390	3.07048624	1.33436961	H	0.80191929	2.62663196	1.72679847	H	-0.57534899	2.24465324	1.82318101

Table S6e Molecular structure plots, computed energies (excerpts from the *Gaussian* output) and atomic coordinates (in [Å]) for the intrinsic reaction coordinate **TS5** (for details, see also Table S6a).

	**starting geometry**	**transition state**	**product geometry**
**TS5**	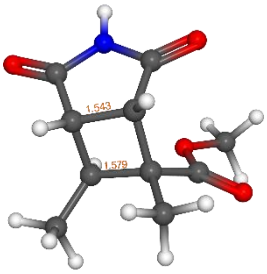	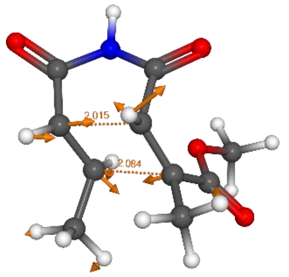	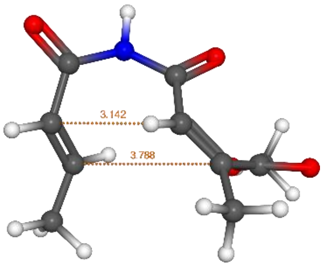
	SCF Energy: E(UB3LYP) = -744.556829396 A.U.<br>Zero-point correction= 0.226822<br>Thermal correction to Energy= 0.241366<br>Thermal correction to Enthalpy= 0.242310<br>Thermal correction to Gibbs Free Energy= 0.185168<br>Sum of electronic and zero-point Energies= -744.330007<br>Sum of electronic and thermal Energies= -744.315463<br>Sum of electronic and thermal Enthalpies= -744.314519<br>Sum of electronic and thermal Free Energies= -744.371661	SCF Energy: E(RB3LYP) = -744.434801047 A.U.<br>Zero-point correction= 0.222042<br>Thermal correction to Energy= 0.237158<br>Thermal correction to Enthalpy= 0.238103<br>Thermal correction to Gibbs Free Energy= 0.180256<br>Sum of electronic and zero-point Energies= -744.212759<br>Sum of electronic and thermal Energies= -744.197643<br>Sum of electronic and thermal Enthalpies= -744.196698<br>Sum of electronic and thermal Free Energies= -744.254545<br>Imaginary frequency: -752.4514 cm-1	SCF Energy: E(UB3LYP) = -744.528241667 A.U.<br>Zero-point correction= 0.222571<br>Thermal correction to Energy= 0.239314<br>Thermal correction to Enthalpy= 0.240258<br>Thermal correction to Gibbs Free Energy= 0.176551<br>Sum of electronic and zero-point Energies= -744.305671<br>Sum of electronic and thermal Energies= -744.288928<br>Sum of electronic and thermal Enthalpies= -744.287984<br>Sum of electronic and thermal Free Energies= -744.351691

C	-2.68699070	-0.35643341	1.54852479	C	-2.48706827	-0.16228908	1.47195537	C	-2.61300810	-1.09616816	2.34750324
C	-1.32453839	-0.33370621	0.86966489	C	-1.14844940	-0.52238406	0.88531965	C	-1.41037340	-1.42489174	1.51859998
C	-1.00524406	0.77164418	-0.21225110	C	-0.93060288	1.04459433	-0.47152527	C	-0.67195768	1.42868648	-0.86138260
C	-1.02501705	-1.44706441	-0.19443253	C	-1.03697562	-1.82196019	0.24526460	C	-1.35382553	-2.41901715	0.62022820
C	-0.43736228	-0.37576209	-1.13638735	C	-0.50102476	-0.26757205	-0.92013226	C	-0.44861189	0.11275803	-1.00612279
C	0.12427812	-2.37300861	0.16353621	C	0.19039749	-2.59447590	0.12225469	C	-0.13331611	-2.84767736	-0.10174645
C	1.06875598	-0.63198447	-1.12770686	C	0.97996292	-0.63810903	-1.02851133	C	0.85856722	-0.53589386	-0.71129032
O	0.10409115	-3.38685247	0.82651838	O	0.31637014	-3.79916917	0.26870349	O	0.06701496	-4.02381655	-0.36068985
O	1.93907676	0.01865018	-1.66395518	O	1.75706567	0.00230995	-1.70341067	O	1.92610642	0.04973898	-0.81756730
N	1.27129778	-1.80347767	-0.39893823	N	1.26704950	-1.78487763	-0.32355186	N	0.86492467	-1.90292800	-0.41092548
H	2.19485625	-2.19569166	-0.25630469	H	2.17940071	-2.21408275	-0.43932077	H	1.77268234	-2.33108831	-0.56949494
C	0.00491991	1.84595380	0.18030328	C	-0.06344397	1.99677840	0.21133688	C	0.34530179	2.35434114	-0.25141010
O	-0.02766904	2.99745179	-0.19462998	O	-0.28916808	3.19463821	0.28965578	O	0.71360114	3.37846623	-0.78359940
O	0.97107243	1.35513864	0.98808153	O	1.02647895	1.41956620	0.80728820	O	0.72105652	1.95381070	0.97891695
C	2.04768985	2.26206820	1.28641921	C	1.95706948	2.33598437	1.40102101	C	1.77600342	2.72847492	1.57750200
H	2.70407300	1.72104697	1.96834050	H	2.75604504	1.71672108	1.81070925	H	1.90005259	2.32516260	2.58273729
C	-2.22725421	1.43024089	-0.85384674	C	-2.18147006	1.62219613	-1.08975294	C	-1.92333942	2.10938530	-1.34336364
H	-1.91421803	2.05690536	-1.69302784	H	-1.95771390	2.09352795	-2.05768439	H	-1.65565001	2.91592322	-2.03526411
H	-0.54366006	-0.33106966	1.63373216	H	-0.28418735	-0.20522841	1.46152399	H	-0.53299135	-0.80241833	1.68605387
H	-1.90257688	-2.00363288	-0.52931112	H	-1.95743755	-2.34370667	0.00122228	H	-2.19777553	-3.09031032	0.47931015
H	-0.78544835	-0.32297284	-2.17180755	H	-1.08317685	-0.65317591	-1.75661189	H	-1.22481271	-0.51205812	-1.43891272
H	2.57795719	2.52415388	0.36777421	H	2.35311847	3.01933486	0.64566910	H	2.69519411	2.60679671	0.99886222
H	1.66321490	3.17005630	1.75675583	H	1.47652465	2.91884349	2.19123376	H	1.50429194	3.78611506	1.61369609
H	-2.93151448	0.67759749	-1.22283037	H	-2.93855814	0.85033939	-1.27191932	H	-2.59288029	1.40907961	-1.84982338
H	-2.75167789	2.07204501	-0.13923788	H	-2.61612173	2.39478702	-0.45119519	H	-2.46739249	2.57474448	-0.51102829
H	-3.51092053	-0.38014427	0.82763908	H	-3.30364326	-0.30976253	0.75951865	H	-3.45835197	-1.75385044	2.12351967
H	-2.77077814	-1.24756669	2.18107996	H	-2.65421666	-0.85926216	2.30688789	H	-2.38187375	-1.17963630	3.41759105
H	-2.82369915	0.52215147	2.18987099	H	-2.51640447	0.86288036	1.84646345	H	-2.92376405	-0.05668064	2.17704413

Table S7a Molecular structure plots of the extended polymer models, computed energies (excerpts from the *Gaussian* output) and atomic coordinates (in [Å]) for the intrinsic reaction coordinate **TS1** (starting geometry, transition state, and final product geometry). For further explanations, see also Table S6a.

	**starting geometry**	**transition state**	**product geometry**
**TS1**	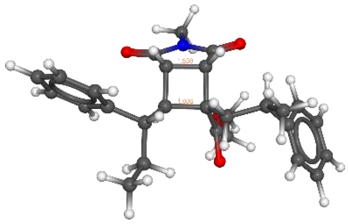	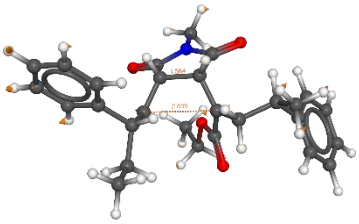	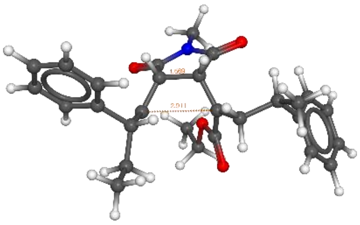
	N/A	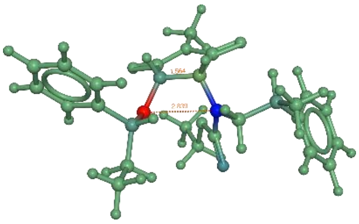	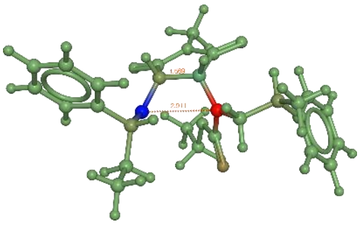
	N/A	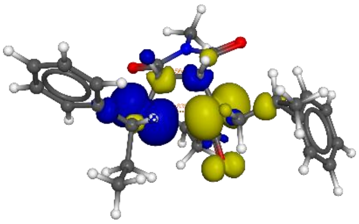	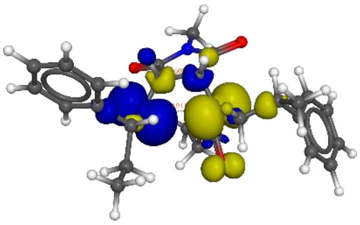
	SCF Energy: E(UB3LYP) = -1442.53621104 A.U.<br>Zero-point correction= 0.560345<br>Thermal correction to Energy= 0.592516<br>Thermal correction to Enthalpy= 0.593460<br>Thermal correction to Gibbs Free Energy= 0.494058<br>Sum of electronic and zero-point Energies= -1441.975866<br>Sum of electronic and thermal Energies= -1441.943695<br>Sum of electronic and thermal Enthalpies= -1441.942751<br>Sum of electronic and thermal Free Energies= -1442.042153	SCF Energy: E(UB3LYP) = -1442.46633114 A.U.<br>Zero-point correction= 0.555126<br>Thermal correction to Energy= 0.587840<br>Thermal correction to Enthalpy= 0.588784<br>Thermal correction to Gibbs Free Energy= 0.486931<br>Sum of electronic and zero-point Energies= -1441.911205<br>Sum of electronic and thermal Energies= -1441.878492<br>Sum of electronic and thermal Enthalpies= -1441.877547<br>Sum of electronic and thermal Free Energies= -1441.979400<br>Imaginary frequency: -22.1592 cm-1	SCF Energy: E(UB3LYP) = -1442.46634637 A.U.<br>Zero-point correction= 0.555153<br>Thermal correction to Energy= 0.588749<br>Thermal correction to Enthalpy= 0.589693<br>Thermal correction to Gibbs Free Energy= 0.484252<br>Sum of electronic and zero-point Energies= -1441.911193<br>Sum of electronic and thermal Energies= -1441.877597<br>Sum of electronic and thermal Enthalpies= -1441.876653<br>Sum of electronic and thermal Free Energies= -1441.982095

C	-3.94656884	0.76957415	2.44754655	C	-3.97596819	0.36925790	2.91695075	C	-3.88259318	0.37148645	2.99840001
C	-2.48397115	0.40656334	2.17666748	C	-2.50080508	-0.01412456	2.77587122	C	-2.41784690	-0.04637117	2.84560568
C	-2.29608668	-0.63466963	1.04349551	C	-2.20531958	-0.96782582	1.59295412	C	-2.15142896	-0.98614829	1.64486585
C	-2.86590233	-1.99816610	1.41978097	C	-2.92458320	-2.31658745	1.71923613	C	-2.91866557	-2.31119311	1.73755469
C	-4.03059767	-2.47109390	0.79933051	C	-3.92798550	-2.68062067	0.81239761	C	-3.89493562	-2.64062755	0.78880134
C	-2.26162367	-2.80654707	2.39444164	C	-2.58345639	-3.22386994	2.73447080	C	-2.64584416	-3.23416150	2.75931325
C	-4.58370223	-3.70603617	1.14406838	C	-4.58232646	-3.91046743	0.91805299	C	-4.58799414	-3.85167992	0.85876486
C	-2.80726520	-4.04287065	2.73824057	C	-3.23467395	-4.45160301	2.84379621	C	-3.33654889	-4.44275199	2.83391006
C	-3.97252462	-4.49670265	2.11700823	C	-4.23769628	-4.79992572	1.93545744	C	-4.31115345	-4.75674837	1.88291848
C	-0.80720682	-0.72091404	0.64743008	C	-0.72274913	-1.16664994	1.43307289	C	-0.67687583	-1.24004693	1.48615580
C	-0.16858349	0.39563090	-0.31361996	C	-0.03242242	0.79803935	-0.49701177	C	-0.05473809	0.78468123	-0.51105958
C	-0.36756914	-1.81651050	-0.36930804	C	-0.13100952	-1.69153513	0.15886563	C	-0.09570992	-1.71576808	0.19143154
C	0.29467117	-0.76780065	-1.27862589	C	0.07278087	-0.63494910	-0.97582722	C	0.05488956	-0.65568480	-0.95579700
C	0.77061708	-2.71886347	0.08682812	C	1.24801169	-2.32866022	0.32460557	C	1.30225522	-2.31764215	0.32540785
C	1.74888721	-1.19168489	-1.40446937	C	1.43929337	-1.00858393	-1.58168443	C	1.41271421	-1.01113930	-1.59564638
O	0.73005652	-3.63009344	0.88856047	O	1.62901602	-3.03488518	1.23518126	O	1.72237634	-3.01178954	1.22804771
O	2.61422698	-0.72491457	-2.11851203	O	1.94757684	-0.54715903	-2.58406117	O	1.88036783	-0.55224527	-2.61886091
N	1.92591564	-2.30939754	-0.58821014	N	2.00960414	-1.99788378	-0.79680681	N	2.02880531	-1.97443394	-0.81487240
C	3.21047121	-2.97066508	-0.43929759	C	3.35516464	-2.49743143	-1.02710575	C	3.37932786	-2.44493387	-1.07655884
C	0.82884072	1.19696759	0.51629513	C	0.80611024	1.34054871	0.56998402	C	0.75950833	1.33770605	0.56984842
O	0.49688422	2.14932398	1.19285708	O	0.60426746	2.41040909	1.12696927	O	0.52810036	2.39915806	1.13152771
O	2.06765880	0.67815188	0.52401932	O	1.84767531	0.52454811	0.89478324	O	1.81334754	0.54141978	0.90006934
C	3.05802559	1.40380996	1.29751548	C	2.67925504	0.97450351	1.98835902	C	2.62395126	0.99818166	2.00688388
C	4.37555010	0.66679519	1.15489081	C	3.80145885	-0.03241023	2.15973032	C	3.77884900	0.02679421	2.16497339
C	-1.14511788	1.35186042	-1.02706665	C	-1.01463387	1.71346277	-1.15399184	C	-1.02416132	1.69207841	-1.19772020
C	-0.56898622	2.11243864	-2.25361327	C	-0.52916825	2.26124305	-2.53943183	C	-0.50843610	2.23624253	-2.57364397
C	0.55291856	3.09187595	-1.92403742	C	0.65513077	3.21087806	-2.40302052	C	0.66937796	3.19044344	-2.41400632
C	0.29787513	4.31873711	-1.29353771	C	0.51783487	4.46567940	-1.79114527	C	0.51750045	4.44071192	-1.79619256
C	1.87710205	2.78251801	-2.26414721	C	1.91286244	2.84884205	-2.90296359	C	1.93549419	2.83864962	-2.90010879
C	2.91666504	3.67455371	-1.98462376	C	3.00069408	3.71968210	-2.80381002	C	3.01703474	3.71493352	-2.78194158
C	1.33161457	5.21068907	-1.01460030	C	1.60172797	5.33665839	-1.69066057	C	1.59515894	5.31701511	-1.67636446
C	2.64731885	4.89346603	-1.36208963	C	2.84876043	4.96760229	-2.20002876	C	2.85061107	4.95810773	-2.17214747

C	-1.72154570	2.79493737	-3.01714116	C	-1.71003372	2.91067974	-3.28418799	C	-1.67353092	2.88087524	-3.34690190
H	-4.02140855	1.50035381	3.26042596	H	-4.11965300	1.05563067	3.75869421	H	-4.00649038	1.04727699	3.85178238
H	-4.41657166	1.21260951	1.56028355	H	-4.34249011	0.87267118	2.01355984	H	-4.23923486	0.89860999	2.10466888
H	-4.53349198	-0.11074225	2.73058781	H	-4.60615023	-0.51006764	3.08767719	H	-4.53374624	-0.49478122	3.15669340
H	-2.02833511	0.00730338	3.09330448	H	-2.14928723	-0.48372027	3.70499643	H	-2.07500928	-0.53946477	3.76577627
H	-1.91674076	1.31127286	1.92794357	H	-1.89381277	0.88999104	2.63793979	H	-1.78904616	0.84439204	2.71982660
H	-2.86836600	-0.28695389	0.17425697	H	-2.59669017	-0.49128501	0.68036776	H	-2.51979036	-0.47544078	0.74155666
H	-4.51421190	-1.86076877	0.03920228	H	-4.20517277	-1.99030736	0.01818283	H	-4.12111116	-1.93738690	-0.01020072
H	-1.34726405	-2.47986145	2.88135163	H	-1.79579244	-2.97087680	3.43951651	H	-1.88161891	-3.00772713	3.49839248
H	-5.48958478	-4.04835761	0.65025747	H	-5.36030811	-4.17077440	0.20486362	H	-5.34386075	-4.08470288	0.11307589
H	-2.31563008	-4.65582572	3.48881006	H	-2.95541764	-5.14037193	3.63686335	H	-3.11061813	-5.14347674	3.63349330
H	-4.39642252	-5.46051655	2.38598646	H	-4.74337667	-5.75806429	2.01987581	H	-4.84756060	-5.70005312	1.94034745
H	-0.21833964	-0.79798477	1.56859948	H	-0.10094450	-1.18430871	2.32387557	H	-0.06581159	-1.34388830	2.37860907
H	-1.18284986	-2.41733919	-0.77997491	H	-0.77558873	-2.49647171	-0.23251718	H	-0.72309400	-2.53538263	-0.20064051
H	-0.11689704	-0.65386107	-2.28346407	H	-0.66522435	-0.77127043	-1.77264275	H	-0.70019758	-0.81782209	-1.73157149
H	3.06180569	-3.83835076	0.20465428	H	3.53151527	-3.31115919	-0.32285999	H	3.58642601	-3.26114972	-0.38370134
H	3.58556866	-3.28142520	-1.41812530	H	3.44402203	-2.85345006	-2.05621521	H	3.45352987	-2.78924170	-2.11066745
H	3.93662149	-2.28843308	0.01215907	H	4.08884528	-1.70186619	-0.86490413	H	4.10044975	-1.63649303	-0.92134741
H	3.11166609	2.42233066	0.90315250	H	3.05214337	1.97671225	1.75712330	H	2.96324243	2.01675460	1.79676087
H	2.71975113	1.45881291	2.33665334	H	2.05895836	1.05213384	2.88801439	H	1.99631964	1.03671038	2.90400785
H	4.65709979	0.58121056	0.10085208	H	4.43506270	-0.06723028	1.26700412	H	4.41619086	0.02961862	1.27417706
H	5.16389825	1.21305674	1.68429139	H	4.42862443	0.25524707	3.01074138	H	4.39303039	0.32037672	3.02341566
H	4.31315759	-0.33979155	1.58222708	H	3.40446732	-1.03582812	2.34313190	H	3.41562942	-0.99256655	2.32893118
H	-1.99815970	0.76589704	-1.39015913	H	-1.95632268	1.17108328	-1.32579184	H	-1.95914895	1.14505100	-1.38915495
H	-1.53381976	2.07155075	-0.30007659	H	-1.22581288	2.55476273	-0.48818123	H	-1.25496388	2.53570470	-0.54111685
H	-0.13856927	1.36610260	-2.93380489	H	-0.19095872	1.40210769	-3.13238181	H	-0.15467341	1.37595924	-3.15507260
H	-0.71747640	4.57980585	-1.00698815	H	-0.44327198	4.76775571	-1.38247418	H	-0.45049043	4.73503890	-1.39824884
H	2.10305568	1.82753654	-2.73265808	H	2.04337785	1.87053312	-3.35668692	H	2.07645310	1.86453934	-3.35959264
H	3.93520704	3.41467407	-2.26212784	H	3.96745177	3.41845016	-3.19962098	H	3.99034625	3.42188017	-3.16777961
H	1.11047763	6.15502361	-0.52381468	H	1.47288922	6.30370693	-1.21122459	H	1.45476926	6.28029843	-1.19261773
H	3.45289086	5.59166108	-1.14955418	H	3.69387920	5.64686133	-2.12275300	H	3.69087356	5.64152004	-2.08000128
H	-2.25277744	3.51812822	-2.38785817	H	-2.13172569	3.74876255	-2.71766253	H	-2.10871436	3.72119340	-2.79389202
H	-1.34393748	3.32952785	-3.89477031	H	-1.38783513	3.29455138	-4.25777753	H	-1.33019597	3.26072938	-4.31484286
H	-2.45429336	2.05346209	-3.35751877	H	-2.51376362	2.18358018	-3.45201074	H	-2.47240411	2.15196102	-3.52946901

Table S7b Molecular structure plots, computed energies (excerpts from the *Gaussian* output) and atomic coordinates (in [Å]) for the intrinsic reaction coordinate **TS2** (for details, see also Table S7a).

	**starting geometry**	**transition state**	**product geometry**
**TS2**	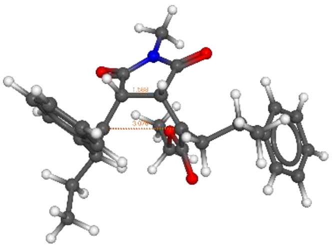	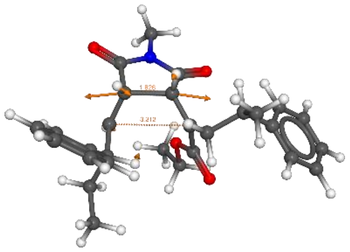	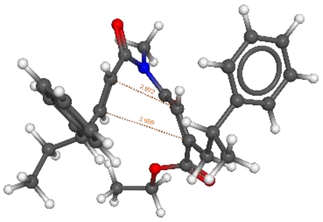
	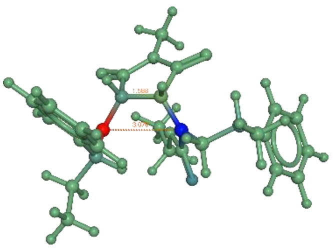	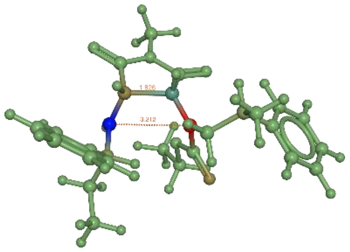	N/A
	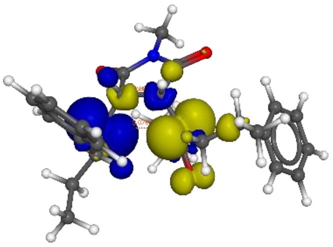	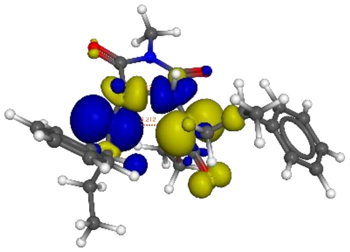	N/A
	SCF Energy: E(UB3LYP) = -1442.46611609 A.U.<br>Zero-point correction= 0.554673<br>Thermal correction to Energy= 0.588399<br>Thermal correction to Enthalpy= 0.589343<br>Thermal correction to Gibbs Free Energy= 0.484440<br>Sum of electronic and zero-point Energies= -1441.911443<br>Sum of electronic and thermal Energies= -1441.877717<br>Sum of electronic and thermal Enthalpies= -1441.876773<br>Sum of electronic and thermal Free Energies= -1441.981676	SCF Energy: E(UB3LYP) = -1442.46085342 A.U.<br>Zero-point correction= 0.553786<br>Thermal correction to Energy= 0.586915<br>Thermal correction to Enthalpy= 0.587859<br>Thermal correction to Gibbs Free Energy= 0.484974<br>Sum of electronic and zero-point Energies= -1441.907067<br>Sum of electronic and thermal Energies= -1441.873938<br>Sum of electronic and thermal Enthalpies= -1441.872994<br>Sum of electronic and thermal Free Energies= -1441.975879<br>Imaginary frequency: -601.6774 cm-1	SCF Energy: E(UB3LYP) = -1442.50422099 A.U.<br>Zero-point correction= 0.556227<br>Thermal correction to Energy= 0.590241<br>Thermal correction to Enthalpy= 0.591185<br>Thermal correction to Gibbs Free Energy= 0.483792<br>Sum of electronic and zero-point Energies= -1441.947994<br>Sum of electronic and thermal Energies= -1441.913980<br>Sum of electronic and thermal Enthalpies= -1441.913036<br>Sum of electronic and thermal Free Energies= -1442.020429

C	-3.36496618	-0.06373346	3.99316726	C	-2.45492807	0.06462210	4.07011309	C	-2.77676366	-1.97903238	4.17502154
C	-2.02475330	-0.73806646	3.69181622	C	-1.35906933	-0.89836508	3.60791175	C	-1.50372846	-2.25136719	3.36922821
C	-1.75466962	-0.97254439	2.18338908	C	-1.33813098	-1.15020029	2.07926681	C	-1.49834136	-1.58144767	1.96707934
C	-2.78363373	-1.91217931	1.55141343	C	-2.62162630	-1.80754237	1.57601648	C	-2.62536033	-2.06112733	1.06518354
C	-3.76025744	-1.42838846	0.67178230	C	-3.59797573	-1.06269292	0.90224678	C	-3.52017578	-1.14124310	0.50327189
C	-2.77971831	-3.28358143	1.85115516	C	-2.86245227	-3.17069682	1.80695124	C	-2.80136389	-3.42178141	0.76970252
C	-4.70904793	-2.28488920	0.10750508	C	-4.78580626	-1.65924786	0.47223899	C	-4.55942588	-1.56079622	-0.32973332
C	-3.72412711	-4.14236730	1.28934379	C	-4.04660200	-3.76935687	1.37783057	C	-3.83802390	-3.84582007	-0.06212555
C	-4.69338467	-3.64545912	0.41416001	C	-5.01362299	-3.01482560	0.70904841	C	-4.72157121	-2.91649334	-0.61532347
C	-0.34161008	-1.43286178	1.96553128	C	-0.09453703	-1.85297537	1.64833579	C	-0.11810000	-1.67646863	1.35410735
C	-0.07262772	0.58233747	-0.34233398	C	0.05836412	0.53835821	-0.49028690	C	0.67803830	1.77254004	0.27979646
C	0.16958710	-1.86927498	0.63680468	C	0.13220990	-2.17677576	0.28064553	C	0.20624879	-2.15437017	0.14487174
C	0.13624341	-0.88215761	-0.60727861	C	0.57738907	-0.76537678	-0.78858097	C	1.17540195	0.63138600	-0.22156071
C	1.61823817	-2.34841270	0.69138030	C	1.35717464	-3.04744364	0.05323723	C	1.59808775	-2.31617183	-0.34636523
C	1.47604047	-1.18358668	-1.31592730	C	2.08614602	-0.99904159	-0.86344434	C	2.41382211	-0.01724057	0.29852604
O	2.15964776	-2.95079421	1.59585854	O	1.44717883	-4.23002965	0.31737763	O	1.91014044	-3.30050567	-1.00090321
O	1.82556897	-0.77770996	-2.40660084	O	2.89800041	-0.22615653	-1.32876436	O	3.30518060	0.63357059	0.82719170
N	2.23117991	-2.02297051	-0.51691557	N	2.40306531	-2.29605707	-0.46337542	N	2.59046266	-1.39197386	0.04594872
C	3.59230002	-2.40775852	-0.85455931	C	3.72966381	-2.86968134	-0.64760718	C	3.97044202	-1.90140207	0.02030885
C	0.72802705	1.31460246	0.63511537	C	0.60639110	1.35555842	0.58965066	C	1.25073381	2.41568925	1.51482251
O	0.45147031	2.42471806	1.06675449	O	0.25986268	2.49369190	0.86642814	O	1.58690313	3.57980778	1.56292797
O	1.82603128	0.61654026	1.03927609	O	1.55482327	0.67928369	1.30690970	O	1.24877846	1.58111644	2.57401837
C	2.64923174	1.25918332	2.03941647	C	2.21363792	1.44044482	2.34100006	C	1.89218433	2.08962515	3.76754005
C	3.87283255	0.38907595	2.25867844	C	3.33890573	0.58719939	2.89631865	C	1.83424242	0.99688005	4.81758215
C	-1.12313489	1.33050886	-1.10062521	C	-1.07015562	1.10666492	-1.30582244	C	-0.46536152	2.56722447	-0.31082466
C	-0.67567896	1.77436879	-2.53524491	C	-0.63889946	1.64783407	-2.70830937	C	-1.14395479	2.04187403	-1.59040539
C	0.41116803	2.84174868	-2.50268815	C	0.26033406	2.87350688	-2.60435158	C	-0.24503033	1.99645947	-2.82223006
C	0.16153427	4.12422003	-1.99194208	C	-0.26211764	4.14961036	-2.34987175	C	0.61082474	3.05494275	-3.15772373

C	1.68917358	2.56445644	-3.00583694	C	1.64745323	2.74305887	-2.75215958	C	-0.30893371	0.89752333	-3.69066043
C	2.68667161	3.54257745	-3.00733273	C	2.48761117	3.85412810	-2.65672714	C	0.45969827	0.85050555	-4.85480922
C	1.15481066	5.10241406	-1.99215097	C	0.57351314	5.26208189	-2.25711549	C	1.38187011	3.01208071	-4.31979865
C	2.42259369	4.81585143	-2.50331260	C	1.95345582	5.11938880	-2.41377886	C	1.30994725	1.90978808	-5.17338495
C	-1.90492427	2.22849762	-3.34397051	C	-1.88385405	1.88865725	-3.58012938	C	-2.40052414	2.88947076	-1.88482498
H	-3.48886583	0.08785623	5.07118175	H	-2.40642987	0.21300525	5.15448696	H	-2.72037483	-2.45237033	5.16146763
H	-3.43075232	0.91892024	3.51002797	H	-2.34427399	1.04650243	3.59395922	H	-2.92416730	-0.90283807	4.32912824
H	-4.20790539	-0.66712218	3.63997421	H	-3.45277335	-0.31530994	3.82770833	H	-3.66442205	-2.36753964	3.66541433
H	-1.96458876	-1.70113245	4.21609668	H	-1.46677166	-1.86239311	4.12243599	H	-1.35602281	-3.33260516	3.25470152
H	-1.20823187	-0.11848832	4.08596849	H	-0.37832196	-0.49752760	3.89742685	H	-0.63240303	-1.88269957	3.92689197
H	-1.87696399	0.00739513	1.68358669	H	-1.27992179	-0.15409836	1.57863601	H	-1.65445168	-0.50450792	2.14569400
H	-3.78277707	-0.36731259	0.43252995	H	-3.42843480	-0.00473773	0.71648365	H	-3.40499408	-0.08250483	0.72645057
H	-2.02529434	-3.67912015	2.52771195	H	-2.10922999	-3.76732796	2.31598656	H	-2.11878439	-4.15729601	1.18732676
H	-5.45914455	-1.88694123	-0.57117101	H	-5.53042754	-1.06296384	-0.04865557	H	-5.24259888	-0.82801103	-0.75117232
H	-3.70334012	-5.20117415	1.53451297	H	-4.21294694	-4.82728902	1.56326421	H	-3.95465666	-4.90439253	-0.27888271
H	-5.42875291	-4.31440436	-0.02469777	H	-5.93567704	-3.48180753	0.37353179	H	-5.52951443	-3.24754742	-1.26211826
H	0.35235444	-1.44450277	2.79990075	H	0.74758938	-1.86305661	2.33562172	H	0.67068527	-1.28461475	1.99510703
H	-0.41498871	-2.74644237	0.31008721	H	-0.75506113	-2.55548778	-0.22920234	H	-0.54005013	-2.60034553	-0.50563127
H	-0.64862061	-1.20031150	-1.30078596	H	0.15918962	-1.13394716	-1.73230409	H	0.73488551	0.19907713	-1.11243222
H	3.90928681	-3.16766961	-0.13964970	H	3.65922063	-3.77829283	-1.25103368	H	4.31868438	-2.00841747	-1.01137580
H	3.61864777	-2.80053767	-1.87345345	H	4.34519136	-2.12281962	-1.14871378	H	4.59591395	-1.19227639	0.55802739
H	4.25946180	-1.54231288	-0.79286070	H	4.16676828	-3.12791159	0.32108849	H	4.00440646	-2.88201361	0.49801344
H	2.90998041	2.26149813	1.68712364	H	2.58384532	2.37308670	1.90514004	H	2.92121124	2.35858226	3.51132101
H	2.05827849	1.37374291	2.95477958	H	1.47923313	1.70581525	3.10958869	H	1.37433703	2.99927653	4.08773922
H	4.46915088	0.31748520	1.34276479	H	4.05197995	0.32685671	2.10772134	H	2.36206989	0.10099144	4.47517051
H	4.50190461	0.82740352	3.04122351	H	3.87352443	1.13894945	3.67732119	H	2.31023949	1.34366582	5.74128667
H	3.58875876	-0.62227076	2.56590179	H	2.95527480	-0.33972484	3.33665442	H	0.79818255	0.72575947	5.04754356
H	-2.00798887	0.68858415	-1.21646536	H	-1.82409332	0.32294539	-1.47294884	H	-1.23874685	2.66666086	0.46659301
H	-1.41902364	2.21517090	-0.52879184	H	-1.54960813	1.91738561	-0.74863716	H	-0.09820336	3.59169358	-0.45804450
H	-0.25220373	0.89334371	-3.03187230	H	-0.05030681	0.85583964	-3.19164368	H	-1.48421560	1.01595346	-1.39133458
H	-0.81675549	4.36469100	-1.58320609	H	-1.33292297	4.28197848	-2.21844181	H	0.68526874	3.92262422	-2.50745558
H	1.90541362	1.56948100	-3.38412730	H	2.07868667	1.75977785	-2.91909747	H	-0.97006116	0.06673677	-3.45055105
H	3.67099053	3.30573378	-3.40380159	H	3.56091078	3.72660518	-2.77313234	H	0.39567858	-0.01532696	-5.50870253
H	0.93927214	6.08887123	-1.58953752	H	0.14543230	6.24173946	-2.05990384	H	2.04307358	3.84172866	-4.55612204
H	3.19710483	5.57858462	-2.50453245	H	2.60518432	5.98643702	-2.34263183	H	1.91343350	1.87594645	-6.07639971
H	-2.40717557	3.08048522	-2.87119353	H	-2.57450065	2.60198305	-3.11606940	H	-2.13099394	3.93445346	-2.07779578
H	-1.61088884	2.53361382	-4.35372141	H	-1.60307367	2.28587439	-4.56120036	H	-2.92377346	2.51172830	-2.76903824
H	-2.63653287	1.41615366	-3.43188249	H	-2.43457285	0.95297156	-3.73419595	H	-3.09783445	2.86980029	-1.03826030

Table S7c Molecular structure plots, computed energies (excerpts from the *Gaussian* output) and atomic coordinates (in [Å]) for the intrinsic reaction coordinate **TS3** (for details, see also Table S7a).

	**starting geometry**	**transition state**	**product geometry**
**TS3**	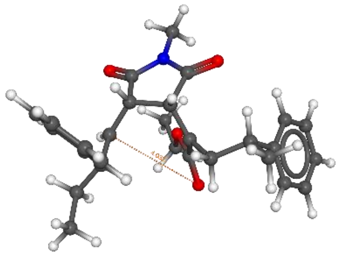	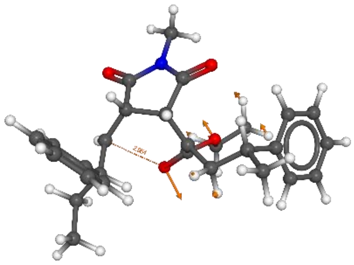	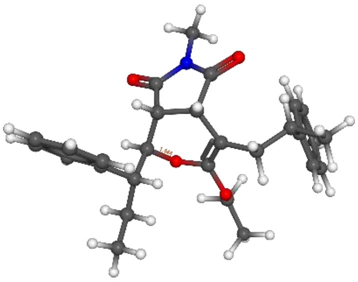
	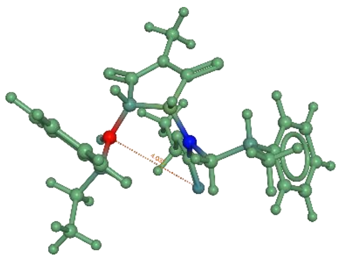	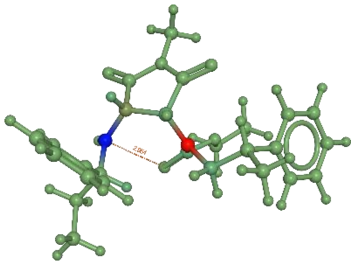	N/A
	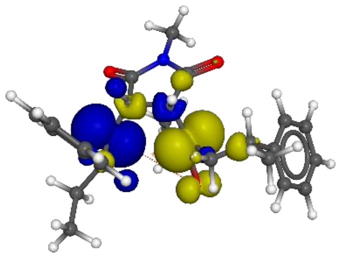	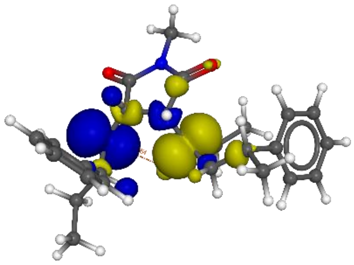	N/A
	SCF Energy: E(UB3LYP) = -1442.46610195 A.U.<br>Zero-point correction= 0.554707<br>Thermal correction to Energy= 0.588366<br>Thermal correction to Enthalpy= 0.589310<br>Thermal correction to Gibbs Free Energy= 0.484209<br>Sum of electronic and zero-point Energies= -1441.911395<br>Sum of electronic and thermal Energies= -1441.877736<br>Sum of electronic and thermal Enthalpies= -1441.876792<br>Sum of electronic and thermal Free Energies= -1441.981893	SCF Energy: E(UB3LYP) = -1442.44831941 A.U.<br>Zero-point correction= 0.554227<br>Thermal correction to Energy= 0.587148<br>Thermal correction to Enthalpy= 0.588092<br>Thermal correction to Gibbs Free Energy= 0.485703<br>Sum of electronic and zero-point Energies= -1441.894093<br>Sum of electronic and thermal Energies= -1441.861171<br>Sum of electronic and thermal Enthalpies= -1441.860227<br>Sum of electronic and thermal Free Energies= -1441.962617<br>Imaginary frequency: -64.2250 cm-1	SCF Energy: E(UB3LYP) = -1442.52908585 A.U.<br>Zero-point correction= 0.561402<br>Thermal correction to Energy= 0.593279<br>Thermal correction to Enthalpy= 0.594223<br>Thermal correction to Gibbs Free Energy= 0.494186<br>Sum of electronic and zero-point Energies= -1441.967683<br>Sum of electronic and thermal Energies= -1441.935807<br>Sum of electronic and thermal Enthalpies= -1441.934862<br>Sum of electronic and thermal Free Energies= -1442.034900
	C -3.23905405 -0.22182948 4.11482186	C -3.53459410 -0.37316132 4.05936067	C -3.16165480 0.57706297 3.78052186
	C -1.83795947 -0.73539417 3.77650006	C -2.16419539 -1.00694004 3.80987092	C -1.75741786 0.15010049 3.34240287
	C -1.61286650 -1.03676944 2.27288404	C -1.82287739 -1.22278130 2.31374618	C -1.74641367 -0.71370580 2.05920050
	C -2.54332618 -2.13604625 1.75633162	C -2.80221619 -2.17209771 1.62356058	C -2.51517879 -2.01808487 2.22565084
	C -3.60116633 -1.83924315 0.88779908	C -3.76608952 -1.69381354 0.72663430	C -3.63839366 -2.29030717 1.43210531
	C -2.36087959 -3.47109218 2.14983621	C -2.76730527 -3.55081264 1.88568180	C -2.12478237 -2.98078267 3.17047685
	C -4.45688759 -2.84252463 0.42578652	C -4.67140126 -2.56121444 0.11005078	C -4.35086867 -3.48310178 1.57258797
	C -3.21200403 -4.47579684 1.69060742	C -3.66799085 -4.42101637 1.27202455	C -2.83170187 -4.17483167 3.31210931
	C -4.26453477 -4.16481717 0.82571917	C -4.62474301 -3.92881125 0.38057118	C -3.94812919 -4.43115345 2.51329073
	C -0.16441667 -1.33854295 2.00888859	C -0.39450089 -1.65013208 2.15683208	C -0.29189646 -0.98958352 1.59364499
	C -0.14845445 0.58474685 -0.38142961	C -0.03809821 0.51369933 -0.10259037	C -0.31103387 0.58587646 -0.77585938
	C 0.33569818 -1.79245200 0.68162430	C 0.18895369 -1.97453344 0.82936351	C -0.17523833 -1.76861867 0.28209216
	C 0.16976315 -0.86843098 -0.59836061	C 0.07607029 -0.95543258 -0.38886291	C -0.35861769 -0.92153911 -1.00473781
	C 1.82143404 -2.14258277 0.69617402	C 1.67923760 -2.30489179 0.89062597	C 1.20944958 -2.40318044 0.15363443
	C 1.50056489 -1.09072205 -1.35246113	C 1.38315936 -1.24169572 -1.16293003	C 0.79258873 -1.41250413 -1.90671246
	O 2.44727835 -2.65364316 1.60229526	O 2.28116436 -2.85335630 1.78859336	O 1.81140104 -3.02357561 1.00611015
	O 1.76988766 -0.71003864 -2.47465931	O 1.64143682 -0.92838881 -2.30877319	O 0.96236502 -1.19459559 -3.09099227
	N 2.35672399 -1.82324215 -0.55049109	N 2.25976109 -1.90218714 -0.31757460	N 1.66094345 -2.18269183 -1.14183417
	C 3.73203416 -2.10597107 -0.92884118	C 3.63485227 -2.20184055 -0.68661832	C 2.90638523 -2.72235150 -1.66468009
	C 0.62319727 1.42135902 0.53388551	C 0.86266069 1.15155232 0.90047604	C 0.09216976 1.03974201 0.42649369
	O 0.26979624 2.52266477 0.93058138	O 0.68226988 1.09972975 2.10421696	O 0.46555733 0.23398426 1.46926218
	O 1.79025341 0.83533613 0.92207960	O 1.89454719 1.80342255 0.33574014	O 0.08158556 2.35353624 0.75266510
	C 2.58919950 1.58594805 1.86485247	C 2.79665888 2.48625033 1.24704139	C 1.25013587 2.86832358 1.43147796
	C 3.88144409 0.82176011 2.08538913	C 3.94568660 3.03931481 0.42725151	C 0.94750766 4.30030089 1.82918500
	C -1.28250466 1.21423383 -1.12698495	C -1.04389549 1.34755122 -0.84772492	C -0.86399733 1.52729881 -1.82655770
	C -0.91746463 1.64501458 -2.58888722	C -0.70320259 1.66702088 -2.34241798	C 0.03920986 1.88473201 -3.04338569

C 0.08353403 2.79379034 -2.62423014	C 0.37112076 2.73485729 -2.50097951	C 1.32923059 2.58484681 -2.63760994
C -0.25428551 4.07217477 -2.15572824	C 0.15716474 4.05446303 -2.07554076	C 1.33173235 3.89674959 -2.13880021
C 1.36882012 2.59559762 -3.14565868	C 1.58844150 2.42721030 -3.12183788	C 2.56186854 1.93169547 -2.77458241
C 2.28757552 3.64633266 -3.20632158	C 2.55873425 3.41070400 -3.32400276	C 3.75783083 2.56319259 -2.42402404
C 0.66034630 5.12255478 -2.21469733	C 1.12577855 5.03904983 -2.27040998	C 2.52348392 4.53379920 -1.79116250
C 1.93592963 4.91430474 -2.74412684	C 2.33132663 4.72134147 -2.90125163	C 3.74416004 3.86806811 -1.93093751
C -2.20353633 1.97819526 -3.36718415	C -1.99143951 2.05476556 -3.09477389	C -0.77093603 2.71544353 -4.05678754
H -3.32545313 -0.01017747 5.18636148	H -3.70422842 -0.22578521 5.13189411	H -3.11298669 1.20557576 4.67674291
H -3.46234235 0.70645565 3.57443394	H -3.61052516 0.60794111 3.57407539	H -3.66062262 1.15824279 2.99510031
H -4.01058501 -0.95406410 3.85435012	H -4.34646278 -0.99957478 3.67453749	H -3.79484697 -0.28661719 4.00942618
H -1.62398483 -1.64429089 4.35475260	H -2.09889813 -1.97100610 4.33205463	H -1.26791650 -0.40333826 4.15570918
H -1.09224922 0.01005009 4.08328850	H -1.38175103 -0.36554091 4.23462539	H -1.14488419 1.03857162 3.15986224
H -1.88005710 -0.11601924 1.72038891	H -1.92812627 -0.23718827 1.82026279	H -2.24068769 -0.13512432 1.26753917
H -3.76116036 -0.80924383 0.57558055	H -3.81354617 -0.62730347 0.51574881	H -3.96170210 -1.55482828 0.69849719
H -1.53965840 -3.72220230 2.81746432	H -2.02158291 -3.94124621 2.57468340	H -1.26038055 -2.79928144 3.80423604
H -5.27256107 -2.58854207 -0.24634770	H -5.41180191 -2.16665777 -0.58129803	H -5.22031045 -3.66863544 0.94725178
H -3.05306379 -5.50343385 2.00730620	H -3.62288019 -5.48526118 1.48917357	H -2.50895582 -4.90653515 4.04806443
H -4.92718392 -4.94769257 0.46676672	H -5.32608749 -4.60645485 -0.09891758	H -4.49903537 -5.36108922 2.62488418
H 0.56024582 -1.22274852 2.80854484	H 0.28533657 -1.54216823 2.99450177	H 0.23918560 -1.54820356 2.36893458
H -0.18060562 -2.73113464 0.41752730	H -0.29276341 -2.89003227 0.44252415	H -0.89232367 -2.59698239 0.29944901
H -0.61389964 -1.27859450 -1.24324533	H -0.75878950 -1.23727825 -1.03748997	H -1.29270357 -1.17111955 -1.52440594
H 4.14778162 -2.78394643 -0.18281290	H 4.11511992 -2.65545056 0.18107088	H 3.28804180 -3.43941906 -0.93699113
H 3.75282113 -2.56369479 -1.92052326	H 3.65888543 -2.89288983 -1.53431088	H 2.71899573 -3.20759022 -2.62530641
H 4.31685790 -1.18129411 -0.95503945	H 4.15147072 -1.28132761 -0.97017983	H 3.63753979 -1.92141021 -1.81103053
H 2.76275498 2.58681900 1.45861456	H 2.23659271 3.27617461 1.75804324	H 1.46438275 2.24871822 2.30738024
H 2.01680141 1.70005396 2.79195463	H 3.13155658 1.77271743 2.00595271	H 2.10229511 2.81823463 0.74311265
H 4.45529968 0.74991073 1.15527436	H 3.58312705 3.73169727 -0.33766358	H 0.08279093 4.34281767 2.49960289
H 4.49710691 1.34372807 2.82615224	H 4.64161405 3.57276185 1.08445335	H 1.81023786 4.73337068 2.34729746
H 3.68406320 -0.19181183 2.44803858	H 4.49325683 2.23284647 -0.07124972	H 0.73309631 4.90902476 0.94563735
H -2.11249319 0.49638468 -1.19309339	H -1.99801319 0.79830345 -0.84746098	H -1.78468469 1.08063783 -2.23307836
H -1.63482226 2.09033824 -0.57432147	H -1.22577574 2.28734252 -0.31222274	H -1.16341370 2.45784784 -1.33307275
H -0.44251526 0.78349310 -3.07296472	H -0.31622949 0.74527969 -2.79059616	H 0.31485133 0.94697778 -3.53210975
H -1.23966427 4.25254494 -1.73329012	H -0.78006468 4.32358127 -1.59305931	H 0.39198486 4.43096049 -2.02320752
H 1.65352207 1.60600735 -3.49134114	H 1.77837324 1.40468270 -3.43556411	H 2.57945027 0.91762710 -3.16553314
H 3.27912740 3.47017814 -3.61617057	H 3.49422091 3.15074379 -3.81326742	H 4.70134844 2.03589074 -2.54317042
H 0.37757769 6.10441434 -1.84368401	H 0.93620695 6.05641679 -1.93695497	H 2.50046233 5.55430292 -1.41608083
H 2.64900596 5.73340672 -2.79132379	H 3.08307512 5.48956110 -3.06404926	H 4.67331200 4.36491736 -1.66372381
H -2.75475130 2.80291315 -2.90080546	H -2.46674518 2.93977659 -2.65507255	H -1.13487699 3.65333123 -3.61992388
H -1.96639522 2.27450772 -4.39432732	H -1.77298319 2.28187634 -4.14337757	H -0.16219938 2.96911226 -4.93127888
H -2.87233469 1.10989038 -3.40764019	H -2.72047096 1.23593523 -3.06735950	H -1.64543522 2.15225729 -4.40431247

Table S7d Molecular structure plots, computed energies (excerpts from the *Gaussian* output) and atomic coordinates (in [Å]) for the intrinsic reaction coordinate **TS4** (for details, see also Table S7a).

	**starting geometry**	**transition state**	**product geometry**
**TS4**	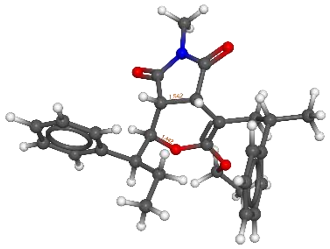	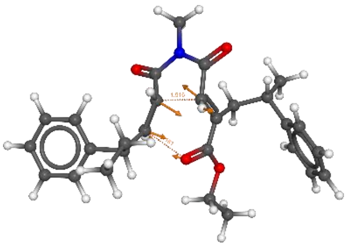	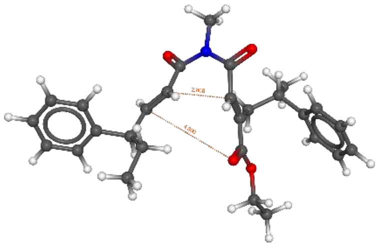
	SCF Energy: E(UB3LYP) = -1442.52566438 A.U.<br>Zero-point correction= 0.561362<br>Thermal correction to Energy= 0.593294<br>Thermal correction to Enthalpy= 0.594238<br>Thermal correction to Gibbs Free Energy= 0.494551<br>Sum of electronic and zero-point Energies= -1441.964303<br>Sum of electronic and thermal Energies= -1441.932371<br>Sum of electronic and thermal Enthalpies= -1441.931427<br>Sum of electronic and thermal Free Energies= -1442.031113	SCF Energy: E(UB3LYP) = -1442.46184547 A.U.<br>Zero-point correction= 0.556161<br>Thermal correction to Energy= 0.588808<br>Thermal correction to Enthalpy= 0.589752<br>Thermal correction to Gibbs Free Energy= 0.487533<br>Sum of electronic and zero-point Energies= -1441.905685<br>Sum of electronic and thermal Energies= -1441.873038<br>Sum of electronic and thermal Enthalpies= -1441.872094<br>Sum of electronic and thermal Free Energies= -1441.974313<br>Imaginary frequency: -470.4503 cm-1	SCF Energy: E(UB3LYP) = -1442.50989003 A.U.<br>Zero-point correction= 0.557043<br>Thermal correction to Energy= 0.590891<br>Thermal correction to Enthalpy= 0.591835<br>Thermal correction to Gibbs Free Energy= 0.485093<br>Sum of electronic and zero-point Energies= -1441.952847<br>Sum of electronic and thermal Energies= -1441.918999<br>Sum of electronic and thermal Enthalpies= -1441.918055<br>Sum of electronic and thermal Free Energies= -1442.024797
	C 0.95262122 0.09876294 3.59291609	C -0.31030432 -2.30673165 4.58633163	C -1.51394255 -1.29139558 4.42941195
	C 0.45376452 -0.02282744 2.14790606	C -0.21178542 -2.20892793 3.06084145	C -1.17214664 -1.60845909 2.97038146
	C -1.02673641 -0.43423895 2.04842555	C -1.38245301 -1.43042273 2.42167126	C -2.40936519 -1.67318867 2.04684278
	C -1.35326168 -1.80967467 2.62710719	C -2.73370122 -2.12969715 2.60153709	C -3.40220069 -2.76287948 2.45943551
	C -2.60839764 -2.02343362 3.21685245	C -3.66780394 -1.62801252 3.51783211	C -4.63750804 -2.43249197 3.02944473
	C -0.46592662 -2.89429415 2.56344530	C -3.06784964 -3.27920117 1.87014024	C -3.08939728 -4.11817358 2.28099421
	C -2.97054653 -3.27232845 3.71980957	C -4.89527857 -2.26301938 3.71334130	C -5.53610903 -3.42731260 3.42010453
	C -0.82238405 -4.14724205 3.06804479	C -4.29555183 -3.91363501 2.06035094	C -3.98385836 -5.11467199 2.67054776
	C -2.07632089 -4.34193248 3.64666424	C -5.21268799 -3.40993799 2.98466870	C -5.21137206 -4.77232479 3.24207652
	C -1.63699100 -0.31306821 0.62039121	C -1.17983760 -1.10861405 0.95995415	C -2.08458525 -1.82064325 0.57967082
	C 0.12945626 0.90705355 -1.36062259	C 0.78587923 0.59544169 -0.83227045	C 0.96847368 0.85744865 -0.51547259

C	0.16962810	-0.60887699	-1.26574358
C	-1.01319066	-1.19072805	-0.46584789
C	-0.03156468	-1.20906654	-2.66600920
C	-2.02697639	-1.59314216	-1.54380244
O	0.77281802	-1.25397569	-3.57535591
O	-3.20314714	-1.85433800	-1.39551713
N	-1.34555702	-1.67070097	-2.75149433
C	-1.95469523	-2.15605971	-3.97950068
C	-0.75378126	1.59140097	-0.60857054
O	-1.68699444	1.07570477	0.23728970
O	-0.77953241	2.94829487	-0.60271137
C	-2.07085236	3.58901360	-0.55380089
C	-1.83631747	5.08200067	-0.69189047
C	1.08860301	1.61454704	-2.29243344
C	2.59122074	1.29381270	-2.08855333
C	3.10604879	1.52422975	-0.67472092
C	2.74976471	2.65503408	0.07620182
C	4.01477171	0.61878271	-0.10812989
C	4.56061579	0.83373062	1.15891319
C	3.29116808	2.87416548	1.34401533
C	4.20181203	1.96685543	1.89050415
C	3.43038499	2.09614346	-3.10473149
H	1.99376358	0.43674482	3.60801543
H	0.35501714	0.82934161	4.15243488
H	0.89435232	-0.85521280	4.12762370
H	1.09301612	-0.72611373	1.59906820
H	0.58286791	0.94320603	1.65063945
H	-1.59856837	0.29858110	2.63478357
H	-3.31077426	-1.19494196	3.28388816
H	0.52221051	-2.76312003	2.13106087
H	-3.94919551	-3.40860134	4.17245682
H	-0.11432024	-4.96999547	3.01122217
H	-2.35299192	-5.31604017	4.04072289
H	-2.68871048	-0.60501823	0.69596075
H	1.14157170	-0.94828052	-0.88707965
H	-0.71339828	-2.13479548	0.00638260
H	-1.16437471	-2.24567337	-4.72574849
H	-2.42361309	-3.12708924	-3.80180063
H	-2.72003147	-1.45608140	-4.32841724
H	-2.69521400	3.20596158	-1.37194885
H	-2.56512759	3.34893691	0.39221485
H	-1.35290054	5.31621738	-1.64570991
H	-2.79180628	5.61590406	-0.64637988
H	-1.19709304	5.44791151	0.11815116
H	0.85846134	1.34685316	-3.33268297
H	0.92550239	2.69240070	-2.21103097
H	2.73295678	0.23176662	-2.32539319
H	2.03299656	3.36670495	-0.32346747
H	4.30574846	-0.26401975	-0.67462453
H	5.26638066	0.11727662	1.57212867
H	3.00006345	3.75766077	1.90694796
H	4.62653401	2.14197717	2.87562824
H	3.33146565	3.17450714	-2.92962832
H	4.49289320	1.84171672	-3.02663406
H	3.10291854	1.88528816	-4.12974158

C	1.13664225	-0.75875138	-0.55372994
C	-0.33462232	-1.81068667	0.07804051
C	1.47243500	-1.63836964	-1.74034256
C	-0.77664609	-2.24139567	-1.30001770
O	2.55115475	-1.71259863	-2.29834010
O	-1.89706383	-2.56556701	-1.64094719
N	0.33014538	-2.32418581	-2.15454169
C	0.26024422	-3.03114305	-3.42686422
C	0.25535044	1.26891109	0.29029209
O	0.02621838	0.66110099	1.37183339
O	-0.14980243	2.55335231	0.10791025
C	-0.71930983	3.21687796	1.25005303
C	-0.96904592	4.66097297	0.85481751
C	0.71124511	1.17748114	-2.22423879
C	2.07970823	1.65037834	-2.80473677
C	2.72162850	2.75645224	-1.97868464
C	2.17857120	4.04876890	-1.92463889
C	3.89398273	2.50488757	-1.25443738
C	4.50918438	3.50758476	-0.50156663
C	2.78979980	5.05536250	-1.17731878
C	3.95952365	4.78875678	-0.46139939
C	1.91102318	2.05398706	-4.28085691
H	0.54597397	-2.85440327	4.99501703
H	-0.31802135	-1.31084634	5.04569483
H	-1.22229305	-2.82687154	4.89742652
H	-0.16424168	-3.22167909	2.63839292
H	0.72168689	-1.69949810	2.79112651
H	-1.45021123	-0.46537638	2.93805517
H	-3.42844582	-0.73182544	4.08590659
H	-2.37632968	-3.67070164	1.12933445
H	-5.60331804	-1.85879842	4.43222333
H	-4.53806201	-4.79919738	1.47890081
H	-6.16933726	-3.90424724	3.13109934
H	-1.96166267	-0.49594322	0.51813173
H	1.75311784	-0.91565229	0.32865539
H	0.24545488	-2.60933338	0.54279068
H	1.26837837	-3.06113564	-3.84100755
H	-0.11526372	-4.04545744	-3.26999173
H	-0.41409724	-2.51195956	-4.11413151
H	-1.64705208	2.71076320	1.54177568
H	-0.02436966	3.14203412	2.09358723
H	-1.66660000	4.72034268	0.01279062
H	-1.39959073	5.21102936	1.69891000
H	-0.03272532	5.14602143	0.56234749
H	0.30532318	0.43036455	-2.92204482
H	0.01099491	2.01876142	-2.22767603
H	2.75378841	0.78721024	-2.77212929
H	1.26612335	4.27399608	-2.47086744
H	4.33053747	1.50899742	-1.28378655
H	5.41942886	3.28625144	0.05021083
H	2.35478218	6.05188642	-1.15772178
H	4.43743232	5.57352557	0.11932770
H	1.20368996	2.88386088	-4.39727704
H	2.86743679	2.36836883	-4.71196755
H	1.53292711	1.21052819	-4.87101639

C	1.46646560	-0.38565007	-0.40486778
C	-0.86987277	-1.98963686	0.03543947
C	1.65008081	-1.34268427	-1.54098193
C	-0.73133689	-2.16105518	-1.43063905
O	2.74961667	-1.40829713	-2.07740314
O	-1.67174490	-2.43760807	-2.15946247
N	0.57284364	-2.10181384	-1.99717494
C	0.73488684	-2.77508126	-3.29567918
C	1.01339404	1.66407381	0.75095236
O	1.39936568	1.23764754	1.82524540
O	0.55089999	2.91621849	0.58059814
C	0.58489488	3.76781093	1.74994720
C	0.08904008	5.13783423	1.32943211
C	0.41267297	1.44900390	-1.79443732
C	1.50066601	1.87103373	-2.83040636
C	2.50164679	2.86761149	-2.25942477
C	2.21649048	4.23668068	-2.15450336
C	3.75064196	2.41469026	-1.81084017
C	4.68629537	3.30013307	-1.27176137
C	3.15107870	5.12556018	-1.62170473
C	4.39047549	4.66014190	-1.17639302
C	0.82288487	2.36450843	-4.11952788
H	-0.60145922	-1.22272683	5.03122801
H	-2.03780710	-0.33089986	4.51327954
H	-2.15475681	-2.06093261	4.87212664
H	-0.63409201	-2.56463279	2.92110477
H	-0.48688573	-0.84078353	2.58923815
H	-2.94128184	-0.71295817	2.14422790
H	-4.89782746	-1.38591248	3.17154585
H	-2.14199737	-4.39386179	1.82440291
H	-6.48969652	-3.14948198	3.86153943
H	-3.72426812	-6.15967322	2.52248642
H	-5.90990648	-5.54841904	3.54303415
H	-2.93700003	-1.79456016	-0.09796927
H	1.91170419	-0.67651573	0.54370440
H	0.01789491	-2.04134912	0.65232577
H	1.79574729	-2.96545962	-3.44509286
H	0.16846869	-3.70618817	-3.28556491
H	0.35521806	-2.14643316	-4.10709489
H	-0.04207992	3.31898987	2.52737588
H	1.61128794	3.79813098	2.12729250
H	-0.93598494	5.08554349	0.94787978
H	0.10158256	5.81621376	2.18952461
H	0.73073002	5.55544158	0.54782085
H	-0.23843034	0.71095205	-2.28021244
H	-0.21164395	2.31376442	-1.55583346
H	2.06650542	0.96665875	-3.08125898
H	1.25734542	4.61841695	-2.49449537
H	3.98587285	1.35526819	-1.88319892
H	5.64835930	2.92539494	-0.93179178
H	2.91344475	6.18501005	-1.56076947
H	5.11952332	5.35287220	-0.76408960
H	0.17949432	3.23319837	-3.93784771
H	1.56942690	2.65103167	-4.86763419
H	0.19392632	1.57637089	-4.54999123

Table S7e Molecular structure plots, computed energies (excerpts from the *Gaussian* output) and atomic coordinates (in [Å]) for the intrinsic reaction coordinate **TS5** (for details, see also Table S7a).

	**starting geometry**	**transition state**	**product geometry**
**TS5**	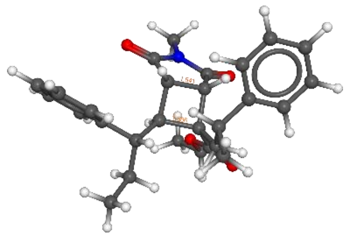	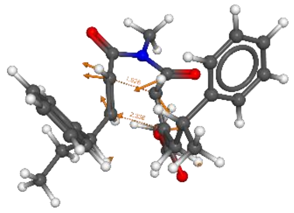	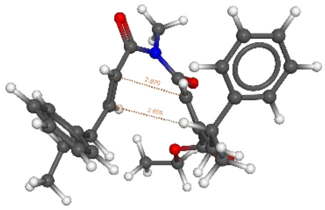
	SCF Energy: E(RB3LYP) = -1442.53266895 A.U.<br>Zero-point correction= 0.560010<br>Thermal correction to Energy= 0.592353<br>Thermal correction to Enthalpy= 0.593298<br>Thermal correction to Gibbs Free Energy= 0.492393<br>Sum of electronic and zero-point Energies= -1441.972659<br>Sum of electronic and thermal Energies= -1441.940316<br>Sum of electronic and thermal Enthalpies= -1441.939371<br>Sum of electronic and thermal Free Energies= -1442.040276	SCF Energy: E(RB3LYP) = -1442.40969779 A.U.<br>Zero-point correction= 0.555684<br>Thermal correction to Energy= 0.588338<br>Thermal correction to Enthalpy= 0.589282<br>Thermal correction to Gibbs Free Energy= 0.488946<br>Sum of electronic and zero-point Energies= -1441.854014<br>Sum of electronic and thermal Energies= -1441.821360<br>Sum of electronic and thermal Enthalpies= -1441.820416<br>Sum of electronic and thermal Free Energies= -1441.920752<br>Imaginary frequency: -832.7201 cm-1	SCF Energy: E(RB3LYP) = -1442.50423811 A.U.<br>Zero-point correction= 0.556099<br>Thermal correction to Energy= 0.590188<br>Thermal correction to Enthalpy= 0.591132<br>Thermal correction to Gibbs Free Energy= 0.483337<br>Sum of electronic and zero-point Energies= -1441.948139<br>Sum of electronic and thermal Energies= -1441.914050<br>Sum of electronic and thermal Enthalpies= -1441.913106<br>Sum of electronic and thermal Free Energies= -1442.020901
	C -1.67900692 0.24735834 1.48537846	C -1.30965714 0.07365637 2.19295660	C -1.20167731 -0.51894550 2.63715000
	C -0.24278579 -0.01319821 0.98986900	C 0.01486630 -0.34854865 1.55679577	C -0.01321032 -1.07113231 1.88083028
	C 0.50641807 1.07510857 0.11344491	C 0.33878230 1.21487757 -0.01216857	C 0.88977880 1.59763855 -0.51556105
	C 0.03203881 -1.08490009 -0.12327507	C 0.20216487 -1.59825151 0.88647879	C -0.02412418 -1.98083657 0.89686610
	C 1.02031771 -0.11069624 -0.79264056	C 0.70619588 -0.17317953 -0.30742014	C 1.13634365 0.28392479 -0.63449376
	C 0.86154918 -2.27750122 0.31891087	C 1.45329477 -2.38081811 0.90506013	C 1.19551683 -2.56461937 0.28290138
	C 2.39467817 -0.69487285 -0.49188792	C 2.18851418 -0.54628448 -0.41338727	C 2.37231225 -0.36843600 -0.11333859
	O 0.49012392 -3.32490848 0.80666138	O 1.55369944 -3.53434559 1.29026490	O 1.22815284 -3.75436350 0.00545341
	O 3.48288416 -0.20942606 -0.73065155	O 2.97093488 0.06234773 -1.11149431	O 3.43239790 0.23517342 -0.01138063
	N 2.20492077 -1.94766472 0.08802756	N 2.51538291 -1.67269042 0.32275020	N 2.34347373 -1.75300252 0.12173742
	C 3.31145036 -2.81268290 0.45751364	C 3.86279938 -2.22442801 0.25520630	C 3.65383281 -2.42098577 0.11397001
	C 1.62352858 1.84655133 0.82745097	C 1.20691255 2.12676381 0.69520983	C 1.77874488 2.50445082 0.29278900
	O 1.94776611 2.98922844 0.58175633	O 1.05382953 3.34275881 0.75414363	O 2.23966955 3.54008437 -0.13824015
	O 2.24114258 1.09418562 1.76305330	O 2.25044084 1.51028772 1.36418020	O 1.91719498 2.08475085 1.56700551
	C 3.42047730 1.68139979 2.36611847	C 3.21314894 2.39668604 1.96437891	C 2.85353687 2.84149599 2.37170470
	C 3.97186696 0.68039877 3.36274584	C 4.37453530 1.55114449 2.45556498	C 2.91091804 2.18936418 3.73972195
	C -0.37514716 2.10548235 -0.63275838	C -0.83540119 1.88675669 -0.70536960	C -0.25354585 2.33815266 -1.17357757
	H 0.37649205 -0.24410097 1.86044261	H 0.88771354 0.05146395 2.06184183	H 0.93801525 -0.64041618 2.19099910
	H -0.84803767 -1.39908585 -0.68678490	H -0.67866505 -2.15105434 0.58102953	H -0.94365549 -2.47008250 0.59042176
	H 0.94310641 0.07020139 -1.86526037	H 0.13221505 -0.58815947 -1.13466845	H 0.47193819 -0.33495335 -1.22637862
	H 4.13447337 1.90543586 1.56852460	H 3.53250967 3.13046814 1.21880168	H 3.82501050 2.82614271 1.86891370
	H 3.13615357 2.62464582 2.84205476	H 2.73549373 2.94838405 2.78191431	H 2.51536049 3.88129995 2.42287486
	C -3.03767566 1.75332841 3.08451116	C -2.25862177 0.47253095 4.56110385	C -1.98642921 0.11432114 5.01417615
	C -1.65286450 1.25223260 2.66467845	C -1.07712868 -0.04563166 3.73530538	C -0.96133767 -0.64467109 4.16722984
	H -2.96066225 2.46156697 3.91691303	H -2.02706553 0.41209210 5.63003450	H -1.75906802 0.01307852 6.08110297
	H -3.54088460 2.26853111 2.25659972	H -2.47536641 1.52072727 4.32369469	H -1.98429180 1.18451215 4.77263412
	H -3.68157533 0.92678408 3.40258257	H -3.16638405 -0.10990176 4.37797886	H -3.00086486 -0.26280741 4.84867533
	H -1.15540753 0.77899610 3.52182540	H -0.86346161 -1.08883762 3.99883924	H -0.95603328 -1.70568209 4.44663100
	H -1.02643795 2.11134487 2.38857539	H -0.17958521 0.53120827 3.99106805	H 0.04611434 -0.26648163 4.38642837
	C -2.39830970 -1.04584864 1.85034501	C -2.55263347 -0.67819190 1.73564706	C -2.54758363 -1.07055045 2.19219811
	C -3.59639287 -1.39297181 1.21186581	C -3.57059283 -0.00241003 1.04960200	C -3.45063481 -0.25503056 1.49826080
	C -1.89713787 -1.91411294 2.83155231	C -2.74534300 -2.03882520 2.03045094	C -2.92312980 -2.39579017 2.46180601
	C -4.27788022 -2.56633729 1.54153168	C -4.73405073 -0.66240023 0.64866388	C -4.69162890 -0.74217023 1.08275087
	C -2.57202579 -3.08808331 3.16261058	C -3.90533659 -2.70220829 1.62897040	C -4.16167451 -2.88713150 2.04837611
	C -3.76663208 -3.41853031 2.51979915	C -4.90331177 -2.01714857 0.93329241	C -5.05111475 -2.06175503 1.35698757
	H -4.00524339 -0.73202652 0.45012401	H -3.45582465 1.05610709 0.83267023	H -3.18071672 0.77759657 1.28666829
	H -0.96462948 -1.67938233 3.33834881	H -1.98588780 -2.58909780 2.57887267	H -2.24015790 -3.05268727 2.99419078
	H -5.20656119 -2.81220817 1.03291123	H -5.50774978 -0.11394364 0.11821392	H -5.37655884 -0.08895104 0.54846658
	H -2.15981579 -3.74970889 3.91968930	H -4.02938035 -3.75559434 1.86517884	H -4.43156503 -3.91706179 2.26647363
	H -4.29121905 -4.33462808 2.77752760	H -5.80703552 -2.53406089 0.62249942	H -6.01614495 -2.44477754 1.03646527
	H -2.25447992 0.71048881 0.67525796	H -1.45620153 1.13890863 1.99127460	H -1.19846281 0.56185149 2.41822927
	C -1.35791453 1.63622310 -1.73923189	C -1.51936197 1.15839646 -1.87987844	C -1.22099534 1.54479182 -2.07268188
	C -0.72636607 1.38830391 -3.10815053	C -0.60029791 0.78763657 -3.04096452	C -0.58769128 0.94936019 -3.32636991
	C 0.14245638 2.31387114 -3.70582846	C 0.51861083 1.55529170 -3.39316382	C 0.28925800 1.68391293 -4.13679635
	C -1.07012119 0.24196488 -3.83984550	C -0.90381684 -0.33718861 -3.82496628	C -0.93074832 -0.34839633 -3.73110975
	C -0.55812196 0.01787798 -5.11899207	C -0.12364527 -0.68266713 -4.92842511	C -0.41268881 -0.90100395 -4.90351441
	C 0.65754764 2.09396984 -4.98376026	C 1.30309625 1.21126241 -4.49583225	C 0.81050116 1.13564960 -5.30922140
	C 0.31086128 0.94439868 -5.69593192	C 0.98537628 0.09419595 -5.26860884	C 0.46210684 -0.15912023 -5.69778421
	C -2.48805440 2.68120592 -1.88328149	C -2.69081827 2.02004584 -2.40068383	C -2.41260259 2.44705029 -2.46049951
	H -1.83329003 0.69574347 -1.43462222	H -1.96128529 0.22431176 -1.50138489	H -1.62453470 0.71289507 -1.47856640
	H 0.42509163 3.21753690 -3.17244272	H 0.79379365 2.42091561 -2.79877196	H 0.57662837 2.69311003 -3.85372040
	H -1.74920041 -0.48622655 -3.40012225	H -1.76778138 -0.94755507 -3.56642158	H -1.61372629 -0.93408941 -3.11834079
	H -0.83790977 -0.88127656 -5.66189736	H -0.37961333 -1.55907436 -5.51857755	H -0.69056878 -1.91132663 -5.19225863
	H 1.33329500 2.82343066 -5.42248314	H 2.17162273 1.81563104 -4.74333141	H 1.49344685 1.72197579 -5.91845280

H	0.71511542	0.77259014	-6.68977656	H	1.60029575	-0.17330832	-6.12381165	H	0.87115369	-0.58625247	-6.60938853
H	-2.07925487	3.66454889	-2.14361999	H	-2.32254932	2.97240449	-2.79922286	H	-2.07433922	3.31277701	-3.04177149
H	-3.18987259	2.39161368	-2.67224208	H	-3.22873251	1.50642636	-3.20443195	H	-3.13294875	1.89534747	-3.07292131
H	-3.04780010	2.78725328	-0.94597162	H	-3.40492716	2.24229862	-1.59822115	H	-2.93295703	2.81905725	-1.56947572
H	-0.95457425	2.64215133	0.12827473	H	-1.62530243	2.13422428	0.02278948	H	-0.84457505	2.81618035	-0.37690351
H	0.30010354	2.85723863	-1.04902657	H	-0.48478079	2.86995686	-1.04197653	H	0.18688401	3.17954833	-1.72481966
H	2.89590888	-3.78461398	0.72659877	H	3.86948909	-3.14818458	0.83286755	H	3.48065325	-3.49367898	0.16450829
H	4.00146562	-2.90840691	-0.38427616	H	4.13578526	-2.42909840	-0.78365761	H	4.19601170	-2.17312891	-0.80183841
H	3.85665852	-2.39440472	1.31001056	H	4.57589519	-1.50627819	0.66881068	H	4.24888498	-2.08263337	0.96618781
H	4.25961365	-0.25000528	2.86275235	H	4.84449352	1.02097273	1.62070877	H	3.25600062	1.15334920	3.66181426
H	4.86077558	1.09587974	3.84975359	H	5.12950112	2.19023709	2.92692684	H	3.60920830	2.73615841	4.38273488
H	3.23363057	0.44649648	4.13686196	H	4.04367420	0.81278968	3.19448112	H	1.92690700	2.19499287	4.22089277

NMR spectra

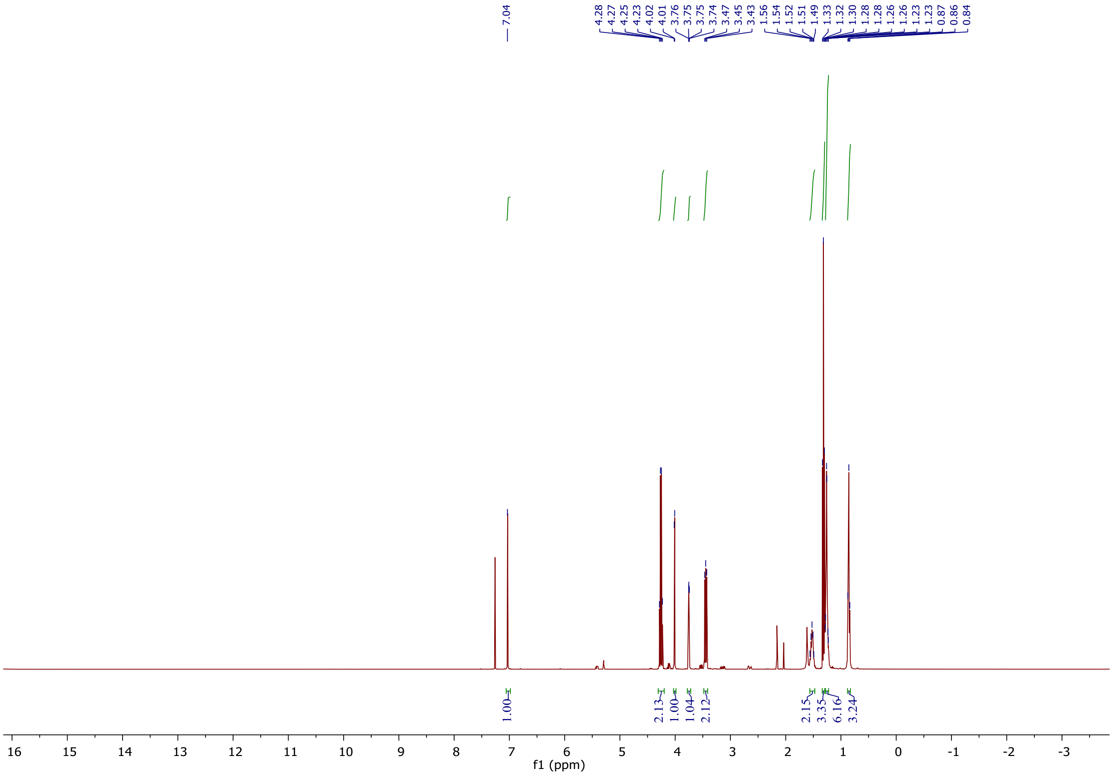

Fig. S117 ^{1}H NMR spectrum (400 MHz, $CDCl_3$) of HCBI.

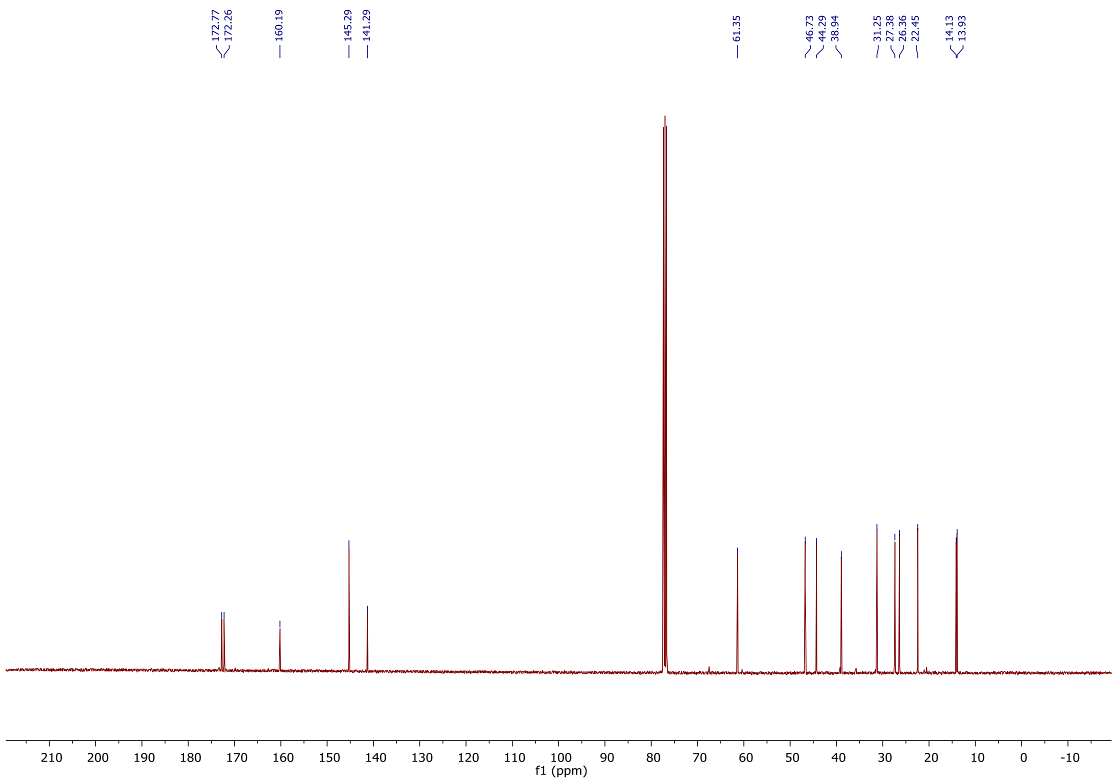

Fig. S118 ^{13}C NMR spectrum (101 MHz, $CDCl_3$) of HCBI.

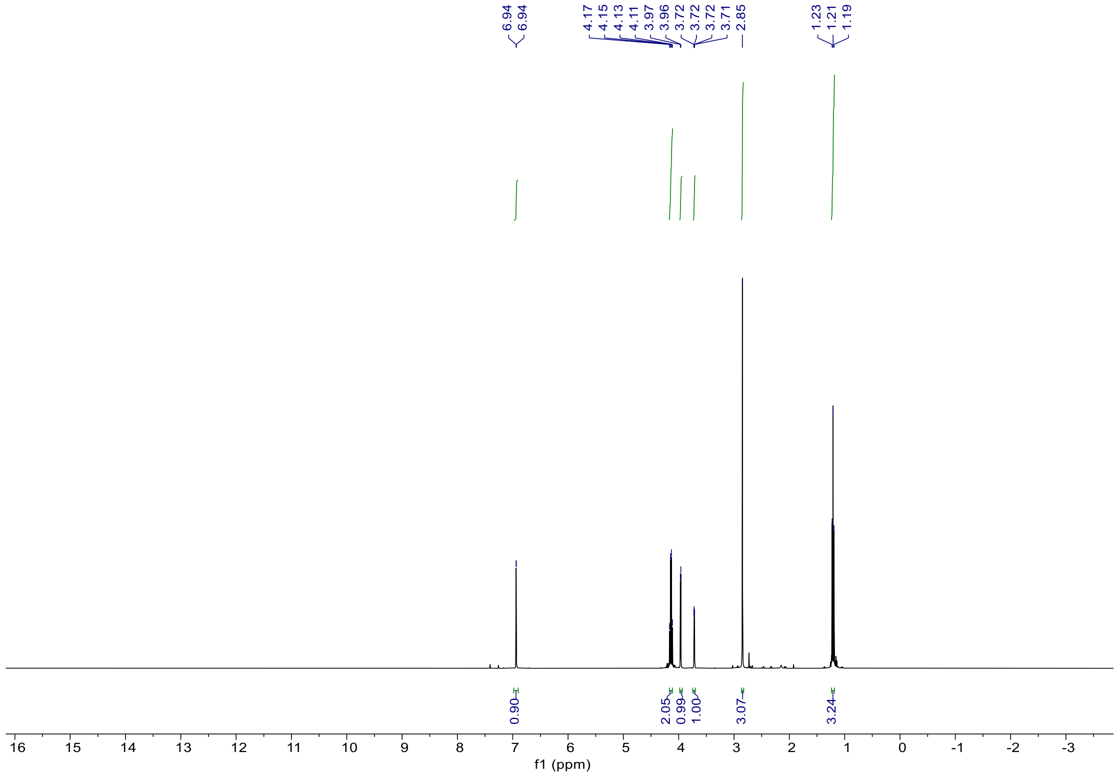

Fig. S119 ^{1}H NMR spectrum (400 MHz, $CDCl_3$) of MCBI.

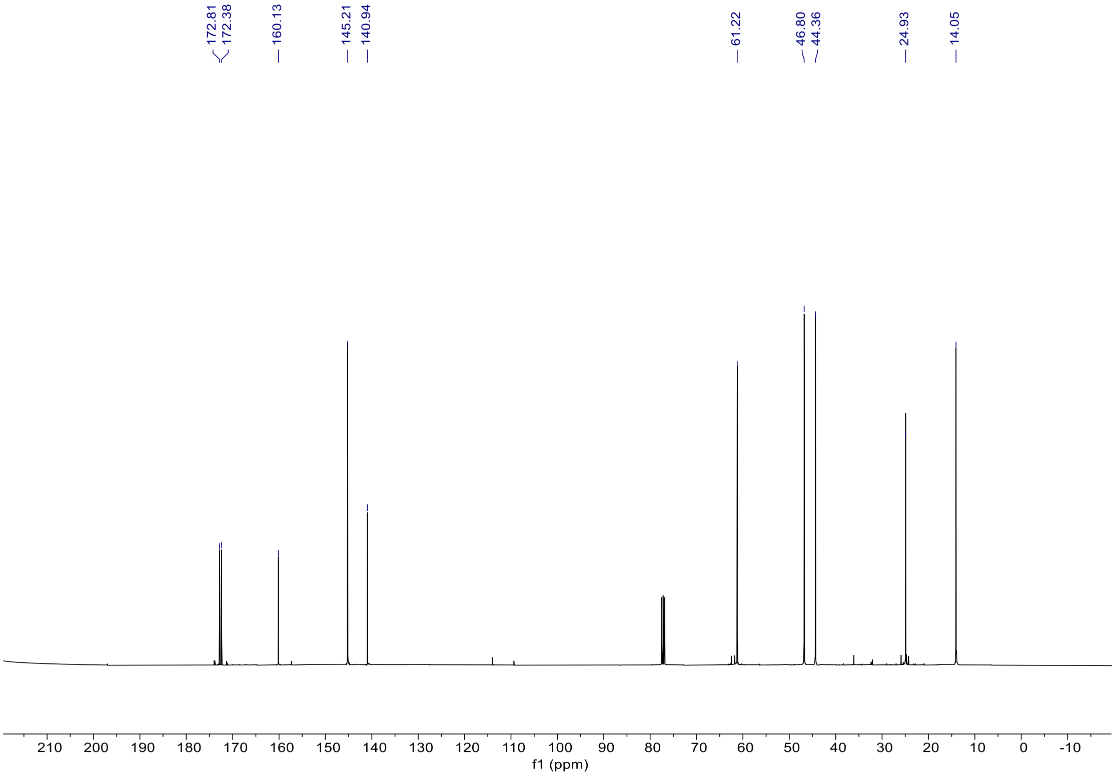

Fig. S120 ^{13}C NMR spectrum (101 MHz, $CDCl_3$) of MCBI.

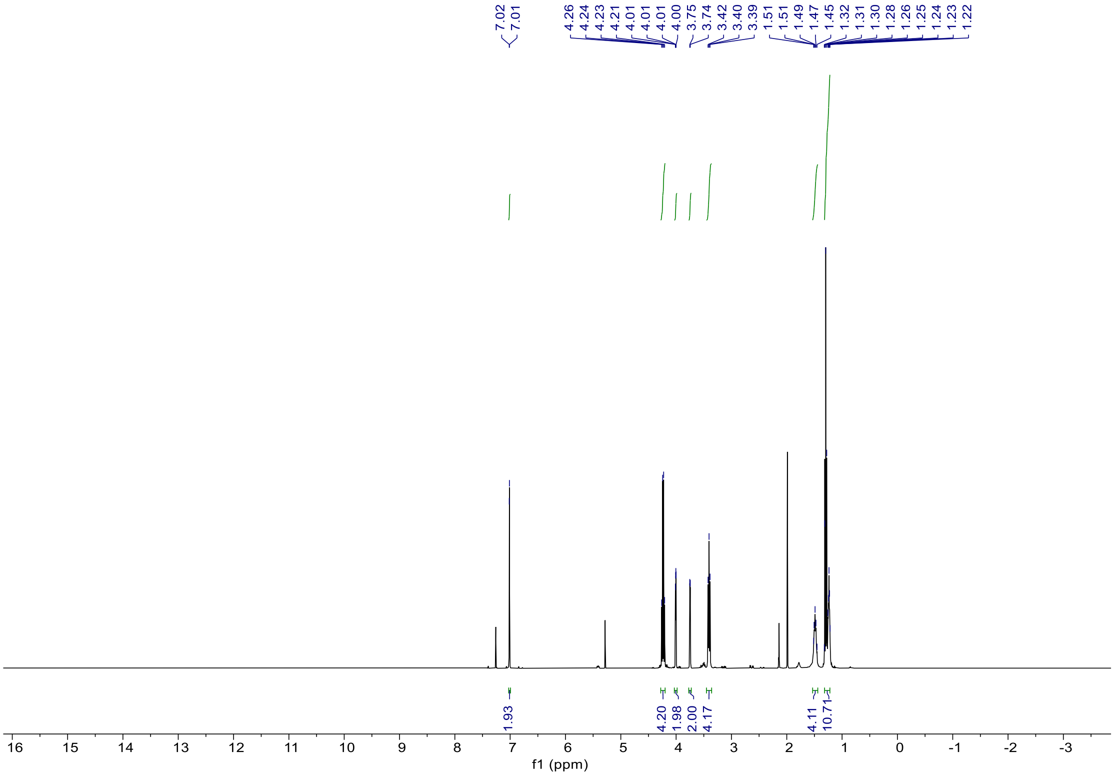

Fig. S121 ^{1}H NMR spectrum (400 MHz, $CDCl_3$) of DCBI.

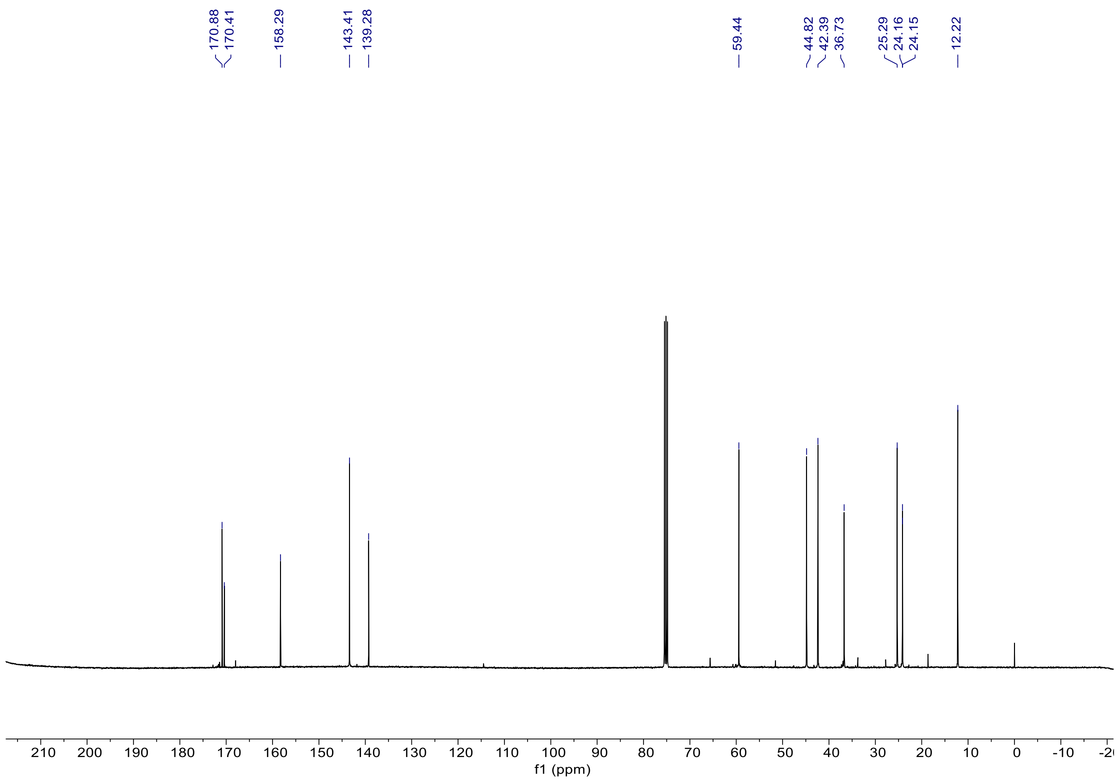

Fig. S122 ^{13}C NMR spectrum (101 MHz, $CDCl_3$) of DCBI.

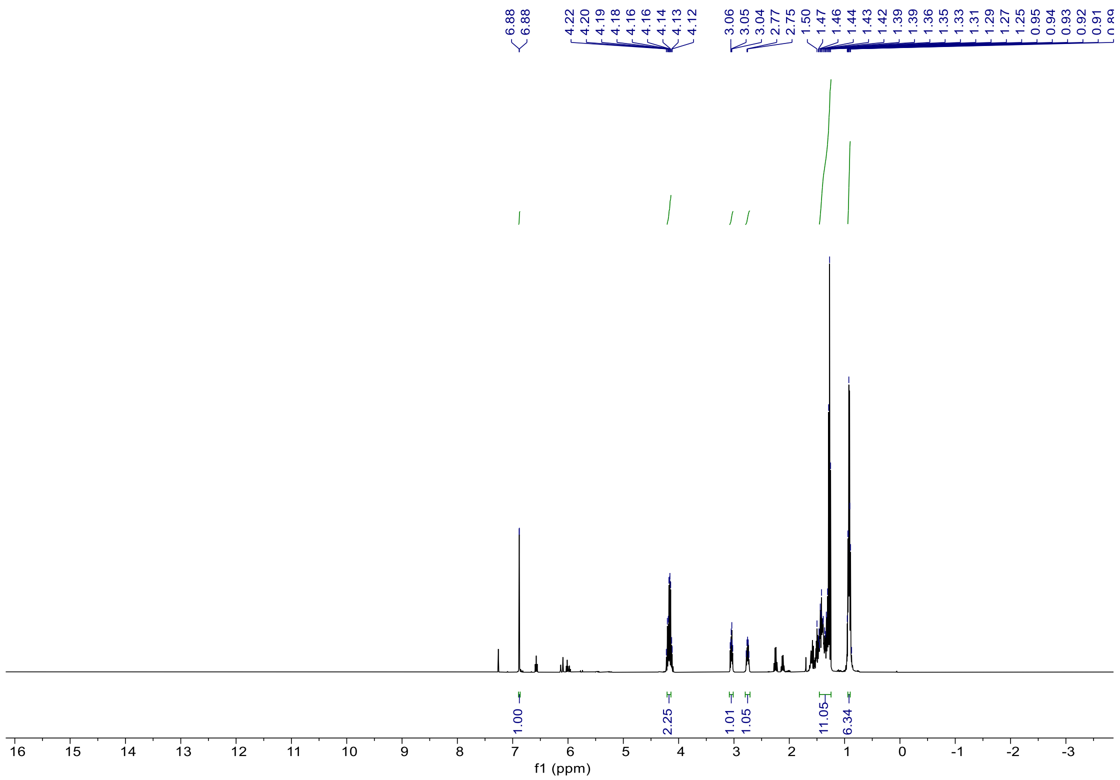

Fig. S123 ^{1}H NMR spectrum (400 MHz, $CDCl_3$) of CBO.

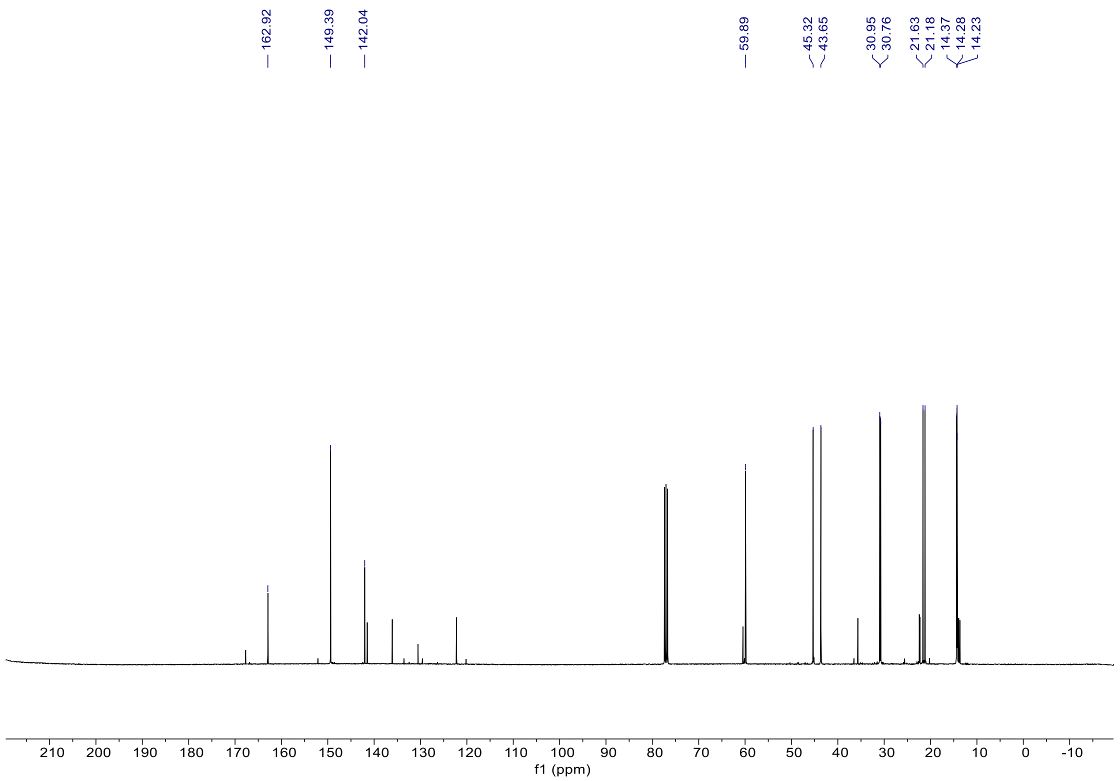

Fig. S124 ^{13}C NMR spectrum (101 MHz, $CDCl_3$) of CBO.

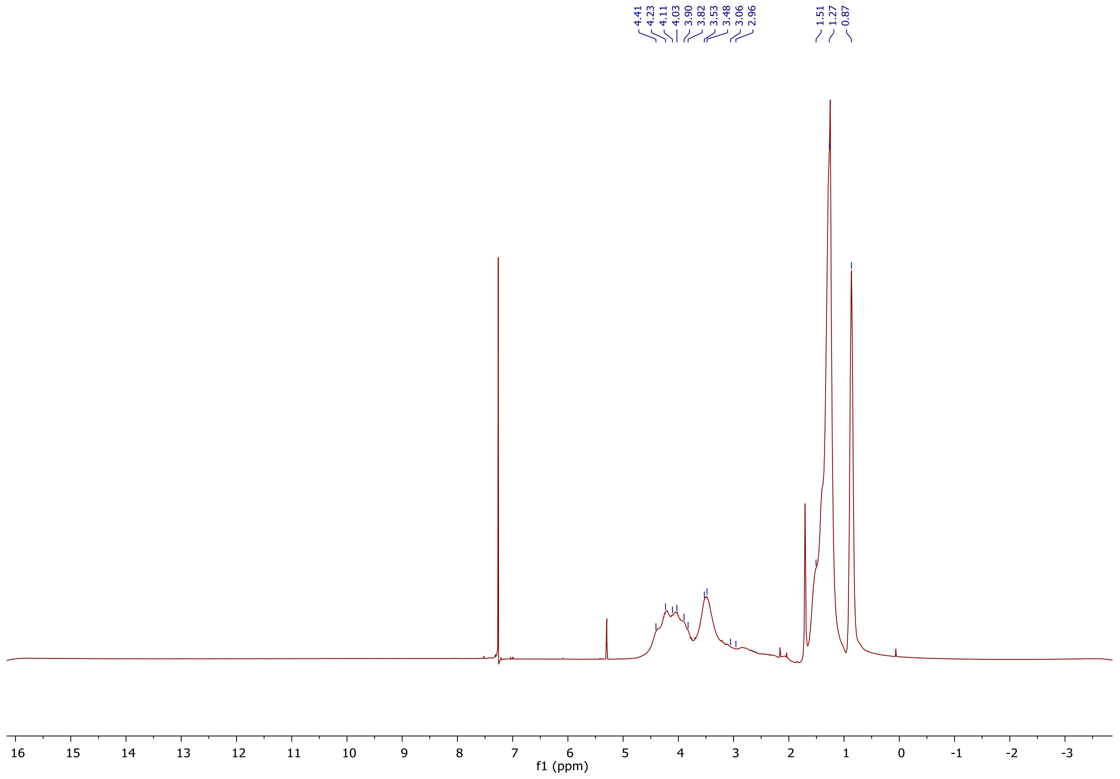

Fig. S125 ^{1}H NMR spectrum (400 MHz, $CDCl_3$) of PHCBI.

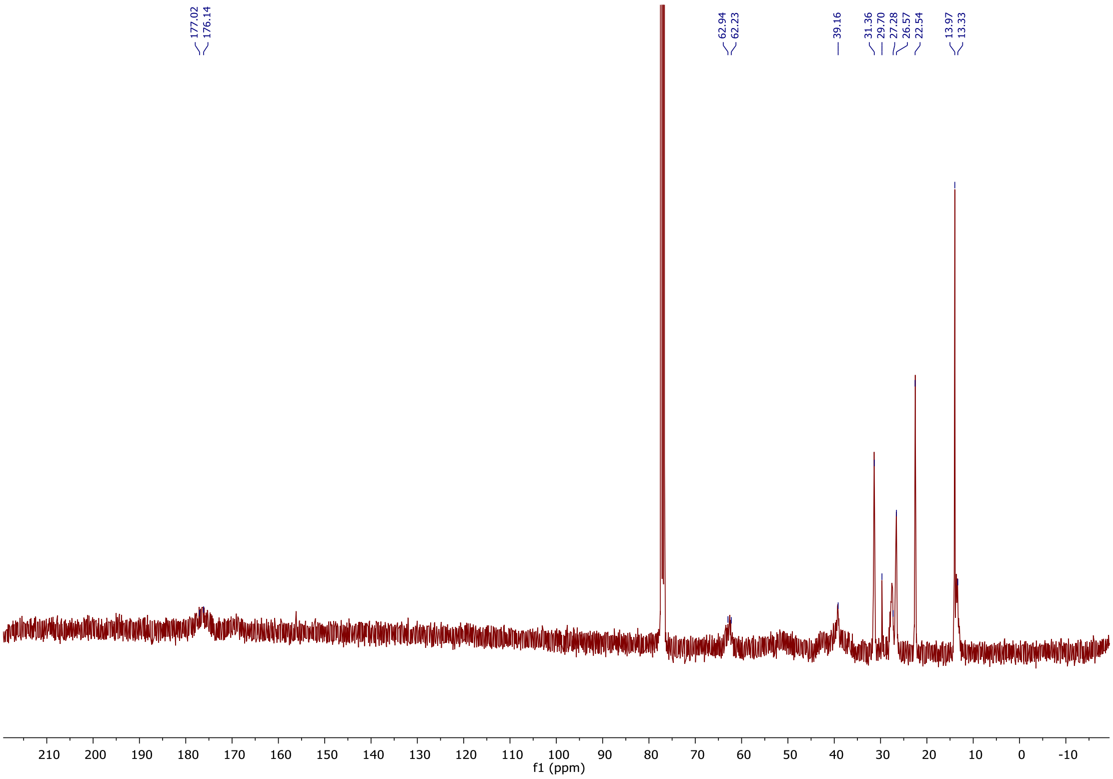

Fig. S126 ^{13}C NMR spectrum (101 MHz, $CDCl_3$) of PHCBI.

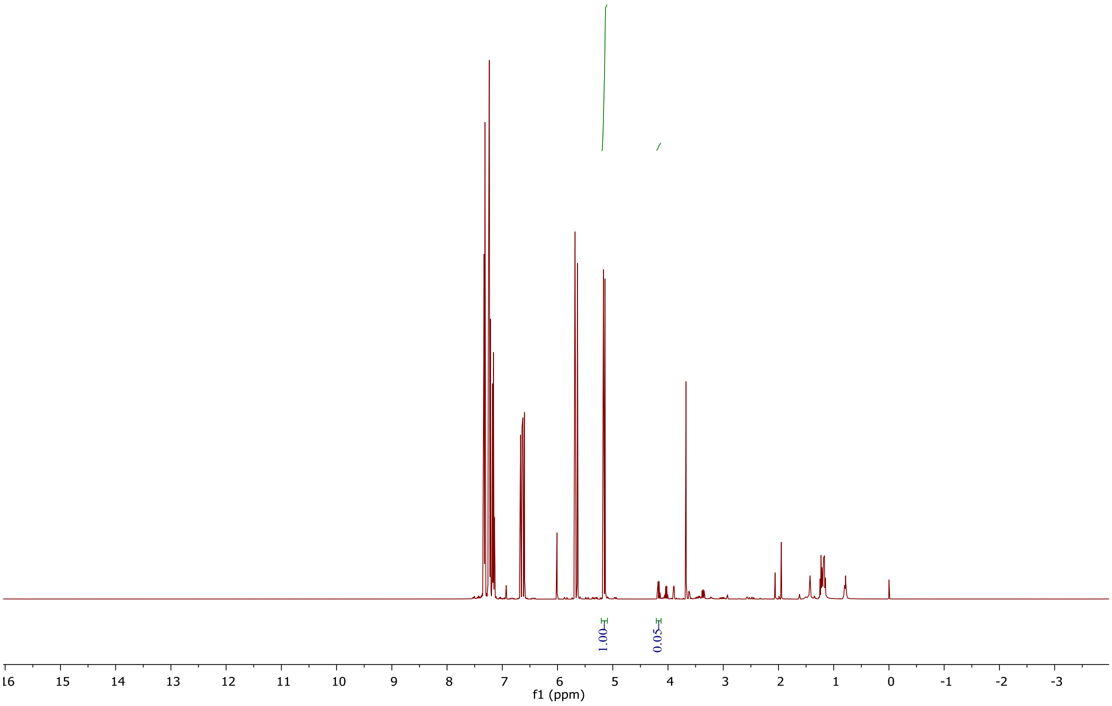

Fig. S127 ^{1}H NMR spectrum (400 MHz, $CDCl_3$) of reaction feed mixture for the synthesis of PS_{82}-*co*-$PHCBI_{18}$. (The fraction HCBI in feed is (0.05/2)/(0.05/2+1) ≈ 0.02. Therefore, the fraction of styrene in feed is 0.98.)

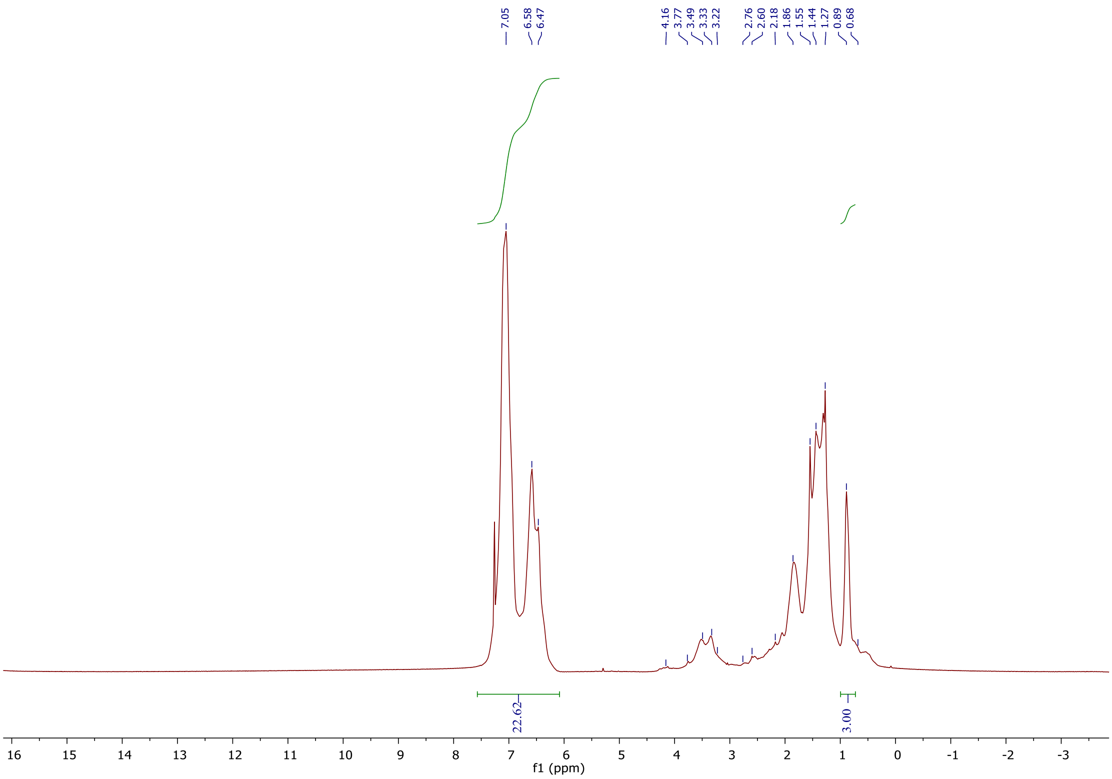

Fig. S128 ^{1}H NMR spectrum (400 MHz, $CDCl_3$) of PS_{82}-*co*-$PHCBI_{18}$. (The fraction of HCBI in copolymers is 1/(22.62/5+1)*100% ≈ 18%. Therefore, the fraction of styrene in copolymer is 82%.)

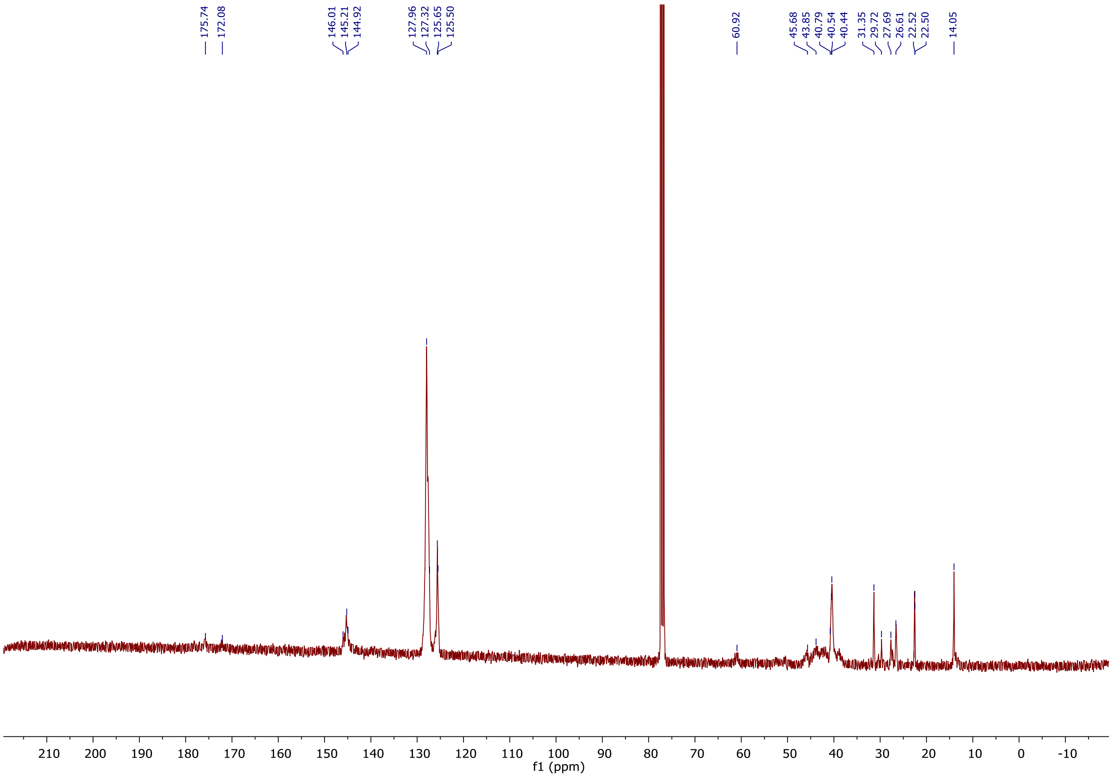

Fig. S129 ^{13}C NMR spectrum (101 MHz, $CDCl_3$) of PS_{82}-*co*-$PHCBI_{18}$.

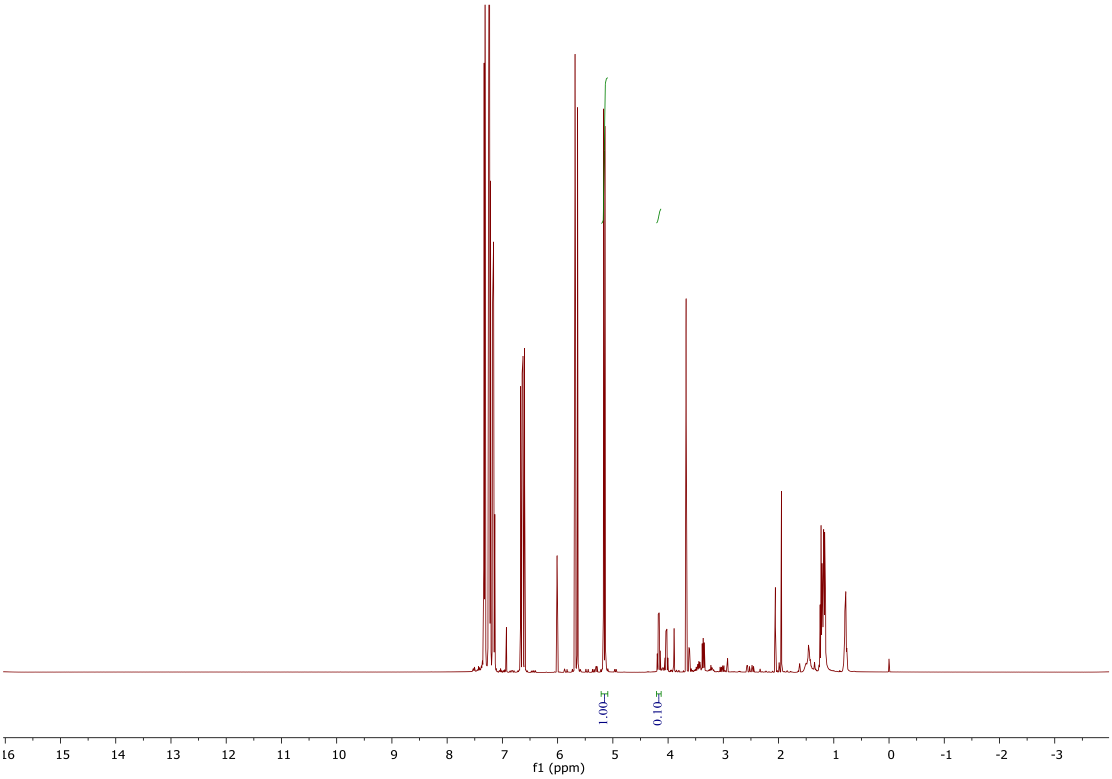

Fig. S130 ^{1}H NMR spectrum (400 MHz, $CDCl_3$) of reaction feed mixture for the synthesis of PS_{75}-*co*-$PHCBI_{25}$. (The fraction of HCBI in feed is (0.10/2)/(0.10/2+1) ≈ 0.05. Therefore, the fraction of styrene in feed is 0.95.)

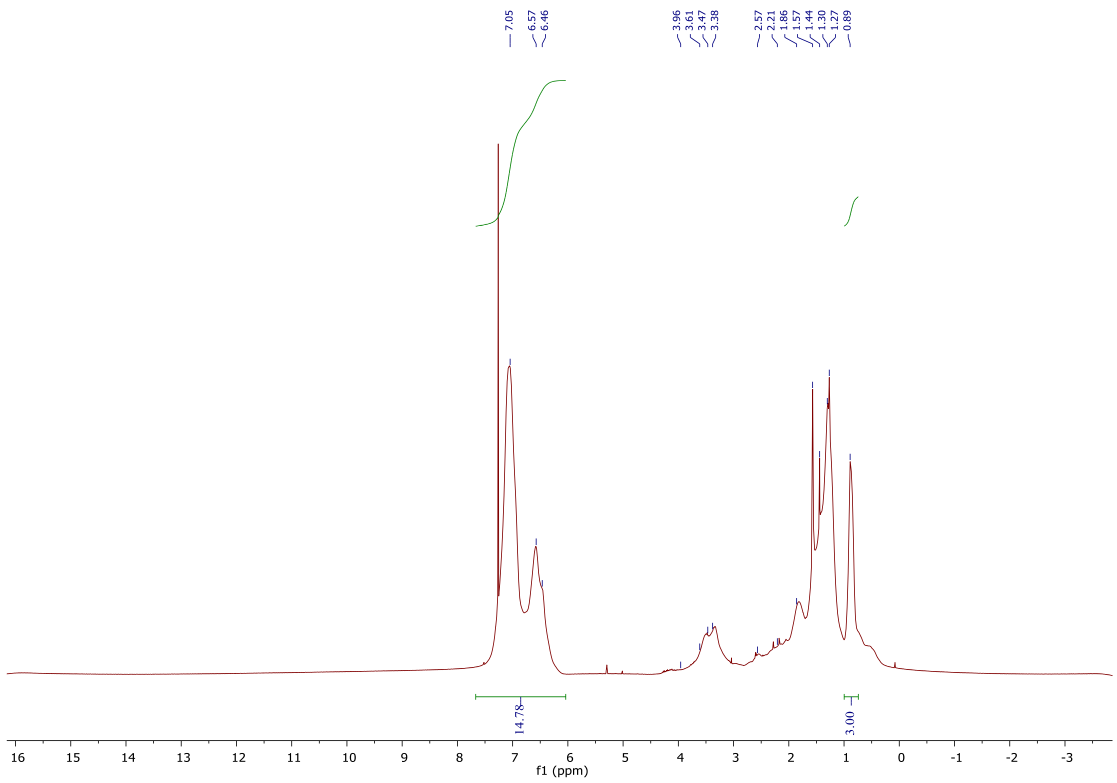

Fig. S131 ^{1}H NMR spectrum (400 MHz, $CDCl_3$) of PS_{75}-*co*-$PHCBI_{25}$. (The fraction of HCBI in copolymers is 1/(14.78/5+1)*100% ≈ 25%. Therefore, the fraction of styrene in copolymer is 75%.)

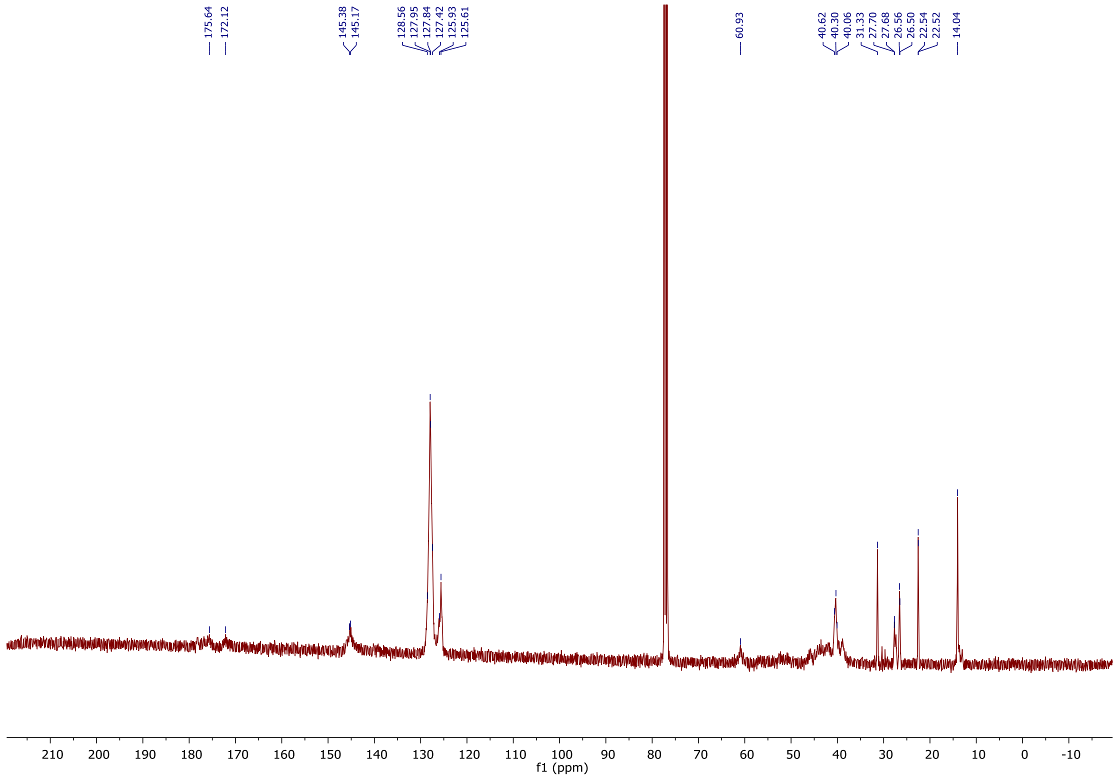

Fig. S132 ^{13}C NMR spectrum (101 MHz, $CDCl_3$) of PS_{75}-*co*-$PHCBI_{25}$.

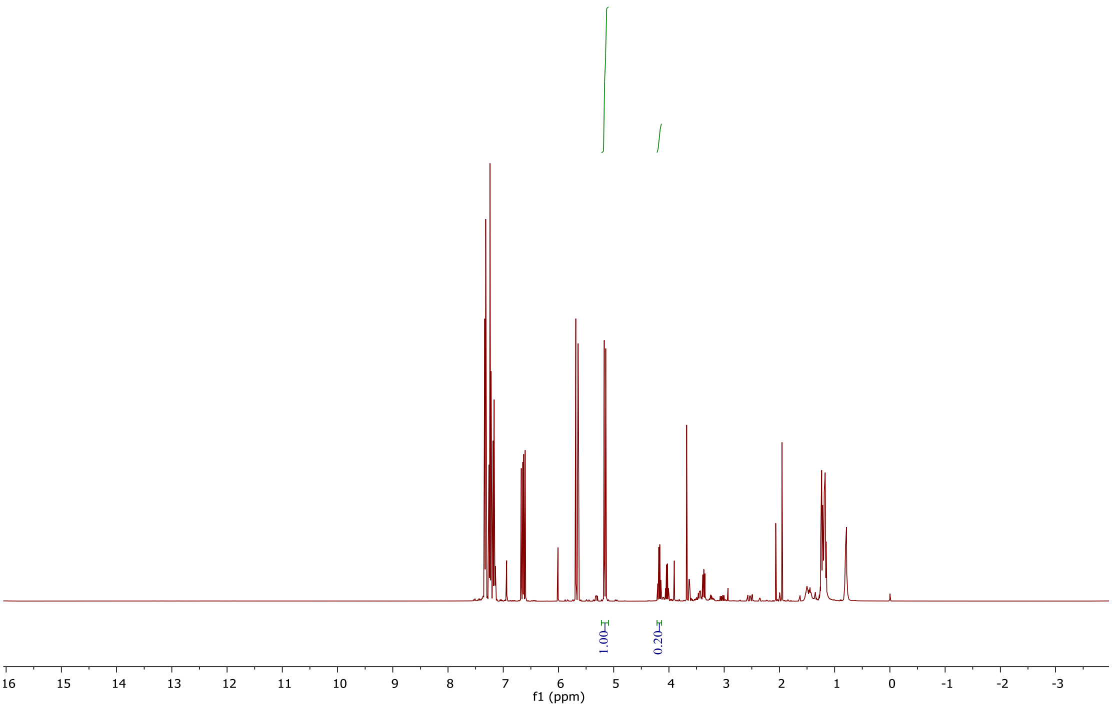

Fig. S133 ^{1}H NMR spectrum (400 MHz, $CDCl_3$) of reaction feed mixture for the synthesis of PS_{60}-*co*-$PHCBI_{40}$. (The fraction of HCBI in feed is (0.20/2)/(0.20/2+1) ≈ 0.09. Therefore, the fraction of styrene in feed is 0.91.)

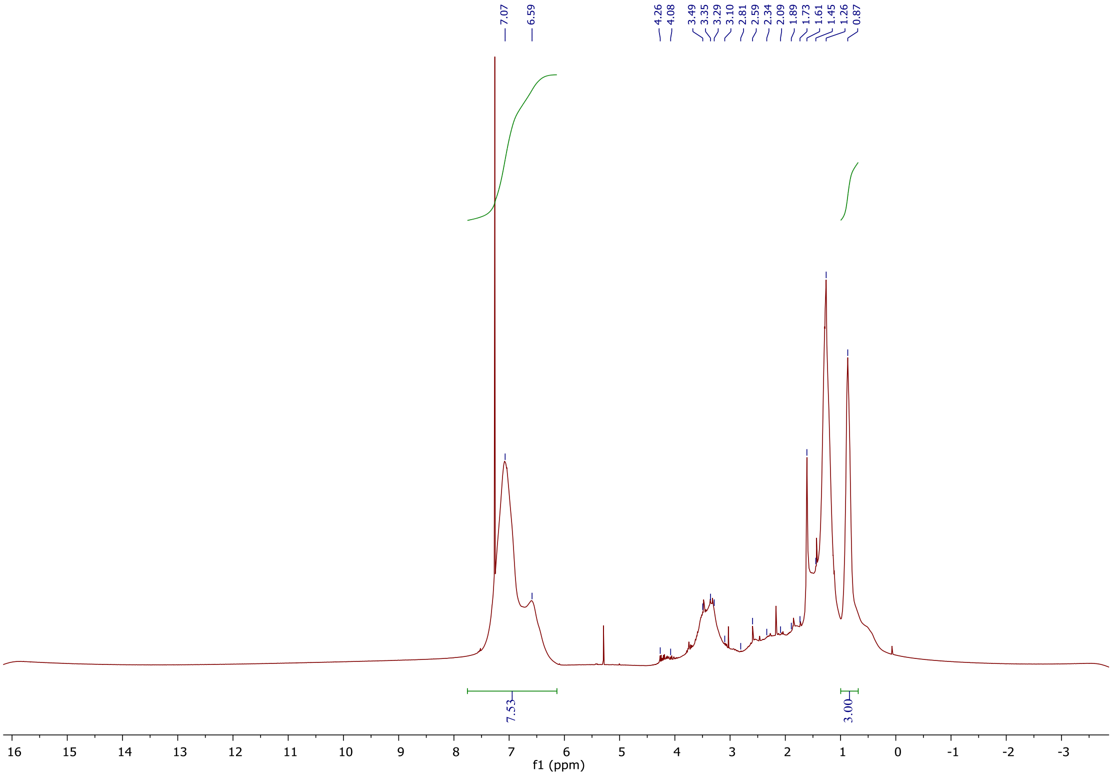

Fig. S134 ^{1}H NMR spectrum (400 MHz, $CDCl_3$) of PS_{60}-*co*-$PHCBI_{40}$. (The fraction of HCBI in copolymers is 1/(7.53/5+1)*100% ≈ 40%. Therefore, the fraction of styrene in copolymer is 60%.)

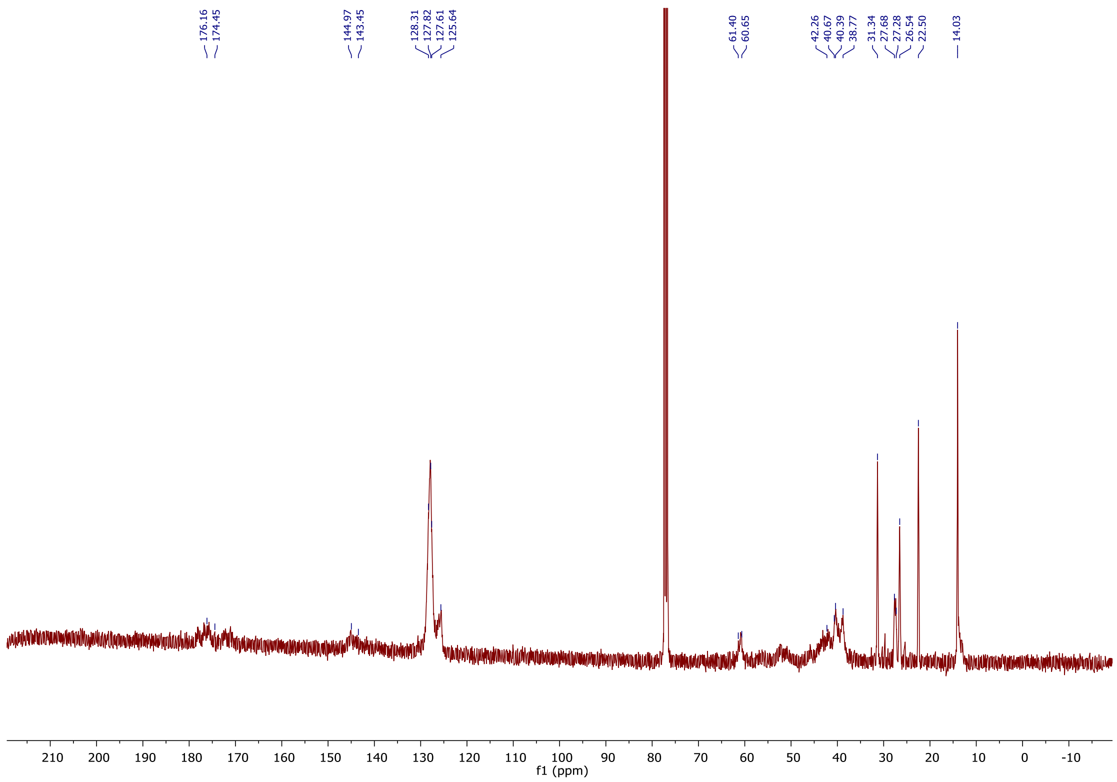

Fig. S135 ^{13}C NMR spectrum (101 MHz, $CDCl_3$) of PS_{60}-*co*-$PHCBI_{40}$.

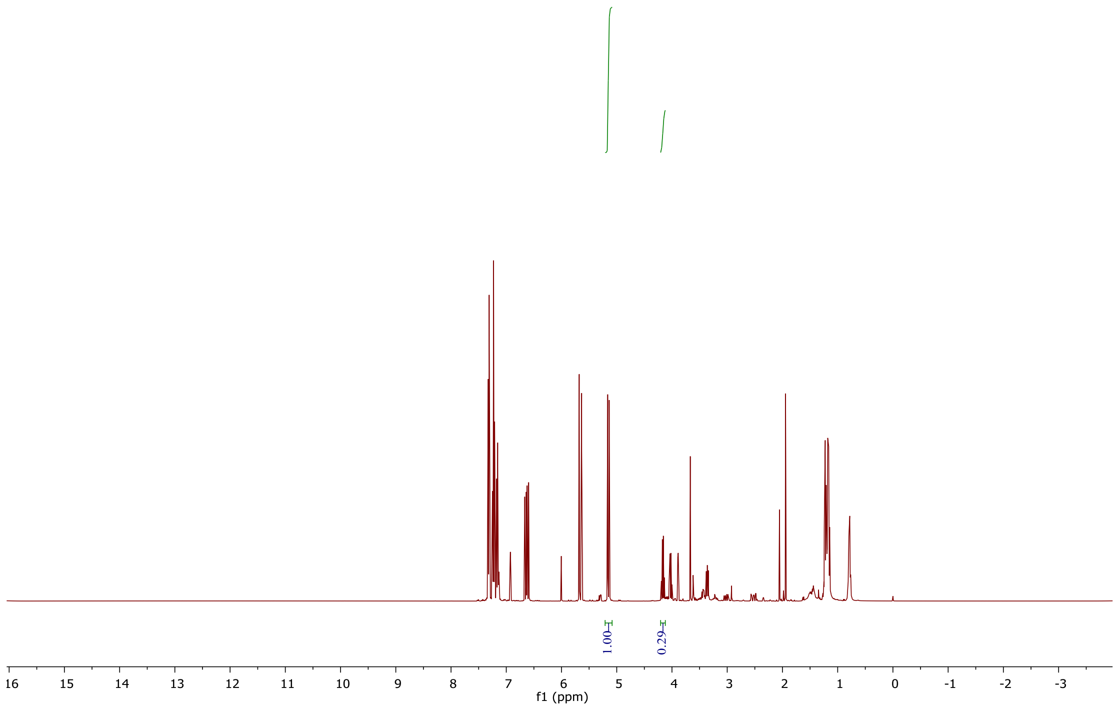

Fig. S136 ^{1}H NMR spectrum (400 MHz, $CDCl_3$) of reaction feed mixture for the synthesis of PS_{55}-*co*-$PHCBI_{45}$. (The fraction of HCBI in feed is (0.29/2)/(0.29/2+1) ≈ 0.13. Therefore, the fraction of styrene in feed is 0.87.)

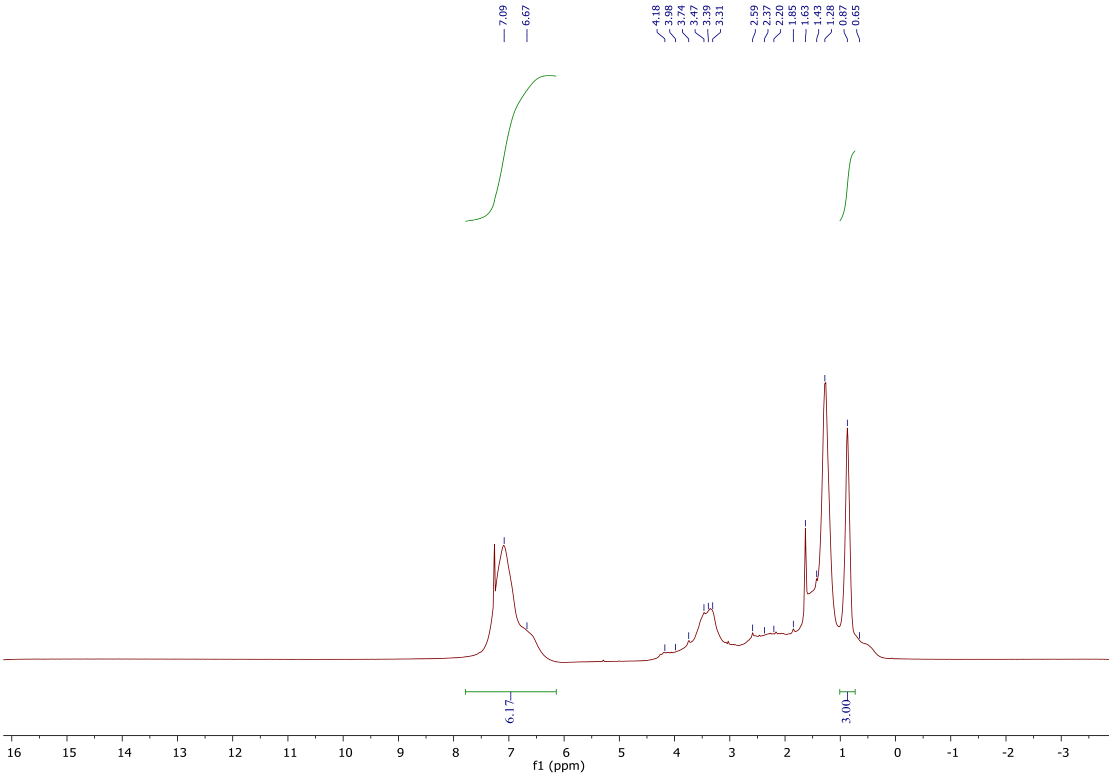

Fig. S137 ^{1}H NMR spectrum (400 MHz, $CDCl_3$) of PS_{55}-*co*-$PHCBI_{45}$. (The fraction of HCBI in copolymers is 1/(6.17/5+1)*100% ≈ 45%. Therefore, the fraction of styrene in copolymer is 55%.)

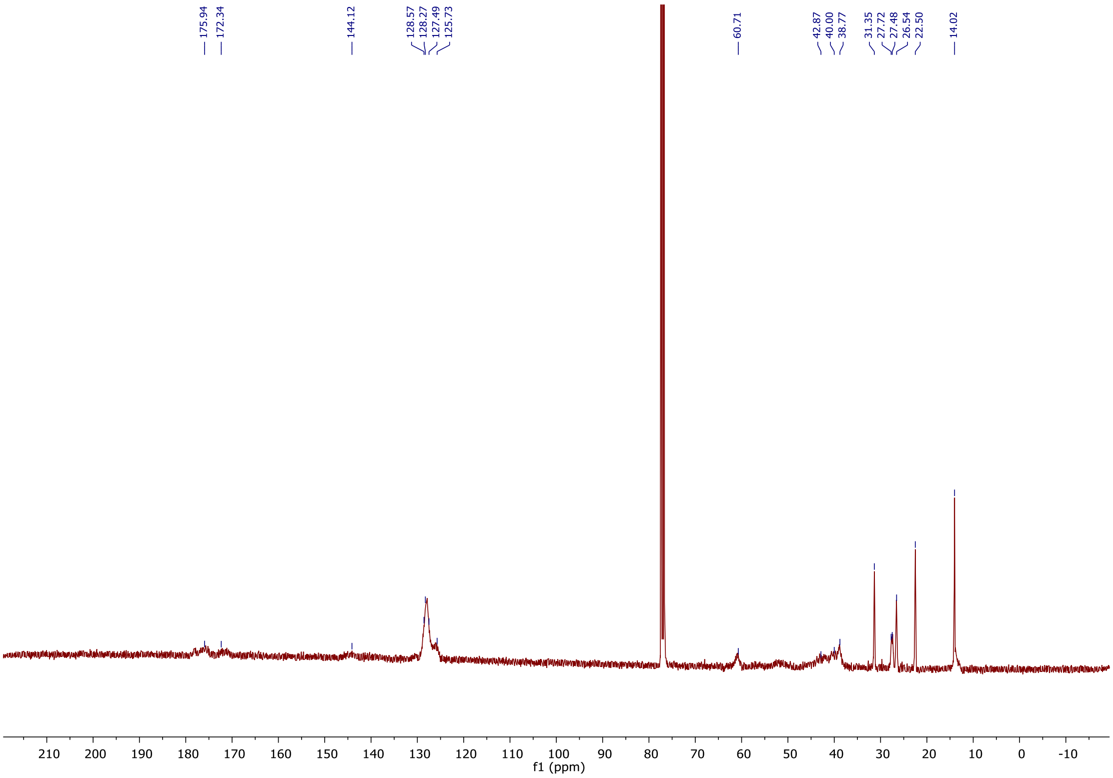

Fig. S138 ^{13}C NMR spectrum (101 MHz, $CDCl_3$) of PS_{55}-*co*-$PHCBI_{45}$.

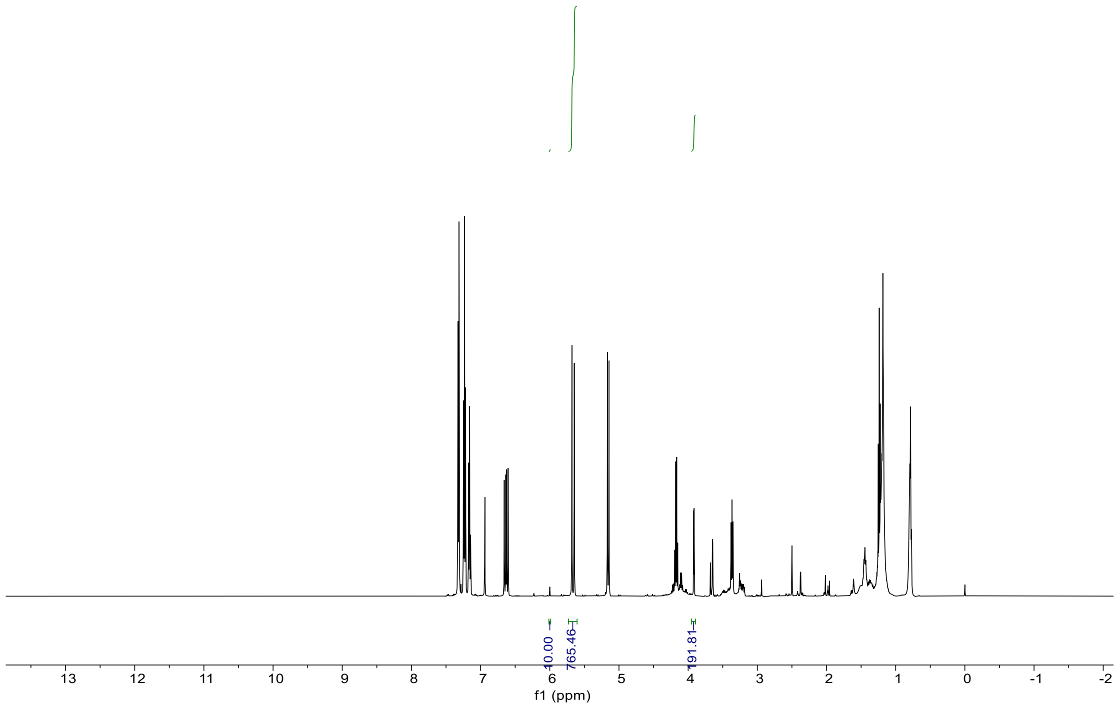

Fig. S139 ^{1}H NMR spectrum (500 MHz, $CDCl_3$) of reaction monomer feed mixture for the synthesis of PS_{50}-*co*-$PHCBI_{50}$. (The fraction of HCBI in feed is (191.81/(191.81+765.46) ≈ 0.2. Therefore, the fraction of styrene in feed is 0.8.)

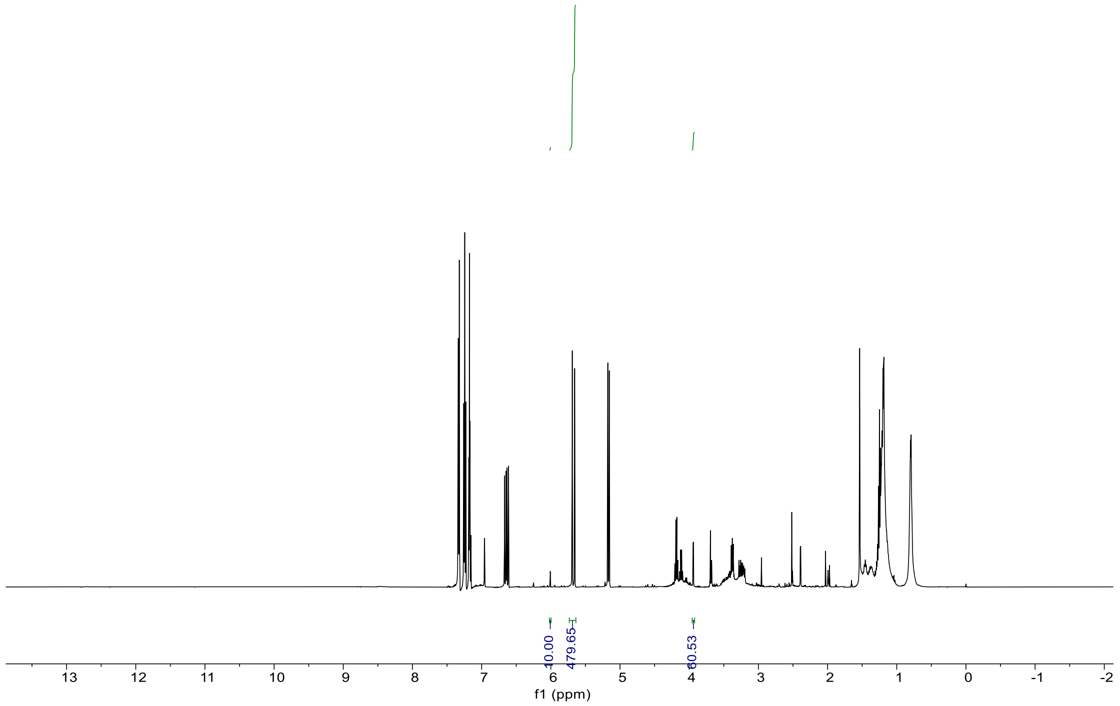

Fig. S140 ^{1}H NMR spectrum (500 MHz, $CDCl_3$) of monomers conversion for the synthesis of PS_{50}-*co*-$PHCBI_{50}$. (Monomers conversion after polymerization is ((191.81+765.46)-(479.65+60.53))/(191.81+765.46) ≈ 44%.)

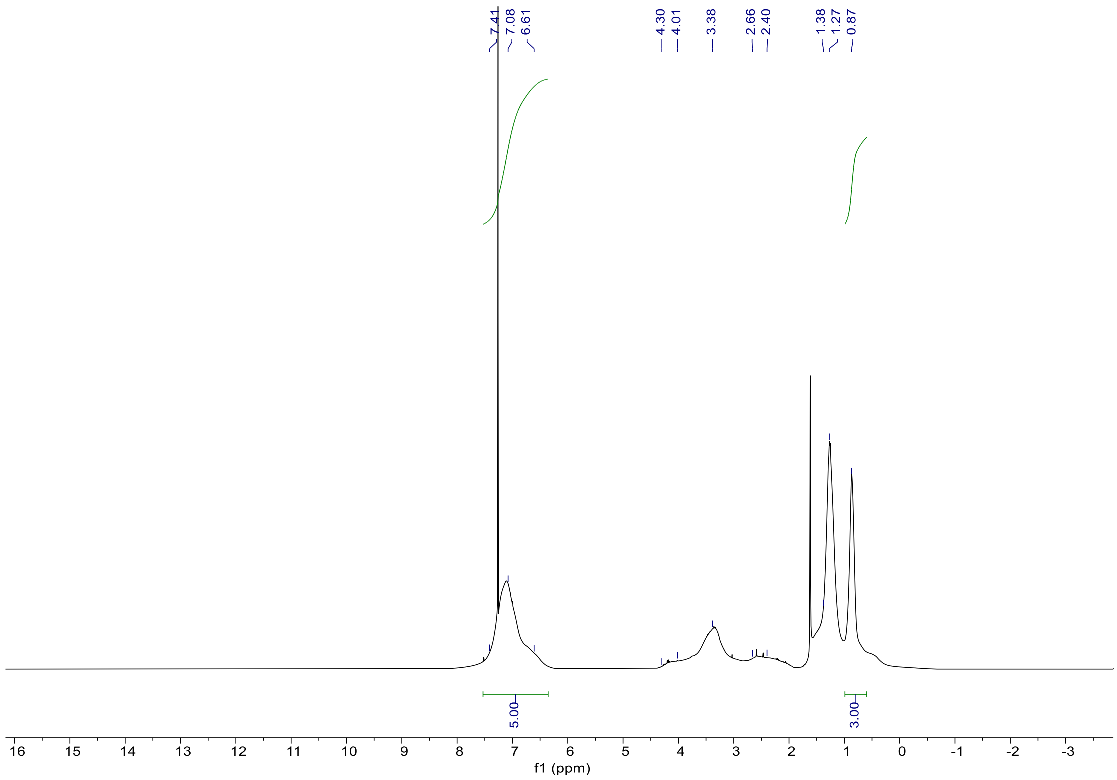

Fig. S141 ^{1}H NMR spectrum (400 MHz, $CDCl_3$) of PS_{50}-*co*-$PHCBI_{50}$. (The fraction of HCBI in copolymers is 1/(5/5+1)*100% ≈ 50%. Therefore, the fraction of styrene in copolymer is 50%.)

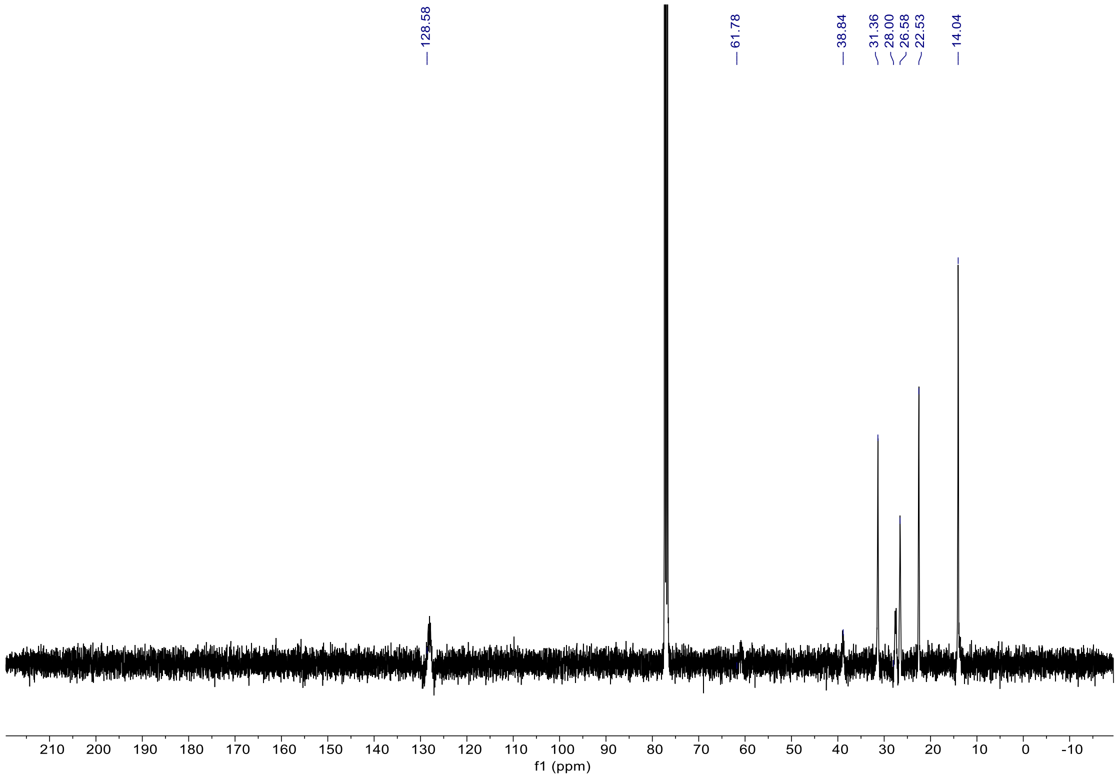

Fig. S142 ^{13}C NMR spectrum (101 MHz, $CDCl_3$) of PS_{50}-*co*-$PHCBI_{50}$.

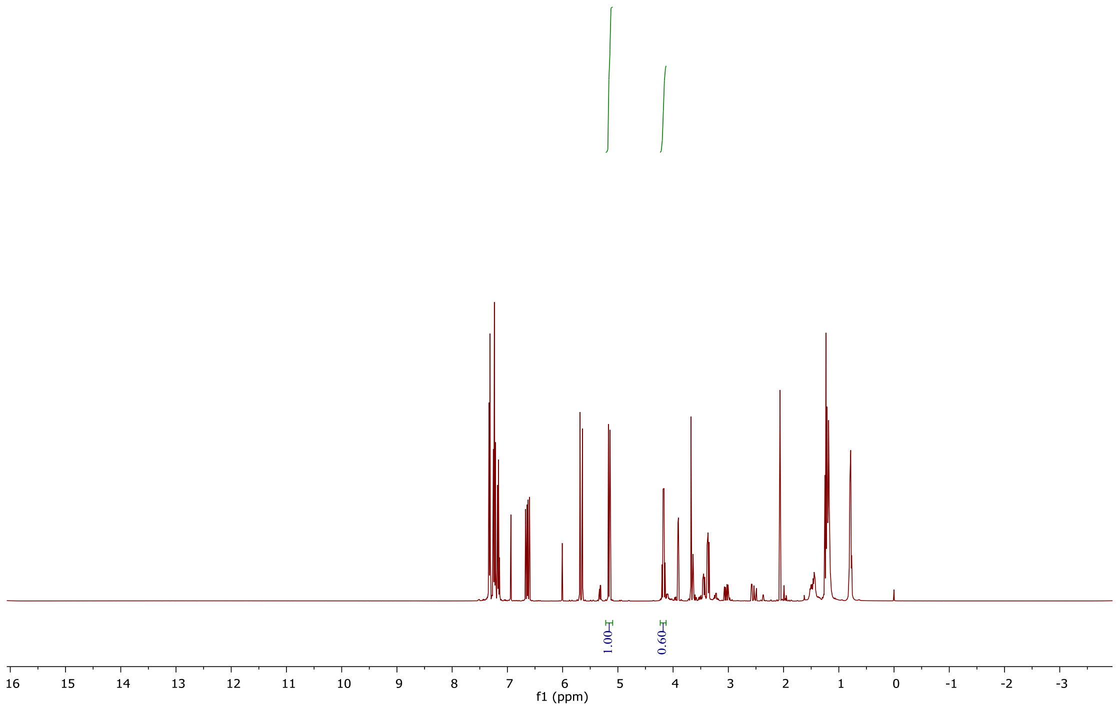

Fig. S143 ^{1}H NMR spectrum (400 MHz, $CDCl_3$) of reaction feed mixture for the synthesis of PS_{49}-*co*-$PHCBI_{51}$. (The fraction of HCBI in feed is (0.60/2)/(0.60/2+1) ≈ 0.23. Therefore, the fraction of styrene in feed is 0.77.)

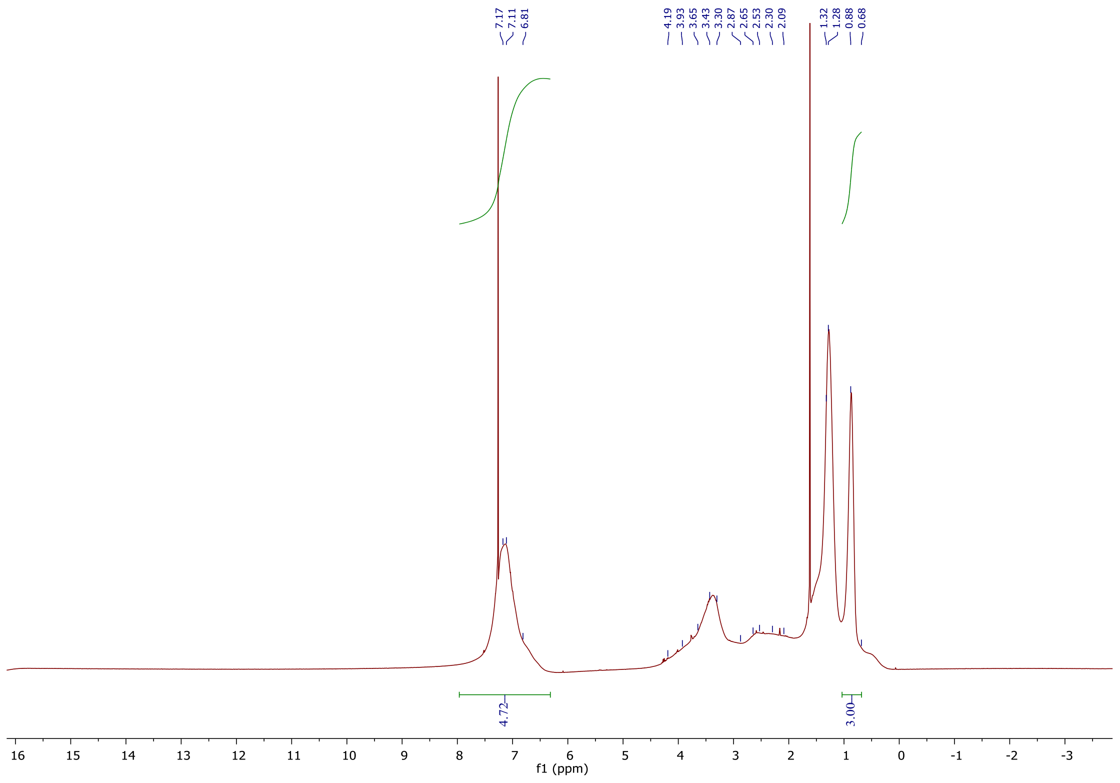

Fig. S144 ^{1}H NMR spectrum (400 MHz, $CDCl_3$) of PS_{49}-*co*-$PHCBI_{51}$. (The fraction of HCBI in copolymers is 1/(4.72/5+1)*100% ≈ 51%. Therefore, the fraction of styrene in copolymer is 49%.)

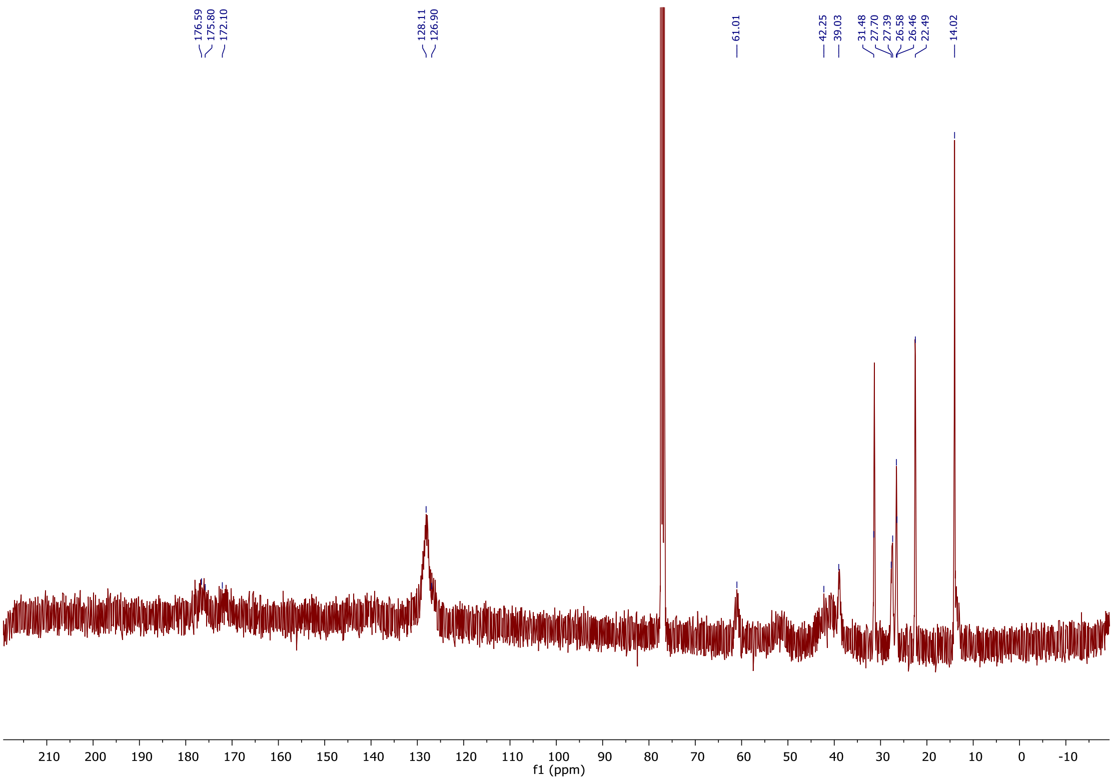

Fig. S145 ^{13}C NMR spectrum (101 MHz, $CDCl_3$) of PS_{49}-*co*-$PHCBI_{51}$.

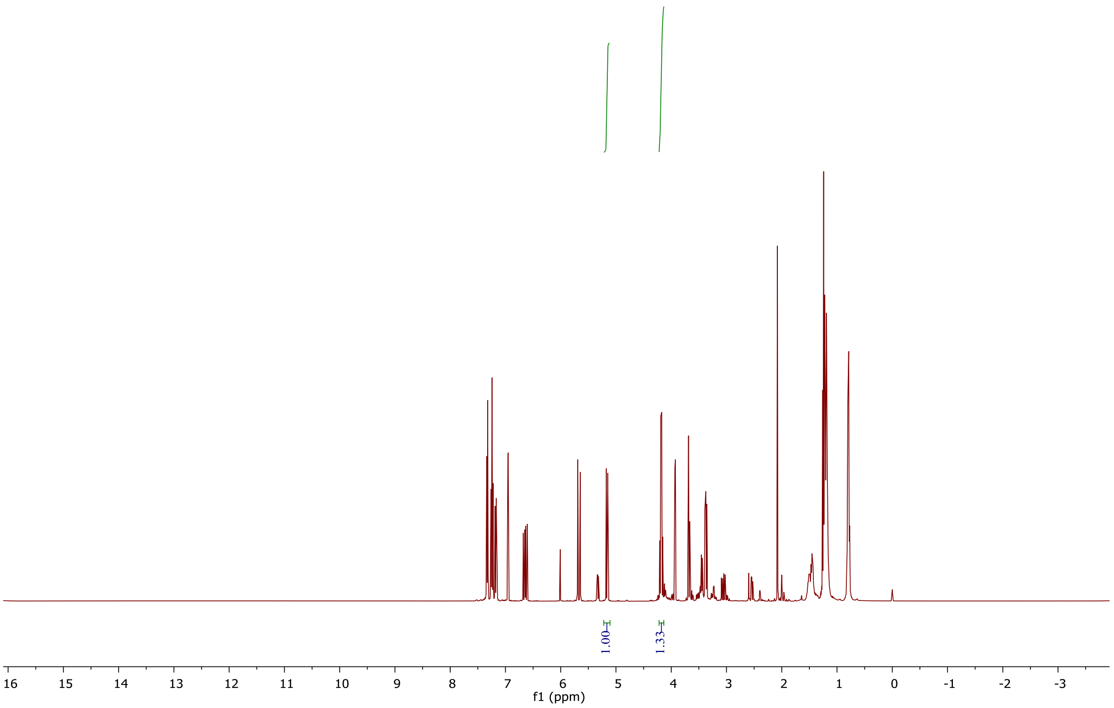

Fig. S146 ^{1}H NMR spectrum (400 MHz, $CDCl_3$) of reaction feed mixture for the synthesis of PS_{45}-*co*-$PHCBI_{55}$. (The fraction of HCBI in feed is (1.33/2)/(1.33/2+1) ≈ 0.4. Therefore, the fraction of styrene in feed is 0.6.)

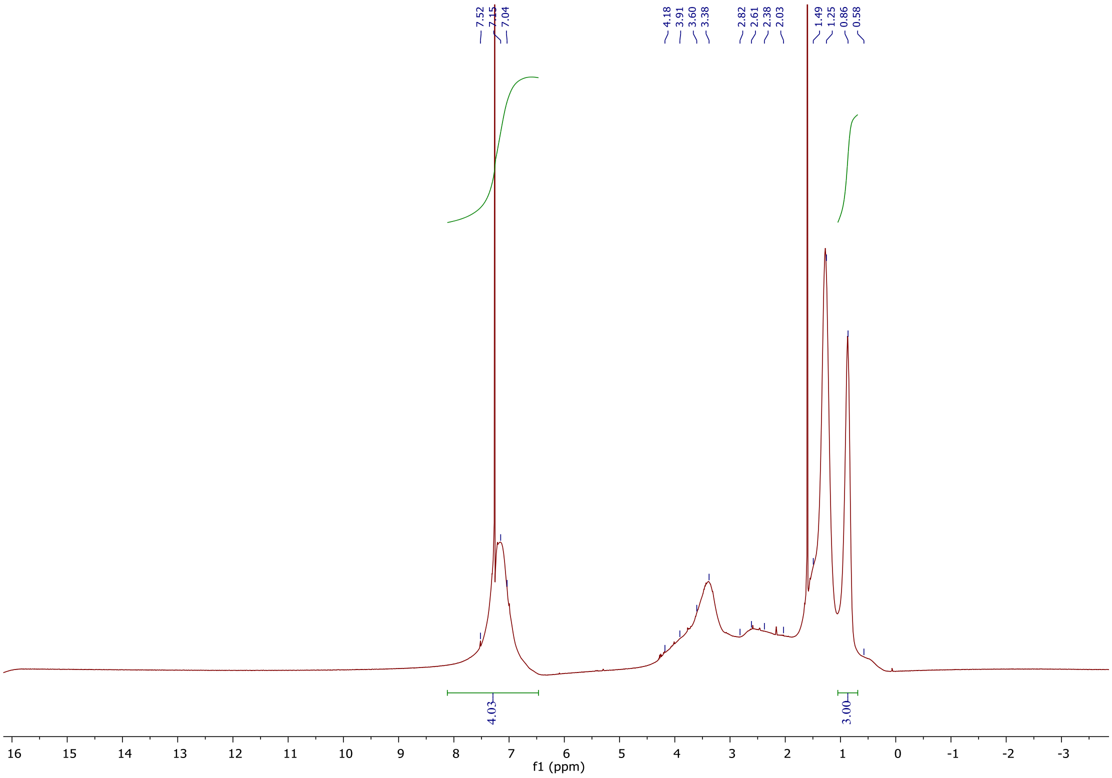

Fig. S147 ^{1}H NMR spectrum (400 MHz, $CDCl_3$) of PS_{45}-*co*-$PHCBI_{55}$. (The fraction of HCBI in copolymers is 1/(4.03/5+1)*100% ≈ 55%. Therefore, the fraction of styrene in copolymer is 45%).

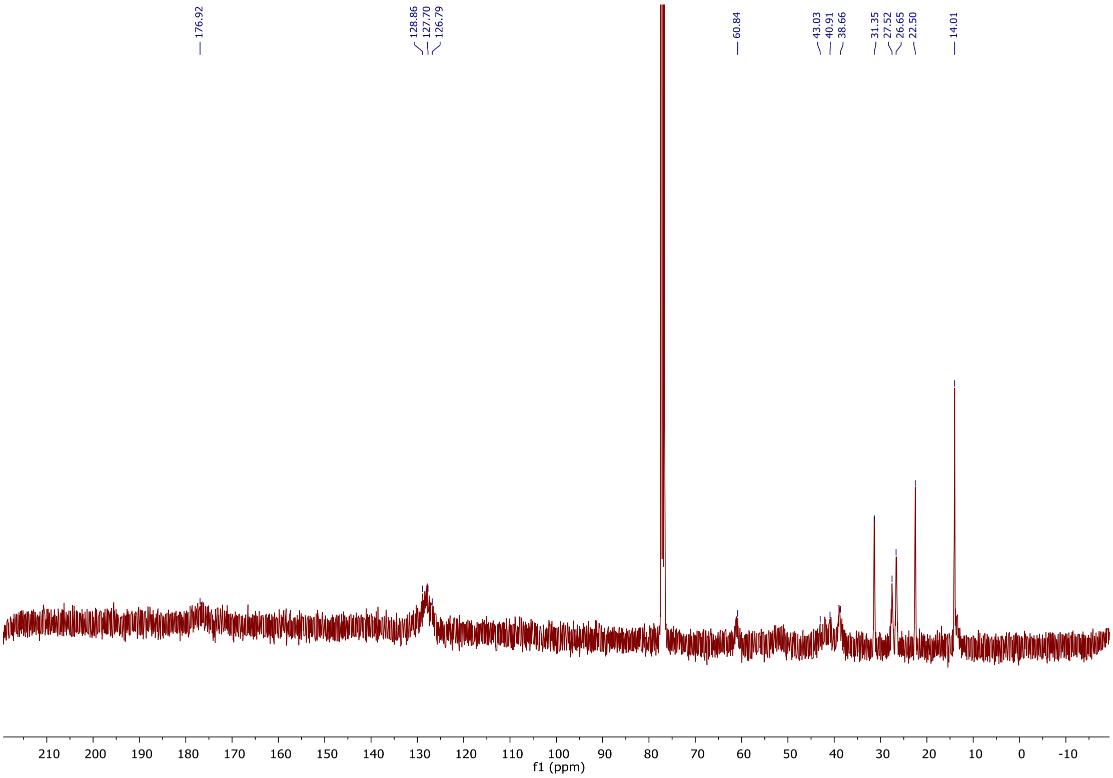

Fig. S148 ^{13}C NMR spectrum (101 MHz, $CDCl_3$) of PS_{45}-*co*-$PHCBI_{55}$.

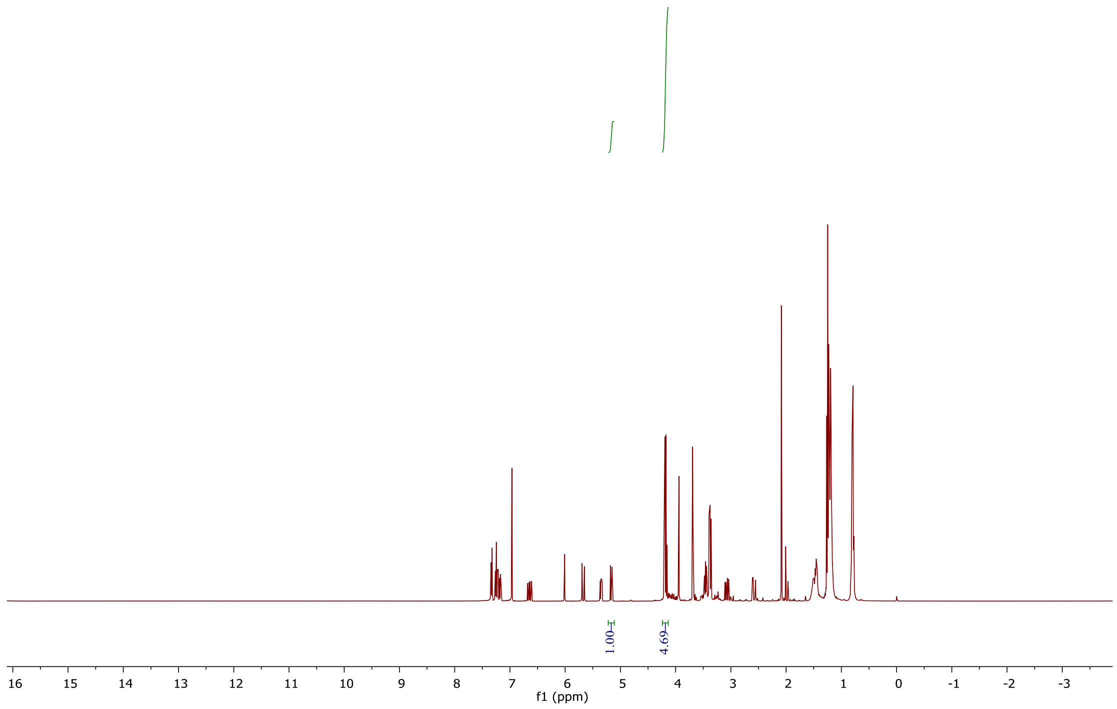

Fig. S149 ^{1}H NMR spectrum (400 MHz, $CDCl_3$) of reaction feed mixture for the synthesis of PS_{38}-*co*-$PHCBI_{62}$. (The fraction of HCBI in feed is (4.69/2)/(4.69/2+1) ≈ 0.7. Therefore, the fraction of styrene in feed is 0.3.)

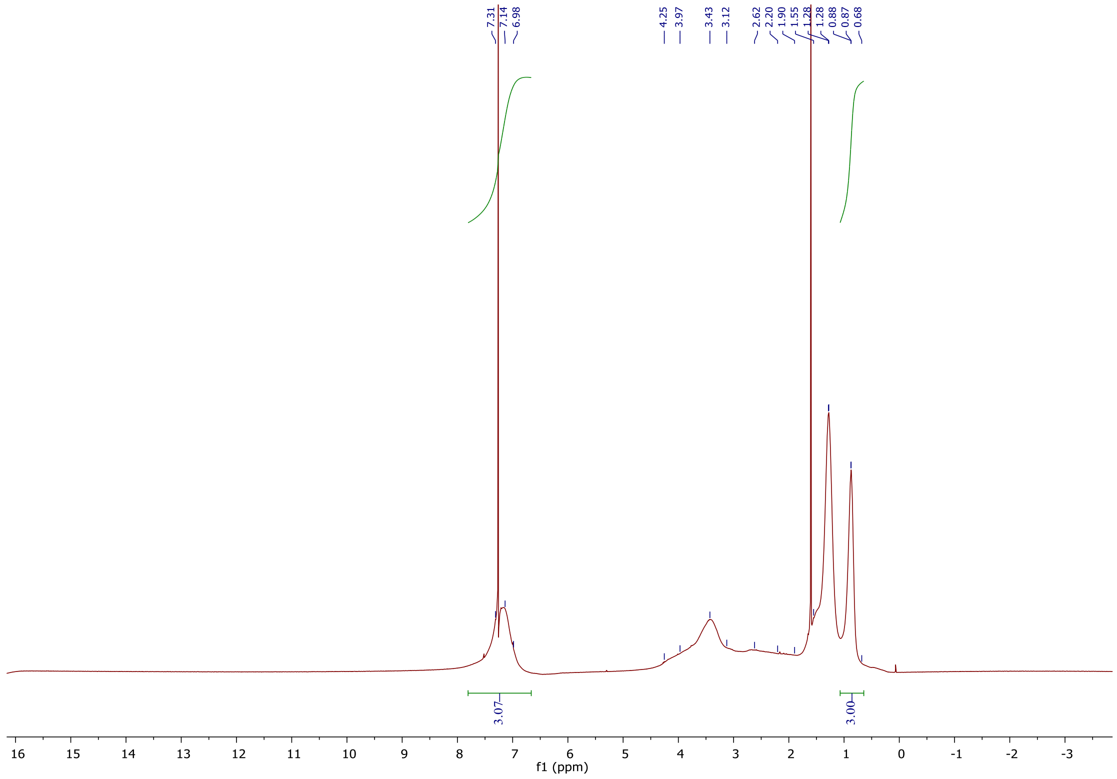

Fig. S150 ^{1}H NMR spectrum (400 MHz, $CDCl_3$) of PS_{38}-*co*-$PHCBI_{62}$. (The fraction of HCBI in copolymers is 1/(3.07/5+1)*100% ≈ 62%. Therefore, the fraction of styrene in copolymer is 38%.)

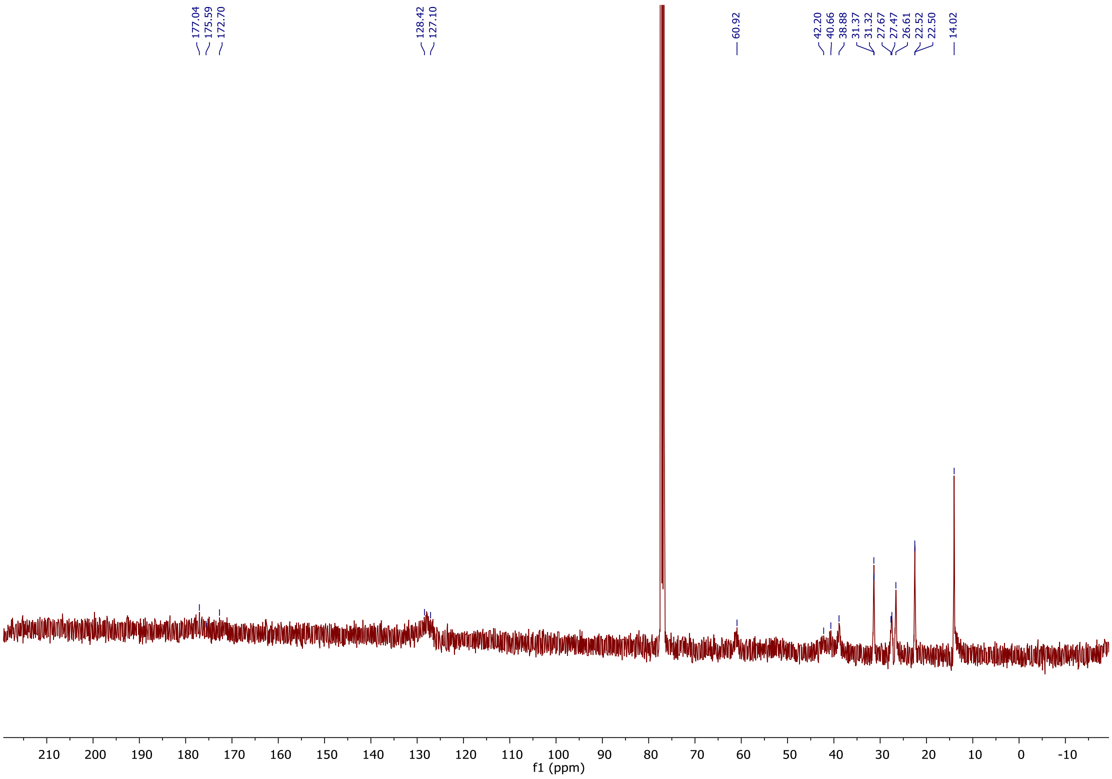

Fig. S151 ^{13}C NMR spectrum (101 MHz, $CDCl_3$) of PS_{38}-*co*-$PHCBI_{62}$.

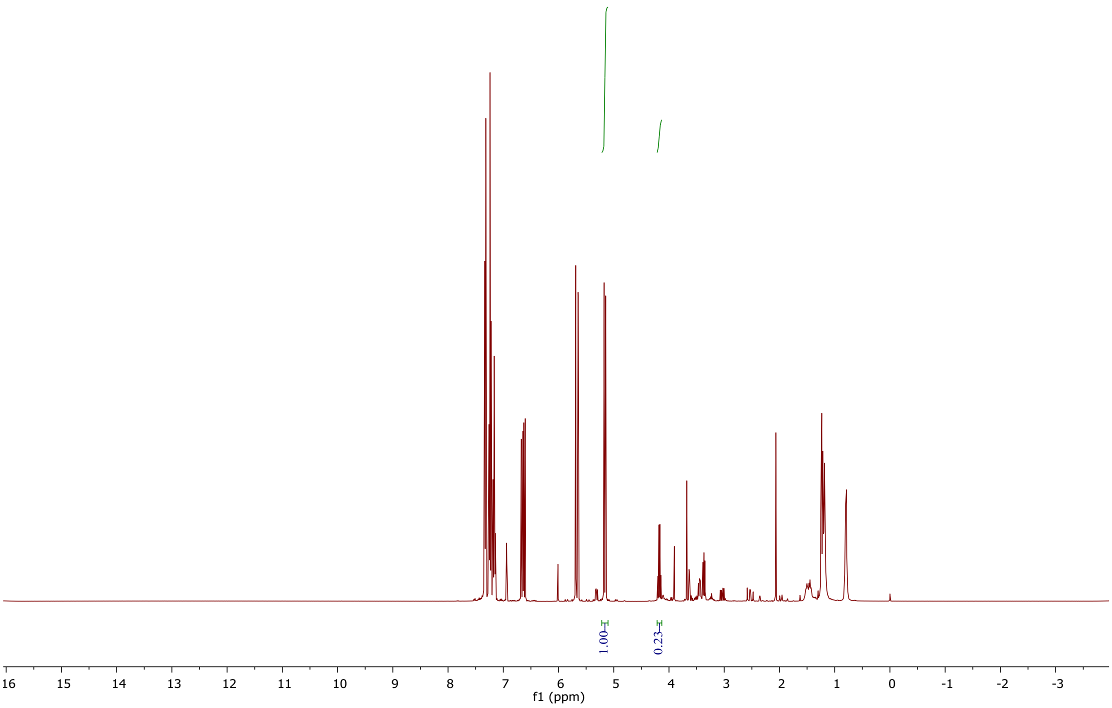

Fig. S152 ^{1}H NMR spectrum (400 MHz, $CDCl_3$) of reaction feed mixture for the synthesis of PS_{73}-*co*-$PHCBI_{27}$. (The fraction of HCBI in feed is (0.23/2)/(0.23/2+1) ≈ 0.1. Therefore, the fraction of styrene in feed is 0.9.)

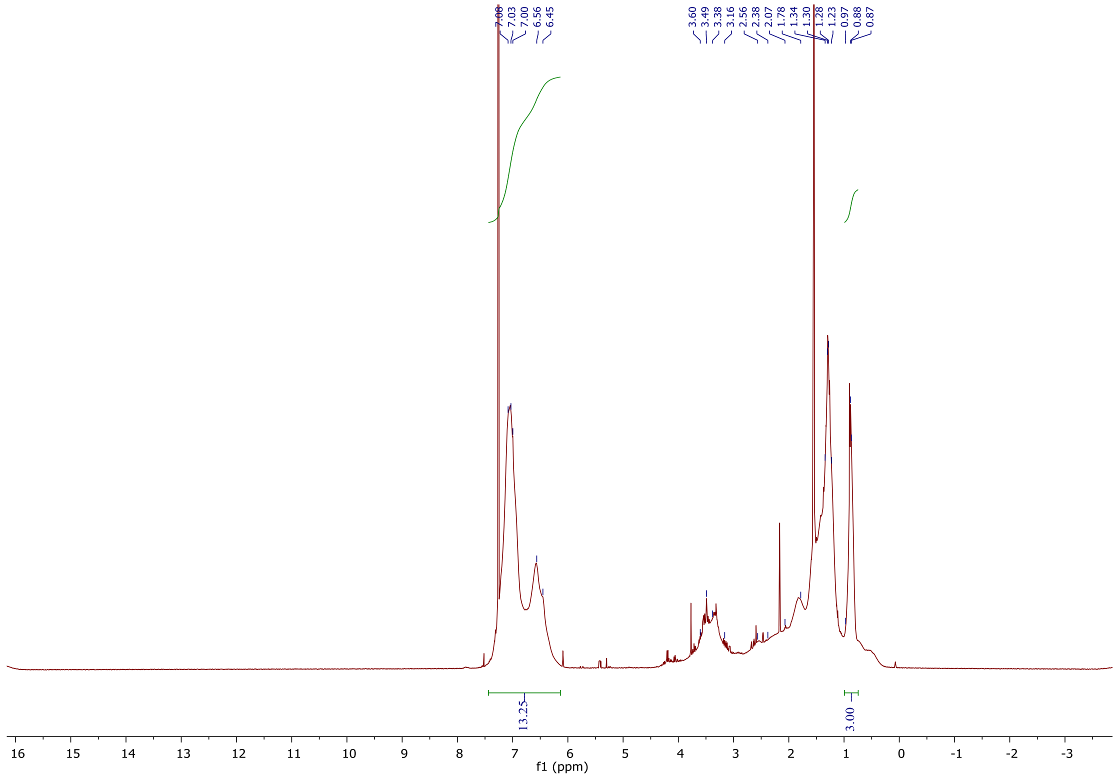

Fig. S153 ^{1}H NMR spectrum (400 MHz, $CDCl_3$) of PS_{73}-*co*-$PHCBI_{27}$.(The fraction of HCBI in copolymers is 1/(13.25/5+1)*100% ≈ 27%. Therefore, the fraction of styrene in copolymer is 73%.)

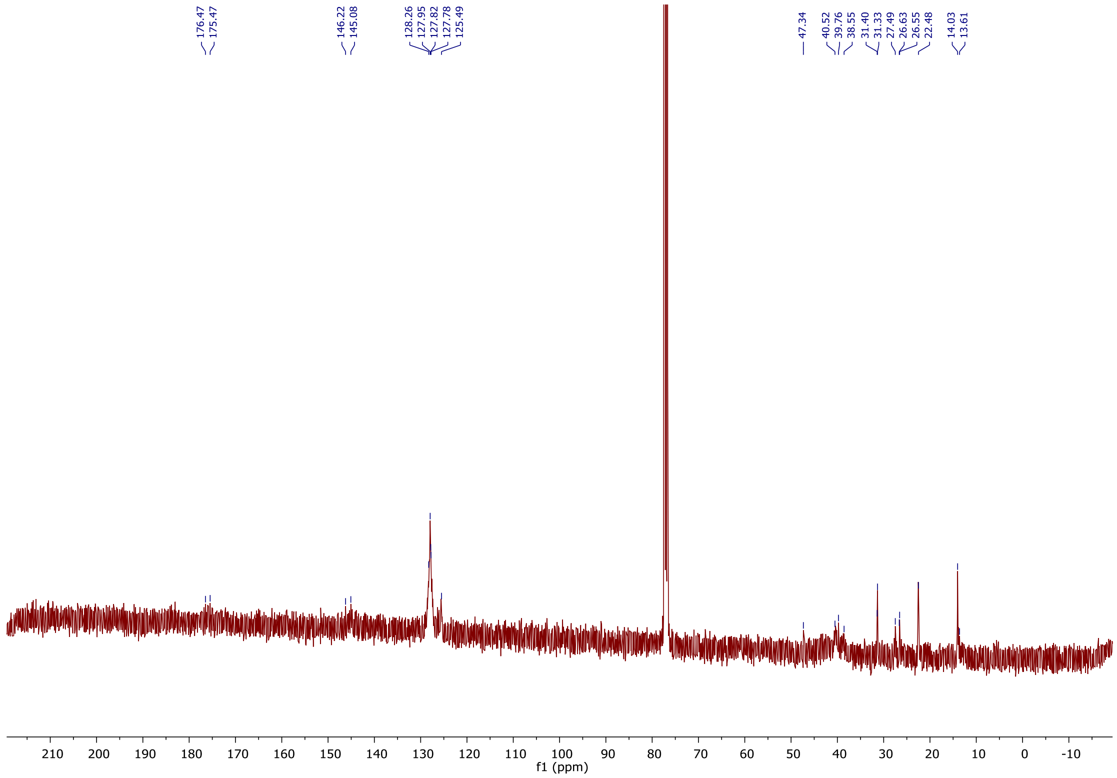

Fig. S154 ^{13}C NMR spectrum (101 MHz, $CDCl_3$) of PS_{73}-*co*-$PHCBI_{27}$.

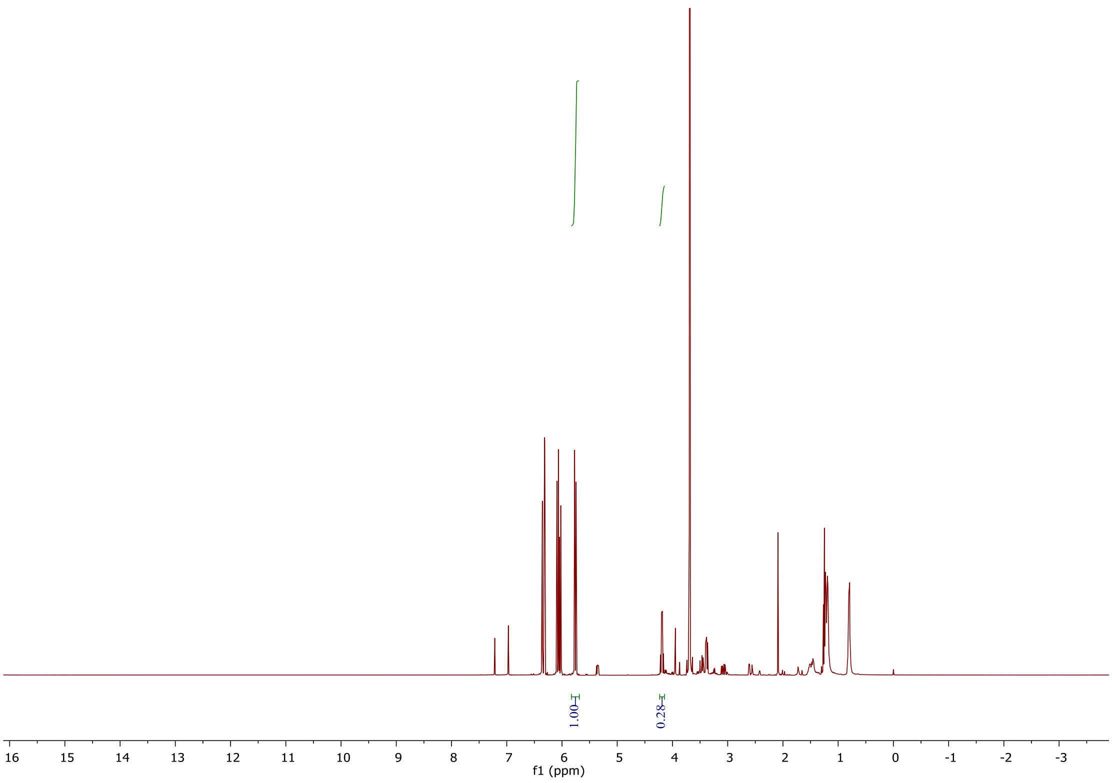

Fig. S155 ^{1}H NMR spectrum (400 MHz, $CDCl_3$) of reaction feed mixture for the synthesis of PMA_{87}-*co*-$PHCBI_{13}$. (The fraction of HCBI in feed is (0.28/2)/(0.28/2+1) ≈ 0.12. Therefore, the fractionof methyl acrylate in feed is 0.88.)

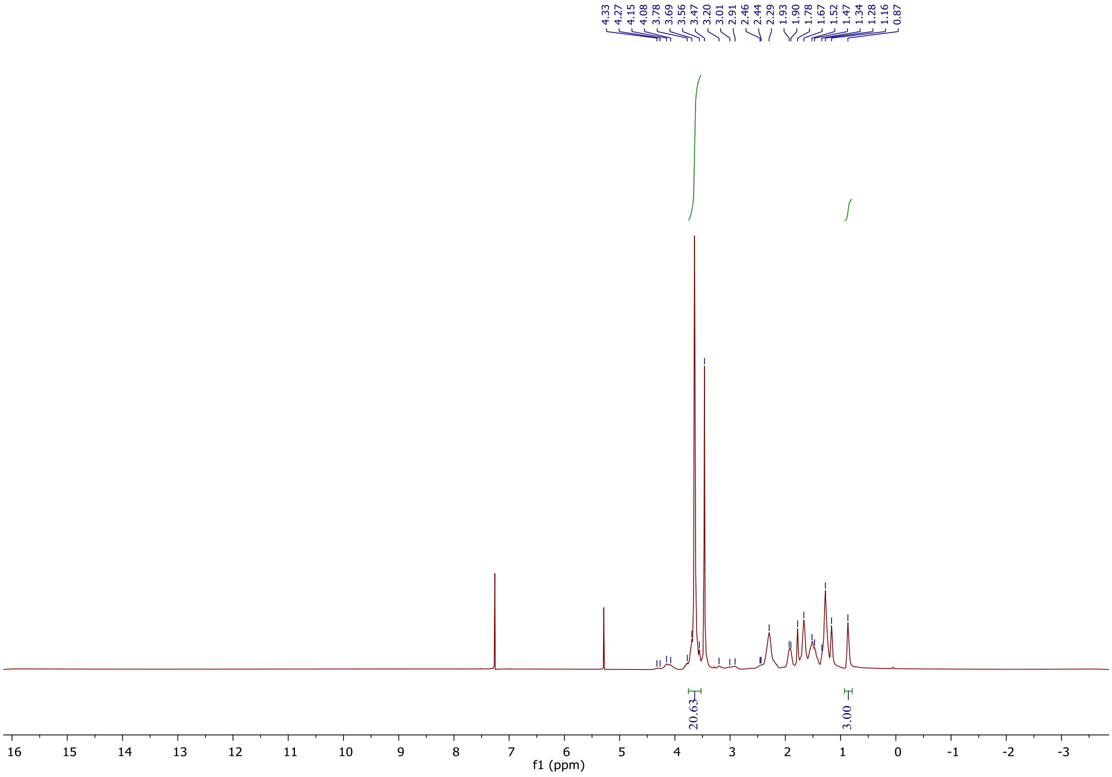

Fig. S156 ^{1}H NMR spectrum (400 MHz, $CDCl_3$) of PMA_{87}-*co*-$PHCBI_{13}$. (The fraction of HCBI in copolymers is 1/(20.63/3+1)*100% ≈ 13%. Therefore, the fraction of methyl acrylate in copolymer is 87%.)

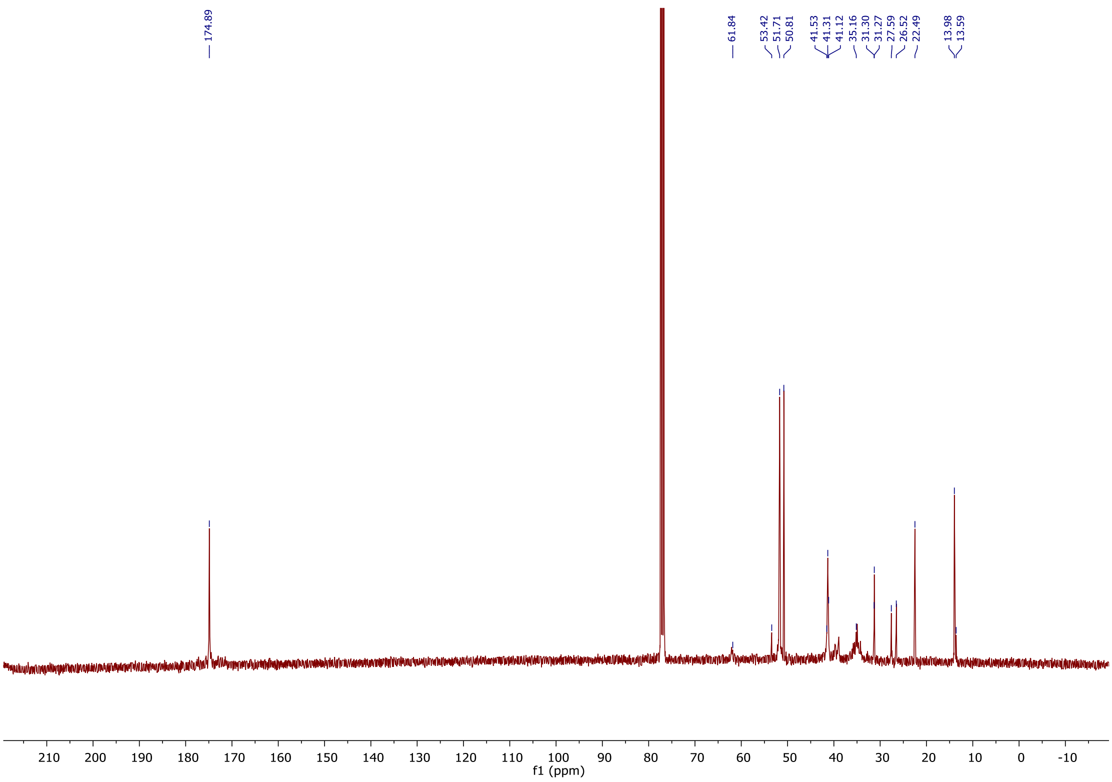

Fig. S157 ^{13}C NMR spectrum (101 MHz, $CDCl_3$) of PMA$_{87}$-*co*-PHCBI$_{13}$.

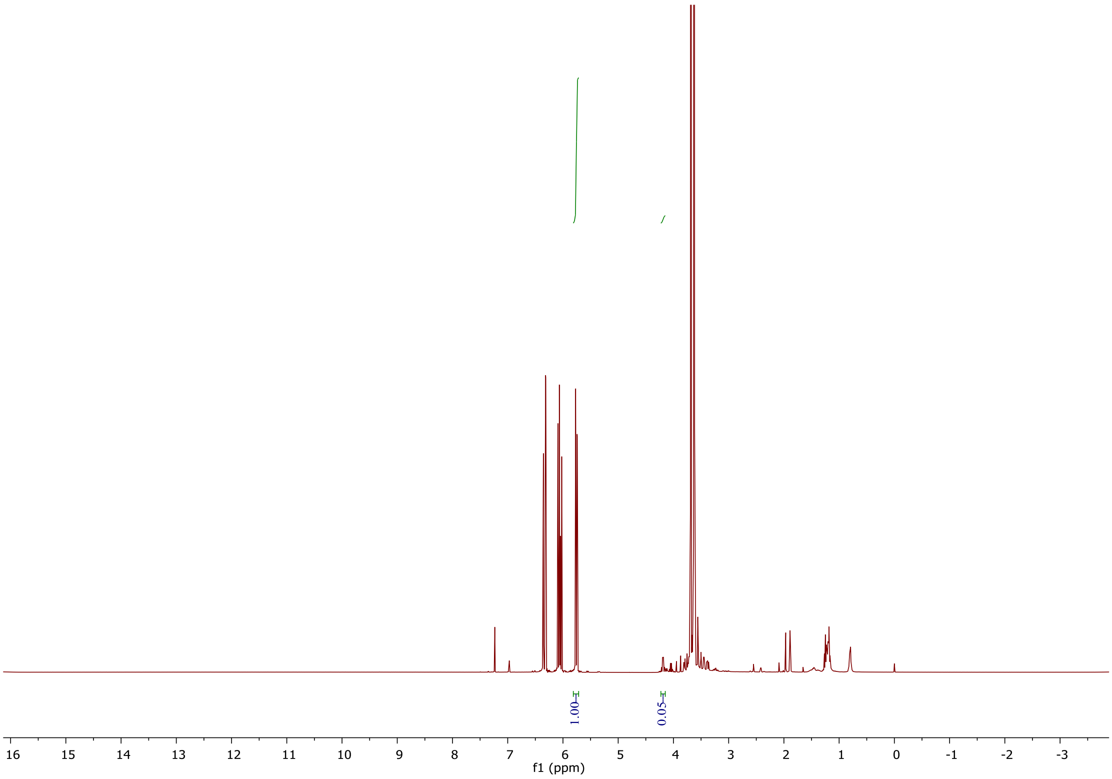

Fig. S158 ^{1}H NMR spectrum (400 MHz, $CDCl_3$) of reaction feed mixture for the synthesis of PMA$_{97}$-*co*-PHCBI$_3$. (The fraction of HCBI in feed is (0.05/2)/(0.05/2+1) ≈ 0.02. Therefore, the fraction of methyl acrylate in feed is 0.98.)

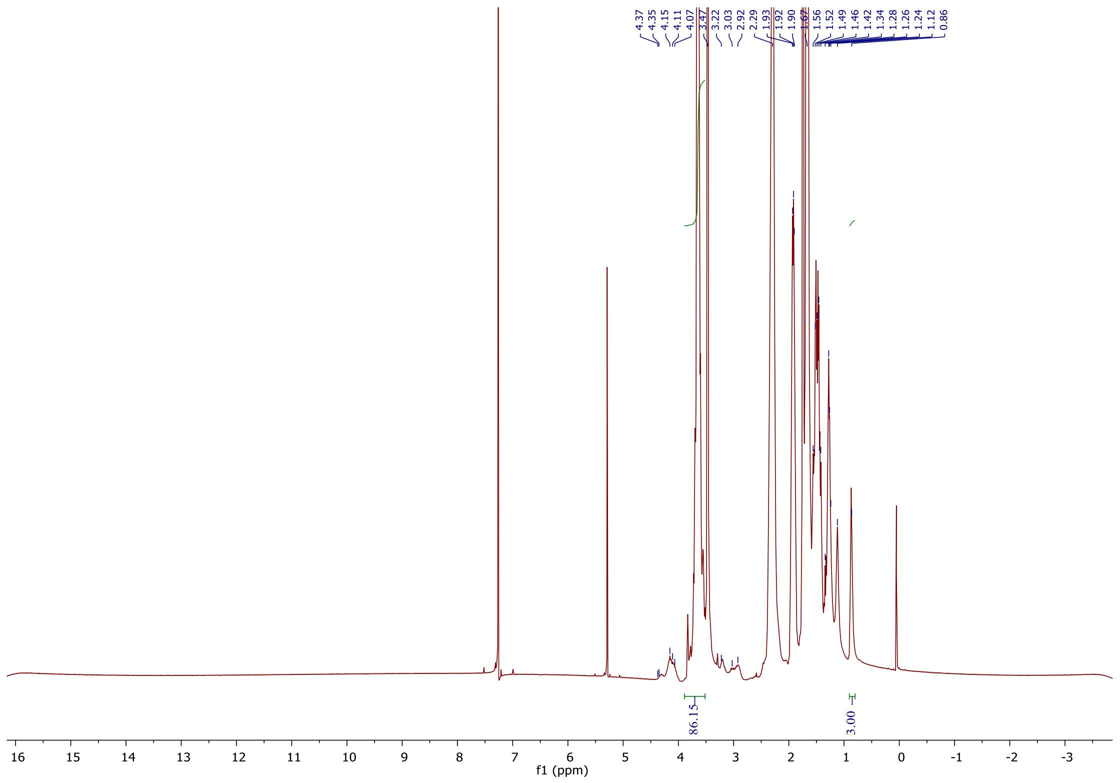

Fig. S159 ^{1}H NMR spectrum (400 MHz, $CDCl_3$) of PMA_{97}-*co*-$PHCBI_3$. (The fraction of HCBI in copolymers is 1/(86.15/3+1)*100% ≈ 3%, therefore, the fraction of methyl acrylate in copolymer is 97%.)

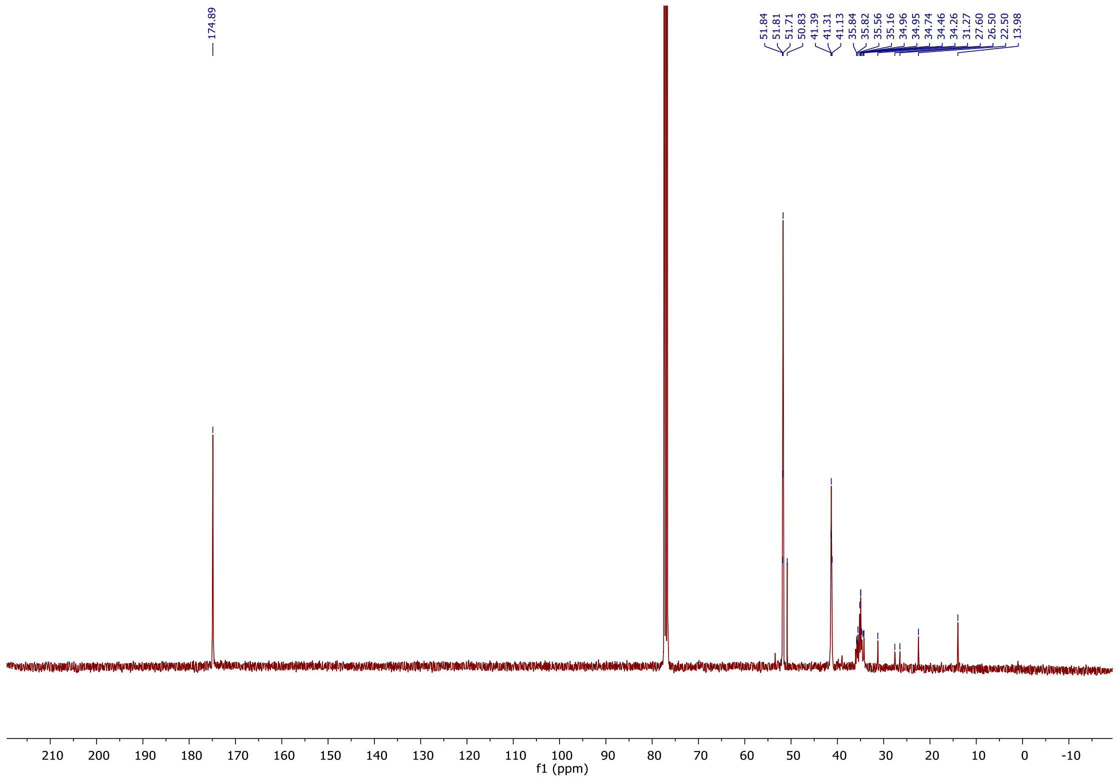

Fig. S160 ^{13}C NMR spectrum (101 MHz, $CDCl_3$) of PMA_{97}-*co*-$PHCBI_3$.

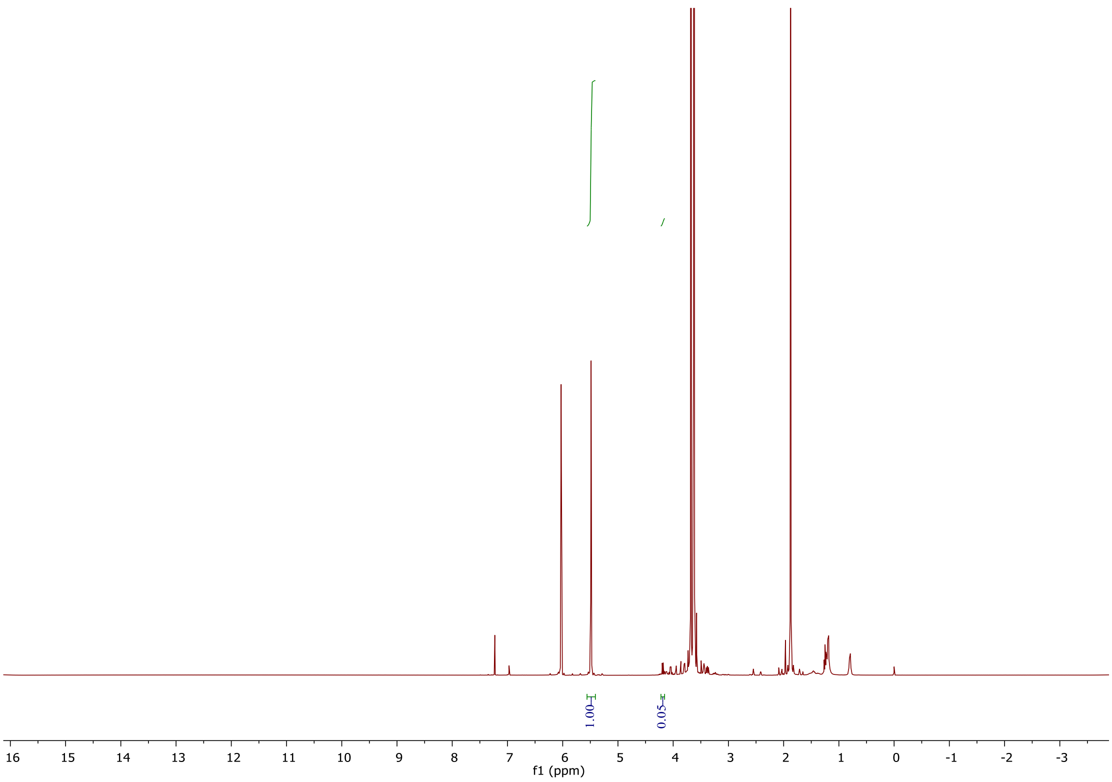

Fig. S161 ^{1}H NMR spectrum (400 MHz, $CDCl_3$) of reaction feed mixture for the synthesis of $PMMA_{98}$-*co*-$PHCBI_2$. (The fraction of HCBI in feed is (0.05/2)/(0.05/2+1) ≈ 0.02. Therefore, the fraction of methyl methacrylate in feed is 0.98.)

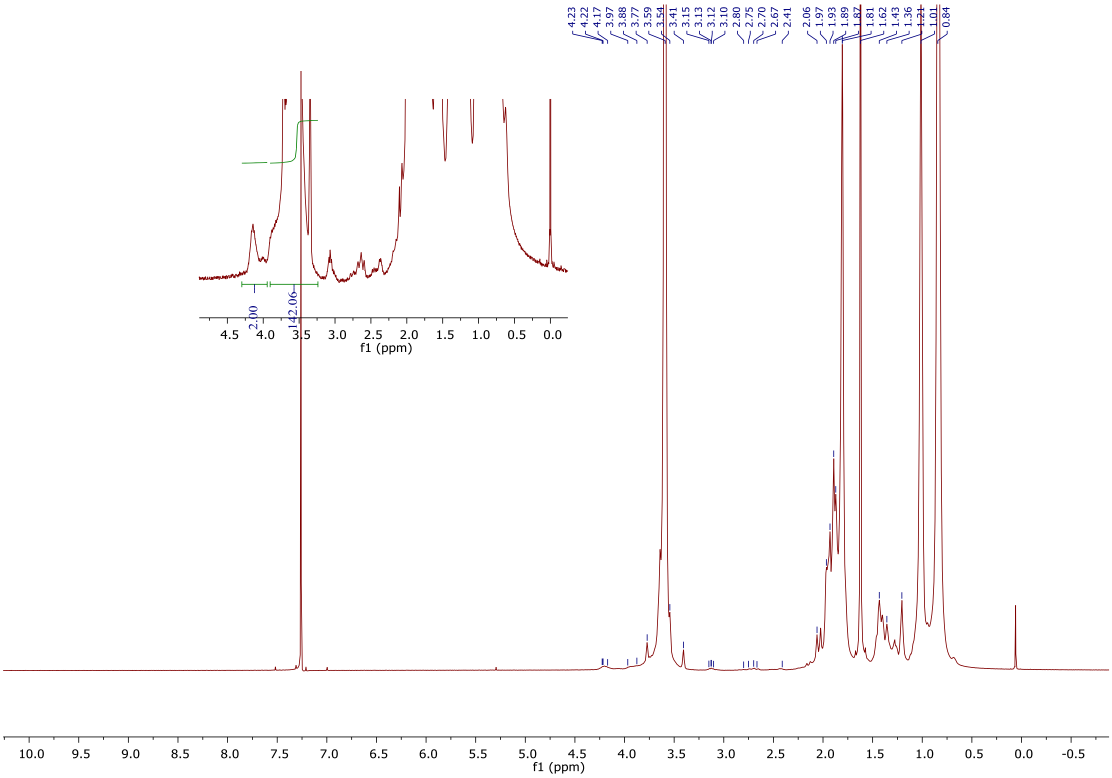

Fig. S162 ^{1}H NMR spectrum (400 MHz, $CDCl_3$) of $PMMA_{98}$-*co*-$PHCBI_2$. (The fraction of HCBI in copolymers is 1/(142.06/3+1)*100% ≈ 2%, therefore, the fraction of methyl methacrylate in copolymer is 98%.)

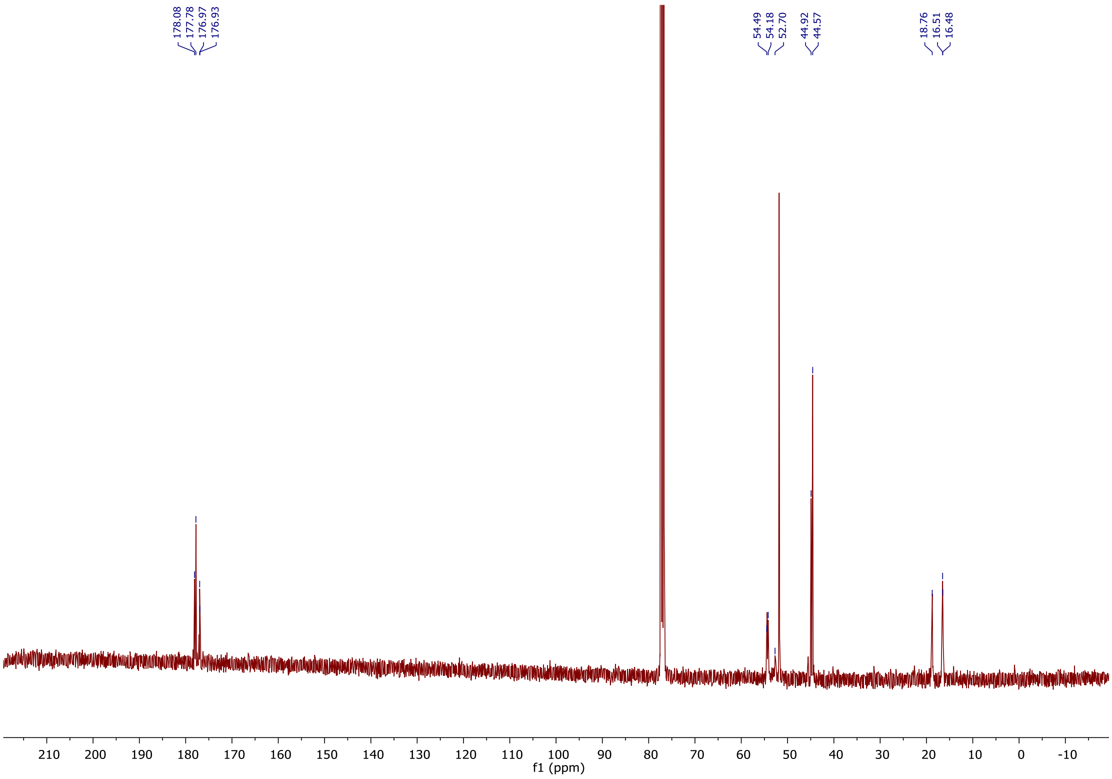

Fig. S163 ^{13}C NMR spectrum (101 MHz, $CDCl_3$) of PMMA$_{98}$-*co*-PHCBI$_2$.

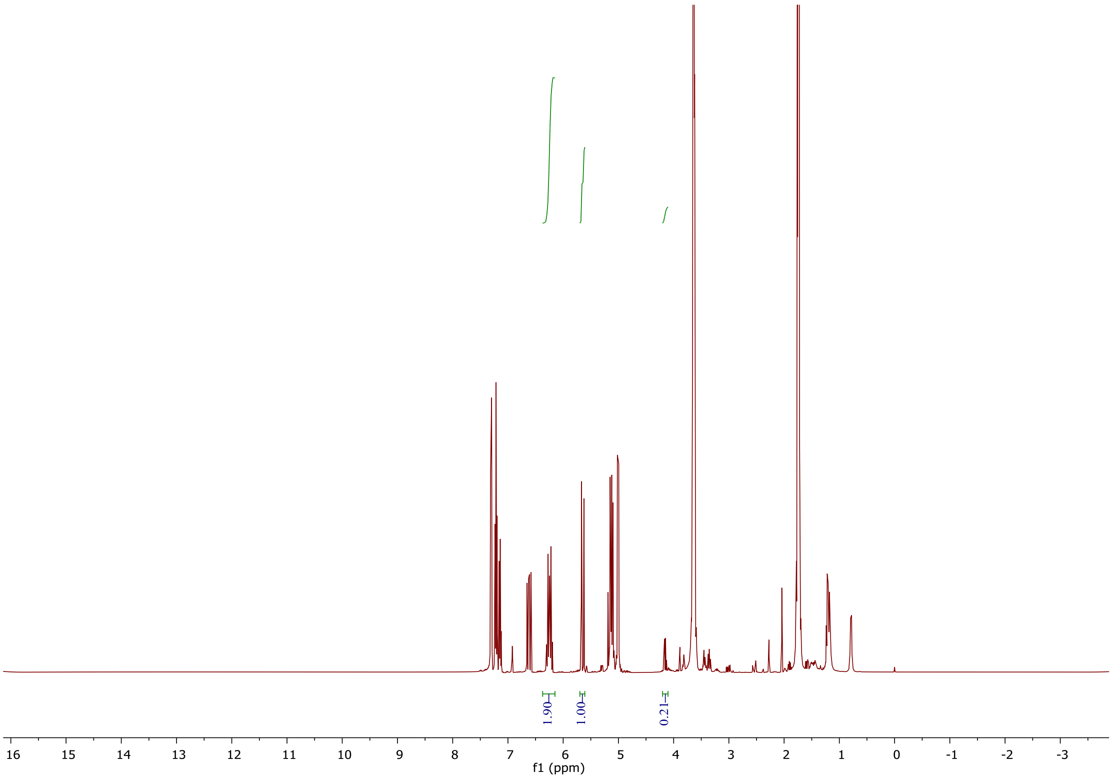

Fig. S164 ^{1}H NMR spectrum (400 MHz, $CDCl_3$) of reaction feed mixture for the synthesis of PS$_{34}$-*co*-PB$_{49}$-*co*-PHCBI$_{17}$. (The fraction of HCBI in feed is (0.21/2)/(1.9/2+0.21/2+1) ≈ 0.05. The fraction of styrene in copolymers is (1/(1.9/2+0.21/2+1) ≈ 0.49. Therefore, the fraction of butadiene in copolymer is 0.46.)

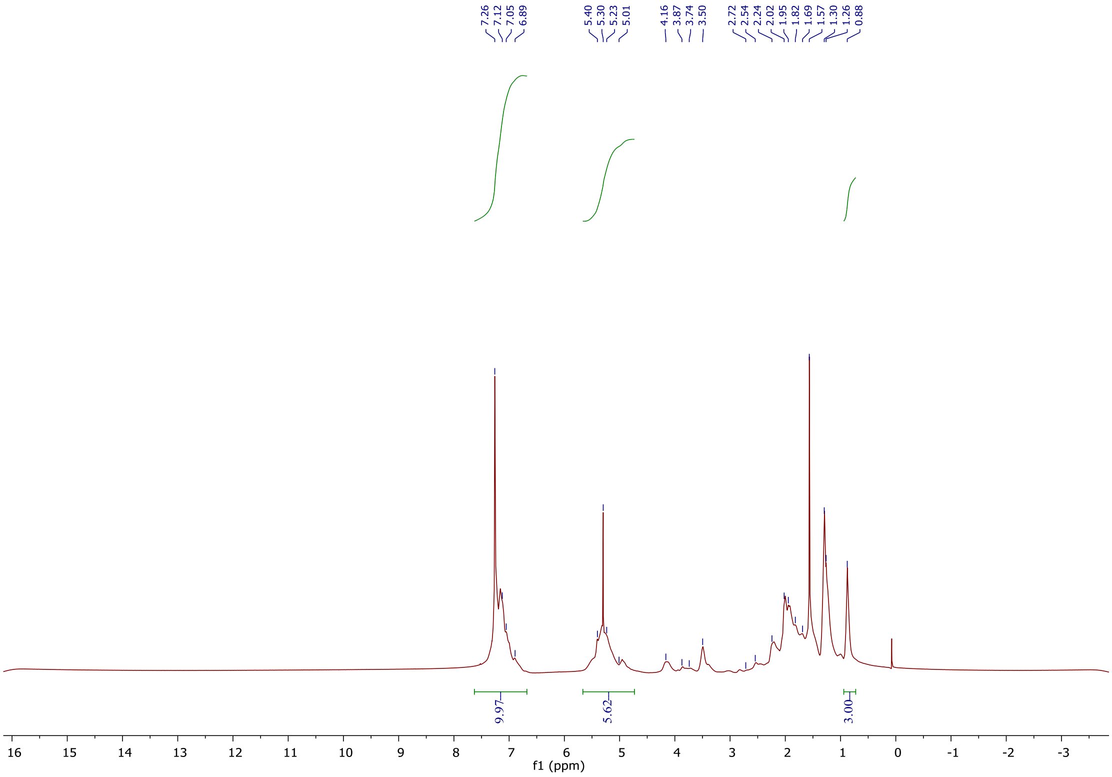

Fig. S165 ^{1}H NMR spectrum (400 MHz, $CDCl_3$) of PS_{34}-*co*-PB_{49}-*co*-$PHCBI_{17}$. (The fraction of HCBI in copolymers is 1/(9.97/5+5.62/2+1)*100% ≈ 17%. The fraction of styrene in copolymers is (9.97/5)/(9.97/5+5.62/2+1)*100% ≈ 34%. Therefore, the fraction of butadiene in copolymer is 49%.)

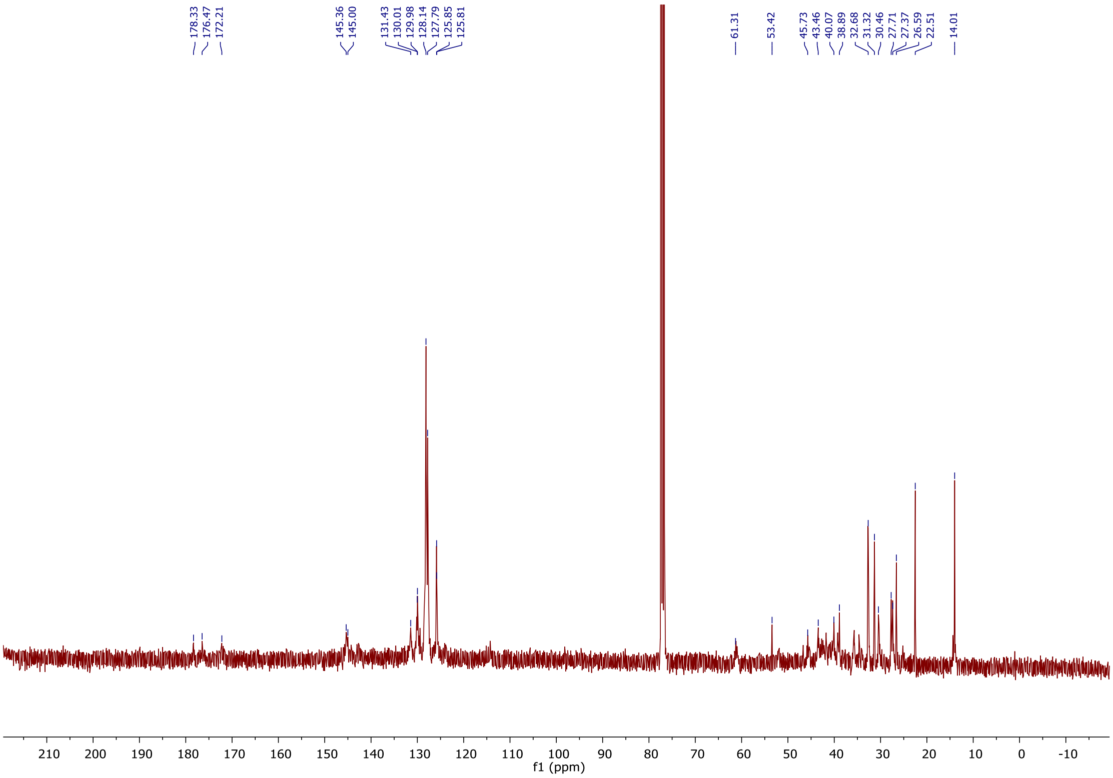

Fig. S166 ^{13}C NMR spectrum (101 MHz, $CDCl_3$) of PS_{34}-*co*-PB_{49}-*co*-$PHCBI_{17}$.

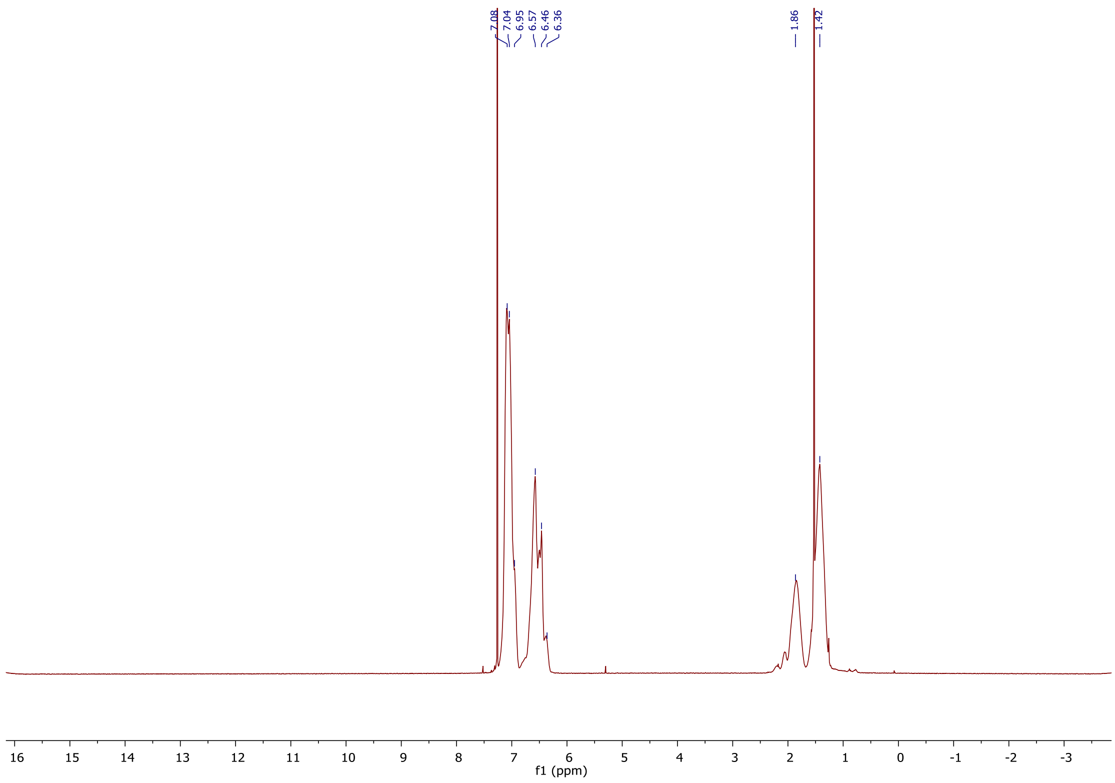

Fig. S167 ^{1}H NMR spectrum (400 MHz, $CDCl_3$) of PS.

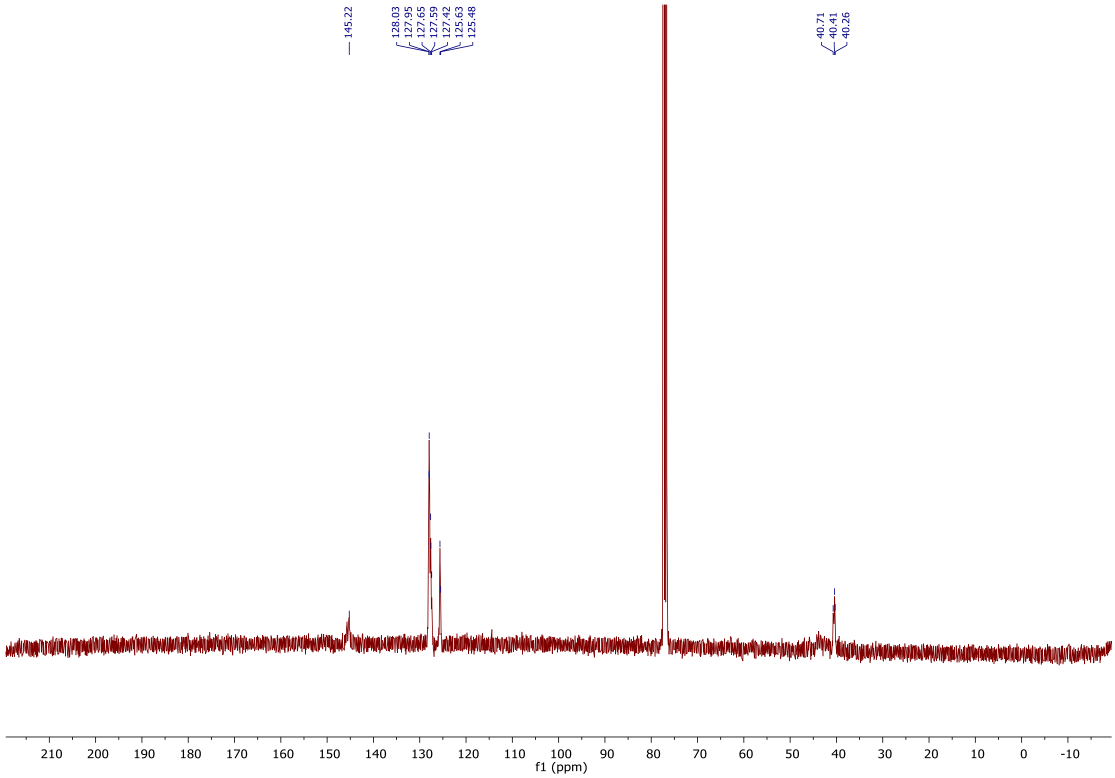

Fig. S168 ^{13}C NMR spectrum (101 MHz, $CDCl_3$) of PS.

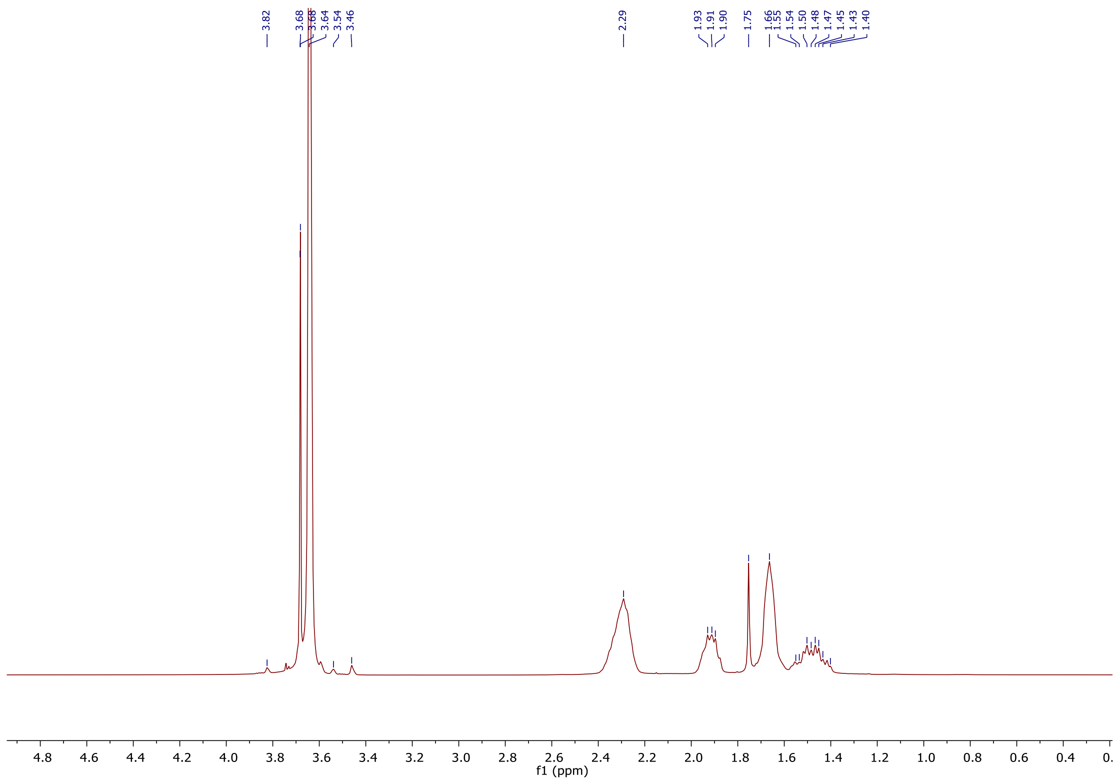

Fig. S169 ^{1}H NMR spectrum (400 MHz, $CDCl_3$) of PMA.

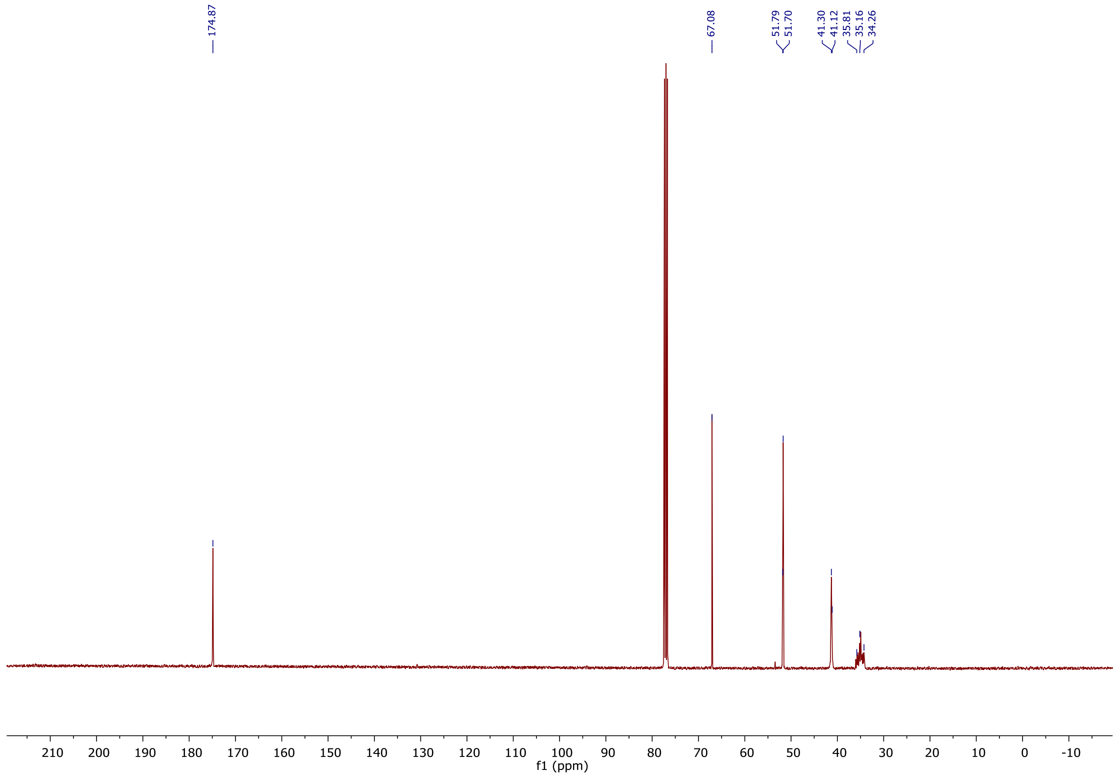

Fig. S170 ^{13}C NMR spectrum (101 MHz, $CDCl_3$) of PMA.

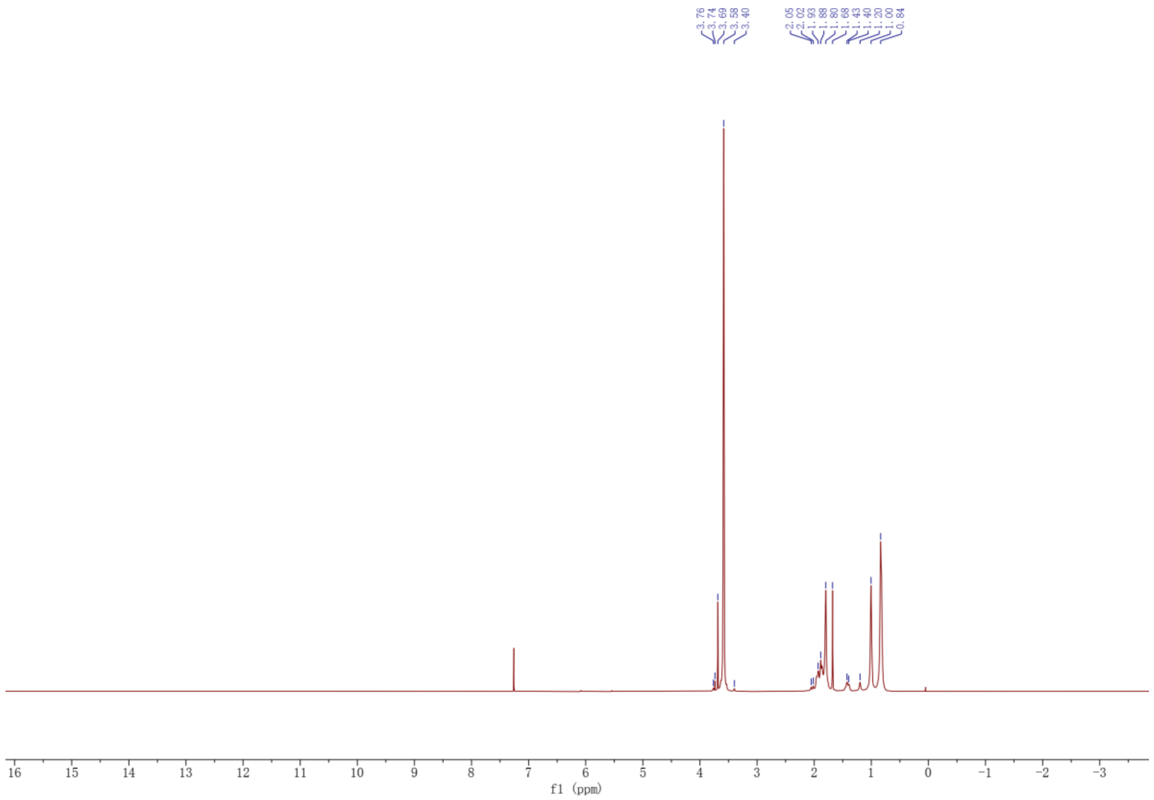

Fig.S171 ^{1}H NMR spectrum (400 MHz, $CDCl_3$) of PMMA.

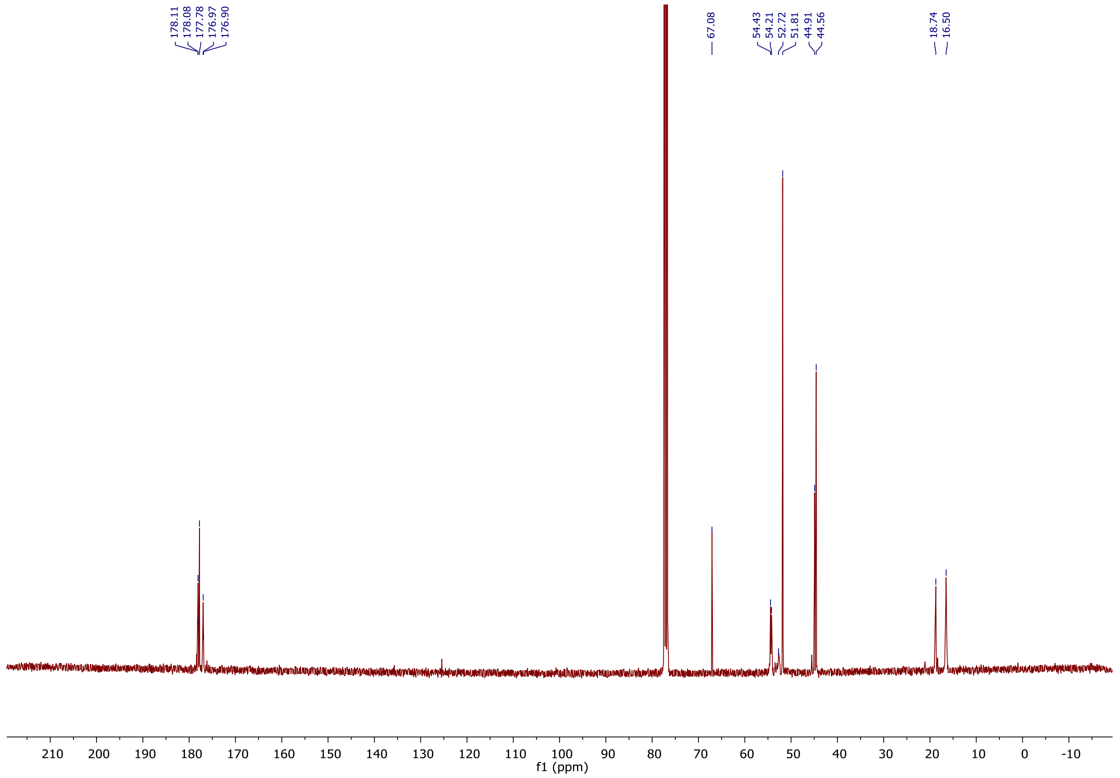

Fig. S172 ^{13}C NMR spectrum (101 MHz, $CDCl_3$) of PMMA.

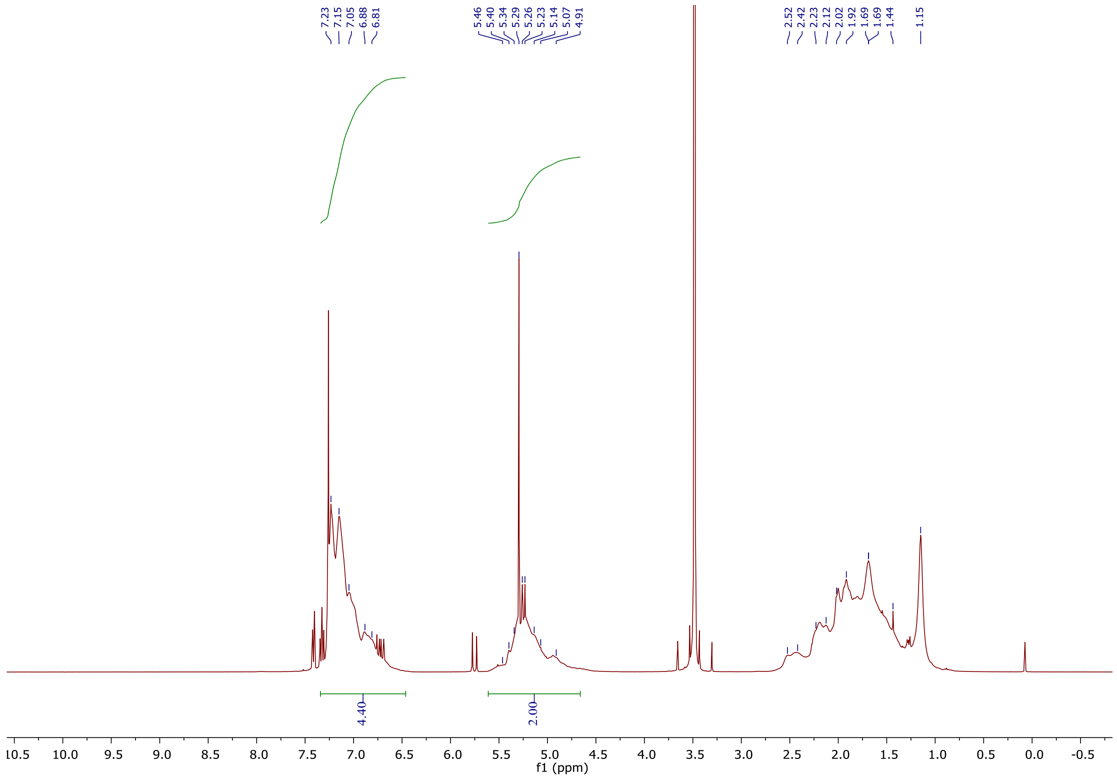

Fig. S173 ^{1}H NMR spectrum (400 MHz, $CDCl_3$) of PS_{47}-*co*-PB_{53}. (The fraction of styrene in copolymers is (4.4/5)/(4.4/5+1)*100% ≈ 47%. Therefore, the fraction of butadiene in copolymer is 53%.)

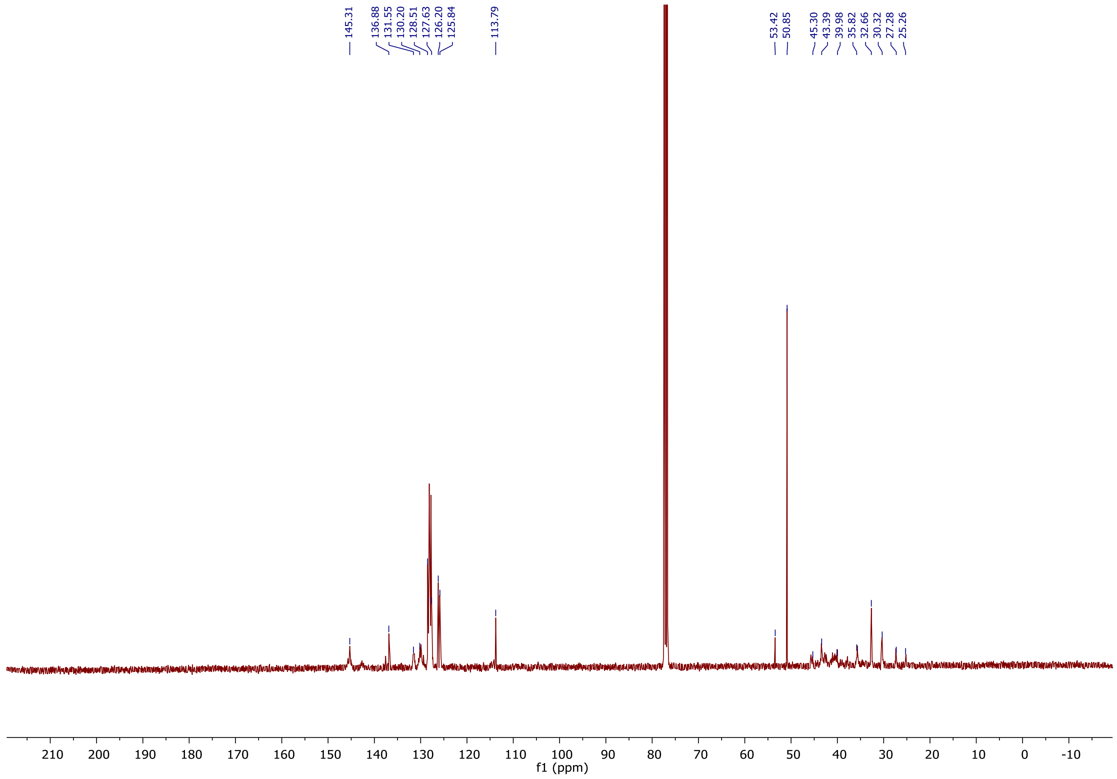

Fig. S174 ^{13}C NMR spectrum (101 MHz, $CDCl_3$) of PS_{47}-*co*-PB_{53}.

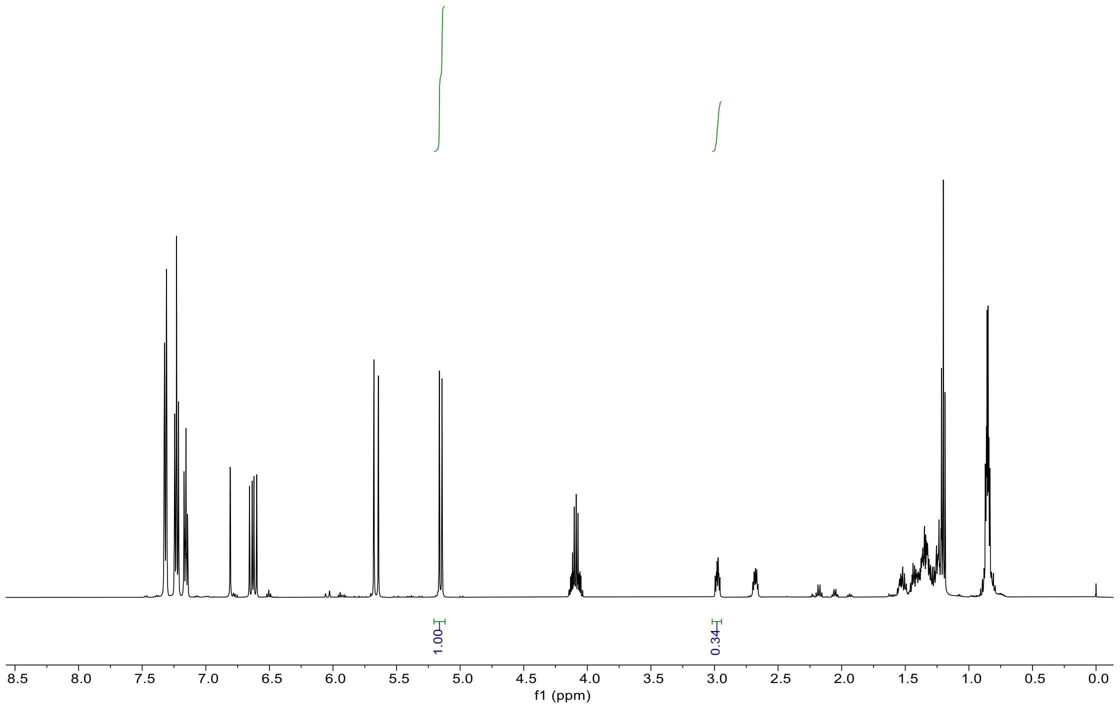

Fig. S175 ^{1}H NMR spectrum (500 MHz, $CDCl_3$) of reaction feed mixture for the synthesis of PS_{65}-*co*-$PCBO_{35}$. (The fraction of CBO in feed is 0.34/(1+0.34) ≈ 0.25. Therefore, the fraction of styrene in feed is 0.75.)

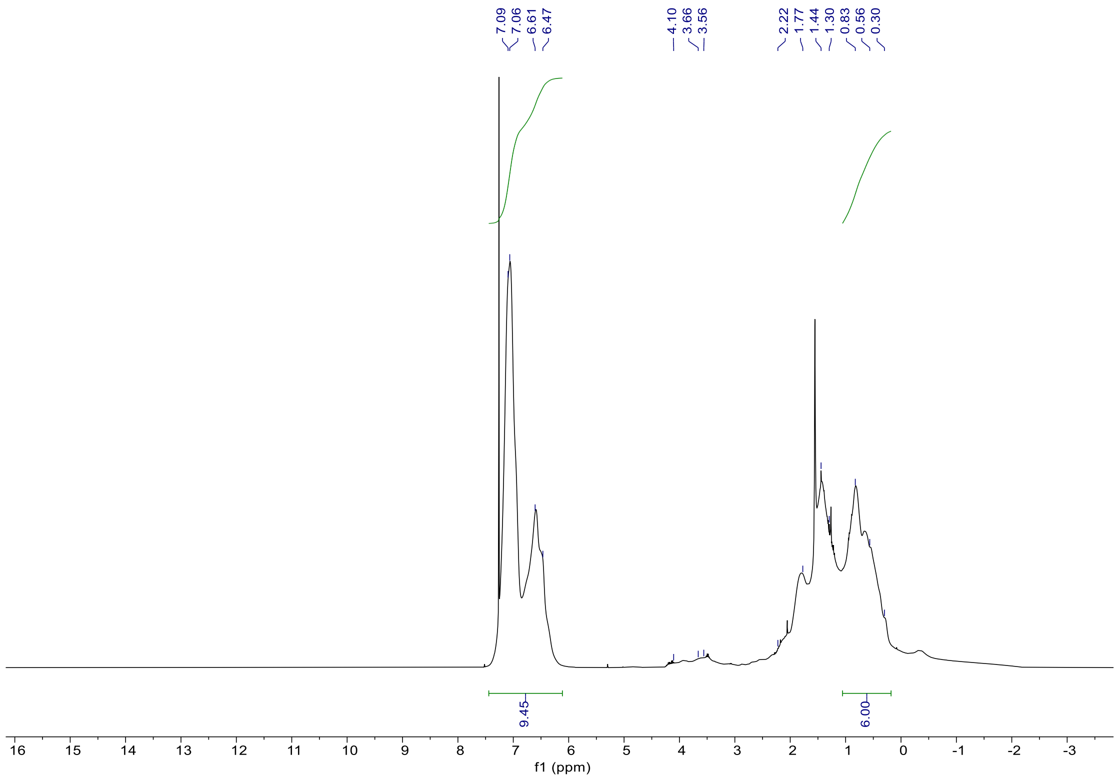

Fig. S176 ^{1}H NMR spectrum (400 MHz, $CDCl_3$) of PS_{65}-*co*-$PCBO_{35}$, (The fraction of styrene in copolymers is (9.45/5)/(9.45/5+1)*100% ≈ 65%. Therefore, the fraction of CBO in copolymer is 35%.)

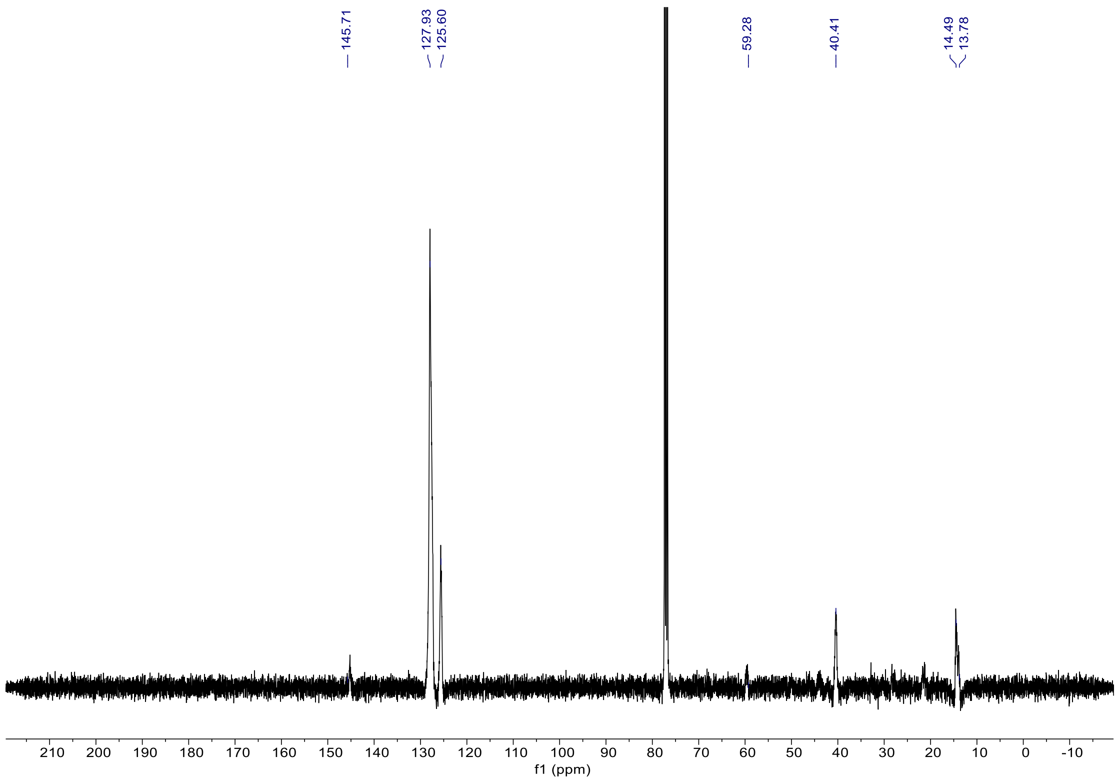

Fig. S177 ^{13}C NMR spectrum (101 MHz, $CDCl_3$) of PS_{65}-*co*-$PCBO_{35}$

Reference

1. Khayum, M. A.; Kandambeth, S.; Mitra, S.; Nair, S. B.; Das, A.; Nagane, S. S.; Mukherjee, R. & Banerjee, R. Chemically delaminated free-standing ultrathin covalent organic nanosheets. *Angew. Chem. Int. Ed.* **55**, 15604-15608 (2016).
2. Asadirad, A. M.; Boutault, S.; Erno, Z. & Branda, N. R. Controlling a polymer adhesive using light and molecular switch. *J. Am. Chem. Soc.* **136**, 3024-3027 (2014).
3. White paper as a reference www.waters.com/webassets/cms/library/docs/720005480en.pdf
4. Triandafillidi, I.; Nikitas, N.; Gkizis, P. L.; Spiliopoulou N. & Kokotos, C. G. Hexafluoroisopropanol-promoted or Brønsted acid-mediated photochemical [2+2] cycloadditions of alkynes with maleimides. *ChemSusChem* **15**, e202102441 (2022).
5. Snider, B. B.; Rodini, D. J.; Conn, R. S. E. & Sealfon, S. Lewis acid catalyzed reactions of methyl propiolate with unactivated alkenes. *J. Am. Chem. Soc.* **101**, 5283-5293 (1979).
6. Fineman, M. & Ross, S. D. Linear method for determining monomer reactivity ratios in copolymerization. *J. Polym. Sci.* **5**, 259−262 (1950).
7. Kelen, T. & Tudos, F. Analysis of the linear methods for determining copolymerization reactivity ratios. I. A new improved linear graphic method. *J. Macromol. Sci., Chem.* **9**, 1−27 (1975).
8. Scholten P. B. V.; Demarteau, J. ; Gennen, S. ; Winter, J. D. ; Grignard, B. ; Debuigne, A. ; Meier, M. A. R. & Detrembleur, C. Merging CO_2-based building blocks with cobalt-mediated radical polymerization for the synthesis of functional poly(vinyl alcohol)s. *Macromolecules*, **51**, 3379-3393 (2018).
9. Drean, M.; Guegan, P.; Detrembleur, C.; Jerome, C.; Rieger, J. & Debuigne, A. Controlled synthesis of poly(vinylamine)-based copolymers by organometallic-mediated radical polymerization. *Macromolecules*, **49**, 4817-4827 (2016).
10. Klein, I. M.; Husic, C. C.; Kovács, D. P.; Choquette, N. J.; Robb, M. J. Validation of the CoGEF Method as a Predictive Tool for Polymer Mechanochemistry. *J. Am. Chem. Soc.* **142**, 16364-16381 (2020).
11. Stauch, T.; Dreuw, A. Advances in Quantum Mechanochemistry: Electronic Structure Methods and Force Analysis. *Chem. Rev.* **116**, 14137-14180 (2016).
12. Caruso, M. M.; Davis, D. A.; Shen, Q.; Odom, S. A.; Sottos, N. R.; White, S. R.; Moore, J. S. Mechanically-Induced Chemical Changes in Polymeric Materials. *Chem. Rev.* **109**, 5755-5798 (2009).
13. Chen, Z.; mercer, J. A. M.; Zhu, X.; Romaniuk, J. A. H.; Pfattner, R.; Cegelski, L.; Martinez, T. J.; Bruns, N. Z.; Xia, Y. Mechanochemical Unzipping of Insulating Polyladderene to Semiconducting Polyacetylene. *Science* **357**, 475-479 (2017).
14. Ong, M. T.; Leiding, J.; Tao, H.; Virshup, A. M.; Martinez, T. J. First rinciples Dynamics and Minimum Energy Pathways for Mechanochemical Ring Opening of Cyclobutene. *J. Am. Chem. Soc.* **131**, 6377-6379 (2009).

15. Kryger, M. J.; Munaretto, A. M.; Moore, J. S. Structure-mechanochemical Activity Relationship for Cyclobutane Mechanophores. *J. Am. Chem. Soc.* **133**, 18992-18998 (2011).
16. Brown, C. L.; Bowser, B. H.; Meisner, J.; Kouznetsova, T. B.; Seritan, S; Martinez, T. J.; Craig, S. L Substituent Effects in Mechanochemical Allowed and Forbidden Cyclobutene Ring-Opeing Reactions. *J. Am. Chem. Soc.* **143**, 3846-3855 (2021).
17. Yang, Q. Z.; Huang, Z.; Kucharski, T. J.; Khvostichenko, D.; Chen, J.; Boulatov, R. A Molecular Force Probe *Nature Nanotech.* **4**, 302-306 (2009).
18. Ong, M. T.; Leiding, J.; Tao, H.; Virshup, A. M.; Mart ńez, T. J. First Principles Dynamics and Minimum Energy Pathways for Mechanochemical Ring Opening of Cyclobutene. *J. Am. Chem. Soc.* **131**, 6377-6379 (2009).
19. Hickenboth, C. R.; Moore, J. S.; White, S. R.; Sottos, N. R.; Baudry, J.; Wilson, S. R. Biasing reaction pathways with mechanical force. *Nature* **446**, 423-427 (2007).
20. Frisch, M. J.; Trucks, G. W.; Schlegel, H. B.; Scuseria, G. E.; Robb, M. A.; Cheeseman, J. R.; Scalmani, G.; Barone, V.; Mennucci, B.; Petersson, G. A.; et al. Gaussian 09 Revision A.01. 2009.
21. Halgren, T. A.; Lipscomb, W. N. The synchronous-transit method for determining reaction pathways and locating molecular transition states. *Chem. Phys. Lett.* **49**, 225-232 (1977).
22. Peng, C.; Bernhard Schlegel, H. Combining Synchronous Transit and Quasi-Newton Methods to Find Transition States. *Isr. J. Chem. 33*, 449-454 (1993).
23. Stauch, T.; Dreuw, A. On the use of different coordinate systems in mechanochemical force analyses. *J. Chem. Phys.* **143**, 074118 (2015).
